# Structural and functional analysis of the *Francisella* lysine decarboxylase as a key actor in oxidative stress resistance

**DOI:** 10.1038/s41598-020-79611-5

**Published:** 2021-01-13

**Authors:** Jan Felix, Claire Siebert, Julia Novion Ducassou, Jérôme Nigou, Pierre Simon Garcia, Angélique Fraudeau, Karine Huard, Caroline Mas, Céline Brochier-Armanet, Yohann Couté, Irina Gutsche, Patricia Renesto

**Affiliations:** 1 Institut de Biologie Structurale, Univ Grenoble Alpes, CNRS, CEA, IBS, 71 avenue des martyrs, 38044 Grenoble, France; 2 TIMC-IMAG UMR 5525 - CNRS, INP, Université Grenoble Alpes, Grenoble Cedex 9, France; 3 Université Grenoble Alpes, CEA, Inserm, IRIG, Grenoble, BGE France; 4 Institut de Pharmacologie et de Biologie Structurale, Université de Toulouse, CNRS, Université Paul Sabatier, Toulouse, France; 5 Univ Lyon, Université Lyon 1, CNRS, UMR5558, Laboratoire de Biométrie et Biologie Evolutive, 43 bd du 11 novembre 1918, 69622 Villeurbanne, France; 6 Department of Microbiology, Stress Adaptation and Metabolism in Enterobacteria Unit, ERL CNRS 6002, Institut Pasteur, 25-28 Rue du Dr Roux, 75015 Paris, France

**Keywords:** Phylogenetics, Biological techniques, Mass spectrometry, Microbiology techniques, Proteomic analysis, Structure determination

## Abstract

*Francisella tularensis* is one of the most virulent pathogenic bacteria causing the acute human respiratory disease tularemia. While the mechanisms underlying *F. tularensis* pathogenesis are largely unknown, previous studies have shown that a *F. novicida* transposon mutant with insertions in a gene coding for a putative lysine decarboxylase was attenuated in mouse spleen, suggesting a possible role of its protein product as a virulence factor. Therefore, we set out to structurally and functionally characterize the *F. novicida* lysine decarboxylase*,* which we termed LdcF*.* Here, we investigate the genetic environment of *ldcF* as well as its evolutionary relationships with other basic AAT-fold amino acid decarboxylase superfamily members, known as key actors in bacterial adaptative stress response and polyamine biosynthesis. We determine the crystal structure of LdcF and compare it with the most thoroughly studied lysine decarboxylase, *E. coli* LdcI*.* We analyze the influence of *ldcF* deletion on bacterial growth under different stress conditions in dedicated growth media, as well as in infected macrophages, and demonstrate its involvement in oxidative stress resistance. Finally, our mass spectrometry-based quantitative proteomic analysis enables identification of 80 proteins with expression levels significantly affected by *ldcF* deletion, including several DNA repair proteins potentially involved in the diminished capacity of the *F. novicida* mutant to deal with oxidative stress. Taken together, we uncover an important role of LdcF in *F. novicida* survival in host cells through participation in oxidative stress response, thereby singling out this previously uncharacterized protein as a potential drug target.

## Introduction

The Gram-negative bacterium *Francisella tularensis* is the etiological agent of tularemia^1^. This zoonotic disease can be contracted by humans through insect bites, contact with infected animal products, ingestion of polluted food or water and inhalation of contaminated aerosols. Respiratory tularemia resulting from aerosol uptake causes typical pneumonia symptoms with 30 to 60% fatality rate of untreated infections. Due to the ease of culture and the extremely high infectivity by airborne route, *F. tularensis* is considered as a dangerous bioweapon classified as a category A bioterrorism agent by the Centers for Disease Control and Prevention (CDC)^2^. Indeed, this bacterium, capable of surviving for weeks at low temperature in water, soil, grass or animal carcasses is one of the most infectious pathogens known because inhalation of as few as a dozen of organisms can suffice to cause illness and death. Yet, no licensed vaccine against tularemia is currently available, and the mechanisms underlying pathogenesis of *F. tularensis* are still largely unknown. The virulent strains are classified as *F. tularensis* subsp. *tularensis* and *F. tularensis* subsp. *holarctica*, whereas the closely related *F. novicida* is considered avirulent for humans and is therefore a suitable working model^3^.

The pathogenicity of *F. tularensis* mainly relies on the *Francisella* Pathogenicity Island (FPI)^4^, a gene cluster encoding 17 proteins comprising a type VI-like secretion system machinery (T6SS)^5,6^. The regulation of *Francisella* virulence genes is complex and poorly understood but requires the expression of the macrophage growth locus protein A (MglA)^7,8^. This major transcriptional regulator associates with the stringent starvation protein A (SspA) to form a heterodimer able to interact with RNA polymerase and whose stability is tightly linked to inorganic polyphosphate^9^. Another FPI transcriptional regulator essential for intracellular bacterial growth and virulence is the *Francisella* effector of virulence regulation (FevR also known as PigR) which physically interacts with the MglA/SspA complex^10^. MglA is also involved in the regulation of genes outside FPI and more specifically in the *Francisella* oxidative stress response^11^.

*Francisella* is a facultative intracellular pathogen whose replication inside macrophages is mostly admitted to be at the heart of the bacterial pathogenesis. However, this bacterium is also capable of invading many other cell types such as dendritic cells and neutrophils^1,12,13^. Murine models of intranasal infection with different *Francisella* species demonstrated that alveolar macrophages were predominantly infected at 4–24 h post-infection (hpi) and that neutrophils serve as a replicative niche accounting for at least 50% of *F. tularensis*-infected cells from day 3 post-infection. The intracellular life cycle exposes *Francisella* to oxidative stress upon bacterial uptake, temporary residence in the phagosomes, and escape into the host cytoplasm for replication. Indeed, as a defense mechanism for the clearance of phagocytosed microorganisms, both macrophages and neutrophils produce reactive oxygen species (ROS), such as superoxide anions (O_2_**·**^−^), hydrogen peroxide (H_2_O_2_) and hydroxyl radicals (OH**·**), which in turn trigger bacterial killing by causing damage to macromolecules including DNA, proteins and membrane lipids^14,15^. Several factors including for example catalase (KatG), superoxide dismutase (SodB, SodC) and peroxiredoxin (AhpC)^16–20^, have been identified as allowing *Francisella* to cope with such oxidative stress and thus contributing to intracellular bacteria survival. Another factor that makes bacteria more resistant to ROS killing is a lower iron content^21,22^ .

A previous report showed that a *F. novicida* transposon mutant with insertions in *cad*A (FTT_0406), which encodes a putative aspartate aminotransferase fold (AAT-fold) pyridoxal 5′-phosphate (PLP)-dependent lysine decarboxylase (hereafter referred to as LdcF), was attenuated in mouse spleen^23^. Its role as virulence factor was further hypothesized from the comparative bioinformatic analysis of *Francisella* strains exhibiting different levels of pathogenicity^24^. Considering the current knowledge on the other members of the superfamily of AAT-fold PLP-dependent basic amino acid decarboxylases and their recognized involvement in bacterial physiology, stress responses and virulence, we set out to investigate the structure and function of LdcF, using the *F. novicida* model as a practicable surrogate of *F. tularensis* for experimental studies^3^.

Bacterial AAT-fold PLP-dependent basic amino acid decarboxylases are grouped into a superfamily termed LAOdc because these enzymes decarboxylate lysine (LdcI, LdcC and LdcA), arginine (AdcI) and ornithine (OdcI and OdcC) into corresponding polyamines (cadaverine, agmantine and putrescine) while consuming protons and producing CO_2_^25–27^. The main role of LdcI, AdcI and OdcI (with I standing for acid stress-inducible LAOdcs) is to buffer the bacterial cytosol in acid stress response, while the primary function of LdcC, LdcA and OdcC is polyamine biosynthesis. As polycations, polyamines bind negatively charged macromolecules such as DNA, proteins and phospholipids^28,29^, thereby contributing to a remarkable diversity of processes such as DNA replication, gene expression, protein synthesis, stress and antibiotic resistance, siderophore synthesis, biofilm formation and virulence^30^. In this work, we investigate the genetic environment of *Francisella ldcF* and the evolutionary relationships of LdcF with the other superfamily members in the light of a recent exhaustive phylogenetic analysis of proteobacterial LAOdcs^26^. We determine its crystal structure and identify specific structural elements which distinguish LdcF from *E. coli* LdcI, the most thoroughly-studied Ldc. We consider functional implications of these structural differences, in particular in terms of nutrient stress response, and analyze the influence of *ldcF* inactivation on bacterial growth under a variety of stress conditions. These experiments demonstrate the involvement of LdcF in oxidative stress resistance in dedicated growth media, as well as in infected macrophages. Finally, a comparative mass spectrometry (MS)-based quantitative analysis of the proteome of the wild-type *F. novicida* versus the Δ*ldcF* mutant provides elements for the explanation of LdcF involvement in defense against oxidative stress, virulence and survival in macrophages. Taken together, this study provides a structural and functional characterization of *Francisella* LdcF. It uncovers the important role of this previously uncharacterized protein in survival in the host cells through participation in oxidative stress response, thereby identifying LdcF as a potential drug target. Finally, the differential proteomic analysis opens up further avenues for mechanistic investigations of the LdcF mode of action.

## Results

### Bioinformatic analysis of LdcF and the *ldc* genetic environment

**Figure 1:**
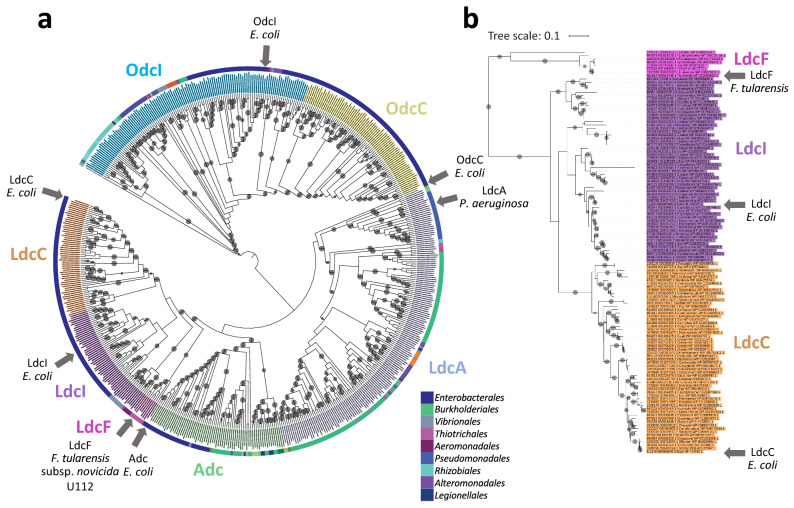
Phylogenetic position of LdcF sequences within the wing-domain containing LAOdc family. (**a**) Tree showing the relationships of 553 WING-containing AAT-fold decarboxylase sequences. The tree is a cladogram, meaning that the length of the branches has no evolutionary significance. The cladogram is rooted according to Carriel et al.^26^. The colour of leaves corresponds to the LdcI, LdcC, AdcI, OdcC, OdcI, and LdcA subfamilies^26^. The group corresponding to *Francisellaceae* sequences (referred as to LdcF) is indicated in pink. *F. tularensis*, *E. coli*, and *P. aeruginosa* sequences are indicated by grey arrows. Grey circles at branches correspond to ultrafast bootstrap values > 95%. The taxonomy of species (Class) is represented by a coloured strip. (**b**) Phylogram corresponding to the LdcI, LdcC, and LdcF subtree (122 sequences). The scale bar corresponds to the average number of substitutions per site. The length of branches is proportional to genetic divergence.

A genomic survey of 4,467 prokaryote complete proteomes identified a single LAOdc sequence in *Francisellaceae*, which we termed LdcF. LdcF sequences found in *Francisellaceae* strains display a high level of sequence identity (83%), and contain four functional regions, corresponding to a wing domain (Pfam ID: PF03709), a PLP-binding domain and a AAT-like domain (both corresponding to Pfam ID: PF01276), and a C-terminal domain (Pfam ID: PF03711) (Supplementary Fig. S1). This corresponds to the canonical organization of the wing-containing LAOdc superfamily^26^. Based on sequence comparison, LdcF proteins appear more similar to *Escherichia coli* LdcI (52.65% identity) and LdcC (48.18%) than to *Pseudomonas aeruginosa* LdcA (36.44%), *E. coli* AdcI (33.03%), *E. coli* OdcI (30.34%) and *E. coli* OdcC (27.28%). The phylogenetic analysis of 553 wing-containing LAOdcs present in 1,904 representative proteomes confirmed the specific relationship of *Francisellaceae* LdcF with the LdcI/C family (Fig. 1a and Supplementary Fig. S2). More precisely, *Francisellaceae* LdcF grouped robustly with a sequence from *Legionella fallonii* at the base of the clade corresponding to LdcI/C (ultrafast bootstrap = 100%, Fig. 1b). However, the long stem of the LdcI/C cluster reflects the large evolutionary distance between LdcF and LdcI/C sequences and thus their relative high divergence (Fig. 1b).

**Figure 2:**
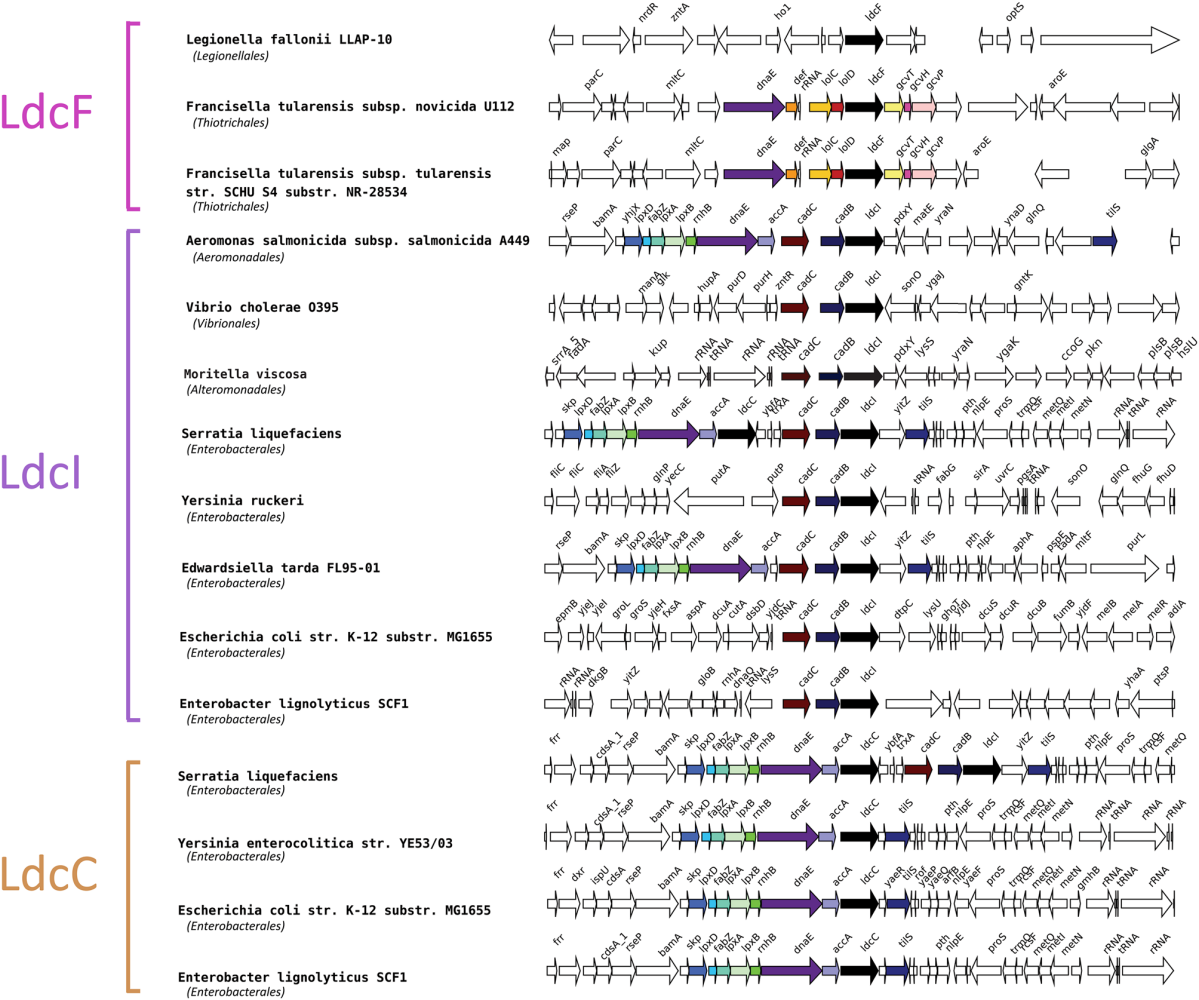
Genomic context of LdcI, LdcC, and LdcF coding genes (black arrows) in a subsample of representative species. Other conserved neighbour genes are highlighted with colour. The taxonomy of species (Class) is indicated in brackets.

Therefore, LdcF sequences could represent a new Ldc family. In line with this hypothesis, the genomic context of *ldcF* is very different of those of *ldcI* and *ldcC* (Fig. 2). In particular, in many genomes, *ldcI* and *ldcC* are present in vicinity of *lpxD, fabZ, lpxA, lpxB, rnhB, dnaE* and *accA* genes involved in lipid synthesis and DNA replication. Furthermore, most *ldcI* are clustered with *cadB* and *cadC*, encoding the lysine-cadaverine antiporter and the transcriptional regulator of the *cadBA* operon respectively. In contrast, *Francisellaceae ldcF* are surrounded by *lolC* and *lolD* on the one hand, and *gcvT*, *gcvH*, and *gcvP* on the other hand, involved in lipid transport and glycine cleavage system, respectively. Altogether, these data underlie differences between LdcF and other LAOdcs. Accordingly, LdcF may constitute a new family of LAOdcs phylogenetically related to LdcI/C but presenting a different genomic context.

### Structural characterization of *Francisella novicida* LdcF

**Figure 3:**
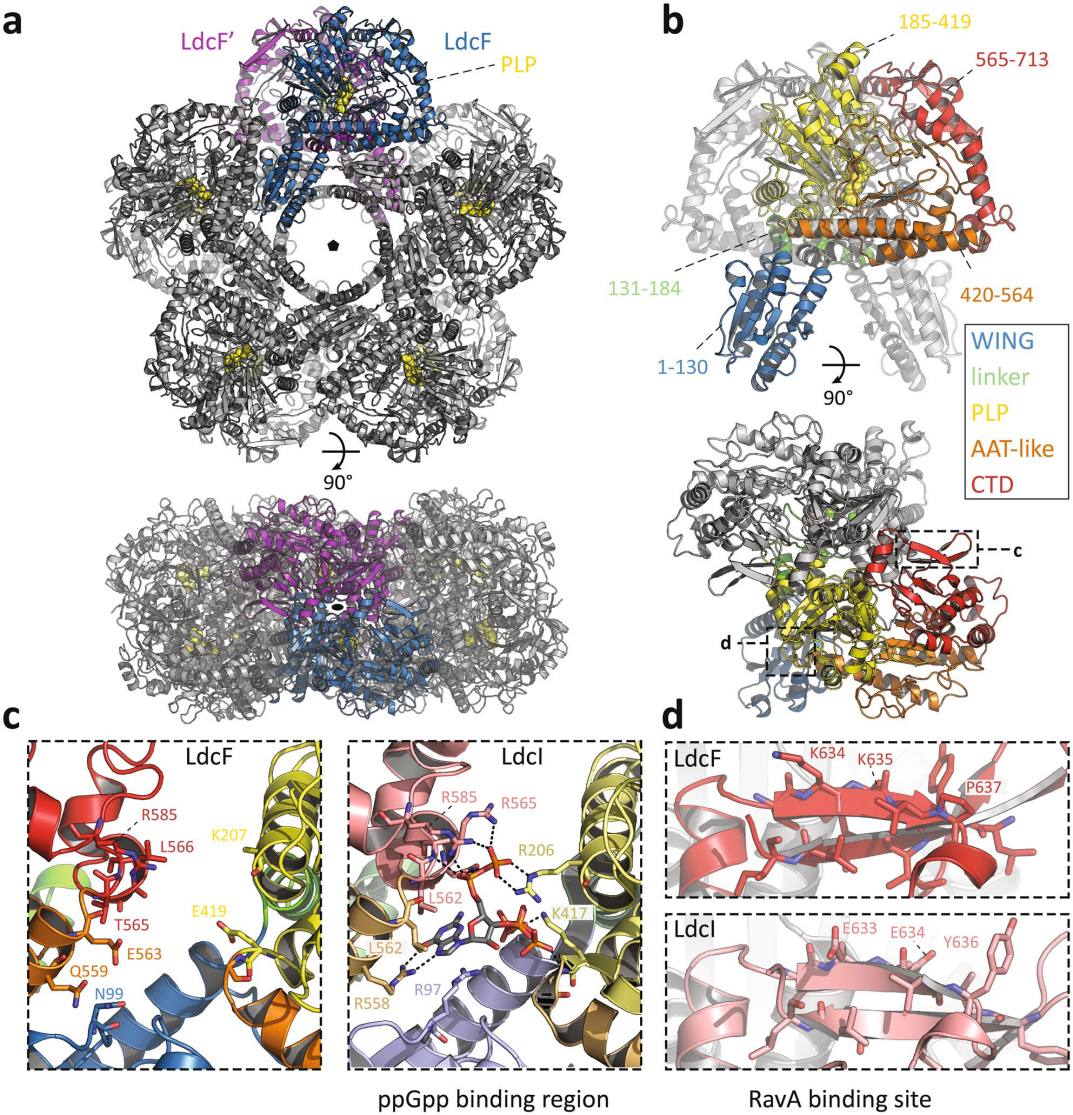
Crystal structure of the *F. novicida* lysine decarboxylase LdcF. (**a**) Front (upper panel) and side view (lower panel) of decameric LdcF, with one highlighted dimer coloured blue and purple, while other dimers are coloured light and dark grey. The covalently bound pyridoxal phosphate (PLP) cofactor is shown as yellow spheres. (**b**) Front (upper panel) and side view (lower panel) of an LdcF dimer extracted from the decamer shown in (**a**). In one monomer, different domains are coloured according to a rainbow scheme (WING domain: blue, linker: green, PLP-binding domain: yellow, AAT-like domain: orange, C-terminal domain: red), with accompanying annotated amino acid residue ranges. (**c**) Comparison between the AAT-like domains (termed ppGpp binding domain in *E. coli* LdcI) of *F. novicida* LdcF (left) and *E. coli* LdcI (right). Residues of *E. coli* LdcI involved in ppGpp binding, and the corresponding residues in the AAT-like domain of *F. novicida* LdcF are annotated and shown as sticks. Domains are coloured as in (**b**), but using lighter tints for *E. coli* LdcI. (**d**) Comparison between the RavA-binding site in *E. coli* LdcI, and the corresponding region in *F. novicida* LdcF. Residues of *E. coli* LdcI involved in RavA binding, and the corresponding residues of *F. novicida* LdcF are annotated and shown as sticks.

Having shown that the LdcF family is distinct from LdcI/C and LdcA, and considering that the structures of *E. coli* LdcI, *E. coli* LdcC and *P. aeruginosa* LdcA solved by either X-ray crystallography or cryo-EM are available, we decided to gain structural insights into *F. novicida* LdcF and to compare its structure with those of the related families. LdcF was purified to homogeneity, and its lysine decarboxylase activity was assessed at pH 6.5 and 37 °C using a 2,4,6-trinitrobenzensulfonic acid colorimetric assay^31^(see “Methods”). The initial activity rate in nanomoles cadaverine produced per minute and per microgram of enzyme was measured to be ~ 5 nmoles cadaverine min^−1^ µg^−1^ LdcF (Supplementary Fig. S3). The observation that this activity rate is 30 times smaller than that of *E. coli* LdcI at the same conditions^31,32^ may indicate that, similarly to LdcI and related enzymes^33,34^, optimal LdcF activity is pH, salt, and temperature-dependent. We were able to determine the structure of *F. novicida* LdcF from X-ray diffraction data collected to a resolution of 3.4 Å (Supplementary Table S1 and Supplementary Fig. S4). The structure was solved by molecular replacement (MR), using the crystal structure of the decameric *E. coli* LdcI (PDB ID: 3N75)^32^ as a starting model (see “Methods”). All Ldcs are pentamers of dimers arranged around a central pore, thereby forming a D5-symmetric decamer^32,35,36^. The LdcF crystal structure contains one LdcF pentamer in the crystallographic asymmetric unit, while an LdcF decamer is generated by a two-fold crystallographic symmetry axis perpendicular to the pentamer pore (Fig. 3a).

**Figure 4:**
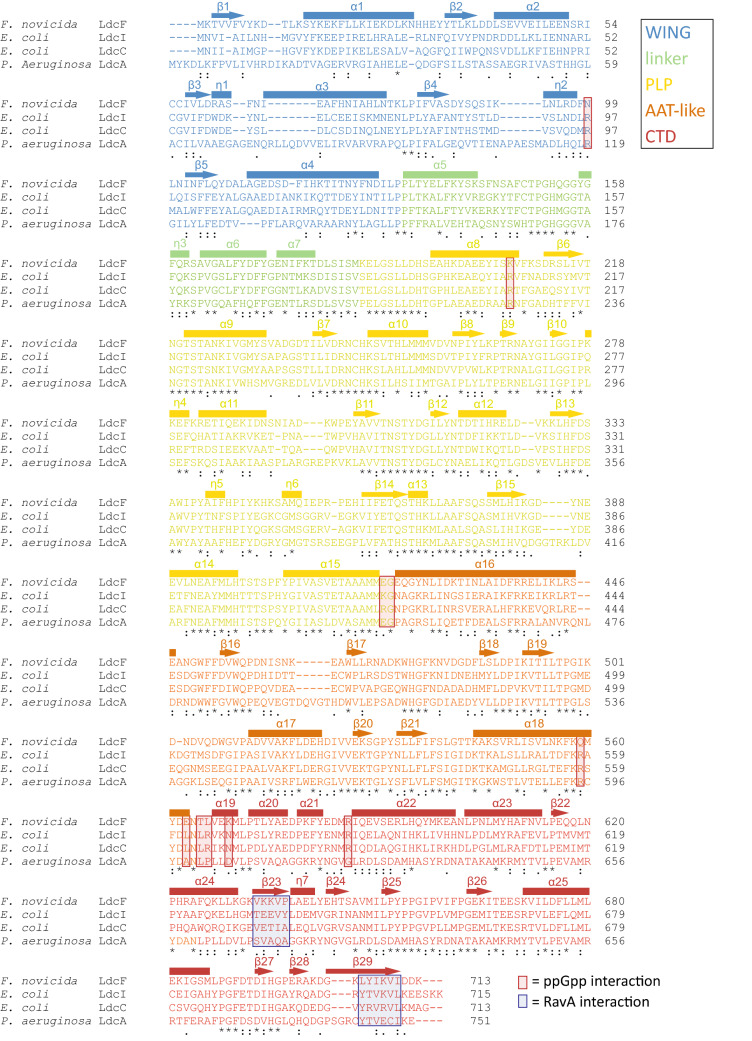
Alignment of *F. novicida* LdcF, *E. coli* LdcI and LdcC, and *P. aeruginosa* LdcA using Clustal Omega. Partially and fully conserved residues are annotated with ‘:’ and ‘*’ respectively. Domains are coloured according to a rainbow scheme (WING domain: blue, linker: green, PLP-binding domain: yellow, AAT-like domain: orange, C-terminal domain: red), and secondary structure elements are annotated. ppGpp and RavA-interaction sites are highlighted using red and blue transparent boxes respectively.

In agreement with the relationship between LdcF and LdcI families disclosed by the phylogenetic analysis and the relatively high level of sequence conservation (see above and Fig. 4), structural alignment between *F. novicida* LdcF and *E. coli* LdcI dimers extracted from their respective decameric crystal structures demonstrates a high overall similarity, with a root-mean-square-deviation (RMSD) of 1.043 Å over 1,223 aligned atoms. Like LdcI and other LAOdcs, the LdcF monomer is organized in three different structural domains (Fig. 3b): A N-terminal wing domain involved in stabilization of the ring assembly though inter-dimer contacts (residues 1–130), a central core domain which contains a covalently bound PLP cofactor (residues 131–564) and a C-terminal domain (residues 565–713), which partially constitutes an entry channel into the active site. The core domain (Fig. 3b) encompasses a linker region (residues 113–184), a PLP-binding domain consisting of a seven-stranded β-sheet surrounded by eight α-helices (residues 185–419), and an AAT-like domain which harbors an antiparallel four-stranded β-sheet and three α-helices near the dimerization interface (residues 420–564).

While the overall structures of LdcF and LdcI are very similar, some notable differences were found in both the AAT-like and the C-terminal domains. In *E. coli* Ldcs, the AAT-like domain is referred to as the ppGpp-binding domain due to its interaction with the stringent response alarmone ppGpp, which causes a strong inhibition of the lysine decarboxylase activity. The ppGpp binding site was actually discovered serendipitously upon building of the *E. coli* LdcI atomic model into the X-ray crystallography map, because under conditions of LdcI overexpression and purification used, the strongly-bound ppGpp was co-purified with LdcI^32^. Later, the enzymatic activity of both *E. coli* LdcI and LdcC was shown to be strongly inhibited by ppGpp^37^. In the case of LdcF, no additional density was present in the corresponding site. Moreover, a comparison of the ppGpp binding pocket in LdcI with the equivalent region in LdcF (Fig. 3c) revealed that, despite the overall high sequence conservation, only two out of 10 ppGpp-interacting residues are conserved between the two proteins. More importantly, 7 of the amino acid substitutions in LdcF result either in a change in charge or polarity, or in a change from hydrophobic to polar or vice versa, revealing that, contrary to LdcI and LdcC but similarly to *P. aeruginosa* LdcA^35^, LdcF is most likely not inhibited by ppGpp.

The C-terminal domain of the *E. coli* LdcI but not LdcC is known to interact with the MoxR AAA + ATPase RavA. The molecular determinant of the LdcI-RavA interaction resides in the C-terminal two-standed β-sheet of LdcI^36,38^ which was shown to be specifically evolved for RavA binding, contrary to OdcIC, AdcI, LdcA and even the closer related LdcC^35,36^. This LdcI-specific interaction leads to the formation of a huge cage-like LdcI-RavA complex^38–40^ proposed to enable enterobacteria, such as *E. coli*, *Salmonella* and *Vibrio*, to withstand acid stress even under conditions of nutrient deprivation eliciting stringent response^41^. Indeed, interaction with RavA was shown to maintain LdcI enzymatic activity upon starvation by preventing ppGpp binding to LdcI particles engaged in the LdcI-RavA complex^41^. Based on a medium-resolution cryo-EM structure of LdcI cross-linked with the LdcI-binding domain of RavA^36^, residues Glutamate 634 (E634), Tyrosine 636 (Y636) and Tyrosine 697 (Y697) are likely to be key players in the LdcI-RavA interaction. These residues are substituted in LdcF by Lysine (K635), Proline (P637) and Glutamate (E698) residues respectively, resulting in an impairment of a putative RavA interaction (Fig. 3d). This result is consistent with the absence of an orthologue of RavA in the *Francicellae* genome and further highlights the specific evolutionary tailoring of LdcI for RavA binding.

### In vitro phenotypic analysis of the *ΔldcF* mutant

**Figure 5:**
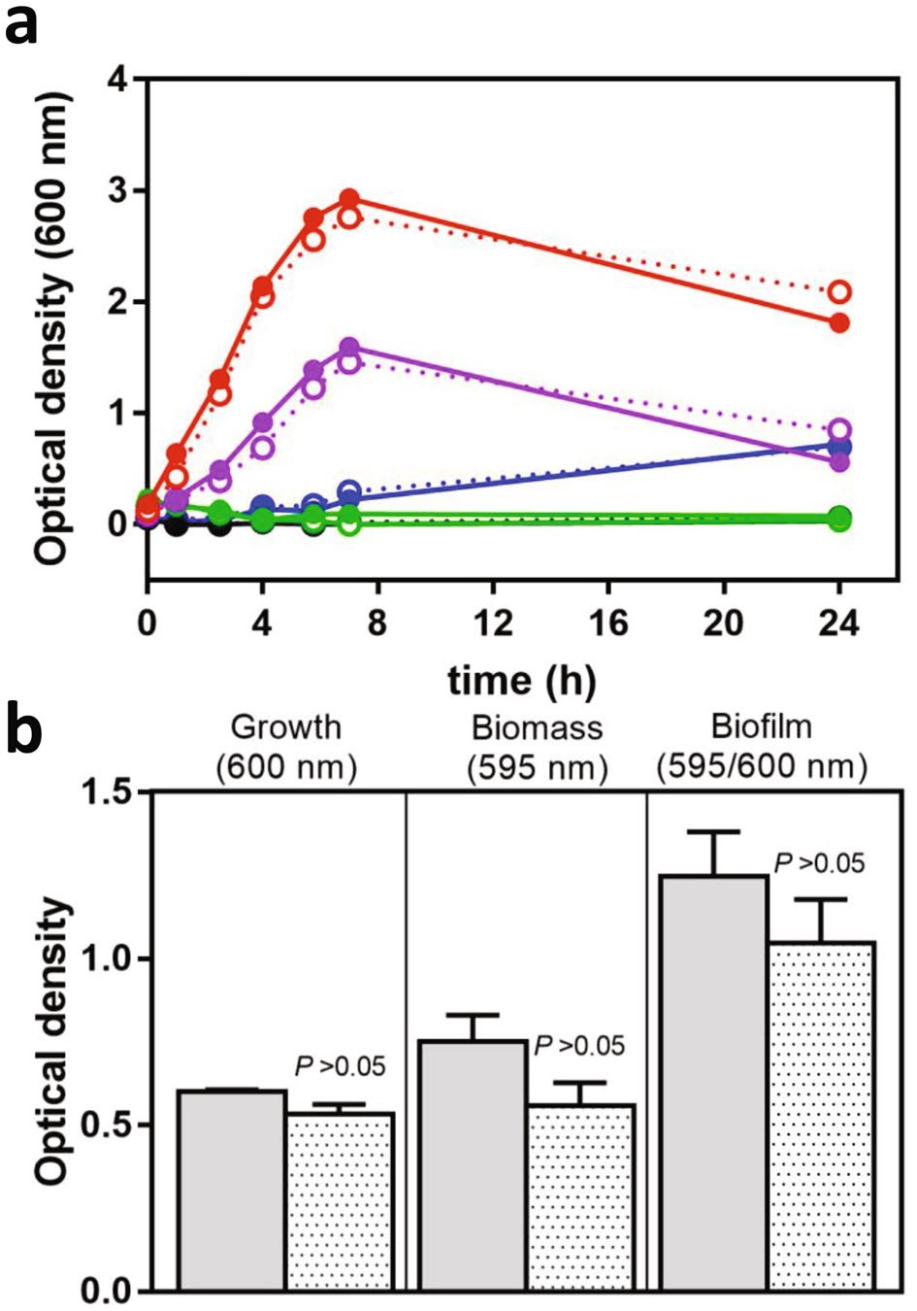
Growth and biofilm formation of *F. novicida*. (**a**) *F. novicida* WT (solid lines) and Δ*ldc*F (dotted lines) were grown under shaking at 37 °C in MMH adjusted at pH 2.5 (green), pH 4 (blue), pH 6.6 (red), pH 8 (purple) or pH 10 (black) and the bacterial growth was monitored by OD_600nm_ measurement. Results are representative of three independent trials. (**b**) *F. novicida* WT (grey columns) and Δ*ldcF* (dotted columns) were grown for 24 h under static conditions at 37 °C in a 96-wells plates. The bacterial growth was evaluated by measurement of OD_600nm_ and the biofilm biomass was further determined by OD_595nm_ after Crystal violet staining. This graph corresponds to mean ± s.e.m. of three independent experiments, with at least 4 technical replicates each.

The physiological significance of LdcF was investigated through the construction of a *F. novicida* FTN_0504 deletion mutant (Δ*ldcF*). Before proceeding with a comprehensive phenotypic analysis, we checked whether *ldcF* deletion affected bacterial fitness. When grown on PolyViteX-enriched chocolate agar (PVX-CHA) plates, *F. novicida* wild-type (WT) and Δ*ld*c*F* displayed similar colony morphology (Supplementary Fig. S5a). Accordingly, bacterial division and metabolism of both strains were found unchanged whether the protein was expressed or not (Supplementary Figs. S5b–d, S6). We then investigated a putative role of LdcF in bacterial tolerance to acidic pH exposure for 1 h but observed no difference between the WT and the deletion mutant (Supplementary Fig. S7). Bacterial growth was then examined in liquid Modified Mueller–Hinton (MMH) medium previously adjusted at different pH values ranging from 2.5 to 10 (Fig. 5a). Under all conditions tested, the replication curves for the WT and the deletion mutant were strictly similar. No growth was observed for the extreme acidic or alkaline pH values tested, while in the range of pH values from 4 to 8, bacteria grew and reproduced best at pH 6.6. At the 24 h time point the survival of bacteria was further evaluated by plating serial dilutions of each bacterial suspensions on PVX-CHA plates. For each pH tested, comparable numbers of colony forming units (cfu) were found for both strains, thus confirming that bacterial viability was not altered upon *ldcF* deletion (Supplementary Table S2). The replication rate of both strains was also found identical at 25 °C or at 37 °C, which correspond to the temperatures in tick and mammal hosts, respectively (Supplementary Fig. S8).

Besides growth fitness and acid stress response, another physiological process in which polyamine products of LAOdcs are likely to be involved is biofilm formation^27,30,42–44^. Yet, as assessed by crystal violet staining, no significant difference between the amount of biofilm produced by *F. novicida* WT and Δ*ldcF* strains could be documented (Fig. 5b). We also investigated whether LdcF activity is promoting antibiotic resistance by determining the minimum inhibitory concentrations (MICs) that were found unchanged for either ciprofloxacin (0.064 µg/mL ; *n* = 3) or gentamicin (1 µg/mL ; *n* = 3). In addition, we examined the rate at which these antibiotics kill bacteria – the minimum duration for killing (MDK) metric—as a quantitative indicator of antibiotic tolerance^45,46^. Again, the MDK_99_ values corresponding to the time required to kill 99% of the bacterial populations including WT, Δ*ldcF* and Δ*ldcF*-complemented (Δ*ldcF::ldcF*) strains exposed either to ciprofloxacin (Supplementary Fig. S9a) or to gentamicin (Supplementary Fig. S9b) were very similar, thus discarding the involvement of LdcF in antibiotic tolerance. This result was confirmed by the Minimal Bactericidal Concentration (MBC)/MIC ratios found to be below 32, i.e. the value defined by the Clinical Laboratory Standards Institute (CLSI) guidelines as the tolerance threshold^47^.

**Figure 6:**
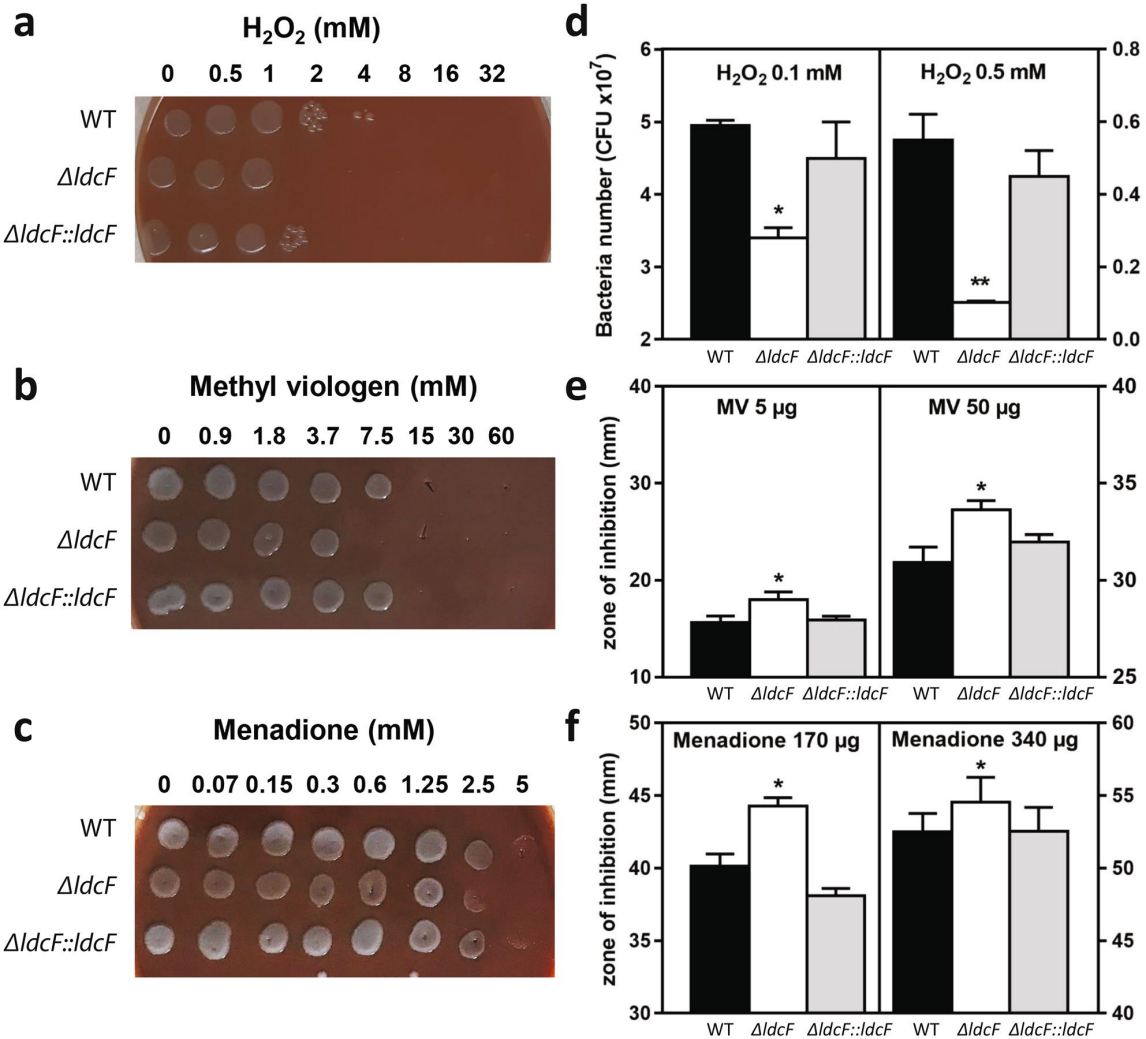
Sensitivity of *F. novicida* to oxidative stress. Exponential growth phase bacteria diluted in MMH were exposed to increasing concentration of (**a**) H_2_O_2_ (**b**) methyl viologen or (**c**) menadione for 1 h at 37 °C and 3 µl of the cell suspensions were spotted on PVX-CHA plates. These pictures are representative of at least 3 distinct experiments performed in duplicate each. The antibacterial activity of oxidative compounds was also quantified by (**d**) cfu counting from a cell suspension containing 10^8^ bacteria incubated for 1 h under shaking in presence of H_2_O_2_ or by disk diffusion assays with (**e**) methyl viologen or menadione (**f**) as detailled in materials and methods section. Histograms correspond to the mean ± s.e.m. of at least 3 distinct experiments performed in duplicate (*P < 0.05, **P < 0.01).

The same set of strains was then tested for susceptibility to oxidative stress. Interestingly, results obtained from spot plating assays indicated that LdcF significantly contributed to survival of bacteria exposed to hydrogen peroxide or to the redox-cycling drugs methyl viologen (MV) and menadione (MD). The ∆*ldcF* mutant was indeed found systematically less resistant to ROS exposure than the other strains, whereas complementation restored the WT phenotype (left panels in Fig. 6). Under such experimental conditions, and while the incubation of Δ*ldcF* with MD was accompanied with a moderate but reproducible inhibition of growth (Fig. 6c), MV (Fig. 6b) was found even more efficient than H_2_O_2_ (Fig. 6a). The enhanced sensitivity of the LdcF-deleted strain to H_2_O_2_ was accurately confirmed by a lower number of cfu when Δ*ldcF* was exposed to this reagent as compared to the value obtained with the WT (Fig. 6d). Because cfu counting is a time-consuming approach not fully appropriate to evaluate the effect of compounds on short incubation periods, the extent of ∆*ldcF* susceptibility to MV and MD was further validated through the disk diffusion assays. Thus, the diameter of inhibition zone, which is related to the susceptibility of the isolate, was significantly higher for ∆*ldcF* than for the WT strain when the disks were impregnated either with both compounds (Fig. 6e,f).

### In vivo phenotypic analysis of the *ΔldcF* mutant

**Figure 7:**
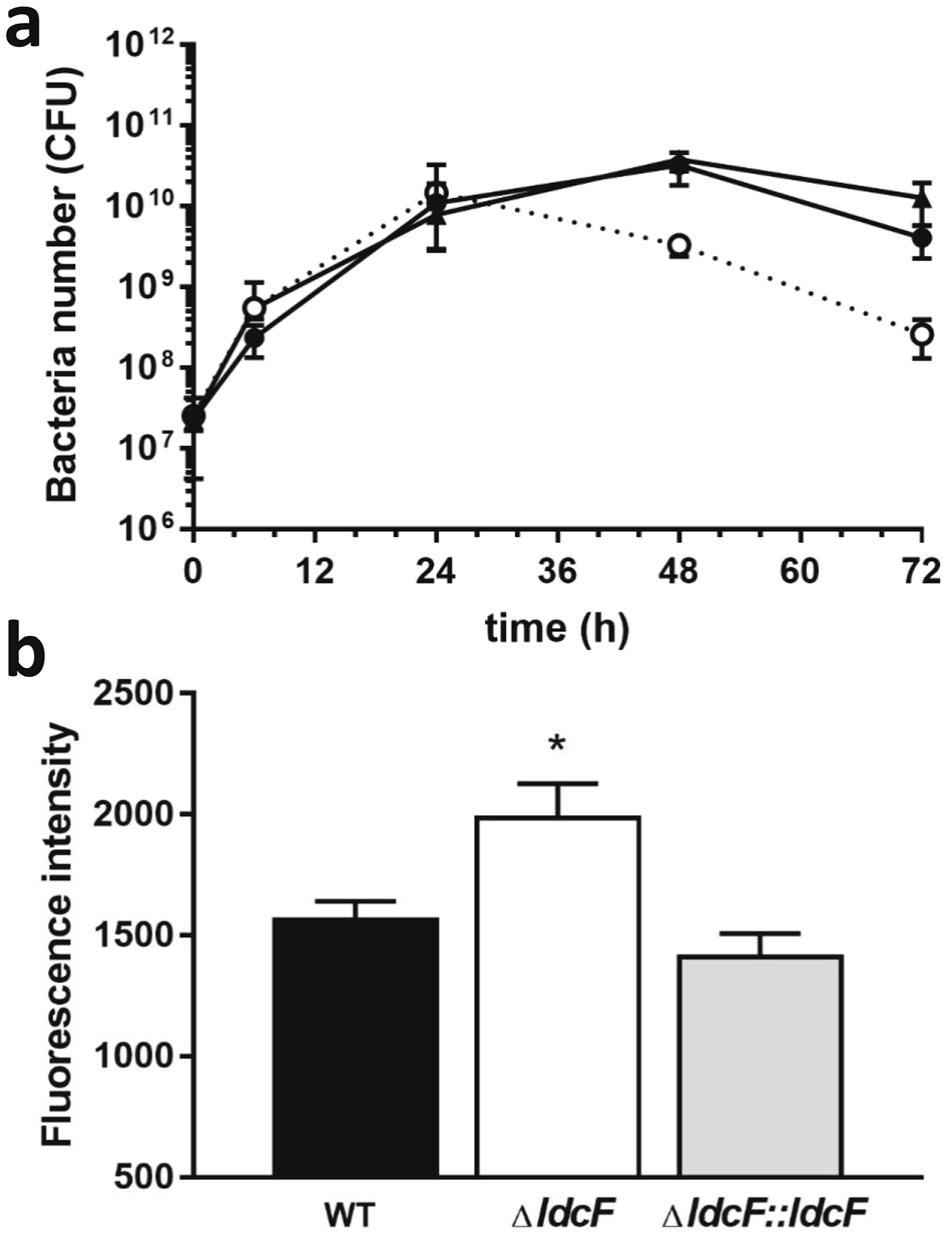
Replication of *F. novicida* strains within the J774 macrophage-like cell line. (**a**) *F. novicida* WT (black circles, solid line), Δ*ldcF* (white circles, dotted line) and Δ*ldcF::ldcF* (black triangles, solid line) were inoculated at a MOI of 100:1 and intracellular bacteria were enumerated by cfu counting at different times post infection (**b**) Production of ROS evaluated at 24 h after infection of macrophages with a MOI of 1,000:1 using the redox-sensitive dye DCFA detected by fluorescence spectroscopy. Data correspond to mean ± s.e.m. of 4 distinct experiments and after subtraction of background values obtained with uninfected macrophages. *P < 0.05.

We next evaluated the consequences of *ldcF* deletion on bacterial replication in macrophages. The uptake of *F. novicida* WT*,* Δ*ldc*F and Δ*ldcF::ldcF* strains into J774 cells, estimated upon macrophage infection with a MOI of 100, was found identical for the three strains which displayed equivalent intracellular growth profiles over the first 24 h (Fig. 7a). However, at 48 hpi and beyond, the number of viable intracellular ∆*ldcF* cells was found significantly lower than observed for the WT strain (∆*ldcF*: 2.63 × 10^8^ ± 0.47 × 10^8^, *n* = 8 *vs* WT: 4.08 × 10^9^ ± 0.64 × 10^9^, *n* = 8 ; *P* < 0.0005), and the effect was reversed with the Δ*ldcF::ldcF* (1.27 × 10^10^ ± 0.35 × 10^10^, *n* = 4). As assessed by measuring lactate dehydrogenase activity, this reduced level of recovered viable bacteria was not related with an increased host cell lysis that would result in the release of bacteria into the extracellular medium (WT: 30.6% ± 4.92% *vs* ∆*ldcF*: 25.95% ± 3.29% and Δ*ldcF::ldcF*: 30.79% ± 7.9%; *P* > 0.05; *n* = 3, measured at 72 hpi). Together, these data suggest that the deletion mutant displays a reduced capacity to escape macrophage killing mechanisms. Their failure to survive the antibacterial activities of host macrophages most probably relies on an intricate overlapping network of signals combining pro-inflammatory and immune responses as well as metabolic response of the infected cell. However, considering the role of macrophage oxidative burst in pathogen clearance, we then evaluated the ROS level in infected J774 cells. Our results demonstrate that macrophages infected with the ∆*ldcF* strain, which is less resistant to oxidative stress, display a higher ROS activity than cells infected either with the WT or the complemented strain (Fig. 7b), an effect that could result either from an impaired degradation or from an increased production of ROS.

### Comparative proteomics reveals proteins impacted by *ldcF* deletion

A MS-based quantitative proteomic comparison was performed on whole-cell extracts of *F. novicida* WT and Δ*ldcF* strains to identify proteins for which abundance was altered by *ldcF* deletion. A bioinformatics analysis reliably identified and quantified 1,263 different proteins from the 1,854 protein*-*coding ORFs annotated in the *F. novicida* genome (Supplementary Table S3; PXD016591). An ensuing statistical analysis revealed that expression levels of 80 proteins were significantly affected by *ldcF* deletion. Equal numbers of proteins were expressed at lower or higher abundance in Δ*ldc*F compared to the WT (Table 1). Consistent with the lack of LdcF in the deletion mutant, the 2,3,4,5-tetrahydropyridine-2,6-carboxylate N-succinyltransferase (FTN_1727, DapD) involved in lysine biosynthesis was found to be downregulated. Surprisingly, although all proteins encoded by the FPI (FTN_1309 to FTN_1326) ^5^ were detected in the MS-based quantitative proteomic assay (Supplementary Table S3), none of them showed altered expression levels in the Δ*ldcF* strain*.* In contrast, the amount of the major transcriptional regulator MglA (FTN_1290, MglA), which is described as a FPI gene regulator^8,48^, was significantly reduced in the Δ*ldcF* mutant (Table 1).

**Table 1:** List of proteins differentially expressed in Δ*ldcF*.

Gene name	Locus	Description	Proteomic data	
			Log 2FC	*P* value
**Downregulated proteins in ΔldcF**				
*cadA*	FTN_0504	**Lysine decarboxylase**	− 6.135487177	**6.5901E−12**
*udp*	FTN_0652	Uridine phosphorylase	− 2.442473795	0.000355044
*uvrB*	FTN_1176	Excinuclease ABC subunit B	− 2.359970258	1.5872E−07
*yhiP*	FTN_0885	Proton-dependent oligopeptide transporter (POT) family protein, di- or tripeptide:H + symporter	− 2.233739832	1.28816E−08
–	FTN_1453	Two-component regulator, sensor histidine kinase	− 2.225623368	0.000269144
–	FTN_0705	Abortive infection bacteriophage resistance protein	− 1.909345488	0.006797082
–	FTN_0655	N6-adenine-specific methylase	− 1.645819843	0.009836595
–	FTN_1348	Acetyltransferase	− 1.493386099	3.68028E−07
–	FTN_0898	Amino acid permease	− 1.424644622	3.5435E−06
*panD*	FTN_1354	Aspartate 1-decarboxylase	− 1.405501066	0.006567341
–	FTN_0862	Hypothetical protein	− 1.276557703	0.000969217
*ung*	FTN_1486	Uracil-DNA glycosylase	− 1.261354286	4.40904E−07
–	FTN_0308	Membrane protein of unknown function	− 1.255628185	0.000806353
–	FTN_1272	Proton-dependent oligopeptide transporter (POT) family protein, di- or tripeptide:H + symporter	− 1.243209089	1.20616E−05
*dapD*	FTN_1727	2,3,4,5-tetrahydropyridine-2,6-carboxylate *N*-succinyltransferase	− 1.185622324	0.000977552
–	FTN_1258	Hypothetical protein	− 1.11437438	0.000177817
*hsdM*	FTN_1152	Type I restriction-modification system, subunit M (methyltransferase)	− 1.098834276	0.000436818
–	FTN_1316	Hypothetical protein	− 1.075883638	0.005337719
–	FTN_1628	LysR family transcriptional regulator	− 1.010217775	0.001268379
*hdsR*	FTN_0710	Type I restriction-modification system, subunit R (restriction)	− 0.974133834	0.00046316
–	FTN_1212	Glycosyl transferases group 1 family protein	− 0.967248679	7.07496E−06
–	FTN_1397	Hypothetical protein	− 0.962399859	6.53792E−05
–	FTN_0976	ThiF family protein	− 0.962086728	6.76869E−05
*waaG*	FTN_1218	Glycosyl transferase, group 1	− 0.942030987	0.000643833
*pilE4*	FTN_0389	Type IV pili, pilus assembly protein	− 0.94007106	2.01122E−05
–	FTN_1440	Hypothetical protein	− 0.935671185	0.007635418
*ubiC*	FTN_0386	Chorismate pyruvate lyase	− 0.934045712	6.57717E−05
–	FTN_0137	Hypothetical protein	− 0.928404833	3.73274E−06
*mglA*	FTN_1290	Macrophage growth locus, protein A	− 0.926959218	6.24086E−07
–	FTN_1697	Galactose mutarotase	− 0.901042767	0.008192095
–	FTN_1148	Glycoprotease family protein	− 0.816650922	0.005012835
*galP1*	FTN_0687	Major facilitator superfamily galactose-proton symporter	− 0.746905316	5.94864E−05
–	FTN_1459	Short chain dehydrogenase	− 0.723695755	0.001285646
–	FTN_1254	Hypothetical protein	− 0.716291799	0.00237079
–	FTN_1266	ABC transporter membrane protein	− 0.66256701	0.001562237
–	FTN_0923	Hypothetical protein	− 0.654493997	0.002785307
*yhbG*	FTN_0902	ABC transporter, ATP-binding protein	− 0.652797305	3.32447E−05
*rpsF*	FTN_0951	30S ribosomal protein S6	− 0.641616628	5.78051E−05
*yrbI*	FTN_0905	3-Deoxy-d-manno-octulosonate 8-phosphate phosphatase	− 0.627622272	0.001032099
–	FTN_1547	Hypothetical protein	− 0.608477024	0.000859667
**Upregulated proteins in ΔldcF**				
*rimM*	FTN_1561	Ribosome maturation factor rimM	0.626513088	0.000555064
*apaH*	FTN_0561	Diadenosine tetraphosphatase	0.629366731	0.000669224
–	FTN_0118	S49 family serine peptidase	0.635972422	0.006578849
–	FTN_1468	Putative deoxyribonucleotide triphosphate pyrophosphatase	0.640919668	0.000191944
–	FTN_0089	Allophanate hydrolase subunit 2	0.672742647	0.000997506
*rnc*	FTN_1463	Ribonuclease 3	0.681039745	0.001289497
–	FTN_0789	Putative rhodanese, sulfurtransferase	0.68404703	0.000345296
*secF*	FTN_1094	Preprotein translocase subunit SecF	0.695399198	3.15477E−05
*murD*	FTN_0542	UDP-*N*-acetylmuramoylalanine–d-glutamate ligase	0.715198106	5.10756E−05
*xthA*	FTN_0838	Exodeoxyribonuclease III	0.726407142	1.90678E−05
*sun*	FTN_1347	tRNA and rRNA cytosine-C5-methylases, sun protein	0.73058117	0.00042405
–	FTN_0872	Small conductance mechanosensitive ion channel (MscS) family protein	0.78091602	2.90828E−05
–	FTN_1387	Hypothetical protein	0.812379067	0.00883337
*ispD*	FTN_0623	2-C-methyl-d-erythritol 4-phosphate cytidylyltransferase	0.824072741	5.66929E−06
–	FTN_0041	Hypothetical protein	0.828287322	0.002571557
*mltA*	FTN_1286	Membrane-bound lytic murein transglycosylase	0.843953091	0.000666109
–	FTN_1080	Phosphosugar binding protein	0.857709173	0.000844453
–	FTN_1015	Isochorismatase family protein	0.860701266	0.000470586
*pilW*	FTN_0307	Type IV pilus assembly protein	0.903906623	0.004575204
–	FTN_1061	Acid phosphatase, HAD superfamily protein	0.951666713	0.001807004
*pilV*	FTN_0413	Type IV pili, pilus assembly protein	0.975215693	0.001866241
*murQ*	FTN_1504	*N*-acetylmuramic acid 6-phosphate etherase	0.994710363	1.2722E−06
*tdh*	FTN_0625	l-Threonine 3-dehydrogenase	1.02107175	2.99619E−05
*ruvA*	FTN_1025	Holliday junction ATP-dependent DNA helicase RuvA	1.125358669	5.65196E−07
–	FTN_1506	Hypothetical protein	1.20406212	0.000656496
*putP*	FTN_0299	Proline/Na + symporter	1.206589507	0.001401361
–	FTN_0452	Hypothetical protein	1.274923951	4.08167E−08
–	FTN_0006	Hypothetical protein	1.345660313	1.02878E−06
–	FTN_0004	Aspartate/glutamate transporter	1.352713527	0.003023321
–	FTN_0829	Hypothetical protein	1.454738617	6.98601E−07
–	FTN_1388	Oxidoreductase	1.515433578	0.000565317
–	FTN_1267	ABC transporter ATP-binding protein	1.74578111	3.84443E−09
*lptC*	FTN_0904	Lipopolysaccharide export ABC transporter periplasmic protein	1.999604273	1.63282E−05
*rnpA*	FTN_0075	Ribonuclease P protein component	2.366923256	0.000708316
–	FTN_0384	Hypothetical protein	2.702678582	0.002040018
–	FTN_0987	tRNA-dihydrouridine synthase	2.830069785	5.08771E−06
–	FTN_1386	Hypothetical protein	2.845246288	4.58158E−09
–	FTN_0722	l-lysine 2,3-aminomutase	3.467288507	1.01829E−10
–	FTN_1220	Lipopolysaccharide synthesis sugar transferase	3.613912551	0.000821341
*recB*	FTN_1357	ATP-dependent exoDNAse (exonuclease V) beta subunit	3.825585172	1.16584E−11

The protein deleted in the mutant was labeled in bold.

The KEGG (Kyoto Encyclopedia of Genes and Genomes) database^49^ includes 99 pathways for *F. novicida* (https://www.genome.jp/kegg-bin/show_organism?org=ftn). Interestingly, following KEGG annotation, only 23 of the 80 proteins affected by the *ldcF* deletion were assigned to functional categories. These 23 proteins can be roughly grouped into a limited number of distinct functional pathways, including bacterial metabolism, DNA proofreading and repair, and pathways related to oxidative stress. One group of the KEGG-annotated differentially expressed proteins (5 out of 23) is associated with DNA proofreading and repair. This group is composed of two proteins involved in base excision repair pathways (FTN_1486, Ung and FTN_0838, XthA), one involved in nucleotide excision repair (FTN_1176, UvrB), and two involved in homologous recombination pathways (FTN_1025, RuvA and FTN_1357, RecB). Two of these proteins (UvrB and Ung), which displayed reduced expression levels in Δ*ldcF*, are enzymes considered to be prokaryotic defense systems involved in virulence through their protection of bacterial DNA^50,51^. Other proteins that were downregulated in Δ*ldcF* may also help bacteria to deal with DNA damage, although they lack functional KEGG assignment. These proteins included enzymes from type I restriction-modification systems (FTN_1152, HsdM; FTN_0710, HsdR)^52^ and the N6-adenine-specific methylase (FTN_0655). In contrast, some other DNA repair proteins were expressed at higher levels following *ldcF* deletion. An example is exodeoxyribonuclease III (XthA), a negative regulator of homologous recombination under log phase growth conditions, of which the overexpression can also result in unrepaired DNA damage.

The KEGG pathway annotation of proteins for which expression levels were significantly altered in Δ*ldcF* also revealed several metabolic and transport pathways that could play a role in bacterial replication. Specifically, the deletion mutant’s reduced capacity to deal with the host immune system and survive within macrophages may be partly related to the observed decrease in uridine phosphorylase levels (FTN_0652, Udp), as previously suggested using *Drosophila melanogaster* as an experimental model^53^. Similarly, UbiC (FNT_0386) catalyzes the first step of ubiquinone (or coenzyme Q) biosynthesis involved in electron transport chains and is considered as a lipid-soluble antioxidant in prokaryotes; its expression was reduced, and could thus impact *F. novicida*’s oxidative defense^54^. These findings are in good agreement with a possible role of LdcF in the activation of the SOS-response, and are underscored by the increased expression levels measured for RuvA (FTN_1025, Holliday junction ATP-dependent DNA helicase) and RecB (FTN_1357, ATP-dependent exoDNAse) that could help *F. novicida* to cope with oxidative stress^53^.

## Discussion

The phylogenetic analysis and amino acid sequence comparisons presented here indicate that the unique lysine decarboxylase identified within *Francisella* proteomes, i.e. LdcF (previously annotated as CadA) is more closely related to *E. coli* LdcI and LdcC than to *P. aeruginosa* LdcA or *E. coli* AdcI, OdcI and OdcC. However, similarly to most of the *P. aeruginosa* strains^26^ and unlike *E. coli*, *Francisella* genomes lack the presence of a RavA orthologue shown to alleviate inhibition of *E. coli* LdcI by the alarmone ppGpp^32^. Consistently, as in LdcA^35^, the C-terminal β-strands of LdcF display different amino acids at locations corresponding to the RavA binding site in *E. coli* LdcI. Furthermore, our structural analysis of *F. novicida* LdcF demonstrates that eight out of the 10 residues involved in *LdcI* interaction with ppGpp in *E. coli* show either a reverse in charge or change in hydrophobicity, which reveals that, again similarly to LdcA^35^, it is highly unlikely that LdcF would be inhibited by ppGpp. These observations underlying major differences between LdcF and LdcI are consistent with the absence of a RavA orthologue in *Francisella* genomes.

Examination of the *ldcF* genetic environment, which is highly conserved within different *Francisella* species, suggests that despite the high sequence identity and strong structural similarity with LdcI, LdcF expression is differently regulated. Notably, genes encoding both CadB, the putative cadaverine transport protein, and CadC, the pH sensor and membrane-bound transcriptional regulator of the *cadBA* operon^55^ are missing in the *ldcF* gene cluster. Upstream of *ldcF* are *lolC* and *lolD* which encode two components of the ABC transporter complex involved in lipoprotein transport and membrane biogenesis and are described as essential genes in *F. tularensis*^56^. The downstream genes belong to the glycine cleavage system (GCS) and were found significantly upregulated in the virulent *F. tularensis* type A Schu S4 strain inside macrophages^57^. Our observations are therefore in agreement with the data on the in vivo negative selection of *F. novicida* transposon mutants that pointed out the importance of GCS genes together with *lolD* and *ldcF* (FTT_0405 to FTT_0409) in the intracellular growth and/or virulence of *F. novicida*^23^.

The combined analysis of the genomic context of *Francisella ldcF*, the structure of *F. novicida* LdcF and the phylogenetic relationships between the new LdcF family and other proteobacterial LAOdcs raised questions about the LdcF regulation and functional activity. Our experiments showed that the growth rates of *F. novicida* WT and Δ*ldcF* are very similar over a broad range of basic to acidic pHs, thus ruling out a strict role of LdcF in acid tolerance and buffering of the bacterial cytosol upon acid stress. Deletion of *ldcF* also failed to affect temperature-dependent bacterial growth. In contrast, in comparison with the WT, Δ*ldcF* displayed a significantly lower resistance to oxidative stress. The capacity of cadaverine to scavenge oxygen radicals, thus providing bacteria with a higher tolerance towards oxidative stress, was previously reported for *E. coli*^58^ and *Vibrio vulnificus*^59,60^. Importantly, these results are corroborated by a greater survival of both the WT and Δ*ldcF::ldcF* strains in infected macrophages which contain a lower amount of ROS than those infected with Δ*ldcF*. Such a survival strategy could be shared with the bacterium that possesses the closest LdcF relative, i.e. *L. fallonii*, which replicates within the protozoan host *Acanthamoeba* in aquatic environments and must face oxidative and acidic stress conditions during its stationary phase of growth^18,61^.

To better understand the mechanism by which removal of *ldcF* and a subsequent defect in cadaverine synthesis altered the oxidative stress resistance, we performed an extensive quantitative comparison of the protein contents between the *F. novicida* WT and the Δ*ldcF* strains. This analysis identified 80 proteins for which expression levels were altered following *ldcF* deletion. Among them, we were surprised not to observe any ROS scavenging enzymes. Indeed, similarly to several other bacterial species, to cope with oxidative stress, *Francisella* utilize enzymes such as SodB, SodC, KatG and the recently identified AhpC^16,17,20,62,63^ to convert harmful ROS into innocuous products^19^. Moreover, expression levels of other factors contributing to ROS defense mechanisms, such as the efflux pump EmrA1 (FTL_0687), involved in SodB and KatG secretion^64^, or the *F. tularensis* (FTL_1014) oxidative stress regulator OxyR^16,18^, also displayed similar expression levels in WT and Δ*ldcF* strains. However, one of the low-abundance proteins in Δ*ldcF* was an ABC transporter (FTN_0902; FTL_1065, YhbG). Interestingly, this transporter was recently reported to be down-regulated in a Δ*oxy*R mutant of *F. tularensis* LVS displaying an enhanced sensitivity to oxidative stress^18^. Furthermore, the reduced UbiC content compared to the WT strain could also contribute to the diminished capacity of the *F. novicida* Δ*ldc*F strain to survive oxidative attack from ROS. Indeed, altered UbiC levels could indirectly promote ROS accumulation^54^. In addition to ROS-neutralizing enzymes, bacteria can also counteract ROS damage using their DNA damage-responsive genes. The products of these genes initiate DNA repair pathways to recognize and correct ROS-induced and other mismatches. An interesting hallmark of the *F. novicida* Δ*ldcF* proteome is the significant changes in levels of proteins involved in DNA repair processes potentially reducing the bacteria’s capacity to deal with oxidative stress. Our results also indicate that MglA expression was significantly reduced in the mutant strain. Interestingly, in addition to ensuring the regulation of *Francisella* virulence factors – which were unchanged in Δ*ldcF* compared to the WT strain – MglA has been reported to play a key role in the intracellular growth of *F. tularensis* and its adaptation to oxidative stress^8,11^.

While the relationships between the proteomic observations and *Francisell*a ROS defense mechanisms are not straightforward and a thorough understanding of the link between *ldcF* inactivation and the changes in the protein expression pattern requires further investigations, our observations provide an important evidence of the LdcF involvement in *F. novicida* oxidative stress resistance. By suppressing lysine decarboxylation, the *ldcF* deletion promotes the accumulation of lysine and the decrease of cadaverine, which both should have a direct impact on the bacterial physiology. Lysine harvesting was indeed described as a powerful preventive metabolic antioxidant strategy displayed by microbial cells^65^, an effect most probably reverted when this amino acid accumulates. The contribution of polyamines in several bacterial infections has long been described^66^, and some studies have specifically emphasized their relevance in *F. tularensis* virulence. For example, an increased expression of ornithine decarboxylase was observed in *F. tularensis* infected mice^67^. The relevance of spermine within host cells infected by *Francisella*, and specifically the capacity of this polyamine to elicit transcriptional changes in *F. tularensis*, leading in turn to altered host cell activation, has also been reported^68^. While never investigated, it could be hypothesized that cadaverine could also exert transcriptional control on genes implicated in *Francisella* resistance against oxidative attack. Taken together, our work provides a biochemical and structural framework to further explore LdcF as a potential virulence factor and its involvement in *Francisella* oxidative stress resistance.

While LAOdcs are long-recognised as drug targets, and development of specific mechanism-based Ldc inhibitors is an active research field^69–72^, we envision that the LdcF structure and the functional findings presented in this work will empower further investigations aimed at design of new LdcF-based therapeutic approaches against tularemia.

## Methods

### Bioinformatic analyses

Sequences of AAT-fold decarboxylases were retrieved from NCBI: LdcI (NP_418555.1), LdcC (NP_414728.1), AdcI (NP_418541.1), OdcC (NP_417440.1), and OdcI (NP_415220.1) from *Escherichia coli* str. K-12 substr. MG1655 and LdcA (NP_250509.1) from *P. aeruginosa* PAO1. These sequences were used as seeds to query a local database containing 4,467 complete proteomes of prokaryotes (Supplementary Table S4) from the National Center for Biotechnology Information (ftp://ftp.ncbi.nlm.nih.gov) with the BLASTP 2.2.6 software^73^ and with HMM-profile based approaches with the HMMER package v3.1b1 (default parameters)^74^. Finally, searches for unannotated sequences were performed with TBLASTN (default parameters) on the complete genome sequences corresponding to the 4,467 proteomes using default parameters. Sequences with an *e-value* lower than 10^–4^ were retrieved and aligned using MAFFT v.7^75^. The resulting multiple alignment was visually inspected with AliView 1.25^76^. Doubtful sequences were systematically verified using reciprocal best reciprocal blast hit. This led to the identification of 4,091 AAT-fold decarboxylase sequences, 13 of which were unannotated or annotated as pseudogenes (Supplementary Table S5).

A phylogeny of WING-containing AAT-fold decarboxylase sequences was inferred using maximum likelihood. To limit taxonomic redundancy, the phylogenetic analysis was performed on a subset of 1,905 representative proteomes by selecting randomly one representative strain per species. The 553 WING-containing AAT-fold decarboxylase sequences contained in these representative proteomes were aligned with MAFFT using the L-INS-i option and trimmed with BMGE v1.1 with matrix substitution BLOSUM30 (589 amino acid positions kept after trimming)^77^. The maximum likelihood tree was inferred with IQ-TREE 1.6.12^78^. IQ-TREE identified the LG + R10 as the best suited evolutionary model according to the Bayesian information^78^. The robustness of the inferred tree was assessed using the ultrafast bootstrap (1,000 replicates implemented in IQ-TREE). The genomic context figure has been generated by GeneSpy 1.1^79^ and phylogeny figures by iTOL^80^.

The percentage of identity between LdcF sequences and LdcI (NP_418555.1), LdcC (NP_414728.1), AdcI (NP_418541.1), OdcC (NP_417440.1), and OdcI (NP_415220.1) from *Escherichia coli* str. K-12 substr. MG1655 and LdcA (NP_250509.1) from *P. aeruginosa* PAO1 has been computed using the Needleman and Wunsch algorithm implemented at the NCBI (default parameters).

### Bacterial strains and growth conditions

The strain *F. novicida* CIP56.12 (Centre de Ressources Biologiques de l'Institut Pasteur, Paris, France) and the *ldc* mutants were grown on PVX-CHA plates (bioMérieux, Marcy l'Étoile, France) incubated at 37 °C in a 5% CO_2_-enriched atmosphere. Liquid cultures were carried out at 25 °C or 37 °C under agitation at 180 rpm in MMH medium, as indicated. For the growth of the complemented strain Δ*ldcF::ldcF*, liquid and solid media were supplemented with kanamycin (10 µg/mL).

### Cloning, expression and purification of LdcF

The sequences of all primers used in this study are given in Supplementary Table S6. The gene encoding LdcF (FTN_0504) was amplified by PCR from genomic DNA using High-Fidelity PCR master mix (Phusion, Finnzymes) and gene-specific primers FTN_0504F/R encoding a Tobacco Etch Virus (TEV) site. The resulting product was cloned into the pDONR 201 vector and subsequently subcloned into pDEST-17 using the Gateway cloning system from Invitrogen following the manufacturer’s instructions. Integrity of the N-terminal 6xHis-tagged construct was confirmed by DNA sequencing (Eurofins, Ebersberg, Germany). Protein expression was started by picking 5–10 colonies of freshly transformed *E. coli* C41(DE3) strain in 2 mL LB medium supplemented with ampicillin (100 µg/mL). After 3 h at 37 °C under shaking the bacterial suspension was transferred in 50 mL medium for additional 3 h, then diluted in 400 mL up to an optical density OD_600 nm_ of approximately 0.5 and expression was induced overnight at 16 °C by addition of 0.5 mM isopropyl-β-d-thiogalactopyranoside (IPTG). After centrifugation (5,000×*g*, 20 min) the bacteria were resuspended in lysis buffer (50 mM Tris pH 7.9, 300 mM NaCl, 0.1 mM pyridoxal 5′-phosphate (PLP) (Sigma), 0.5% CHAPS (Sigma), 5% glycerol, 2 mM ß-mercaptoethanol, 1 mM PMSF, Complete Protease Inhibitor (Roche Diagnostics) and 10 mM imidazole), then disrupted by sonication. The bacterial lysate was then heated for 5 min at 70 °C, centrifuged (20,000 rpm, 30 min, 4 °C) and applied onto a Ni^2+^-NTA column (Qiagen) for affinity purification. After extensive washing (50 mM Tris pH 7.9, 300 mM NaCl, 0.1 mM PLP, 5% glycerol, 2 mM ß-mercaptoethanol, and 20 mM imidazole) the protein was eluted with the same buffer supplemented with 300 mM imidazole. The fractions containing LdcF were pooled and dialyzed overnight at 4 °C against 50 mM HEPES pH 7, 25 mM NaCl, 0.1 mM PLP, 1 mM DTT and further purified on a Superose 6 size exclusion chromatography column (GE Healthcare, UK) and using the NGC Chromatography System (Bio-Rad). The peak fractions were analyzed on 10% SDS-PAGE and Coomassie blue staining and concentrated for crystallization without His-tag removal (Supplementary Fig. S10). In addition to the N-terminally His-tagged LdcF, a C-terminally His-tagged construct was also evaluated. The lysine decarboxylase activity of purified LdcF was assessed at pH 6.5 and 37 °C using a 2,4,6-trinitrobenzensulfonic acid assay as described^31,39^, with 8 mM lysine and 500 nM LdcF in the initial solutions before the mixture. Purified recombinant protein (1 mg/mL) was also used for rabbit immunization and production of a polyclonal antibody (Biotem, France) which appeared to also recognize *E. coli* LdcI but not AdcI, illustrating the close relationships between LdcF and LdcI.

### Crystallization, data collection and structure determination of LdcF

Prior to setting up crystallization trials, purified LdcF (50 mM HEPES pH 7, 25 mM NaCl, 0.1 mM PLP, 1 mM DTT) was concentrated to 3 mg/mL. Extensive crystallization trials that included 576 conditions tested in 96-well sitting drop vapor diffusion plates with drop volumes of 200 nL (100 nL protein solution + 100 nL reservoir solution, T: 20 °C) were performed at the high-throughput crystallization facility at the EMBL Grenoble outstation, France. LdcF initially crystallized in a condition containing 25% Ethylene Glycol (Crystal Screen HT, condition E4, Hampton Research). After manual optimization in 24-well hanging drop vapor diffusion plates (Molecular Dimensions, 1 µL protein solution + 1 µL reservoir solution, T: 20 °C), large brick shaped crystals appeared after several weeks of incubation, which displayed a bright yellow colour due to present PLP. LdcF crystals were scooped directly from the crystallization plates and were subsequently flash-cooled in liquid nitrogen.

Diffraction data was collected at beamline ID-29 at the European Synchrotron Radiation Facility (ESRF), Grenoble, France, and was processed using the XDS package^81^ in space group C2 2 2_1_ (a = 165.25 Å, b = 318.21 Å, c = 183.98 Å, α = β = γ = 90). The structure of LdcF was solved by maximum-likelihood molecular replacement using Phaser^82^ in the Phenix software package^83^, starting from the crystal structure of *E. coli* LdcI (PDB ID: 3n75) as an initial model^32^ , with side-chains trimmed using the Schwarzenbacher method^84^ in Sculptor from the Phenix package. The molecular replacement solution was subsequently refined in Phenix, using reciprocal- and real-space refinement, with noncrystallographic symmetry (NCS) restraints, occupancy refinement, individual B-factor refinement with TLS (translation liberation screw), and optimized x-ray/stereochemistry and x-ray/ADP weights. Several rounds of alternating refinement in Phenix and manual building in Coot^85^ were performed, followed by a final refinement in Phenix.

### Construction of the FTN_0504 knock-out strain

The *F. novicida ldcF* chromosomal deletion mutant was generated by allelic exchange as previously described^86^. Briefly, around 650 bp of both the 5′ and 3′ regions of FTN_0504 were amplified from genomic *F. novicida* DNA using Phusion DNA polymerase and fused with a kanamycin resistance cassette. The second round overlapping PCR was carried out with the primers FTN_0504ForUp and FTN_0504DownRev and using the mixture of the three previous PCR amplicons as template. Following purification from agarose gel (QIAquick Gel Extraction Kit, Qiagen), the resulting 2200 bp fragment of interest (1 µg) was used to transform chemically competent *F. novicida* U112 spread on PVX-CHA containing kanamycin (15 μg/mL). The antibiotic-resistance marker was further deleted through Flp-mediated excision and using the pKEK1112 temperature sensitive plasmid. The final mutant sentitive to both kanamycin and tetracycline was checked for loss of FTN_0504 by PCR product direct sequencing using appropriate primers (Eurofins).

### Construction of the LdcF complementation plasmid

The gene encoding the *F. novicida* LdcF WT was amplified using a primer pair on which NotI and AgeI restriction sites have been engineered and cloned into the pFNLTP6 shuttle plasmid downstream of the *gro* promoter^87^ using a DNA ligation kit (TAKARA BIO INC.). Following restriction enzyme digestion and sequencing, the resulting LdcF complementation plasmid designed pFNLTP6-*ldcF* was introduced into chemically competent *F. novicida.* Transformed colonies selected on PVX-CHA containing kanamycin (15 μg/mL) appeared after 2 days of incubation. Complementation was confirmed by PCR on purified DNA and by western-blot with the anti-LdcF antibody on whole bacterial extracts.

### Rezasurin assay

The metabolic activity of *F. novicida* wild-type and Δ*ldcF* was estimated through their capacity to reduce resazurin (7-hydroxy-3H-phenoxazin-3-one 10-oxide; λ_max_ = 600 nm ; Sigma-Aldrich) into the pink fluorescent compound resorufin (λ_max_ = 570 nm). This oxidation–reduction indicator of mitochondrial function was conveniently used to evaluate cell viability of several bacterial species^88^ including *F. tularensis, F. novicida and F. philomiragia*^89,90^. Exponential growth phase bacteria grown in MMH at 37 °C under shaking were transferred (200 µL) into a 96-well plate. After addition of 20 µL of resazurin (0.2 mg/mL) the microtiter plate was incubated for 1 h at 37 °C, and the cell viability was determined by OD_570nm_ − OD_600nm_ measurement (Tecan Plate reader). The experiment was repeated thrice with 6 replicates for each condition.

### Biofilm assay

Assessment of the biofilm formation was carried out using crystal violet assay and following optimized protocol for *F. novicida* starting from a 200 µL bacterial inoculum (1.10^7^ cells/mL) in flat-bottom 96-well plates. The biofilm biomass was quantified following 24 h incubation at 37 °C in a 5% CO_2_ incubator without shaking^90^.

### Evaluation of antimicrobial susceptibility

Antibiotic susceptibility was assessed by MIC, MBC/MIC and time-dependent killing assays, as previously described^89^. Briefly, the MICs were determined through the broth microdilution method following the CLSI recommended guidelines^47^ but using MMH as culture medium and with final antibiotic concentrations ranging from 0.125 to 64 μg/mL for gentamicin and from 0.002 to 1 μg/mL for ciprofloxacin. Plate counting of serial dilutions of the wells where no bacterial growth was visually observed allowed to estimate the MBC and to calculate the MBC/MIC ratio as a tolerance criterion^47^. The antibiotic tolerance was also evaluated using the method based on Minimum Duration for Killing 99% of the population—MDK metric^45^ starting from bacterial cultures grown in MMH to an OD_600nm_ of 0.5 prior addition of 25 times the MIC of gentamicin (25 µg/mL) or ciprofloxacin (1.6 µg/mL). Bacterial suspension were kept at 37 °C under shaking and the number of tolerant cells was assessed at different time points following antibiotic addition, through cfu counting. Each sample was processed in duplicate and results were expressed as a percent of viable bacteria relative to unexposed population.

### Spot plating assay

To accurately compare the oxidative stress responses of the different strains, two-fold serial dilutions of the drugs were prepared in MMH using 2 mL Eppendorf tubes, then dispensed in a 96 well plate (100 µL/well). Exponential phase bacteria diluted to a OD_600nm_ of 0.2 were then added (100 µL) in the wells containing drug dilutions. Posititive (without drug) and negative (without bacteria) controls were also included. Following 1 h incubation at 37 °C in a 5% CO_2_ incubator without shaking, 3 μL aliquots were spotted PVX-CHA plates further incubated at 37 °C for 48 h to 72 h before being photographed. All strains were processed in parallel in each experiment which included two independent replicates spotted twice.

### Disk diffusion method

A protocol essentially identical to that recently described for *F. tularensis* LVS and SCHU S4 strains was applied^20^. PVX-CHA plates were inoculated onto the entire surface with early stationary phase bacteria by using a sterile cotton swabs. Sterile cellulose disks (diameter 6 mm, D. Dutscher, France) were placed with sterile forceps and slightly pressed onto the gelose surface (up to 3 discs per plate), then impregnated with 10 µL of methyl viologen dichloride hydrate (Sigma) or menadione (Sigma). After 48 h incubation at 37 °C the diameter of the zone of complete inhibition (in mm) around each disk was measured thrice from numerized agar plate pictures and using ImageJ software.

### Western-blot analysis of LdcF expression

Whole bacterial extracts were resolved by 12% polyacrylamide SDS-PAGE before transfer on nitrocellulose membrane (Trans-Blot Turbo, Bio-Rad). Western-blot analysis was performed using a polyclonal antibody towards LdcF produced in this study (1:10,000) and a rabbit secondary antibody coupled to peroxidase (1:10,000; Jackson ImmunoResearch, Baltimore, PA, USA). The mouse anti-*Francisella*-IglC (1:10,000; Jackson ImmunoResearch, Baltimore, PA, USA) was used as positive control. Detection was carried out with enhanced chemiluminescence (Clarity Western ECL, Bio-Rad) using the Bio-Rad Chemidoc XRS + System.

### Mass spectrometry-based quantitative proteomic analyses

Bacterial cultures were grown in MMH at 37 °C under shaking to late logarithmic phase. Five independent replicates were prepared for each sample type. Aliquots corresponding to 1 × 10^9^ cells were centrifuged (7500×*g*; 10 min; 4 °C) and the resulting pellets were resuspended in 50 µL BugBuster Reagent to improve the release of soluble proteins before addition of 50 µL Laemmli buffer and storage at − 20 °C until further use. The protein concentrations estimated by the method of Bradford from the same amount of bacteria resuspended in distilled water and heated at 70 °C for 30 min was found homogenous ranging between 180 and 210 µg. Samples solubilized in Laemmli buffer were stacked in the top of a SDS-PAGE gel (4–12% NuPAGE, Life Technologies) and stained with Coomassie blue R-250 before in-gel digestion using modified trypsin (Promega, sequencing grade) as previously described^91^. Resulting peptides were analyzed by online nanoliquid chromatography coupled to tandem MS (UltiMate 3000 and Q Exactive Plus, Thermo Scientific). Peptides were sampled on a 300 µm × 5 mm PepMap C18 precolumn and separated on a 75 µm × 250 mm C18 column (ReproSil-Pur 120 C18-AQ 1.9 µm, Dr. Maisch GmbH) using a 120-min gradient. MS and MS/MS data were acquired using Xcalibur (Thermo Scientific).

Data were processed automatically using Mascot Distiller software (version 2.7.1.0, Matrix Science). Peptides and proteins were identified using Mascot (version 2.6) through concomitant searches against the *F. novicida* U112 database from MicroScope^92^, the classical contaminants database (homemade) and the corresponding reversed databases. The Proline software^93^ was used to filter the results: minimum peptide length of 7 amino acids, conservation of rank 1 peptides, peptide-spectrum match identification FDR < 1% as calculated on scores by employing the reverse database strategy, minimum peptide score of 25, and minimum of 1 specific peptide per identified protein group. Proline was then used to perform a compilation, grouping and MS1-based label-free quantification of the protein groups from the different samples.

Statistical analysis was performed using ProStaR^94^. Proteins identified in the reverse and contaminant databases, proteins identified with only 1 peptide, proteins identified by MS/MS in less than 3 replicates of one condition and proteins exhibiting less than 4 quantification values in one condition were discarded. After log2 transformation, abundance values were normalized using the variance stabilizing normalization procedure before missing value imputation (slsa method for POV and DetQuantile with quantile and factor set to 1 for MEC). Statistical testing was conducted using limma. Differentially expressed proteins were sorted out using a log2 (fold change) cut-off of ± 0.6 and *P*-values < 0.01 (FDR inferior to 2% according to the Benjamini–Hochberg estimator).

### Macrophage culture, infection, and cytotoxicity assay

Murine macrophage J774 cells were grown in DMEM GlutaMAX supplemented with 10% fetal calf serum and 1% penicillin/streptomycin at 37 °C, 5% CO_2_. One day before infection with bacteria, confluent flask of cells was trypsinized and seeded into 96-well plates at a concentration of 1.5 × 10^4^ cells/well and using antibiotic-free culture medium. The next day, host cells at 60–80% confluency were washed with 200 µL PBS and infected at a multiplicity of infection (MOI) of 100 with exponential growth phase bacteria. After centrifugation (350 × *g*; 5 min), the microtiter plate was incubated for 2 h at 37 °C, 5% CO_2_. The cell monolayer was then washed with PBS and remaining extracellular bacteria were killed by the addition of 10 μg/mL gentamicin (37 °C, 5% CO_2_) for 1 h. The antibiotic was removed by two washings with PBS and infected J774 incubated with the complete antibiotic-free culture medium. At a given time points after infection, cells were lysed by addition of 100 µL of Triton X-100 (0.5%) and the amount of viable bacteria was assessed though cfu counting from serial dilutions of lysed samples on PVX-CHA plates incubated at 37 °C for 24–48 h. Macrophage killing was measured using the CytTox 96 kit (Promega) following the manufacturer's instructions.

### Measurement of intracellular ROS in infected macrophages

J774 cells seeded in a 96-well plate were infected as described above with the different *F. novicida* strains and using MOI 1,000:1 with 6 technical replicates per condition. Unifected macrophages were used as negative controls. After 24 h incubation at 37 °C the monolayers were washed twice with 100 µL PBS and incubated for 45 min with 20 µM DCFA reagent (Abcam) following the manufacturer’s instructions. The ROS were detected by fluorescence spectroscopy (Tecan Plate reader) using excitation and emission wavelengths of 485 and 535 nm, respectively.

### Statistical analysis

All data correspond to at least 3 biological replicates. Otherwise indicated they were analyzed with Student’s t-tests and using the GraphPad PRISM software. The number of independent data points and *P* values are reported in figure legends.

## Supplementary Information

Supplementary Information.

## Data Availability

Crystallographic coordinates and structure factors for the crystal structure of *F. novicida* LdcF have been deposited in the wwPDB with accession code PDB: 6Y3X. The mass spectrometry proteomics data have been deposited to the ProteomeXchange Consortium via the PRIDE (PubMed ID: 30395289) partner repository with the dataset identifier PXD016669.
