# Supplementary material for: Structural and functional analysis of the Francisella lysine decarboxylase as a key actor in oxidative stress resistance
Source: Sci Rep. 2021 Jan 13;11:972. doi: 10.1038/s41598-020-79611-5 (PMC7806604; doi:10.1038/s41598-020-79611-5)
Supplement: Supplementary file 1 — Supplementary Information. [file 41598_2020_79611_MOESM1_ESM.pdf]

Supplementary Information for:

Structural and Functional Analysis of the *Francisella* Lysine Decarboxylase as a Key Actor in Oxidative Stress Resistance

Jan Felix[1¶], Claire Siebert[2¶] Julia Novion Ducassou[3], Jérôme Nigou[4], Pierre Simon Garcia[5,6], Angélique Fraudeau[1], Karine Huard[1], Caroline Mas[1], Céline Brochier-Armanet[5], Yohann Couté[3], Irina Gutsche[1*] and Patricia Renesto[2*]

[1]Institut de Biologie Structurale, Univ Grenoble Alpes, CNRS, CEA, IBS, 71 avenue des martyrs, F-38044 Grenoble, France
[2]TIMC-IMAG UMR 5525 - CNRS, INP, Université Grenoble Alpes, Grenoble Cedex 9, France
[3]Université Grenoble Alpes, CEA, Inserm, IRIG, BGE, Grenoble, France
[4]Institut de Pharmacologie et de Biologie Structurale, Université de Toulouse, CNRS, Université Paul Sabatier, Toulouse, France
[5]Univ Lyon, Université Lyon 1, CNRS, UMR5558, Laboratoire de Biométrie et Biologie Evolutive, 43 bd du 11 novembre 1918, F-69622 Villeurbanne, France
[6] Department of Microbiology, Stress Adaptation and Metabolism in Enterobacteria Unit, ERL CNRS 6002, Institut Pasteur, 25-28 Rue du Dr Roux, 75015, Paris, France
[*] Corresponding authors
Irina Gutsche (irina.gutsche@ibs.fr) and Patricia Renesto (patricia.renesto@univ-grenoble-alpes.fr)
[¶] J.F. and C.S. contributed equally to this work.

Supplementary Fig. 1 shows a multiple sequence alignment of the 122 sequences present in the phylogenetic tree shown in Figure 1b.
Supplementary Fig. 2 displays a high-resolution version of the cladogram and phylogram shown in Figure 1.
Supplementary Fig. 3 shows lysine decarboxylase activity measurements of LdcF using a 2,4,6-trinitrobenzensulfonic acid colorimetric assay.
Supplementary Fig. 4 shows the electron density map of the *F. novicida* LdcF crystal structure and is related to Figure 3.
Supplementary Fig. 5 corresponds to the construction of the *F. novicida* Δ*ldc*F deletion mutant and is related to Figs 5 to 7.
Supplementary Fig. 6 shows the full-length blots presented as cropped images in Supp. Figure 4.
Supplementary Fig. 7 shows the survival of *F. novicida* WT and Δ*ldc*F upon exposure to extreme pHs, and is complementary to Figure 5.
Supplementary Fig. 8 shows the growth of *F. novicida* WT and Δ*ldc*F at different temperatures and is complementary to Figure 5.
Supplementary Fig. 9 shows susceptibility of *F. novicida* WT and Δ*ldc*F to antibiotics.
Supplementary Fig. 10 shows purification of *F. novicida* LdcF and is related to Fig. 3.
Supplementary Table 1 shows crystallographic data collection and refinement statistics and is related to Figure 3.
Supplementary Table 2 shows viability of *F. novicida* WT and Δ*ldc*F grown for 24 h at different pHs and is complementary to Figure 5.
Supplementary Table 3 shows the differential analysis of total proteomes from *F. novicida* WT and Δ*ldcF* strains.
Supplementary Table 4 lists the 4,467 prokaryotic proteomes used for the bioinformatics analysis of LdcF**.**
Supplementary Table 5 lists the identified AAT-fold decarboxylase sequences.
Supplementary Table 6 lists the primers used in this study.

Supplementary Figure 1. Multiple sequence alignment of the 122 LdcF, LdcC, and LdcI sequences present in the phylogenetic trees shown in Fig. 1b. Functional domains are indicated by rectangles (wing domain (PF03709), PLP-binding domain and AAT-like domain (PF01276), C-terminal domain (PF03711)).

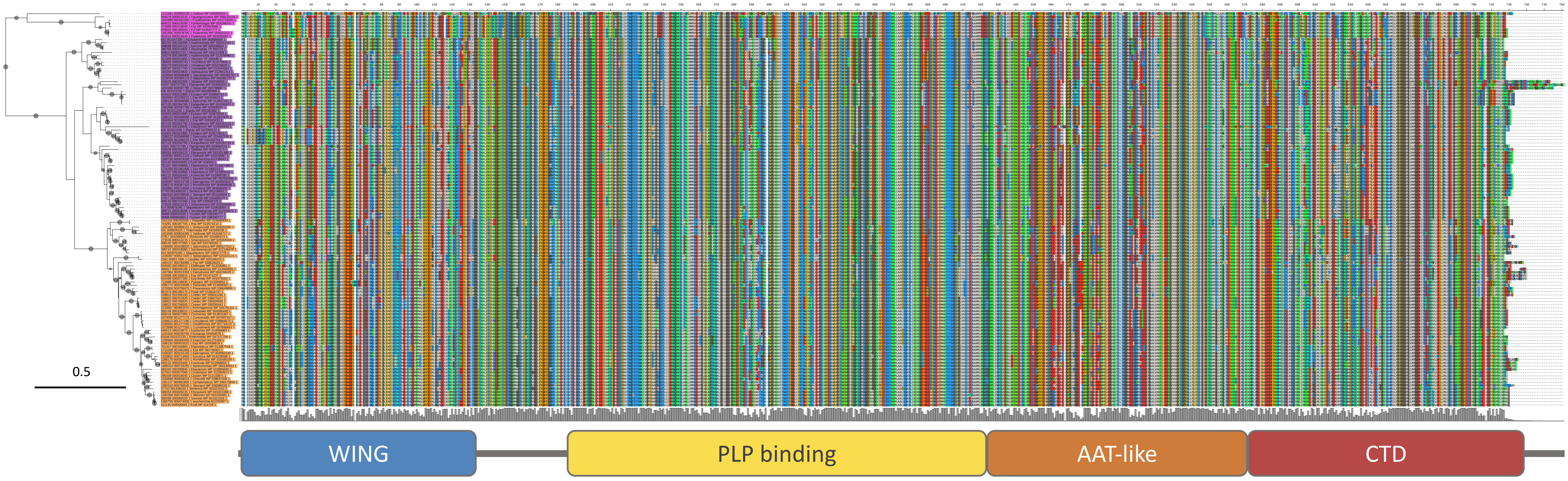
0.5
WING
PLP binding
AAT-like
CTD

Supplementary Figure 2. High resolution version of the phylogenetic trees displayed in Figure 1.

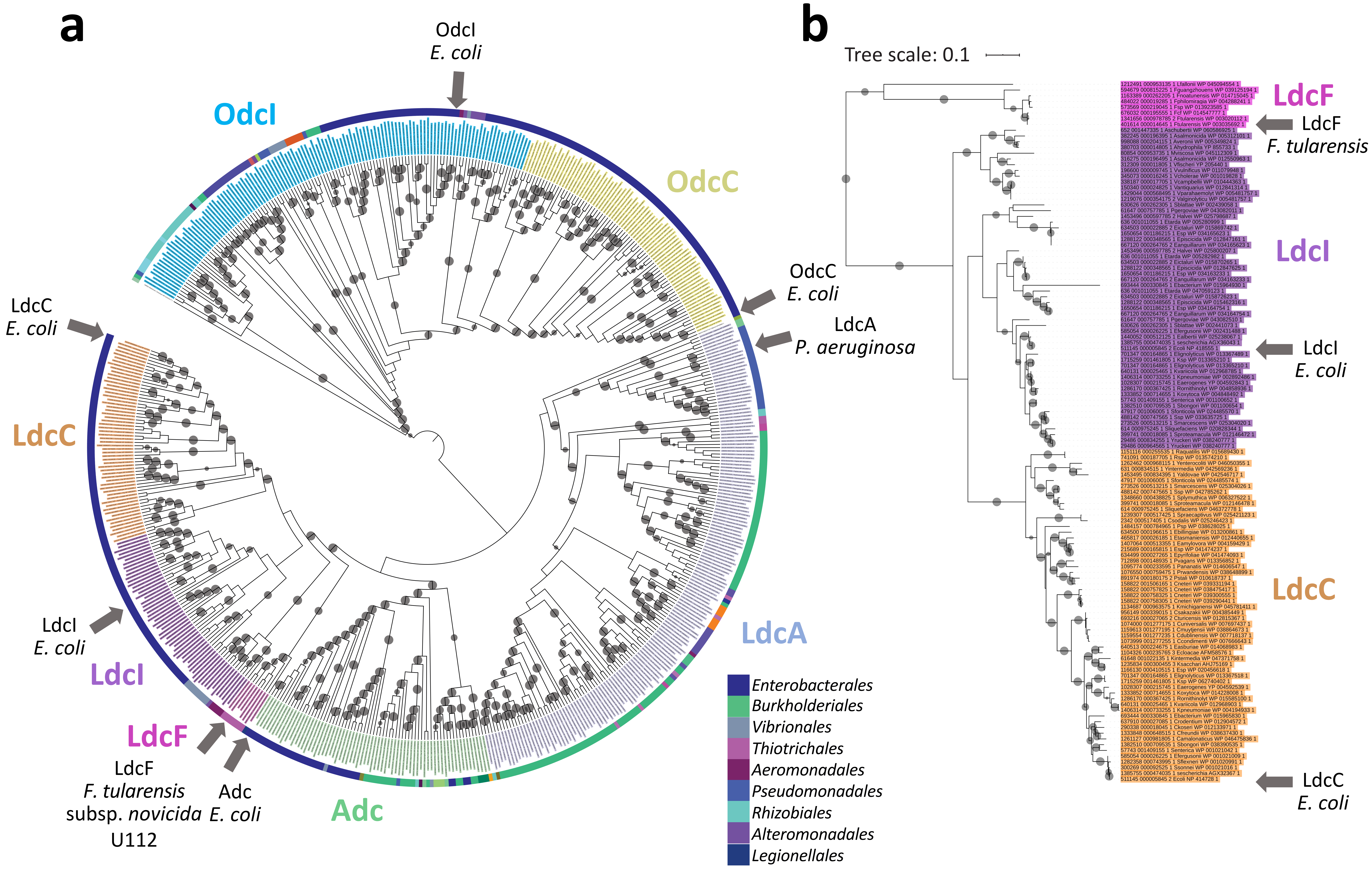

a
b
OdcI
E. coli
OdcI
OdcC
OdcC
E. coli
LdcA
P. aeruginosa
LdcC
E. coli
LdcC
LdcA
LdcI
E. coli
LdcI
LdcF
LdcF
F. tularensis
subsp. novicida
U112
Adc
E. coli
Adc
Enterobacterales
Burkholderiales
Vibrionales
Thiotrichales
Aeromonadales
Pseudomonadales
Rhizobiales
Alteromonadales
Legionellales
Tree scale: 0.1
LdcF
LdcF
F. tularensis
LdcI
LdcI
E. coli
LdcC
LdcC
E. coli

Supplementary Fig. 3. Lysine decarboxylase activity measurements of *F. novicida* LdcF using a 2,4,6-trinitrobenzensulfonic acid colorimetric assay adapted from: Kanjee, U., Houry, W.A. J. Vis. Exp. (46), e2094, doi:10.3791/2094 (2010). The full black line represents the logarithmic least-squares fit of the data. The dotted line represents the tangent used to calculate the initial activity rate (in nanomoles cadaverine produced per minute and per microgram of enzyme), which was measured to be ~5 nmoles cadaverine min^{-1} μg^{-1} LdcF.

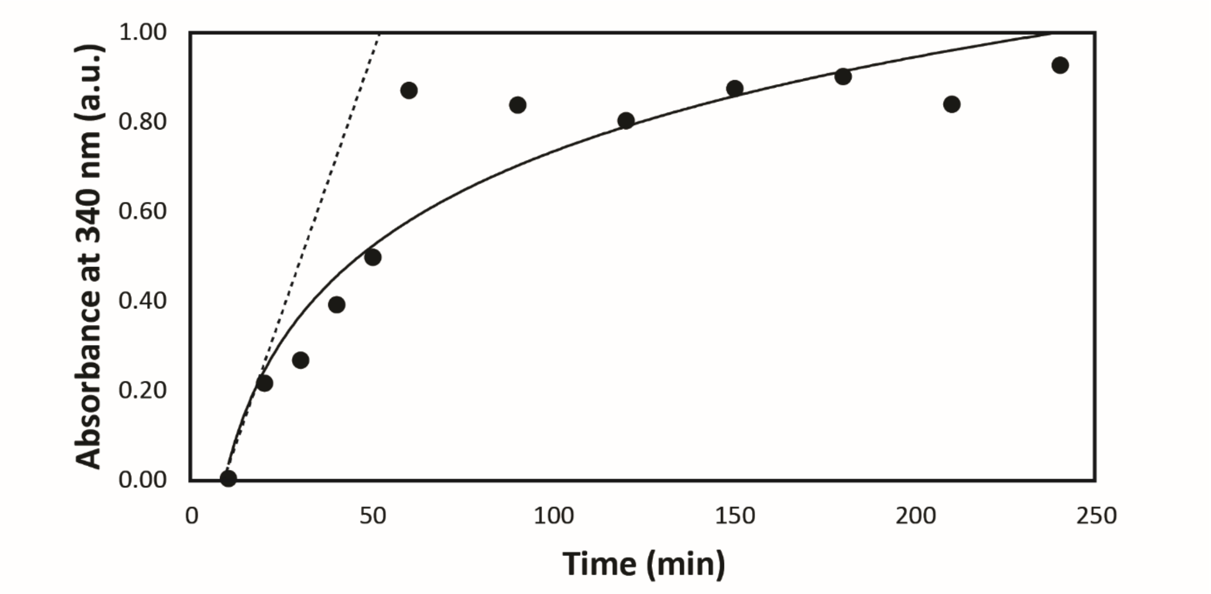

Supplementary Fig. 4 (**a**) Electron density map of an *F. novicida* LdcF decamer (left) and extracted dimer (right). (**b**) Representative electron density for the WING domain (left), with zooms showing selected WING-linker (middle) and WING-WING interactions (right).

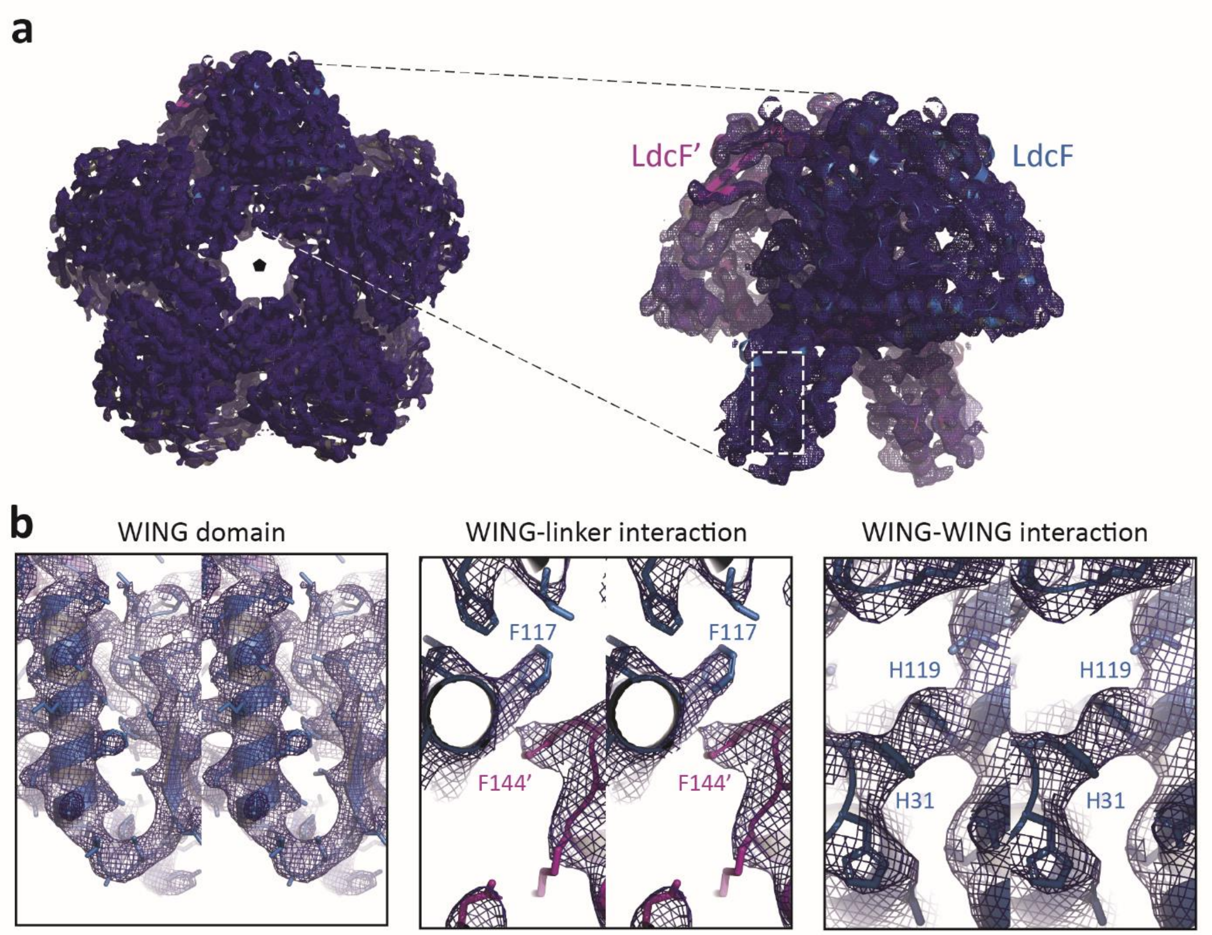

Supplementary Fig. 5. Phenotypic characteristics of *F. novicida* WT and Δ*ldcF*. (**a**) Homogeneity of both strains that form round and uniform colonies when grown on solid PVX-CHA plates for 24 h at 37°C with 5% CO_2. (**b**) The replication rate estimated by OD_{600nm} and (**c**) the metabolic activity evaluated by resazurin assay are similar for both strains during the first hours of replication. (**d**) LdcF expression from the WT and the Δ*ldcF::ldcF* strain was checked by western-blot with anti-LdcF antibodies and using anti-FupA as positive control. The original blots from which images were cropped are shown in Supplementary Figure 5.

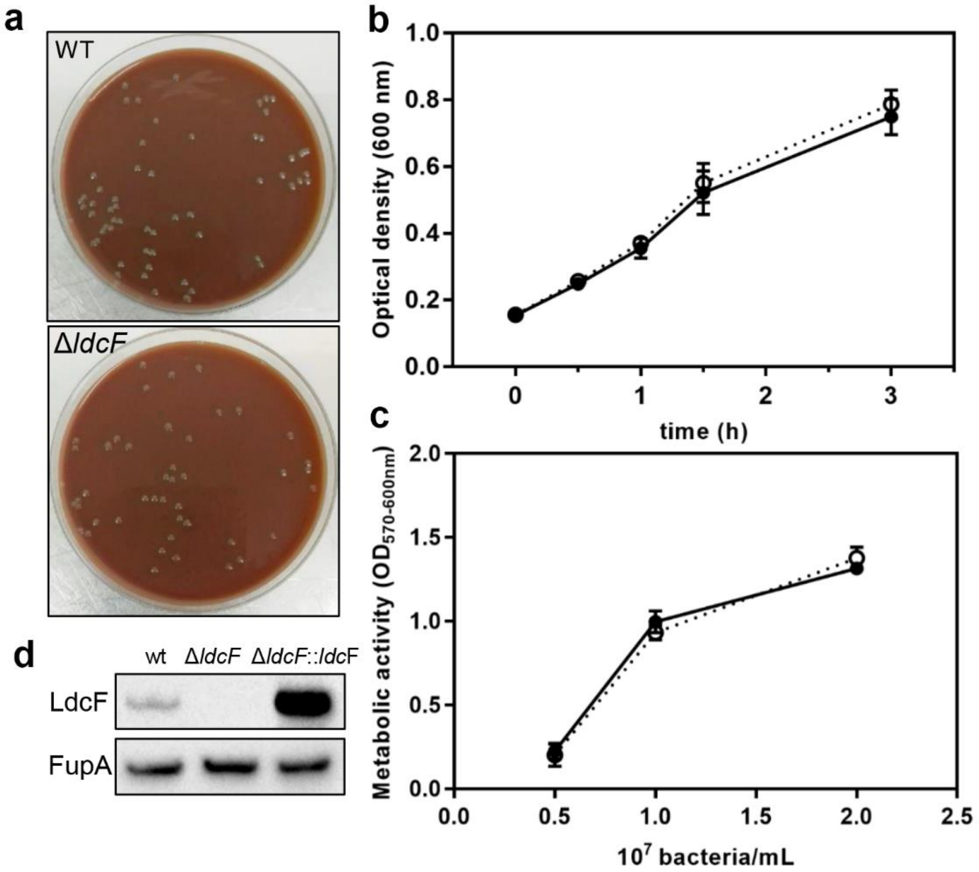

Supplementary Fig. 6. Western-blot analysis of LdcF expression in *F. novicida*. Left panel: A band with approximate size of 80 kDa corresponding to LdcF was detected in the lysate of *F. novicida* wild-type (1) but not in the Δ*ldcF* deletion mutant (2) and overexpressed in the Δ*ldcF::ldcF* complemented strain (3). Right panel : the amount of protein in each sample was checked using FupA (58 kDa) as positive control. This graph is representative of more than 6 experiments and corresponds to zoomed cropped sections shown in Supplementary Figure 4d.

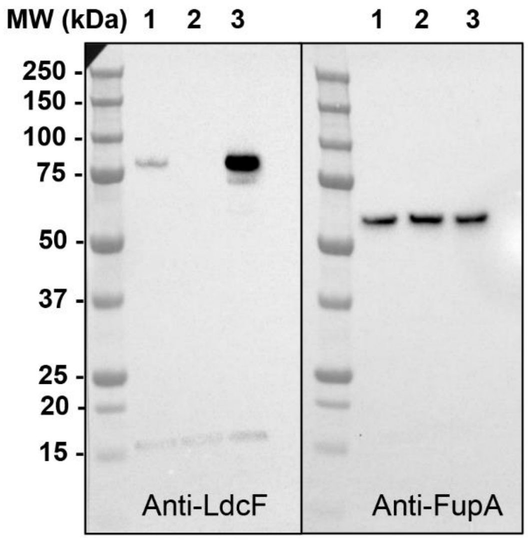

Supplementary Fig. 7. Tolerance response of *F. novicida* WT and Δ*ldc*F to extreme pHs. Late-phase exponential bacteria grown in MMH (pH 6.6) were centrifuged and resuspended in MMH adjusted to different pHs ($1x10^9$cells/mL). After 1 h incubation at room temperature, bacterial viability was assessed by cfu counting on PVX-CHA plates.

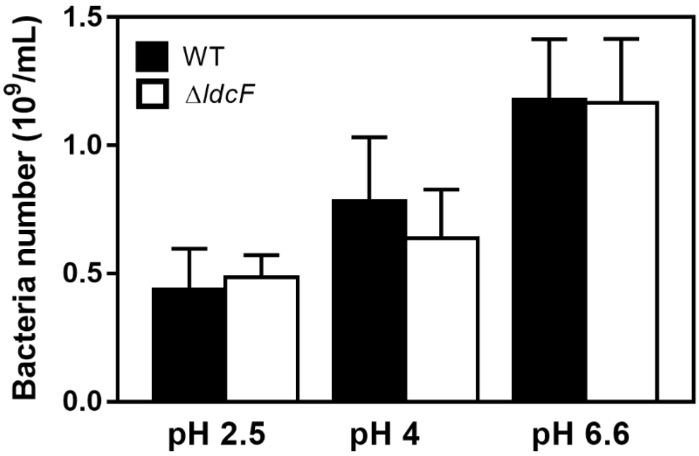

Supplementary Fig. 8. Growth of *F. novicida* WT and Δ*ldc*F at different temperatures. *F. novicida* WT (solid lines) and Δ*ldcF* (dotted lines) were grown in MMH under shaking at 25°C (blue) or at 37°C (red) and the bacterial growth was monitored by OD_{600nm} measurement. This graph is a representative of 3 distinct experiments.

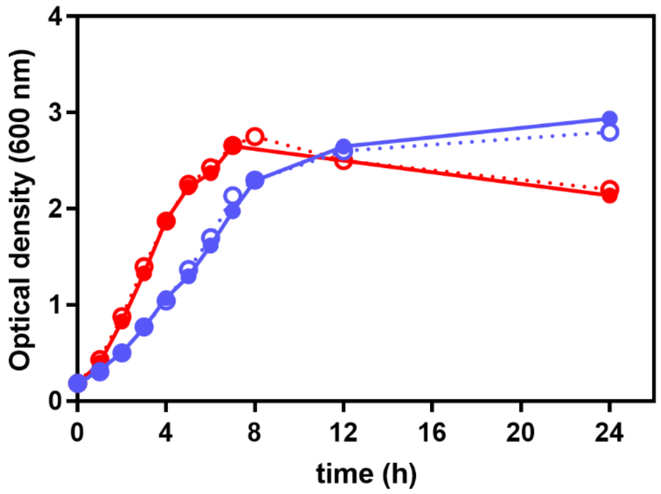

Supplementary Figure 9. Killing curves of exponential growth phase bacteria. *F. novicida* WT (black circles), Δ*ldcF* (white circles, dotted lines) and Δ*ldcF::ldcF* strains (black triangles) were exposed to 25-fold the MIC of (**a**) ciprofloxacin (1.6 mg/L) or (**b)** gentamicin (25 mg/L) and the proportions of surviving bacteria relative to the inoculum were quantified by assessing the number of cfu grown on PVX-CHA plates. This graph is a representative of 3 independent experiments each performed in duplicate.

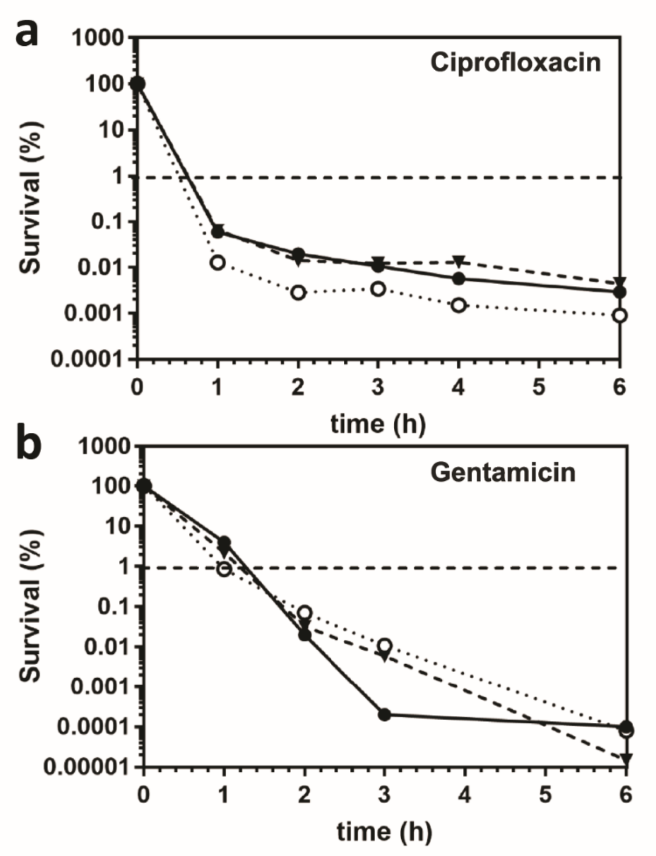

Supplementary Fig. 10. Purification of *F. novicida* LdcF. The protein eluted from the Ni^{2+}-NTA column (**a**) and was subsequently applied onto a Superose 6 column (**b**, **c**). The fractions corresponding to the elution peak (around 12 ml, collected fractions 23-25) were concentrated for crystallization trials.

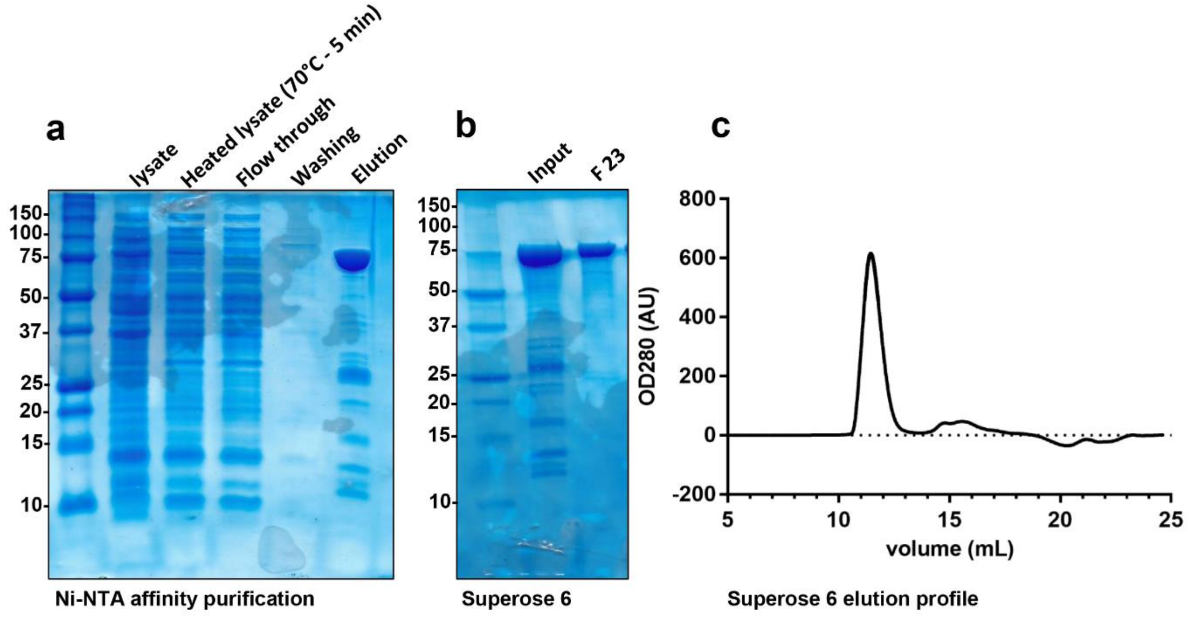

Supplementary Table 1. Crystallographic data collection and refinement statistics.

	Francisella novicida Lysine decarboxylase LdcF
**Data collection statistics**	
Beamline	ID29 – ESRF, Grenoble, France
Space group	*C222₁*
Cell dimensions	
a, b, c (Å)	165.27 318.25 184.02
α, β, γ (°)	90, 90, 90
Resolution (Å)	146.7 - 3.4 (3.60 - 3.40)
Unique reflections	66664 (10429)
**R_{meas} (%)**	0.087 (1.709)
I/σ(I)	12.16 (0.94)
CC(1/2)	99.8[#] (44.9)[#]
Completeness (%)	99.0 (96.6)
Multiplicity	3.8 (3.8)
Wilson B-factor	152.10
**Refinement**	
Resolution (Å)	89.27 - 3.4
Reflections used in refinement	66458 (6551)
Reflections used for R-free	6644 (653)
**R_{work}/R_{free}**	0.1873 (0.3660)/0.2365 (0.4023)
No. atoms	28440
Macromolecules	28440
Ligands	-
Solvent	-
**Average B factor (Å^2)**	175.92
**Macromolecules**	175.92
**Ligands**	-
**Solvent**	-
Number of TLS groups	5
r.m.s. deviations	
bonds (Å)	0.004
angles (°)	0.69
Ramachandran favored (%)	94.04
Ramachandran outliers (%)	0.00
Rotamer outliers (%)	0.39
Clashscore	6.57
PDB access code	6Y3X

Values in parentheses correspond to the highest-resolution shell.
CC(1/2) = percentage of correlation between intensities from random half-datasets[1,2].
Correlation significant at the 0.1% level is marked by an #

1. Diederichs, K., and Karplus, P.A. (2013). Better models by discarding data? Acta Crystallogr. Sect. D Biol. Crystallogr. *69*, 1215–1222.

2. Karplus, P.A., and Diederichs, K. (2012). Linking crystallographic model and data quality. Science (80-.). *336*, 1030–1033.

Supplementary Table 2. Viability of bacteria grown for 24 h at different pHs. Cfu counting was performed from serial dilutions of the bacterial suspensions grown for 24 h at 37°C under shaking in MMH adjusted at different pHs. Data corresponding to the mean ± SEM of two points obtained from distinct serial dilutions were expressed as Log_{10} cfu/mL. The experiment was performed in duplicate, with similar results obtained for each replicate.

**pH**	***F. novicida* WT**	***F. novicida* Δ*ldcF***
**2.5**	0	0
**4**	0.73 ± 0.036	0.68 ± 0.028
**6.6**	5.45 ± 0.65	5.79 ± 0.066
**8**	0.72 ± 0.38	0.86 ± 0.2

Supplementary Table 3: Differential analysis of total proteomes from *F. novicida* WT and Δ*ldc* F strains.

Gene ID	Gene name	Protein name	peptides	Differential proteomic analysis			KEGG pathways
				log_2(Fold Change WT/Δ*ldc* F)	p-value	Significant	
FTN_0504		lysine decarboxylase	17	6.14	6.6E-12	1	Lysine degradation;Metabolic pathways;Biosynthesis of secondary metabolites;Microbial metabolism in diverse environments
FTN_0652	udp	uridine phosphorylase	3	2.44	3.6E-04	1	Pyrimidine metabolism;Metabolic pathways
FTN_1176	uvrB	excinuclease ABC subunit B	3	2.36	1.6E-07	1	Nucleotide excision repair
FTN_0885		proton-dependent oligopeptide transporter (POT) family protein, di- or tripeptide:H+ symporter	3	2.23	1.3E-08	1	
FTN_1453		two-component regulator, sensor histidine kinase	4	2.23	2.7E-04	1	
FTN_0705		abortive infection bacteriophage resistance protein	3	1.91	6.8E-03	1	
FTN_0655		N6-adenine-specific methylase	2	1.65	9.8E-03	1	
FTN_1348		acetyltransferase	5	1.49	3.7E-07	1	
FTN_0898		amino acid permease	2	1.42	3.5E-06	1	
FTN_1354	panD	Aspartate 1-decarboxylase	2	1.41	6.6E-03	1	beta-Alanine metabolism;Pantothenate and CoA biosynthesis;Metabolic pathways;Biosynthesis of secondary metabolites
FTN_0862		hypothetical protein	6	1.28	9.7E-04	1	
FTN_1486	ung	Uracil-DNA glycosylase	2	1.26	4.4E-07	1	Base excision repair
FTN_0308		membrane protein of unknown function	4	1.26	8.1E-04	1	
FTN_1272		proton-dependent oligopeptide transporter (POT) family protein, di- or tripeptide:H+ symporter	2	1.24	1.2E-05	1	
FTN_1727	dapD	2,3,4,5-tetrahydropyridine-2,6-carboxylate N-succinyltransferase	4	1.19	9.8E-04	1	Lysine biosynthesis;Metabolic pathways;Microbial metabolism in diverse environments;Biosynthesis of amino acids
FTN_1258		hypothetical protein	6	1.11	1.8E-04	1	
FTN_1152		type I restriction-modification system, subunit M (methyltransferase)	9	1.10	4.4E-04	1	
FTN_1316		hypothetical protein	8	1.08	5.3E-03	1	
FTN_1628		LysR family transcriptional regulator	9	1.01	1.3E-03	1	
FTN_0710		type I restriction-modification system, subunit R (restriction)	11	0.97	4.6E-04	1	
FTN_1212		glycosyl transferases group 1 family protein	3	0.97	7.1E-06	1	
FTN_1397		hypothetical protein	25	0.96	6.5E-05	1	
FTN_0976		ThiF family protein	6	0.96	6.8E-05	1	
FTN_1218		glycosyl transferase, group 1	5	0.94	6.4E-04	1	
FTN_0389		Type IV pili, pilus assembly protein	6	0.94	2.0E-05	1	
FTN_1440		hypothetical protein	4	0.94	7.6E-03	1	
FTN_0386	ubiC	chorismate pyruvate lyase	5	0.93	6.6E-05	1	Ubiquinone and other terpenoid-quinone biosynthesis;Metabolic pathways;Biosynthesis of secondary metabolites
FTN_0137		hypothetical protein	4	0.93	3.7E-06	1	
FTN_1290	mglA	macrophage growth locus, protein A	5	0.93	6.2E-07	1	
FTN_1697		galactose mutarotase	5	0.90	8.2E-03	1	
FTN_1148		glycoprotease family protein	8	0.82	5.0E-03	1	
FTN_0687	galP1	major facilitator superfamily galactose-proton symporter	4	0.75	5.9E-05	1	
FTN_1459		short chain dehydrogenase	8	0.72	1.3E-03	1	
FTN_1254		hypothetical protein	8	0.72	2.4E-03	1	
FTN_1266		ABC transporter membrane protein	4	0.66	1.6E-03	1	ABC transporters
FTN_0923		hypothetical protein	3	0.65	2.8E-03	1	
FTN_0902	yhbG	ABC transporter, ATP-binding protein	12	0.65	3.3E-05	1	ABC transporters
FTN_0951	rpsF	30S ribosomal protein S6	11	0.64	5.8E-05	1	Ribosome
FTN_0905	yrbI	3-deoxy-D-manno-octulosonate 8-phosphate phosphatase	8	0.63	1.0E-03	1	Lipopolysaccharide biosynthesis;Metabolic pathways
FTN_1547		hypothetical protein	7	0.61	8.6E-04	1	
FTN_1561	rimM	Ribosome maturation factor rimM	4	-0.63	5.6E-04	1	
FTN_0561	apaH	diadenosine tetraphosphatase	12	-0.63	6.7E-04	1	Purine metabolism;Metabolic pathways
FTN_0118		S49 family serine peptidase	8	-0.64	6.6E-03	1	
FTN_1468		putative deoxyribonucleotide triphosphate pyrophosphatase	5	-0.64	1.9E-04	1	Purine metabolism;Metabolic pathways
FTN_0089		hydrolase subunit	6	-0.67	1.0E-03	1	
FTN_1463	rnc	Ribonuclease 3	7	-0.68	1.3E-03	1	
FTN_0789		UPF0176 protein FTN_0789	13	-0.68	3.5E-04	1	
FTN_1094	secF	preprotein translocase subunit SecF	5	-0.70	3.2E-05	1	Protein export;Bacterial secretion system
FTN_0542	murD	UDP-N-acetylmuramoylalanine--D-glutamate ligase	11	-0.72	5.1E-05	1	D-Glutamine and D-glutamate metabolism;Peptidoglycan biosynthesis;Metabolic pathways
FTN_0838	xthA	exodeoxyribonuclease III	7	-0.73	1.9E-05	1	Base excision repair
FTN_1347	sun	tRNA and rRNA cytosine-C5-methylases, sun protein	7	-0.73	4.2E-04	1	
FTN_0872		small conductance mechanosensitive ion channel (MscS) family protein	10	-0.78	2.9E-05	1	
FTN_1387		hypothetical protein	3	-0.81	8.8E-03	1	
FTN_0623	ispD	2-C-methyl-D-erythritol 4-phosphate cytidylyltransferase	6	-0.82	5.7E-06	1	Terpenoid backbone biosynthesis;Metabolic pathways;Biosynthesis of secondary metabolites;Biosynthesis of antibiotics

FTN_0041		hypothetical protein	4	-0.83	2.6E-03	1	
FTN_1286	mltA	membrane-bound lytic murein transglycosylase	8	-0.84	6.7E-04	1	
FTN_1080		phosphosugar binding protein	10	-0.86	8.4E-04	1	Alanine, aspartate and glutamate metabolism;Amino sugar and nucleotide sugar metabolism;Metabolic pathways;Biosynthesis of antibiotics
FTN_1015		isochorismatase family protein	5	-0.86	4.7E-04	1	
FTN_0307		hypothetical protein	2	-0.90	4.6E-03	1	
FTN_1061		acid phosphatase, HAD superfamily protein	3	-0.95	1.8E-03	1	
FTN_0413	pilV	Type IV pili, pilus assembly protein	8	-0.98	1.9E-03	1	
FTN_1504	murQ	N-acetylmuramic acid 6-phosphate etherase	13	-0.99	1.3E-06	1	Amino sugar and nucleotide sugar metabolism;Metabolic pathways
FTN_0625	tdh	L-threonine 3-dehydrogenase	7	-1.02	3.0E-05	1	Glycine, serine and threonine metabolism;Metabolic pathways
FTN_1025	ruvA	Holliday junction ATP-dependent DNA helicase RuvA	2	-1.13	5.7E-07	1	Homologous recombination
FTN_1506		hypothetical protein	6	-1.20	6.6E-04	1	
FTN_0299	putP	proline:Na+ symporter	3	-1.21	1.4E-03	1	
FTN_0452		hypothetical protein	2	-1.27	4.1E-08	1	
FTN_0006		hypothetical protein	4	-1.35	1.0E-06	1	
FTN_0004		aspartate/glutamate transporter	3	-1.35	3.0E-03	1	
FTN_0829		hypothetical protein	5	-1.45	7.0E-07	1	
FTN_1388		oxidoreductase	3	-1.52	5.7E-04	1	
FTN_1267		ABC transporter ATP-binding protein	7	-1.75	3.8E-09	1	ABC transporters
FTN_0904		hypothetical protein	3	-2.00	1.6E-05	1	
FTN_0075	rnpA	ribonuclease P protein component	2	-2.37	7.1E-04	1	
FTN_0384		hypothetical protein	2	-2.70	2.0E-03	1	
FTN_0987		tRNA-dihydrouridine synthase	3	-2.83	5.1E-06	1	
FTN_1386		hypothetical protein	5	-2.85	4.6E-09	1	
FTN_0722		hypothetical protein	2	-3.47	1.0E-10	1	
FTN_1220		lipopolysaccharide synthesis sugar transferase	2	-3.61	8.2E-04	1	
FTN_1357	recB	ATP-dependent exoDNAse (exonuclease V) beta subunit	2	-3.83	1.2E-11	1	Homologous recombination
FTN_1583	glpF	glycerol uptake facilitator protein	2	-0.84	9.9E-02	0	
FTN_0813	bioC	biotin synthesis protein BioC	3	-1.53	3.1E-02	0	Biotin metabolism;Metabolic pathways
FTN_0977		hypothetical protein	3	-0.44	5.0E-01	0	
FTN_1531		hypothetical protein	3	0.38	4.0E-01	0	
FTN_1771		hypothetical protein	3	-0.35	2.6E-01	0	
FTN_0877	cls	cardiolipin synthetase	5	-1.02	1.6E-01	0	Glycerophospholipid metabolism;Metabolic pathways
FTN_0544		hypothetical protein	2	-0.14	2.9E-01	0	
FTN_0662		thiamine pyrophosphokinase	3	0.27	6.4E-01	0	Thiamine metabolism;Metabolic pathways
FTN_0037		hypothetical protein	4	-0.02	9.8E-01	0	
FTN_0067		hypothetical protein	2	-0.11	6.9E-01	0	
FTN_0313		acetyltransferase	3	-0.47	3.0E-01	0	
FTN_1399		hypothetical protein	3	0.06	6.6E-01	0	
FTN_0345		SMF family DNA uptake protein	4	0.02	9.6E-01	0	
FTN_0031		LysR family transcriptional regulator	4	-0.18	7.3E-01	0	
FTN_0888		hypothetical protein	2	0.26	5.1E-02	0	
FTN_0857		hypothetical protein	3	-1.08	1.9E-02	0	
FTN_1611		major facilitator transporter	2	0.21	4.2E-01	0	
FTN_1161		hypothetical protein	2	-0.01	9.9E-01	0	
FTN_0968	dnaQ	DNA polymerase III, epsilon subunit	3	-0.28	5.2E-01	0	DNA replication;Mismatch repair;Homologous recombination
FTN_1103		hypothetical protein	3	-1.74	4.2E-02	0	
FTN_1260		hypothetical protein	7	-0.24	6.4E-01	0	
FTN_1587		hypothetical protein	2	-0.23	6.4E-01	0	
FTN_0429		hypothetical protein	2	0.08	7.9E-01	0	
FTN_1345		hypothetical protein	2	-0.01	9.8E-01	0	
FTN_0712	hslR	heat shock protein 15 (HSP15)	2	0.02	9.5E-01	0	
FTN_0281		hypothetical protein	2	-0.36	1.3E-02	0	
FTN_0017		phage integrase	2	0.01	9.6E-01	0	
FTN_0086		metabolite:H+ symporter (MHS) family protein	2	-1.67	3.6E-02	0	
FTN_0479		M50B family metallopeptidase	2	-0.06	8.6E-01	0	
FTN_0438	rlmE	Ribosomal RNA large subunit methyltransferase E	2	-0.21	1.1E-01	0	
FTN_1558	xerD	site-specific recombinase	2	-0.18	5.1E-01	0	
FTN_0199	cyoE	Protoheme IX farnesyltransferase	2	0.23	2.8E-01	0	Oxidative phosphorylation;Porphyrin and chlorophyll metabolism;Metabolic pathways;Biosynthesis of secondary metabolites
FTN_0407		amino acid ABC transporter permease	2	-0.25	3.5E-01	0	
FTN_0596		hypothetical protein	2	-1.00	6.0E-02	0	
FTN_0234	pgsA	phosphatidylglycerophosphate synthetase	3	0.18	4.8E-01	0	Glycerophospholipid metabolism;Metabolic pathways
FTN_0932		ABC transporter, ATP-binding protein	3	0.21	3.4E-01	0	
FTN_0783		isochorismatase family protein	3	-0.18	6.9E-01	0	
FTN_0758		hypothetical protein	3	0.83	4.5E-02	0	

FTN_0206		hypothetical protein	3	-0.01	9.4E-01	0	
FTN_0867		Putative Holliday junction resolvase	4	-0.79	7.3E-02	0	
FTN_1607	cca	CCA-adding enzyme	4	0.12	4.5E-01	0	
FTN_0267		hypothetical protein	4	-1.25	8.8E-02	0	
FTN_0954		histidine acid phosphatase	4	-0.38	3.9E-01	0	
FTN_1195	murG	UDP-N-acetylglucosamine--N-acetylmuramyl-(pentapeptide) pyrophosphoryl-undecaprenol N-acetylglucosamine transferase	4	-0.88	1.8E-02	0	Peptidoglycan biosynthesis;Metabolic pathways;Vancomycin resistance
FTN_1300		LysR family transcriptional regulator	4	1.03	8.5E-02	0	
FTN_0930		hypothetical protein	5	-1.45	1.4E-02	0	
FTN_0359		short-chain alcohol dehydrogenase-like dehydrogenase	5	-0.04	9.4E-01	0	
FTN_1150		GTP-binding protein	6	0.73	4.1E-01	0	
FTN_0880		hypothetical protein	2	-0.26	6.0E-01	0	
FTN_1312		hypothetical protein	3	-0.23	5.4E-01	0	
FTN_1776	trpD	Anthranilate phosphoribosyltransferase	3	-0.39	5.3E-01	0	Phenylalanine, tyrosine and tryptophan biosynthesis;Metabolic pathways;Biosynthesis of secondary metabolites;Biosynthesis of antibiotics;Biosynthesis of amino acids
FTN_1250	tilS	tRNA(Ile)-lysidine synthase	5	0.18	7.4E-01	0	
FTN_0113	ribC	riboflavin synthase subunit alpha	5	-0.44	4.2E-01	0	Riboflavin metabolism;Metabolic pathways;Biosynthesis of secondary metabolites
FTN_0630		hypothetical protein	2	1.02	5.0E-02	0	
FTN_0895		hypothetical protein	2	-0.13	7.8E-01	0	
FTN_1183		hypothetical protein	2	0.27	5.6E-01	0	
FTN_1256		hypothetical protein	2	-0.14	2.7E-01	0	
FTN_1036		hypothetical protein	2	0.13	2.9E-01	0	
FTN_1654		major facilitator transporter	2	-0.24	2.4E-01	0	
FTN_0405	sodC	superoxide dismutase (Cu-Zn) precusor	2	0.32	2.9E-01	0	
FTN_0762	grxC	glutaredoxin like protein	2	0.10	5.1E-01	0	
FTN_1162		hypothetical protein	2	-0.34	3.6E-01	0	
FTN_0339		arsenate reductase	2	-0.56	1.4E-01	0	
FTN_0467		major facilitator superfamily sugar transporter	2	-0.82	9.5E-02	0	
FTN_1179		LysR family transcriptional regulator	2	-0.55	2.7E-01	0	
FTN_0934		hypothetical protein	2	0.45	3.7E-02	0	
FTN_0707		type I restriction-modification system, subunit S	2	-0.63	2.5E-01	0	
FTN_0534		hypothetical protein	2	0.03	8.4E-01	0	
FTN_1530	lysA	diaminopimelate decarboxylase	2	-0.06	8.1E-01	0	Lysine biosynthesis;Metabolic pathways;Biosynthesis of secondary metabolites;Microbial metabolism in diverse environments;Biosynthesis of antibiotics;Biosynthesis of amino acids
FTN_1620	appB	cytochrome bd-II terminal oxidase subunit II	2	0.01	9.6E-01	0	Oxidative phosphorylation;Two-component system
FTN_0326		hypothetical protein	3	-0.18	2.1E-01	0	ABC transporters
FTN_1691	gatC	Glu-tRNAGln amidotransferase C subunit	3	-1.42	3.0E-02	0	Aminoacyl-tRNA biosynthesis;Metabolic pathways
FTN_1129		major facilitator transporter	3	-0.41	2.5E-01	0	
FTN_1495		hypothetical protein	3	-0.28	3.0E-01	0	
FTN_1052	miaA	tRNA dimethylallyltransferase	3	0.14	5.9E-01	0	Metabolic pathways;Biosynthesis of secondary metabolites
FTN_1533		hypothetical protein	3	-0.46	3.6E-01	0	
FTN_1353	panC	Pantothenate synthetase	3	0.72	6.4E-02	0	beta-Alanine metabolism;Pantothenate and CoA biosynthesis;Metabolic pathways;Biosynthesis of secondary metabolites
FTN_0573		hypothetical protein	3	-0.12	5.6E-01	0	
FTN_1741		hypothetical protein	3	-0.23	6.1E-01	0	
FTN_0123	recX	inhibitor of RecA	3	0.14	7.5E-01	0	
FTN_1217		ABC transporter ATP-binding protein	3	0.20	5.2E-01	0	
FTN_1378		hypothetical protein	3	-0.31	2.5E-01	0	
FTN_0651	cdd	cytidine deaminase	3	0.31	2.7E-01	0	Pyrimidine metabolism;Metabolic pathways
FTN_1293	rnhB	Ribonuclease HII	3	0.04	8.0E-01	0	DNA replication
FTN_0207		von Willebrand factor type A domain-containing protein	3	-0.37	8.3E-02	0	
FTN_0106	rpsT	30S ribosomal protein S20	3	0.41	1.1E-01	0	Ribosome
FTN_1368	feoA	Fe2+ transport system protein A	3	0.09	5.3E-01	0	
FTN_0005	corA	divalent inorganic cation transporter	3	0.94	2.7E-02	0	
FTN_1314		hypothetical protein	3	-0.29	4.1E-01	0	
FTN_1488		prophage maintenance system killer protein (DOC)	3	0.30	7.7E-01	0	
FTN_0747		amino acid-polyamine-organocation (APC) superfamily protein	3	-0.12	5.4E-01	0	
FTN_0821		AMP-binding enzyme	3	-0.19	2.5E-01	0	
FTN_0154	rimK	glutathione synthase/ribosomal protein S6 modification enzyme	3	0.64	7.1E-02	0	
FTN_0394		heavy metal cation transport ATPase	3	-0.04	7.4E-01	0	
FTN_1598	tmk	Thymidylate kinase	3	0.14	5.8E-01	0	Pyrimidine metabolism;Metabolic pathways
FTN_1303		DsbB family disulfide bond formation protein	3	-0.21	2.8E-01	0	
FTN_0371		hypothetical protein	3	0.35	2.0E-01	0	
FTN_1262	mreA	FAD binding family protein	3	0.73	2.4E-02	0	

FTN_0744		hypothetical protein	4	-0.35	1.7E-01	0	
FTN_1139	pilO	Type IV pili glycosylation protein	4	-0.06	8.6E-01	0	
FTN_1270		hypothetical protein	4	0.44	3.9E-01	0	
FTN_1359	recC	exodeoxyribonuclease V, gamma subunit	4	0.31	1.2E-01	0	Homologous recombination
FTN_0205		hypothetical protein	4	-0.42	1.3E-01	0	
FTN_0218	nfnB	dihydropteridine reductase	4	-0.03	8.7E-01	0	Nitrotoluene degradation;Microbial metabolism in diverse environments
FTN_0208		hypothetical protein	4	-0.21	3.5E-01	0	
FTN_0912	ndh	NADH dehydrogenase	4	0.70	2.6E-02	0	Oxidative phosphorylation
FTN_1490		hypothetical protein	4	0.01	9.7E-01	0	
FTN_0636	glpT	glycerol-3-phosphate transporter	4	0.55	3.1E-02	0	
FTN_0742	serB	phosphoserine phosphatase	4	-0.07	5.0E-01	0	Glycine, serine and threonine metabolism;Methane metabolism;Metabolic pathways;Microbial metabolism in diverse environments;Biosynthesis of antibiotics;Carbon metabolism;Biosynthesis of amino acids
FTN_0424		UPF0102 protein FTN_0424	4	0.64	1.4E-01	0	
FTN_0456		signal transduction protein	4	-0.55	1.7E-01	0	
FTN_0551	holB	DNA polymerase III, delta prime subunit	5	0.79	9.2E-02	0	DNA replication;Mismatch repair;Homologous recombination
FTN_1077		hypothetical protein	5	-0.31	2.7E-02	0	
FTN_1343		hypothetical protein	5	0.44	1.2E-01	0	
FTN_1446		hypothetical protein	5	0.19	7.4E-01	0	
FTN_1534		hypothetical protein	5	0.33	5.6E-01	0	
FTN_1006		HlyC/CorC family transporter-associated protein	5	-0.34	9.7E-02	0	
FTN_0399	radA	DNA repair protein RadA	5	-0.03	9.1E-01	0	
FTN_1326	anmK	Anhydro-N-acetylmuramic acid kinase	6	-0.11	7.9E-01	0	
FTN_0935	asnB	asparagine synthase	6	-0.66	4.1E-02	0	Alanine, aspartate and glutamate metabolism;Metabolic pathways;Biosynthesis of secondary metabolites;Biosynthesis of amino acids
FTN_1526		dihydrolipoamide dehydrogenase	6	-0.23	8.0E-02	0	
FTN_1466		hypothetical protein	6	-0.75	1.2E-01	0	
FTN_0694	nadB	L-aspartate oxidase	7	0.47	1.1E-02	0	Alanine, aspartate and glutamate metabolism;Nicotinate and nicotinamide metabolism;Metabolic pathways
FTN_1452		two-component response regulator	7	0.23	4.1E-02	0	
FTN_1287	hemG	hypothetical protein	7	0.15	5.9E-01	0	
FTN_1165	ttcA2	tRNA 2-thiocytidine biosynthesis protein TtcA 2	8	-0.15	5.0E-01	0	
FTN_0320		hypothetical protein	9	0.50	8.2E-03	0	
FTN_1432	hrpA	HrpA-like helicase	10	-0.54	3.8E-01	0	
FTN_0257	rpmD	50S ribosomal protein L30	2	-0.07	6.7E-01	0	Ribosome
FTN_0350	pssA	CDP-alcohol phosphatidyltransferase	2	0.00	9.9E-01	0	Glycine, serine and threonine metabolism;Glycerophospholipid metabolism;Metabolic pathways;Biosynthesis of secondary metabolites
FTN_0506	gcvH	glycine cleavage system H protein	2	0.26	4.4E-02	0	Glycine, serine and threonine metabolism;Glyoxylate and dicarboxylate metabolism;Metabolic pathways;Biosynthesis of secondary metabolites;Biosynthesis of antibiotics;Carbon metabolism
FTN_1291	mglB	ClpXP protease specificity-enhancing factor	2	-0.05	7.2E-01	0	
FTN_0982	grxA	glutaredoxin 1	2	0.34	1.8E-01	0	
FTN_1608	dsbB	disulfide bond formation protein	2	-0.09	4.3E-01	0	
FTN_0213	holC	DNA polymerase III (chi subunit) protein	2	-0.22	4.0E-01	0	DNA replication;Mismatch repair;Homologous recombination
FTN_0353	tolR	TolR protein	2	-0.05	7.9E-01	0	
FTN_1552		acid phosphatase	2	0.03	8.8E-01	0	
FTN_0620		major facilitator transporter	2	-0.12	4.8E-01	0	
FTN_1651	atpE	F0F1 ATP synthase subunit C	2	0.08	8.0E-01	0	Oxidative phosphorylation;Metabolic pathways
FTN_0414		Type IV pili, pilus assembly protein	2	-0.12	2.4E-01	0	
FTN_1232		hypothetical protein	2	0.15	6.8E-01	0	
FTN_0556		hypothetical protein	2	-0.13	5.9E-01	0	
FTN_1297		hypothetical protein	2	-0.31	1.9E-01	0	
FTN_0416	lpxE	lipid A 1-phosphatase	2	0.22	1.8E-01	0	
FTN_0160		zinc-binding protein	2	-0.34	3.3E-01	0	
FTN_0806		glycosy hydrolase family protein	2	0.35	2.2E-01	0	Amino sugar and nucleotide sugar metabolism;Metabolic pathways;beta-Lactam resistance
FTN_0723	cspA	cold shock protein	2	0.74	2.2E-01	0	
FTN_0400		hypothetical protein	2	0.09	5.8E-01	0	
FTN_1543	rlmH	Ribosomal RNA large subunit methyltransferase H	2	0.09	3.7E-01	0	
FTN_1079		major facilitator superfamily sugar transporter	2	0.08	6.5E-01	0	
FTN_0437		HD superfamily hydrolase	2	0.26	6.5E-01	0	
FTN_0622	ftsB	cell division protein, septum formation initiator	2	0.06	7.6E-01	0	
FTN_1638	sdhD	succinate dehydrogenase hydrophobic membrane anchor protein	2	0.00	9.9E-01	0	Citrate cycle (TCA cycle);Oxidative phosphorylation;Butanoate metabolism;Metabolic pathways;Biosynthesis of secondary metabolites;Microbial metabolism in diverse environments;Biosynthesis of antibiotics;Carbon metabolism
FTN_0162	ftsQ	cell division protein FtsQ	2	0.25	2.8E-01	0	

FTN_0335	recG	ATP-dependent DNA helicase RecG	2	0.13	8.2E-01	0	Homologous recombination
FTN_0156	plsC	putative acyltransferase	2	-0.05	8.3E-01	0	
FTN_0664	fimT	Type IV pili, pilus assembly protein	2	-0.17	4.5E-01	0	
FTN_0185		outer membrane lipoprotein	2	-0.16	1.3E-01	0	
FTN_0105		outer membrane lipoprotein	2	0.28	2.9E-02	0	
FTN_0676	rpmA	50S ribosomal protein L27	2	-0.19	1.4E-01	0	Ribosome
FTN_1082		hypothetical protein	2	0.51	2.0E-01	0	
FTN_0894		GDSL-like lipolytic enzyme	2	-0.48	5.9E-01	0	
FTN_0978	coq7	2-nonaprenyl-3-methyl-6-methoxy-1,4-benzoquinol hydroxylase	2	0.56	1.2E-03	0	Ubiquinone and other terpenoid-quinone biosynthesis;Metabolic pathways;Biosynthesis of secondary metabolites
FTN_1709		amino acid transporter	2	0.46	5.0E-03	0	
FTN_0093		cytochrome b561 family protein	2	-0.40	2.8E-02	0	
FTN_0528	lpxH	UDP-2,3-diacylglucosamine hydrolase	2	0.39	1.0E-01	0	Lipopolysaccharide biosynthesis;Metabolic pathways
FTN_0848		amino acid antiporter	2	0.32	1.0E-01	0	Quorum sensing
FTN_0296	lysP	lysine:H+ symporter	2	0.29	4.6E-02	0	
FTN_0233	dut	Deoxyuridine 5'-triphosphate nucleotidohydrolase	3	-0.02	9.0E-01	0	Pyrimidine metabolism;Metabolic pathways
FTN_0643		hypothetical protein	3	-0.01	9.7E-01	0	
FTN_0235	rpsL	30S ribosomal protein S12	3	-0.09	3.4E-01	0	Ribosome
FTN_0697		hypothetical protein	3	0.14	2.7E-01	0	
FTN_0617		ROK family protein	3	0.45	1.4E-02	0	
FTN_1559	rplS	50S ribosomal protein L19	3	-0.08	4.7E-01	0	Ribosome
FTN_0247	rpmC	50S ribosomal protein L29	3	-0.89	9.2E-02	0	Ribosome
FTN_1668	nuoM	NADH dehydrogenase I, M subunit	3	0.13	4.4E-01	0	Oxidative phosphorylation;Metabolic pathways
FTN_1105	plsY	Glycerol-3-phosphate acyltransferase	3	-0.01	9.7E-01	0	Glycerolipid metabolism;Glycerophospholipid metabolism;Metabolic pathways;Biosynthesis of secondary metabolites
FTN_1507		hypothetical protein	3	-0.14	6.7E-01	0	
FTN_0610	erpA	Iron-sulfur cluster insertion protein erpA	3	0.10	2.0E-01	0	
FTN_1737	eriC	chloride channel protein	3	-0.03	8.8E-01	0	
FTN_0092		inorganic phosphate transporter (PiT) family protein	3	1.04	2.5E-02	0	
FTN_0767	betT	betaine/carnitine/choline transporter (BCCT) family protein	3	1.07	1.0E-02	0	
FTN_0791		hypothetical protein	3	0.05	7.4E-01	0	
FTN_0802		hypothetical protein	3	-0.15	7.7E-02	0	
FTN_0568		birA-like protein	3	-0.06	5.8E-01	0	Biotin metabolism;Metabolic pathways
FTN_1147		glutamine amidotransferases class-II family protein	3	-0.24	1.8E-01	0	
FTN_1340	acpP	acyl carrier protein	3	0.07	6.6E-01	0	Metabolic pathways;Biosynthesis of antibiotics
FTN_1118		hypothetical protein	3	0.46	1.1E-02	0	
FTN_0039		hypothetical protein	3	0.26	1.4E-01	0	
FTN_0328		ABC transporter ATP-binding protein	3	-0.05	7.6E-01	0	ABC transporters
FTN_1732		Mg-dependent DNase	3	0.73	1.7E-02	0	
FTN_1295	ptsN	PEP-dependent sugar phosphotransferase system (PTS) family protein	3	-0.02	9.4E-01	0	
FTN_1154		type I restriction-modification system, subunit S	3	0.28	6.3E-01	0	
FTN_0552	yhbY	RNA-binding protein	3	-0.33	3.5E-01	0	
FTN_1081		dicarboxylate/amino acid:cation (Na+ or H+) symporter	3	0.14	4.9E-01	0	
FTN_1130		amino acid transporter	3	0.12	4.2E-01	0	
FTN_0547		Probable Fe(2+)-trafficking protein	3	0.12	3.7E-01	0	
FTN_1681	fur	ferric uptake regulation protein	3	-0.02	8.7E-01	0	
FTN_1282		LysR family transcriptional regulator	3	0.06	6.5E-01	0	
FTN_0333	rpmB	50S ribosomal protein L28	3	-0.04	6.7E-01	0	Ribosome
FTN_1046	wzb	low molecular weight (LMW) phosphotyrosine protein phosphatase	3	-0.09	7.3E-01	0	Two-component system
FTN_0465		Sua5/YciO/YrdC family protein	3	0.08	4.2E-01	0	
FTN_0586		hypothetical protein	3	-0.03	9.3E-01	0	
FTN_0960		hypothetical protein	3	0.03	8.9E-01	0	
FTN_0677		hypothetical protein	3	-0.12	4.7E-01	0	
FTN_1373	hemD	uroporphyrinogen-III synthase	3	-0.03	8.1E-01	0	Porphyrin and chlorophyll metabolism;Metabolic pathways;Biosynthesis of secondary metabolites;Microbial metabolism in diverse environments
FTN_1271		16S ribosomal RNA methyltransferase RsmE	3	-0.10	3.5E-01	0	
FTN_0079	murC	UDP-N-acetylmuramate--L-alanine ligase	3	0.55	2.4E-01	0	D-Glutamine and D-glutamate metabolism;Peptidoglycan biosynthesis;Metabolic pathways
FTN_0570	perM	PerM family protein	3	-0.81	2.1E-02	0	
FTN_1560	trmD	tRNA (guanine-N(1)-)-methyltransferase	3	0.58	2.7E-02	0	
FTN_0566		mechanosensitive ion channel protein	3	0.17	3.1E-01	0	
FTN_0117		ferredoxin	3	0.22	4.5E-02	0	
FTN_1155		type I restriction-modification system, subunit R (restriction)	3	0.07	7.8E-01	0	
FTN_0201		hypothetical protein	3	-0.15	4.5E-01	0	
FTN_0324		hypothetical protein	3	0.04	8.8E-01	0	ABC transporters
FTN_0404		methionine sulfoxide reductase B	3	-0.04	6.5E-01	0	

FTN_1320		hypothetical protein	3	0.61	8.3E-02	0	
FTN_0143		monovalent cation:proton antiporter	3	-0.07	8.1E-01	0	
FTN_0150		YggT family protein	3	0.36	2.4E-02	0	
FTN_0608	rpsO	30S ribosomal protein S15	4	-0.01	9.3E-01	0	Ribosome
FTN_0258	rplO	50S ribosomal protein L15	4	-0.03	8.2E-01	0	Ribosome
FTN_1289	rpsI	30S ribosomal protein S9	4	0.00	9.6E-01	0	Ribosome
FTN_1630	secG	preprotein translocase subunit SecG	4	0.00	1.0E+00	0	Quorum sensing;Protein export;Bacterial secretion system
FTN_0271	ndk	Nucleoside diphosphate kinase	4	-0.05	6.1E-01	0	Purine metabolism;Pyrimidine metabolism;Metabolic pathways;Biosynthesis of secondary metabolites;Biosynthesis of antibiotics
FTN_0278	rpmE	50S ribosomal protein L31	4	-0.53	1.1E-02	0	Ribosome
FTN_1280		tryptophan repressor binding protein-like flavidoxin	4	0.17	6.2E-01	0	
FTN_0241	rplW	50S ribosomal protein L23	4	-0.12	3.3E-01	0	Ribosome
FTN_0488	cspC	cold shock protein	4	0.06	8.5E-01	0	
FTN_0318		hypothetical protein	4	0.14	5.2E-01	0	
FTN_0186	wrbA	trp repressor binding protein	4	0.00	1.0E+00	0	Ubiquinone and other terpenoid-quinone biosynthesis;Metabolic pathways;Biosynthesis of secondary metabolites
FTN_1096	yajC	preprotein translocase family protein	4	-0.07	4.8E-01	0	Quorum sensing;Protein export;Bacterial secretion system
FTN_1194	gcvH1	glycine cleavage system protein H	4	-0.11	4.1E-01	0	Glycine, serine and threonine metabolism;Glyoxylate and dicarboxylate metabolism;Metabolic pathways;Biosynthesis of secondary metabolites;Biosynthesis of antibiotics;Carbon metabolism
FTN_0428		hypothetical protein	4	-0.36	1.5E-01	0	
FTN_1110	ispF	2-C-methyl-D-erythritol 2,4-cyclodiphosphate synthase	4	0.00	9.8E-01	0	Terpenoid backbone biosynthesis;Metabolic pathways;Biosynthesis of secondary metabolites;Biosynthesis of antibiotics
FTN_0157		hypothetical protein	4	-0.01	9.1E-01	0	
FTN_0605	rsmH	Ribosomal RNA small subunit methyltransferase H	4	0.63	4.3E-01	0	
FTN_1133		hypothetical protein	4	0.20	2.6E-02	0	
FTN_0203		hypothetical protein	4	-0.38	4.4E-02	0	
FTN_0919		hypothetical protein	4	0.19	1.3E-01	0	
FTN_0248	rpsQ	30S ribosomal protein S17	4	0.01	9.3E-01	0	Ribosome
FTN_1188	rplT	50S ribosomal protein L20	4	-0.11	4.2E-01	0	Ribosome
FTN_1311		hypothetical protein	4	0.22	2.1E-01	0	
FTN_0274		hypothetical protein	4	-0.02	9.1E-01	0	
FTN_1402	ppnK	Probable inorganic polyphosphate/ATP-NAD kinase	4	0.39	3.4E-01	0	Nicotinate and nicotinamide metabolism;Metabolic pathways
FTN_0658		membrane protein of unknown function	4	-0.44	1.7E-02	0	ABC transporters
FTN_0892		short chain dehydrogenase family protein	4	0.18	2.6E-01	0	
FTN_0582	gph	phosphoglycolate phosphatase	4	0.24	4.4E-01	0	Glyoxylate and dicarboxylate metabolism;Metabolic pathways;Biosynthesis of secondary metabolites;Biosynthesis of antibiotics
FTN_0124	ssb	single-strand DNA binding protein	4	-0.23	2.9E-01	0	DNA replication;Mismatch repair;Homologous recombination
FTN_1641	ampG	major facilitator transporter	4	-0.01	9.1E-01	0	beta-Lactam resistance
FTN_0068	orn	Oligoribonuclease	4	0.31	6.9E-03	0	
FTN_0908		hypothetical protein	4	0.13	2.0E-01	0	
FTN_0653	miaE	tRNA-(ms(2)io(6)a)-hydroxylase	4	-0.29	5.9E-02	0	
FTN_1230		hypothetical protein	4	-0.02	8.6E-01	0	
FTN_0967	vanY	D-alanyl-D-alanine carboxypeptidase	4	-0.57	2.8E-02	0	Peptidoglycan biosynthesis;Metabolic pathways;Vancomycin resistance;Two-component system
FTN_1599	nupC	nucleoside permease NUP family protein	4	-0.17	1.7E-01	0	
FTN_1429	wbtP	galactosyl transferase	4	-0.21	2.5E-01	0	
FTN_1384	nusB	transcription termination factor	4	0.10	2.1E-01	0	
FTN_1034	rnfB	iron-sulfur cluster-binding protein	4	0.13	6.0E-01	0	
FTN_0910		sugar:cation symporter family protein	4	-1.07	9.8E-02	0	
FTN_1392		rhodanese-related sulfurtransferase	4	-0.85	9.9E-02	0	
FTN_1003	pth	Peptidyl-tRNA hydrolase	4	-0.51	1.2E-02	0	
FTN_1356	recD	exodeoxyribonuclease V, alpha subunit	4	1.01	7.4E-02	0	Homologous recombination
FTN_0785		isochorismatase family protein	4	0.08	7.8E-01	0	
FTN_0243	rpsS	30S ribosomal protein S19	4	-0.07	5.0E-01	0	Ribosome
FTN_1673	nuoH	NADH-quinone oxidoreductase subunit H	4	-0.20	2.7E-01	0	Oxidative phosphorylation;Metabolic pathways
FTN_1035	nth	endonuclease III	4	-0.31	1.3E-01	0	Base excision repair
FTN_0323		BolA family protein	4	0.03	7.3E-01	0	
FTN_1435		arsenate reductase	4	0.13	4.7E-01	0	
FTN_1669	nuoL	NADH dehydrogenase I, L subunit	4	0.22	7.1E-02	0	Oxidative phosphorylation;Metabolic pathways
FTN_0688	galP2	major facilitator superfamily galactose-proton symporter	4	-0.15	5.8E-01	0	
FTN_0944		hypothetical protein	4	1.05	1.5E-02	0	
FTN_1012		small conductance mechanosensitive ion channel family protein	4	0.48	1.5E-01	0	
FTN_1239		5-formyltetrahydrofolate cycloligase	4	0.13	5.3E-01	0	One carbon pool by folate;Metabolic pathways
FTN_1117		ATP binding protein	4	-0.18	5.0E-01	0	
FTN_0820	folB	dihydroneopterin aldolase	4	-0.20	1.8E-01	0	Folate biosynthesis;Metabolic pathways

FTN_0928	cysD	sulfate adenylyltransferase subunit 2	4	0.16	5.5E-01	0	Purine metabolism;Monobactam biosynthesis;Selenocompound metabolism;Sulfur metabolism;Metabolic pathways;Microbial metabolism in diverse environments;Biosynthesis of antibiotics
FTN_0603	mutM	formamidopyrimidine-DNA glycosylase	4	0.30	1.9E-01	0	Base excision repair
FTN_1318		hypothetical protein	4	0.04	9.3E-01	0	
FTN_0158		Sua5/YciO/YrdC family protein	4	0.35	3.3E-01	0	
FTN_1138	pilP	Type IV pili periplasmic component	4	0.29	2.9E-02	0	
FTN_1428	wbtO	transferase	4	-0.22	2.0E-01	0	
FTN_1374	sufE	sulfur acceptor protein SufE	4	-0.02	9.3E-01	0	
FTN_1164	smpB	SsrA-binding protein	4	-0.44	1.2E-01	0	
FTN_1184		hypothetical protein	4	0.04	7.7E-01	0	
FTN_1753		Rieske (2Fe-2S) domain-containing protein	4	0.12	4.3E-01	0	
FTN_0388		hypothetical protein	4	-0.29	3.8E-01	0	
FTN_0832		proton-dependent oligopeptide transporter (POT) family protein, di- or tripeptide:H+ symporter	4	-0.01	9.8E-01	0	
FTN_1240		hypothetical protein	4	0.09	4.3E-01	0	
FTN_0202	pdxY	pyridoxal kinase	4	-0.07	7.8E-01	0	Vitamin B6 metabolism;Metabolic pathways
FTN_1500		hypothetical protein	4	-0.11	6.0E-01	0	
FTN_0526	thrB	homoserine kinase	4	-0.01	9.4E-01	0	Glycine, serine and threonine metabolism;Metabolic pathways;Biosynthesis of secondary metabolites;Microbial metabolism in diverse environments;Biosynthesis of amino acids
FTN_0994		hypothetical protein	4	0.19	6.0E-01	0	
FTN_0866		UPF0301 protein FTN_0866	4	0.49	1.1E-01	0	
FTN_0255	rplR	50S ribosomal protein L18	5	-0.11	2.9E-01	0	Ribosome
FTN_1716	kdpC	potassium-transporting ATPase C chain	5	0.10	3.0E-01	0	Two-component system
FTN_1679	nuoB	NADH-quinone oxidoreductase subunit B	5	-0.02	8.6E-01	0	Oxidative phosphorylation;Metabolic pathways
FTN_1748		4Fe-4S ferredoxin	5	-0.07	4.5E-01	0	
FTN_0289	proQ	activator of osmoprotectant transporter ProP	5	0.08	4.6E-01	0	
FTN_0408		mannose-6-phosphate isomerase	5	0.07	3.6E-01	0	
FTN_1382		hypothetical protein	5	0.02	8.4E-01	0	
FTN_0069	efp	Elongation factor P	5	0.00	1.0E+00	0	
FTN_0430		hypothetical protein	5	-0.03	6.7E-01	0	
FTN_0716		hypothetical protein	5	-0.71	1.7E-02	0	
FTN_1196		UPF0133 protein FTN_1196	5	0.08	6.3E-01	0	
FTN_1627		hypothetical protein	5	-0.12	5.6E-01	0	
FTN_0269		hypothetical protein	5	-0.12	7.8E-01	0	
FTN_0144	paaY	carbonic anhydrase	5	-0.15	3.8E-01	0	
FTN_0495		BNR/Asp-box repeat-containing protein	5	0.17	2.6E-01	0	
FTN_0450		hypothetical protein	5	-0.07	5.0E-01	0	
FTN_1369		hypothetical protein	5	0.28	6.6E-02	0	
FTN_0329	minE	Cell division topological specificity factor	5	-0.01	9.4E-01	0	
FTN_0639		endoribonuclease L-PSP	5	-0.16	1.6E-01	0	
FTN_1248	thyA	Thymidylate synthase	5	-0.10	3.0E-01	0	Pyrimidine metabolism;One carbon pool by folate;Metabolic pathways
FTN_0613	rpoZ	DNA-directed RNA polymerase subunit omega	5	0.17	1.5E-01	0	RNA polymerase
FTN_1083		hypothetical protein	5	0.56	9.0E-02	0	
FTN_0065		hypothetical protein	5	0.09	5.5E-01	0	
FTN_1659	rbfA	Ribosome-binding factor A	5	0.09	2.2E-01	0	
FTN_0576		hypothetical protein	5	0.01	9.3E-01	0	
FTN_1231	gloA	lactoylglutathione lyase	5	0.26	5.8E-02	0	Pyruvate metabolism;Metabolic pathways
FTN_0777		hypothetical protein	5	-0.24	1.3E-01	0	
FTN_0114	ribD	pyrimidine reductase/pyrimidine deaminase	5	0.36	3.5E-01	0	Riboflavin metabolism;Metabolic pathways;Biosynthesis of secondary metabolites;Quorum sensing
FTN_1342		hypothetical protein	5	0.06	6.9E-01	0	
FTN_1769		HSP20 family protein	5	0.48	1.2E-01	0	
FTN_0491		hypothetical protein	5	0.73	9.8E-02	0	
FTN_0642	cydD	cysteine/glutathione ABC transporter membrane/ATP-binding component	5	-0.50	4.3E-03	0	ABC transporters
FTN_1718	kdpA	Potassium-transporting ATPase A chain	5	0.01	9.4E-01	0	Two-component system
FTN_0864		hypothetical protein	5	0.18	4.7E-01	0	
FTN_0520	murE	UDP-N-acetylmuramoylalanyl-D-glutamate--2,6- diaminopimelate ligase	5	0.34	1.1E-01	0	Lysine biosynthesis;Peptidoglycan biosynthesis;Metabolic pathways
FTN_0352	tolQ	TolQ protein	5	-0.02	8.8E-01	0	
FTN_1062		o-methyltransferase family protein	5	-0.06	7.6E-01	0	
FTN_1551	ampD	N-acetyl-anhydromuranmyl-L-alanine amidase	5	0.30	1.1E-01	0	
FTN_0774		hypothetical protein	5	0.01	9.1E-01	0	
FTN_0290		hypothetical protein	5	-0.49	3.4E-02	0	
FTN_0988	prmA	50S ribosomal protein L11, methyltransferase	5	0.07	8.4E-01	0	
FTN_0763		thioesterase superfamily protein	5	0.06	6.4E-01	0	

FTN_0634		short chain dehydrogenase/reductase family oxidoreductase	5	-0.02	9.5E-01	0	
FTN_0554		RNA methyltransferase	5	0.37	3.3E-01	0	
FTN_0483		bifunctional nicotinamide mononucleotide adenylyltransferase/ADP-ribose pyrophosphatase	5	0.25	7.0E-01	0	Nicotinate and nicotinamide metabolism;Metabolic pathways
FTN_0753		hypothetical protein	5	0.50	2.3E-02	0	
FTN_0574		GTPase	5	-0.02	9.1E-01	0	Thiamine metabolism;Metabolic pathways
FTN_1652	atpB	F0F1 ATP synthase subunit A	5	0.10	5.0E-01	0	Oxidative phosphorylation;Metabolic pathways
FTN_1211		haloacid dehalogenase-like hydrolase	5	-0.32	1.6E-01	0	
FTN_0569	recJ	single-stranded-DNA-specific exonuclease	5	0.90	1.5E-01	0	Base excision repair;Mismatch repair;Homologous recombination
FTN_1236	ubiF	2-octaprenyl-3-methyl-6-methoxy-1,4-benzoquinol hydroxylase	5	0.53	6.2E-03	0	Ubiquinone and other terpenoid-quinone biosynthesis;Metabolic pathways;Biosynthesis of secondary metabolites
FTN_1024		RmuC family protein	5	0.23	3.6E-01	0	
FTN_1760		zinc-binding alcohol dehydrogenase	5	-0.40	5.6E-02	0	
FTN_0042		hypothetical protein	5	0.32	1.0E-01	0	
FTN_0458	hitA	histidine triad (HIT) family protein	5	0.16	2.2E-01	0	
FTN_0830	folE	GTP cyclohydrolase I	5	0.04	8.5E-01	0	Folate biosynthesis;Metabolic pathways
FTN_0916	rpsU	30S ribosomal protein S21	5	0.03	8.5E-01	0	Ribosome
FTN_0562	aroQ	3-dehydroquinate dehydratase	5	0.02	8.5E-01	0	Phenylalanine, tyrosine and tryptophan biosynthesis;Metabolic pathways;Biosynthesis of secondary metabolites;Biosynthesis of antibiotics;Biosynthesis of amino acids
FTN_0803	fmt	Methionyl-tRNA formyltransferase	5	0.03	7.9E-01	0	One carbon pool by folate;Aminoacyl-tRNA biosynthesis
FTN_0311		N5-glutamine S-adenosyl-L-methionine-dependent methyltransferase	5	0.09	4.5E-01	0	
FTN_1140	pilN	Type IV pili associated protein	5	-0.02	9.3E-01	0	
FTN_0180		dienelactone hydrolase family protein	5	0.23	3.8E-01	0	
FTN_0679	rnd	ribonuclease D	5	0.30	2.8E-02	0	
FTN_0321	ubiG	3-demethylubiquinone-9 3-methyltransferase	5	0.70	1.1E-02	0	Ubiquinone and other terpenoid-quinone biosynthesis;Metabolic pathways;Biosynthesis of secondary metabolites
FTN_0338		MutT/nudix family protein	5	-0.14	5.8E-01	0	
FTN_1398		acetyltransferase	5	-0.04	7.2E-01	0	
FTN_1489		hypothetical protein	5	-0.10	7.7E-01	0	
FTN_1511		hypothetical protein	5	-0.54	1.7E-01	0	
FTN_0657		M16 family metallopeptidase	5	0.21	4.2E-01	0	
FTN_0220	fumC	fumarate hydratase	5	0.46	3.4E-02	0	Citrate cycle (TCA cycle);Pyruvate metabolism;Metabolic pathways;Biosynthesis of secondary metabolites;Microbial metabolism in diverse environments;Biosynthesis of antibiotics;Carbon metabolism
FTN_0173	holA	DNA polymerase III, delta subunit	5	0.54	6.6E-02	0	DNA replication;Mismatch repair;Homologous recombination
FTN_1400	trmB	tRNA (guanine-N(7)-)-methyltransferase	5	0.64	5.3E-02	0	
FTN_0581		phospholipase D family protein	5	-0.49	5.3E-01	0	
FTN_0599		hypothetical protein	5	-0.57	2.5E-03	0	
FTN_0682		UPF0434 protein FTN_0682	5	-0.65	1.4E-02	0	
FTN_0855		hypothetical protein	6	0.20	1.8E-01	0	
FTN_0340		hypothetical protein	6	-0.06	7.3E-01	0	
FTN_1481	ompH	outer membrane protein OmpH	6	-0.11	5.4E-01	0	
FTN_1007	rplY	50S ribosomal protein L25	6	-0.09	8.3E-01	0	Ribosome
FTN_1278	nadE	NAD synthase	6	0.10	7.7E-01	0	Nicotinate and nicotinamide metabolism;Metabolic pathways
FTN_0782		hypothetical protein	6	-0.21	3.6E-01	0	
FTN_0675	rplU	50S ribosomal protein L21	6	-0.09	7.3E-01	0	Ribosome
FTN_0503		ABC transporter, ATPase component	6	-0.07	5.1E-01	0	ABC transporters
FTN_0439		hypothetical protein	6	0.09	4.4E-01	0	
FTN_1735		hypothetical protein	6	-0.05	6.9E-01	0	
FTN_0810		ROK family protein	6	0.04	7.7E-01	0	Glycolysis / Gluconeogenesis;Galactose metabolism;Starch and sucrose metabolism;Amino sugar and nucleotide sugar metabolism;Streptomycin biosynthesis;Metabolic pathways;Biosynthesis of secondary metabolites;Microbial metabolism in diverse environments;Biosynthesis of antibiotics;Carbon metabolism
FTN_1601	deoC	deoxyribose-phosphate aldolase	6	-0.05	5.0E-01	0	Pentose phosphate pathway;Metabolic pathways
FTN_0754	coaD	Phosphopantetheine adenylyltransferase	6	0.03	8.0E-01	0	Pantothenate and CoA biosynthesis;Metabolic pathways
FTN_0850		transcriptional regulator	6	-0.21	2.4E-01	0	
FTN_0036	pyrD	dihydroorotate oxidase	6	0.31	3.4E-02	0	Pyrimidine metabolism;Metabolic pathways
FTN_0449		hypothetical protein	6	0.14	3.1E-01	0	
FTN_1067		apolipoprotein N-acyltransferase	6	-0.18	3.0E-01	0	
FTN_0130		glycosyl transferase, group 1	6	0.01	9.1E-01	0	
FTN_0577	mutL	DNA mismatch repair protein	6	0.20	3.3E-01	0	Mismatch repair
FTN_1385		hypothetical protein	6	0.17	2.9E-01	0	
FTN_1695		hypothetical protein	6	-0.37	1.5E-02	0	
FTN_0297		hypothetical protein	6	-0.13	4.0E-01	0	
FTN_0174	blc	outer membrane lipoprotein	6	0.26	8.4E-02	0	

FTN_0481	psd	Phosphatidylserine decarboxylase proenzyme	6	-0.46	6.0E-04	0	Glycerophospholipid metabolism;Metabolic pathways;Biosynthesis of secondary metabolites
FTN_0250	rplX	50S ribosomal protein L24	6	-0.12	2.7E-01	0	Ribosome
FTN_1221	rpe	D-ribulose-phosphate 3-epimerase	6	-0.07	4.7E-01	0	Pentose phosphate pathway;Pentose and glucuronate interconversions;Metabolic pathways;Biosynthesis of secondary metabolites;Microbial metabolism in diverse environments;Biosynthesis of antibiotics;Carbon metabolism;Biosynthesis of amino acids
FTN_1273		long chain fatty acid CoA ligase	6	-0.01	9.7E-01	0	Fatty acid biosynthesis;Fatty acid degradation;Metabolic pathways;Fatty acid metabolism;Quorum sensing
FTN_0918		hypothetical protein	6	0.21	7.0E-02	0	
FTN_0169		hypothetical protein	6	-0.02	9.5E-01	0	
FTN_0828		hypothetical protein	6	-0.28	3.3E-01	0	
FTN_1075		hypothetical protein	6	-0.15	6.5E-01	0	
FTN_0702		YjeF-related protein	6	0.29	5.8E-01	0	
FTN_1777	trpG	anthranilate synthase component II	6	-0.20	2.8E-01	0	Phenylalanine, tyrosine and tryptophan biosynthesis;Metabolic pathways;Biosynthesis of secondary metabolites;Biosynthesis of antibiotics;Biosynthesis of amino acids;Quorum sensing
FTN_0511		shikimate 5-dehydrogenase	6	0.13	2.8E-01	0	Phenylalanine, tyrosine and tryptophan biosynthesis;Metabolic pathways;Biosynthesis of secondary metabolites;Biosynthesis of antibiotics;Biosynthesis of amino acids
FTN_1519		hypothetical protein	6	-0.47	3.9E-02	0	
FTN_1458		hypothetical protein	6	0.20	3.9E-01	0	
FTN_0835		hypothetical protein	6	-0.23	1.2E-01	0	
FTN_0426		hypothetical protein	6	0.14	5.1E-01	0	
FTN_1269		hypothetical protein	6	0.15	1.7E-01	0	
FTN_0448	murB	UDP-N-acetylenolpyruvoylglucosamine reductase	6	0.92	5.1E-02	0	Amino sugar and nucleotide sugar metabolism;Peptidoglycan biosynthesis;Metabolic pathways
FTN_0531	yjfH	tRNA/rRNA methyltransferase	6	0.11	4.1E-01	0	
FTN_0766	spoU	rRNA methyltransferase	6	0.04	7.0E-01	0	
FTN_1071		hypothetical protein	6	-0.15	4.3E-01	0	
FTN_0998		potassium channel protein	6	-0.40	2.9E-01	0	
FTN_0392		LysR family transcriptional regulator	6	-0.20	1.1E-01	0	
FTN_1019	hdc	histidine decarboxylase	6	-0.02	9.1E-01	0	Histidine metabolism;Metabolic pathways;Biosynthesis of secondary metabolites
FTN_1237	ubiH	2-octaprenyl-6-methoxyphenyl hydroxylase	6	0.34	1.1E-02	0	Ubiquinone and other terpenoid-quinone biosynthesis;Metabolic pathways;Biosynthesis of secondary metabolites
FTN_0814	bioF	8-amino-7-oxononanoate synthase	6	0.11	8.4E-01	0	Biotin metabolism;Metabolic pathways
FTN_1235		hypothetical protein	6	0.20	1.9E-01	0	
FTN_1265		hypothetical protein	6	0.10	4.7E-01	0	
FTN_1073		DNA/RNA endonuclease G	6	-1.03	1.2E-02	0	
FTN_1403	gtrB	glycosyl transferase	6	0.14	1.9E-01	0	
FTN_0833		UPF0145 protein FTN_0833	6	-0.15	3.1E-01	0	
FTN_0931		hypothetical protein	6	0.14	3.1E-01	0	
FTN_0868		membrane protein of unknown function	6	-0.22	7.5E-02	0	
FTN_0048		hypothetical protein	6	0.24	3.6E-01	0	
FTN_1264	rluD	ribosomal large subunit pseudouridine synthase D	6	-0.20	1.1E-01	0	
FTN_0718		membrane fusion protein	6	-0.58	2.3E-02	0	
FTN_1605	lpxK	Tetraacyldisaccharide 4'-kinase	6	-0.16	2.3E-01	0	Lipopolysaccharide biosynthesis;Metabolic pathways
FTN_1655	rluC	ribosomal large subunit pseudouridine synthase C	6	-0.06	8.9E-01	0	
FTN_0956	cmk	cytidylate kinase	6	0.04	7.2E-01	0	Pyrimidine metabolism;Metabolic pathways
FTN_0816	bioA	adenosylmethionine-8-amino-7-oxononanoate aminotransferase	6	0.39	3.1E-01	0	Biotin metabolism;Metabolic pathways
FTN_0349	aroH	chorismate mutase	6	0.17	9.7E-02	0	Phenylalanine, tyrosine and tryptophan biosynthesis;Metabolic pathways;Biosynthesis of secondary metabolites;Biosynthesis of antibiotics;Biosynthesis of amino acids
FTN_1197	recR	Recombination protein recR	6	-0.48	3.4E-03	0	Homologous recombination
FTN_1002	blaA	beta-lactamase class A	6	-0.38	1.6E-02	0	Biosynthesis of antibiotics;beta-Lactam resistance
FTN_1714	kdpE	two-component response regulator	6	0.14	1.3E-01	0	Two-component system;Quorum sensing
FTN_0135	hemC	Porphobilinogen deaminase	6	0.14	3.2E-01	0	Porphyrin and chlorophyll metabolism;Metabolic pathways;Biosynthesis of secondary metabolites;Microbial metabolism in diverse environments
FTN_0546		dolichyl-phosphate-mannose-protein mannosyltransferase family protein	6	0.43	4.1E-03	0	
FTN_0721		hypothetical protein	6	0.04	7.7E-01	0	
FTN_1656		ATPase	6	-0.27	1.9E-01	0	
FTN_1413		recombination factor protein RarA	6	0.05	7.2E-01	0	
FTN_1215	kpsC	capsule polysaccharide export protein KpsC	6	-0.72	1.9E-02	0	
FTN_1505		hypothetical protein	6	0.25	1.5E-01	0	
FTN_0056	dtd	D-tyrosyl-tRNA(Tyr) deacylase	6	0.30	1.1E-01	0	
FTN_0082		HAD superfamily hydrolase	6	0.38	2.9E-01	0	

FTN_0262	rpsK	30S ribosomal protein S11	7	-0.07	5.0E-01	0	Ribosome
FTN_0689	ppiC	parvulin-like peptidyl-prolyl isomerase domain-containing protein	7	-0.07	7.0E-01	0	
FTN_0254	rplF	50S ribosomal protein L6	7	-0.04	6.8E-01	0	Ribosome
FTN_0244	rplV	50S ribosomal protein L22	7	0.02	8.7E-01	0	Ribosome
FTN_0856		thioredoxin	7	0.05	7.3E-01	0	
FTN_1028		UPF0082 protein FTN_1028	7	-0.14	1.9E-01	0	
FTN_1057	clpP	ATP-dependent Clp protease proteolytic subunit	7	0.14	2.2E-01	0	
FTN_1234	queA	S-adenosylmethionine:tRNA ribosyltransferase-isomerase	7	0.26	3.6E-02	0	
FTN_1672	nuoI	NADH-quinone oxidoreductase subunit I	7	0.31	4.1E-02	0	Oxidative phosphorylation;Metabolic pathways
FTN_1688		hypothetical protein	7	-0.01	9.7E-01	0	
FTN_0840	mdaB	NADPH-quinone reductase (modulator of drug activity B)	7	0.09	7.0E-01	0	
FTN_0752		hypothetical protein	7	-0.20	2.2E-01	0	
FTN_1097		isochorismatase hydrolase family protein	7	0.26	2.9E-01	0	
FTN_0110		peptide deformylase	7	0.15	2.7E-01	0	
FTN_0607	ftsI	cell division protein, peptidoglycan synthetase (PBP)	7	0.03	7.9E-01	0	Peptidoglycan biosynthesis;beta-Lactam resistance
FTN_1510	secB2	Protein-export protein secB 2	7	0.16	2.2E-01	0	Quorum sensing;Protein export;Bacterial secretion system
FTN_0728		Co/Zn/Cd cation transporter	7	0.11	4.3E-01	0	
FTN_1068		hypothetical protein	7	-0.01	9.7E-01	0	
FTN_0354	tolA	group A colicin translocation; tolA protein	7	0.61	2.1E-01	0	
FTN_0252	rpsN	30S ribosomal protein S14	7	-0.11	3.3E-01	0	Ribosome
FTN_0460		hypothetical protein	7	0.07	4.4E-01	0	
FTN_0099		hypothetical protein	7	-0.03	8.7E-01	0	
FTN_0104		phosphoheptose isomerase	7	0.41	2.8E-01	0	Lipopolysaccharide biosynthesis;Metabolic pathways
FTN_1613		U61 family peptidase	7	0.17	4.5E-01	0	
FTN_1247	lgt	Prolipoprotein diacylglyceryl transferase	7	-0.01	9.4E-01	0	
FTN_1749		acyltransferase	7	-0.31	5.5E-02	0	Glycerolipid metabolism;Glycerophospholipid metabolism;Metabolic pathways;Biosynthesis of secondary metabolites
FTN_0567		tRNA synthetase class II (D, K and N)	7	-0.29	2.8E-02	0	
FTN_0903		hypothetical protein	7	0.34	8.4E-04	0	
FTN_1294		rRNA methylase	7	0.27	1.9E-01	0	
FTN_1467	proC	pyrroline-5-carboxylate reductase	7	0.10	4.7E-01	0	Arginine and proline metabolism;Metabolic pathways;Biosynthesis of secondary metabolites;Biosynthesis of antibiotics;Biosynthesis of amino acids
FTN_0827		carbon-nitrogen hydrolase family protein	7	-0.07	6.0E-01	0	
FTN_0585	cutC	copper homeostasis protein CutC family protein	7	-0.86	4.7E-01	0	
FTN_0788		hypothetical protein	7	0.08	6.8E-01	0	
FTN_0231	uppS	undecaprenyl pyrophosphate synthase	7	0.42	9.9E-02	0	Terpenoid backbone biosynthesis;Biosynthesis of secondary metabolites
FTN_0502		ABC transporter, involved in lipoprotein release, permease component	7	-0.10	2.5E-01	0	ABC transporters
FTN_0909	lipB	Octanoyltransferase	7	0.12	3.5E-01	0	Lipoic acid metabolism;Metabolic pathways
FTN_1120	hemH	Ferrochelatase	7	0.61	1.6E-02	0	Porphyrin and chlorophyll metabolism;Metabolic pathways;Biosynthesis of secondary metabolites
FTN_1772		peptide methionine sulfoxide reductase	7	-0.97	2.7E-02	0	
FTN_0683	kdsB	3-deoxy-manno-octulosonate cytidylyltransferase	7	0.11	3.1E-01	0	Lipopolysaccharide biosynthesis;Metabolic pathways
FTN_0590		hypothetical protein	7	0.11	5.0E-01	0	
FTN_0182		ATP-binding cassette (ABC) superfamily protein	7	0.02	7.5E-01	0	
FTN_1245	iscS	cysteine desulfarase	7	0.41	1.1E-01	0	Thiamine metabolism;Metabolic pathways;Sulfur relay system
FTN_1750		acyltransferase	7	-0.02	9.1E-01	0	Glycerolipid metabolism;Glycerophospholipid metabolism;Metabolic pathways;Biosynthesis of secondary metabolites
FTN_1041	ilvN	acetolactate synthase small subunit	7	0.23	4.6E-01	0	Valine, leucine and isoleucine biosynthesis;Butanoate metabolism;C5-Branched dibasic acid metabolism;Pantothenate and CoA biosynthesis;Metabolic pathways;Biosynthesis of secondary metabolites;Biosynthesis of antibiotics;2-Oxocarboxylic acid metabolism;Biosynthesis of amino acids
FTN_0347	fkpB	FKBP-type peptidyl-prolyl cis-trans isomerase	7	-0.11	3.8E-01	0	
FTN_0500		peptide deformylase	7	-0.28	2.8E-01	0	
FTN_1619	appC	cytochrome bd-II terminal oxidase subunit I	7	-0.21	2.5E-01	0	Oxidative phosphorylation;Two-component system
FTN_0948	dnaB	replicative DNA helicase	7	-0.18	2.9E-01	0	DNA replication
FTN_0098	rsmG	Ribosomal RNA small subunit methyltransferase G	7	-0.36	2.6E-02	0	
FTN_1701		glutamate decarboxylase	7	0.03	8.9E-01	0	Alanine, aspartate and glutamate metabolism;beta-Alanine metabolism;Taurine and hypotaurine metabolism;Butanoate metabolism;Metabolic pathways;Biosynthesis of secondary metabolites;Microbial metabolism in diverse environments;Quorum sensing
FTN_1175		hypothetical protein	7	0.15	9.4E-02	0	
FTN_1116	pilC	Type IV pili polytopic inner membrane protein	7	0.25	1.2E-01	0	
FTN_0587		deoxyguanosinetriphosphate triphosphohydrolase	7	0.22	2.6E-01	0	
FTN_0293	lolA	Outer-membrane lipoprotein carrier protein	7	-0.12	3.4E-01	0	
FTN_1624		hypothetical protein	7	0.10	4.4E-01	0	
FTN_1774		hypothetical protein	7	0.23	1.3E-01	0	

FTN_1408		Mg-dependent DNase	7	0.15	1.2E-01	0	
FTN_1456	cfa	cyclopropane fatty acid synthase, methyltransferase	7	0.32	6.3E-03	0	
FTN_0259	secY	preprotein translocase subunit SecY	7	-0.59	3.5E-02	0	Quorum sensing;Protein export;Bacterial secretion system
FTN_1334		hypothetical protein	7	-0.34	2.7E-02	0	
FTN_1127	galM	aldose 1-epimerase	7	-0.15	3.5E-01	0	Glycolysis / Gluconeogenesis;Galactose metabolism;Metabolic pathways;Biosynthesis of secondary metabolites;Microbial metabolism in diverse environments;Biosynthesis of antibiotics
FTN_1114	glpQ	glycerophosphoryl diester phosphodiesterase	7	0.04	6.4E-01	0	Glycerophospholipid metabolism
FTN_1174	murI	glutamate racemase	7	0.05	6.5E-01	0	D-Glutamine and D-glutamate metabolism;Metabolic pathways
FTN_0901		fumarylacetoacetate hydrolase family protein	7	0.28	6.4E-01	0	
FTN_0989	yqhD	Fe-dependent alcohol dehydrogenase	7	0.10	5.6E-01	0	
FTN_0691	gmk	guanylate kinase	7	-0.36	4.1E-01	0	Purine metabolism;Metabolic pathways
FTN_1472		hypothetical protein	7	-0.21	4.3E-02	0	Oxidative phosphorylation;RNA degradation
FTN_0615		hypothetical protein	7	-0.46	1.1E-03	0	
FTN_0698		glutathione peroxidase	7	-0.22	2.3E-02	0	Glutathione metabolism;Arachidonic acid metabolism;Metabolic pathways
FTN_0140		ABC-type anion transport system, duplicated permease component	7	-0.05	7.5E-01	0	
FTN_0421	purN	phosphoribosylglycinamide formyltransferase	7	0.02	8.6E-01	0	Purine metabolism;One carbon pool by folate;Metabolic pathways;Biosynthesis of secondary metabolites;Biosynthesis of antibiotics
FTN_1621		NAD/FAD-dependent oxidoreductase	7	-0.08	4.9E-01	0	
FTN_1423	wbtG	glycosyl transferase, group 1	7	0.28	3.5E-01	0	
FTN_1066		HlyC/CorC family transporter-associated protein	7	0.00	9.9E-01	0	
FTN_0066	feoB	ferrous iron transport protein B	7	-0.06	6.4E-01	0	
FTN_0080		SAM-dependent methyltransferase	7	0.71	2.8E-01	0	
FTN_1092	rpoH	RNA polymerase factor sigma-32	7	0.02	9.1E-01	0	
FTN_1101		hypothetical protein	8	-0.28	4.1E-01	0	
FTN_0246	rplP	50S ribosomal protein L16	8	-0.09	4.9E-01	0	Ribosome
FTN_1383		carbon-nitrogen hydrolase	8	0.06	6.2E-01	0	Alanine, aspartate and glutamate metabolism;Metabolic pathways
FTN_0261	rpsM	30S ribosomal protein S13	8	-0.21	7.2E-02	0	Ribosome
FTN_1462	truB	tRNA pseudouridine synthase B	8	-0.54	5.2E-03	0	
FTN_0238	rpsJ	30S ribosomal protein S10	8	-0.02	9.0E-01	0	Ribosome
FTN_1031	ftnA	ferric iron binding protein, ferritin-like	8	-0.09	5.5E-01	0	
FTN_1288	rplM	50S ribosomal protein L13	8	0.00	9.7E-01	0	Ribosome
FTN_1233		haloacid dehalogenase-like hydrolase	8	-0.20	8.2E-01	0	
FTN_1113		hypothetical protein	8	0.15	1.4E-01	0	
FTN_0120		rhodanese-related sulfurtransferase	8	-0.07	3.8E-01	0	Cysteine and methionine metabolism;Sulfur metabolism;Metabolic pathways;Microbial metabolism in diverse environments;Sulfur relay system
FTN_1302	cysK	cysteine synthase	8	0.40	8.8E-02	0	Glycine, serine and threonine metabolism;Cysteine and methionine metabolism;Metabolic pathways;Biosynthesis of antibiotics;Biosynthesis of amino acids
FTN_0965		metal-dependent exopeptidase	8	-0.44	5.3E-02	0	
FTN_0915		hypothetical protein	8	-0.05	7.8E-01	0	
FTN_0171	glsA	glutaminase	8	0.06	7.1E-01	0	Arginine biosynthesis;Alanine, aspartate and glutamate metabolism;D-Glutamine and D-glutamate metabolism;Metabolic pathways;Two-component system
FTN_0555	ansB	periplasmic L-asparaginase II precursor	8	-0.31	1.8E-01	0	Alanine, aspartate and glutamate metabolism;Cyanoamino acid metabolism;Metabolic pathways;Biosynthesis of secondary metabolites
FTN_1093		hypothetical protein	8	0.12	3.3E-01	0	
FTN_0222		hypothetical protein	8	-0.12	5.9E-01	0	
FTN_0831		ATP-dependent RNA helicase	8	0.05	6.2E-01	0	RNA degradation
FTN_1069		hypothetical protein	8	-0.29	1.7E-01	0	
FTN_0846	infA	Translation initiation factor IF-1	8	0.05	6.8E-01	0	
FTN_1054	hupB	DNA-binding protein HU-beta	8	-0.23	2.3E-01	0	
FTN_0890		hypothetical protein	8	0.32	6.6E-02	0	
FTN_1185	rpiA	Ribose-5-phosphate isomerase A	8	-0.18	4.7E-01	0	Pentose phosphate pathway;Metabolic pathways;Biosynthesis of secondary metabolites;Microbial metabolism in diverse environments;Biosynthesis of antibiotics;Carbon metabolism;Biosynthesis of amino acids
FTN_1662	rimP	Ribosome maturation factor rimP	8	0.03	7.9E-01	0	
FTN_0482		hypothetical protein	8	-0.39	6.9E-02	0	
FTN_0748	pheA	prephenate dehydratase	8	0.53	5.6E-03	0	Phenylalanine, tyrosine and tryptophan biosynthesis;Metabolic pathways;Biosynthesis of secondary metabolites;Biosynthesis of antibiotics;Biosynthesis of amino acids
FTN_1045	engB	Probable GTP-binding protein EngB	8	0.37	1.4E-01	0	
FTN_0684		hypothetical protein	8	0.56	3.8E-03	0	
FTN_0055	tyrA	prephenate dehydrogenase	8	0.03	7.8E-01	0	Phenylalanine, tyrosine and tryptophan biosynthesis;Novobiocin biosynthesis;Metabolic pathways;Biosynthesis of secondary metabolites;Biosynthesis of antibiotics;Biosynthesis of amino acids
FTN_0273	folC	folylpoly-gamma-glutamate synthetase/ dihydrofolate synthetase	8	0.12	6.9E-01	0	Folate biosynthesis;Metabolic pathways

FTN_1665		magnesium chelatase	8	0.05	7.1E-01	0	
FTN_1626	hemN	coproporphyrinogen III oxidase, anaerobic	8	0.36	3.9E-02	0	
FTN_0598		tRNA-dihydrouridine synthase A	8	0.24	3.5E-01	0	
FTN_0849	trkA	potassium transporter peripheral membrane component	8	-0.26	1.3E-01	0	
FTN_1141	pilM	hypothetical protein	8	0.54	9.1E-04	0	
FTN_0442	ribF	riboflavin biosynthesis protein RibF	8	0.31	2.9E-02	0	Riboflavin metabolism;Metabolic pathways;Biosynthesis of secondary metabolites
FTN_1417	manB	phosphomannomutase	8	-0.29	2.1E-03	0	Fructose and mannose metabolism;Amino sugar and nucleotide sugar metabolism;Metabolic pathways;Biosynthesis of secondary metabolites;Biosynthesis of antibiotics
FTN_0641	cydC	cysteine/glutathione ABC transporter membrane/ATP-binding component	8	0.25	2.5E-01	0	ABC transporters
FTN_0560	rsmA	Ribosomal RNA small subunit methyltransferase A	8	-0.16	3.3E-01	0	
FTN_1020		hypothetical protein	8	0.28	1.0E-01	0	
FTN_0700		hypothetical protein	8	0.68	2.5E-01	0	
FTN_0077		hypothetical protein	8	0.39	5.9E-03	0	
FTN_1414	ppx	exopolyphosphatase	8	-0.59	1.3E-05	0	Purine metabolism;Metabolic pathways
FTN_0822		para-aminobenzoate synthase component I	8	0.75	8.5E-02	0	Folate biosynthesis
FTN_1734		hypothetical protein	9	0.02	8.4E-01	0	
FTN_0119		outer membrane protein of unknown function	9	0.10	4.1E-01	0	
FTN_0871		rare lipoprotein B family protein	9	-0.08	5.0E-01	0	
FTN_0059	leuB	3-isopropylmalate dehydrogenase	9	-0.07	5.0E-01	0	Valine, leucine and isoleucine biosynthesis;C5-Branched dibasic acid metabolism;Metabolic pathways;Biosynthesis of secondary metabolites;2-Oxocarboxylic acid metabolism;Biosynthesis of amino acids
FTN_0807		short chain dehydrogenase	9	0.11	4.4E-01	0	
FTN_0790	rdgC	recombination associated protein	9	-0.24	1.1E-02	0	
FTN_0477		hypothetical protein	9	0.11	5.0E-01	0	
FTN_0597		protein-disulfide isomerase	9	0.19	4.4E-02	0	
FTN_0602	pdxT	Glutamine amidotransferase subunit pdxT	9	-0.03	7.2E-01	0	Vitamin B6 metabolism
FTN_1565	gcp	Probable tRNA threonylcarbamoyladenosine biosynthesis protein Gcp	9	0.07	6.7E-01	0	
FTN_0695	add	deoxyadenosine deaminase/adenosine deaminase	9	-0.63	2.2E-02	0	Purine metabolism;Metabolic pathways
FTN_0149		hypothetical protein	9	-0.17	9.5E-02	0	
FTN_0331	minC	septum site-determining protein MinC	9	-0.10	3.9E-01	0	
FTN_0906	ppa	inorganic pyrophosphatase	9	0.14	5.7E-01	0	Oxidative phosphorylation
FTN_1447		hypothetical protein	9	-0.27	3.8E-03	0	
FTN_0572	potF	ATP-binding cassette putrescine uptake system, periplasmic protein	9	-0.03	7.7E-01	0	ABC transporters
FTN_1355		pantothenate kinase	9	0.11	2.6E-01	0	Pantothenate and CoA biosynthesis;Metabolic pathways
FTN_1370	gloB	Hydroxyacylglutathione hydrolase	9	0.14	1.6E-01	0	Pyruvate metabolism;Metabolic pathways
FTN_1051	hfq	Protein hfq	9	-0.01	8.7E-01	0	Quorum sensing;RNA degradation
FTN_0953	hemF	Coproporphyrinogen-III oxidase, aerobic	9	0.07	4.6E-01	0	Porphyrin and chlorophyll metabolism;Metabolic pathways;Biosynthesis of secondary metabolites
FTN_0125	ackA	propionate kinase 2 / acetate kinase A	9	0.16	4.5E-01	0	Taurine and hypotaurine metabolism;Pyruvate metabolism;Propanoate metabolism;Methane metabolism;Metabolic pathways;Microbial metabolism in diverse environments;Carbon metabolism
FTN_1639	sdhC	succinate dehydrogenase, cytochrome b556	9	0.07	6.6E-01	0	Citrate cycle (TCA cycle);Oxidative phosphorylation;Butanoate metabolism;Metabolic pathways;Biosynthesis of secondary metabolites;Microbial metabolism in diverse environments;Biosynthesis of antibiotics;Carbon metabolism
FTN_1692		membrane fusion protein	9	-0.47	4.4E-02	0	
FTN_0032		hypothetical protein	9	0.18	3.6E-01	0	
FTN_0390	htpX	heat shock protein HtpX	9	0.14	4.3E-01	0	
FTN_1088	ansA	L-asparaginase	9	0.57	2.9E-03	0	Alanine, aspartate and glutamate metabolism;Cyanoamino acid metabolism;Metabolic pathways;Biosynthesis of secondary metabolites
FTN_0685	galK	galactokinase	9	0.43	3.8E-03	0	Galactose metabolism;Amino sugar and nucleotide sugar metabolism;Metabolic pathways
FTN_1713		hypothetical protein	9	-0.08	5.8E-01	0	
FTN_1371		hypothetical protein	9	0.07	6.3E-01	0	
FTN_0210		hypothetical protein	9	0.20	2.3E-01	0	
FTN_0204		methanol dehydrogenase regulatory protein	9	0.06	5.6E-01	0	
FTN_0134		carboxylesterase/phospholipase family protein	9	-0.13	1.4E-01	0	
FTN_1617		two-component regulator, sensor histidine kinase	9	-0.14	2.3E-01	0	Two-component system;Quorum sensing
FTN_0035	pyrF	orotidine-5'-phosphate decarboxylase	9	-0.50	2.1E-03	0	Pyrimidine metabolism;Metabolic pathways
FTN_1252		choloylglycine hydrolase family protein	9	0.35	1.1E-02	0	Secondary bile acid biosynthesis;Metabolic pathways
FTN_0397	gpsA	Glycerol-3-phosphate dehydrogenase [NAD(P)+]	9	-0.14	1.2E-01	0	Glycerophospholipid metabolism;Biosynthesis of secondary metabolites
FTN_1315		hypothetical protein	9	0.31	2.8E-01	0	
FTN_0779		hypothetical protein	9	-0.02	9.2E-01	0	
FTN_1422	wbtN	glycosyl transferase, group 1	9	0.38	5.5E-03	0	
FTN_0226	folA	dihydrofolate reductase type I	9	0.00	9.9E-01	0	One carbon pool by folate;Folate biosynthesis;Metabolic pathways
FTN_0853	sufD	sufS activator complex, sufD subunit	9	0.01	9.3E-01	0	

FTN_0108	mnmA	tRNA-specific 2-thiouridylase mnmA	9	0.23	9.4E-03	0	Sulfur relay system
FTN_1454		NAD/FAD-binding protein	9	0.55	3.3E-02	0	
FTN_1216		hypothetical protein	9	-0.30	1.0E-01	0	
FTN_1255		glycosyl transferase family protein	9	0.06	8.7E-01	0	
FTN_0995	hslV	ATP-dependent protease peptidase subunit	10	0.05	7.5E-01	0	
FTN_1572	rplK	50S ribosomal protein L11	10	-0.11	3.7E-01	0	Ribosome
FTN_0427		lipoprotein of unknown function	10	0.53	1.9E-01	0	
FTN_1704	pcm	protein-L-isoaspartate O-methyltransferase	10	-0.18	3.0E-01	0	
FTN_1324	iglA	intracellular growth locus protein A	10	0.04	8.2E-01	0	
FTN_1476		hypothetical protein	10	-0.06	4.3E-01	0	
FTN_0559		peptidyl-prolyl cis-trans isomerase (PPIase)	10	0.01	9.2E-01	0	
FTN_1142	adk	Adenylate kinase	10	-0.12	4.8E-01	0	Purine metabolism;Thiamine metabolism;Metabolic pathways;Biosynthesis of secondary metabolites;Biosynthesis of antibiotics
FTN_1645	atpC	ATP synthase epsilon chain	10	0.02	7.7E-01	0	Oxidative phosphorylation;Metabolic pathways
FTN_0245	rpsC	30S ribosomal protein S3	10	-0.17	3.3E-01	0	Ribosome
FTN_1767	rbsK	ribokinase	10	-0.55	5.4E-02	0	Pentose phosphate pathway;Metabolic pathways
FTN_0402	aroC	Chorismate synthase	10	-0.07	5.3E-01	0	Phenylalanine, tyrosine and tryptophan biosynthesis;Metabolic pathways;Biosynthesis of secondary metabolites;Biosynthesis of antibiotics;Biosynthesis of amino acids
FTN_0693	nadC	nicotinate-nucleotide pyrophosphorylase	10	0.21	7.0E-02	0	Nicotinate and nicotinamide metabolism;Metabolic pathways
FTN_1208	ans	asparaginase	10	0.01	9.7E-01	0	Alanine, aspartate and glutamate metabolism;Cyanoamino acid metabolism;Metabolic pathways;Biosynthesis of secondary metabolites
FTN_1539	groS	10 kDa chaperonin	10	0.21	1.5E-01	0	
FTN_1676	nuoE	NADH dehydrogenase I, E subunit	10	0.04	6.7E-01	0	Oxidative phosphorylation;Metabolic pathways
FTN_1415		thioredoxin	10	0.02	7.9E-01	0	
FTN_0167	prfB	peptide chain release factor 2	10	-0.08	5.8E-01	0	
FTN_0745		hypothetical protein	10	0.08	3.6E-01	0	
FTN_0522	murF	UDP-N--acetylmuramoylalanyl-D-glutamyl-2,6- diaminopimelate-D-alanyl-D-alanyl ligase	10	0.08	2.4E-01	0	Lysine biosynthesis;Peptidoglycan biosynthesis;Metabolic pathways;Vancomycin resistance
FTN_1048	hflK	HflK-HflC membrane protein complex, HflK	10	-0.41	1.6E-02	0	
FTN_1745	purT	Phosphoribosylglycinamide formyltransferase 2	10	-0.11	1.8E-01	0	Purine metabolism;One carbon pool by folate;Metabolic pathways;Biosynthesis of secondary metabolites;Biosynthesis of antibiotics
FTN_1643		glutaredoxin-related protein	10	0.05	7.2E-01	0	
FTN_1001		hypothetical protein	10	-0.10	2.2E-01	0	
FTN_1445		hypothetical protein	10	-0.26	1.7E-01	0	
FTN_1283	dnaJ	chaperone protein DnaJ	10	0.19	3.3E-01	0	
FTN_1296		sigma54 modulation protein	10	0.19	1.8E-01	0	
FTN_1469	kdtA	3-deoxy-D-manno-octulosonic-acid transferase	10	-0.05	6.2E-01	0	Lipopolysaccharide biosynthesis;Metabolic pathways
FTN_0179	hpt	hypoxanthine-guanine phosphoribosyltransferase	10	-0.57	2.8E-05	0	Purine metabolism;Metabolic pathways;Biosynthesis of secondary metabolites
FTN_0132		hypothetical protein	10	0.15	7.9E-02	0	
FTN_1482		outer membrane protein of unknown function	10	-0.02	8.5E-01	0	
FTN_0974		haloacid dehalogenase-like hydrolase	10	0.32	1.8E-02	0	
FTN_0656		zinc-dependent peptidase	10	-0.55	6.2E-03	0	
FTN_0060	leuD	3-isopropylmalate dehydratase small subunit	10	-0.13	3.6E-01	0	Valine, leucine and isoleucine biosynthesis;C5-Branched dibasic acid metabolism;Metabolic pathways;Biosynthesis of secondary metabolites;2-Oxocarboxylic acid metabolism;Biosynthesis of amino acids
FTN_0927	cysN	Sulfate adenylyltransferase subunit 1	10	-0.10	5.8E-01	0	Purine metabolism;Monobactam biosynthesis;Selenocompound metabolism;Sulfur metabolism;Metabolic pathways;Microbial metabolism in diverse environments;Biosynthesis of antibiotics
FTN_1050	hflX	protease, GTP-binding subunit	10	-0.26	1.4E-01	0	
FTN_1190	infC	translation initiation factor IF-3	10	-0.41	1.1E-02	0	
FTN_0540	pckA	phosphoenolpyruvate carboxykinase	10	0.58	1.6E-02	0	Glycolysis / Gluconeogenesis;Citrate cycle (TCA cycle);Pyruvate metabolism;Metabolic pathways;Biosynthesis of secondary metabolites;Microbial metabolism in diverse environments;Biosynthesis of antibiotics;Carbon metabolism
FTN_0518	malQ	4-alpha-glucanotransferase	10	0.42	1.2E-02	0	Starch and sucrose metabolism;Metabolic pathways;Biosynthesis of secondary metabolites
FTN_1595	ftsY	cell division protein, signal recognition particle GTPase	10	-0.34	1.8E-03	0	Quorum sensing;Protein export;Bacterial secretion system
FTN_0852	sufC	sufS activator complex, sufC subunit	10	-0.07	7.3E-01	0	
FTN_1263	comL	competence lipoprotein ComL	10	-0.27	3.2E-02	0	
FTN_0946	pilF	Type IV pili, pilus assembly protein	10	0.12	1.4E-01	0	
FTN_1564		fatty acid desaturase	10	0.63	1.4E-02	0	Biosynthesis of unsaturated fatty acids;Metabolic pathways;Fatty acid metabolism
FTN_0975		hypothetical protein	10	-0.28	1.7E-01	0	
FTN_1222	kpsF	phosphosugar isomerase	10	0.20	2.3E-01	0	Lipopolysaccharide biosynthesis;Metabolic pathways
FTN_0751	sufS	selenocysteine lyase	10	-0.36	3.7E-03	0	Selenocompound metabolism;Metabolic pathways
FTN_0536	yjjK	drug resistance ATPase-1 family protein	10	0.33	1.9E-02	0	

FTN_1546	hemA	Glutamyl-tRNA reductase	10	0.71	1.0E-02	0	Porphyrin and chlorophyll metabolism;Metabolic pathways;Biosynthesis of secondary metabolites;Microbial metabolism in diverse environments
FTN_0490	map	methionine aminopeptidase	10	-0.27	8.9E-03	0	
FTN_1253	lpcC	glycosyl transferase, group 1	10	0.50	1.6E-02	0	
FTN_0959	oxyR	oxidative stress transcriptional regulator	10	-0.07	7.0E-01	0	
FTN_1615		hypothetical protein	10	0.46	4.6E-02	0	
FTN_1589	oppF	peptide/opine/nickel uptake transporter (PepT) family protein	10	-0.07	7.5E-01	0	beta-Lactam resistance;ABC transporters;Quorum sensing
FTN_0512	glgX	pullulanase	10	-0.03	8.5E-01	0	Starch and sucrose metabolism;Metabolic pathways;Biosynthesis of secondary metabolites
FTN_0275		hypothetical protein	11	-0.25	1.2E-01	0	
FTN_1496	coaE	dephospho-CoA kinase	11	-0.14	4.3E-01	0	Pantothenate and CoA biosynthesis;Metabolic pathways
FTN_1756	bcp	bacterioferritin comigratory protein	11	0.10	4.3E-01	0	
FTN_1029		isoprenoid biosynthesis protein with amidotransferase-like domain	11	-0.02	7.8E-01	0	
FTN_0387	rph	Ribonuclease PH	11	-0.27	1.4E-02	0	
FTN_1573	nusG	transcription antitermination protein nusG	11	-0.13	1.4E-01	0	
FTN_1276		membrane fusion protein	11	-0.25	1.2E-01	0	
FTN_0812	bioD	ATP-dependent dethiobiotin synthetase BioD	11	0.01	9.3E-01	0	Biotin metabolism;Metabolic pathways
FTN_0230	frr	Ribosome-recycling factor	11	-0.04	7.2E-01	0	
FTN_1033	grxB	glutaredoxin 2	11	-0.10	4.5E-01	0	
FTN_0957		short chain dehydrogenase	11	0.25	5.5E-02	0	
FTN_0433	parA	chmomosome partition protein A	11	-0.25	5.7E-02	0	
FTN_0765		choloylglycine hydrolase family protein	11	-0.01	8.9E-01	0	Secondary bile acid biosynthesis;Metabolic pathways
FTN_0111	ribH	riboflavin synthase beta-chain	11	-0.58	4.3E-02	0	Riboflavin metabolism;Metabolic pathways;Biosynthesis of secondary metabolites
FTN_0236	rpsG	30S ribosomal protein S7	11	-0.09	4.2E-01	0	Ribosome
FTN_1009		hypothetical protein	11	0.03	8.6E-01	0	
FTN_1277		outer membrane efflux protein	11	-0.02	9.2E-01	0	
FTN_0078	aroE	shikimate 5-dehydrogenase	11	0.07	6.3E-01	0	Phenylalanine, tyrosine and tryptophan biosynthesis;Metabolic pathways;Biosynthesis of secondary metabolites;Biosynthesis of antibiotics;Biosynthesis of amino acids
FTN_0436		hypothetical protein	11	0.07	4.3E-01	0	
FTN_0127	gabD	succinate semialdehyde dehydrogenase (NAD(P)+ dependent)	11	-0.08	5.7E-01	0	Alanine, aspartate and glutamate metabolism;Lysine degradation;Tyrosine metabolism;Butanoate metabolism;Nicotinate and nicotinamide metabolism;Metabolic pathways;Microbial metabolism in diverse environments
FTN_1542		UPF0246 protein FTN_1542	11	0.08	5.5E-01	0	
FTN_0646	cscK	ROK family protein	11	0.04	6.9E-01	0	Fructose and mannose metabolism;Starch and sucrose metabolism;Amino sugar and nucleotide sugar metabolism;Metabolic pathways
FTN_0612	udk	uridine kinase	11	-0.07	5.0E-01	0	Pyrimidine metabolism;Metabolic pathways
FTN_1163		hypothetical protein	11	0.33	1.3E-03	0	
FTN_1535		short chain dehydrogenase	11	0.05	6.7E-01	0	
FTN_0225		hypothetical protein	11	0.53	1.1E-02	0	
FTN_0325		membrane protein of unknown function	11	0.00	9.7E-01	0	ABC transporters
FTN_0873	dcd	Deoxycytidine triphosphate deaminase	11	0.26	6.8E-03	0	Pyrimidine metabolism;Metabolic pathways
FTN_1618		hypothetical protein	11	0.21	3.8E-01	0	
FTN_1298	mnmE	tRNA modification GTPase MnmE	11	-0.13	1.1E-01	0	
FTN_0133		ribonuclease II family protein	11	0.43	4.3E-02	0	
FTN_1238		hypothetical protein	11	0.04	6.7E-01	0	
FTN_0524	asd	aspartate-semialdehyde dehydrogenase	11	-0.08	3.8E-01	0	Glycine, serine and threonine metabolism;Monobactam biosynthesis;Cysteine and methionine metabolism;Lysine biosynthesis;Metabolic pathways;Biosynthesis of secondary metabolites;Microbial metabolism in diverse environments;Biosynthesis of antibiotics;2-Oxocarboxylic acid metabolism;Biosynthesis of amino acids
FTN_0769		bifunctional methionine sulfoxide reductase B/A protein	11	-0.26	2.5E-02	0	
FTN_0993	lplA	lipoate-protein ligase A	11	0.04	7.4E-01	0	Lipoic acid metabolism;Metabolic pathways
FTN_0629	purU	formyltetrahydrofolate deformylase	11	-0.37	2.8E-03	0	Glyoxylate and dicarboxylate metabolism;One carbon pool by folate
FTN_0072		LPS fatty acid acyltransferase	11	0.13	4.3E-01	0	Lipopolysaccharide biosynthesis;Metabolic pathways
FTN_0094		LysR family transcriptional regulator	11	-0.13	3.6E-01	0	
FTN_1590	oppD	peptide/opine/nickel uptake transporter (PepT) family protein	11	0.20	1.7E-01	0	beta-Lactam resistance;ABC transporters;Quorum sensing
FTN_1642	sodB	iron/manganese superoxide dismutase family protein	12	0.03	7.5E-01	0	
FTN_0249	rplN	50S ribosomal protein L14	12	-0.08	4.7E-01	0	Ribosome
FTN_0240	rplD	50S ribosomal protein L4	12	-0.02	8.9E-01	0	Ribosome
FTN_1557		oxidoreductase iron/ascorbate family protein	12	-0.11	1.2E-01	0	
FTN_0665	greA	Transcription elongation factor greA	12	0.06	3.8E-01	0	
FTN_1480	lpxD	UDP-3-O-[3-hydroxymyristoyl] glucosamine N-acyltransferase	12	-0.24	1.4E-01	0	Lipopolysaccharide biosynthesis;Metabolic pathways
FTN_1021		hypothetical protein	12	0.02	8.3E-01	0	
FTN_1285	grpE	Protein grpE	12	-0.03	9.0E-01	0	
FTN_1522		subunit of DnaJ/DnaK/GrpE: chaperone with DnaK; heat shock protein	12	-0.16	5.9E-01	0	
FTN_0434	parB	chromosome partition protein B	12	0.01	9.7E-01	0	

FTN_0874		nucleotide-binding protein	12	0.38	6.3E-04	0	
FTN_0565		hypothetical protein	12	0.01	9.3E-01	0	
FTN_0033		chorismate mutase	12	-0.07	7.2E-01	0	
FTN_0121	secB1	Protein-export protein secB 1	12	-0.43	2.1E-02	0	Quorum sensing;Protein export;Bacterial secretion system
FTN_1047	hflC	HflK-HflC membrane protein complex, HflC	12	-0.01	9.4E-01	0	
FTN_0461	ubiE	Ubiquinone/menaquinone biosynthesis methyltransferase ubiE	12	0.03	7.7E-01	0	Ubiquinone and other terpenoid-quinone biosynthesis;Metabolic pathways;Biosynthesis of secondary metabolites
FTN_0191		polar amino acid uptake transporter	12	0.06	5.5E-01	0	Phenylalanine, tyrosine and tryptophan biosynthesis;Metabolic pathways;Biosynthesis of secondary metabolites;Biosynthesis of antibiotics;Biosynthesis of amino acids
FTN_1478	lpxA	UDP-N-acetylglucosamine acyltransferase	12	-0.06	5.8E-01	0	Lipopolysaccharide biosynthesis;Metabolic pathways;Cationic antimicrobial peptide (CAMP) resistance
FTN_1757		D-isomer specific 2-hydroxyacid dehydrogenase	12	-0.42	2.3E-03	0	
FTN_0265	rplQ	50S ribosomal protein L17	12	-0.10	4.2E-01	0	Ribosome
FTN_1548		hypothetical protein	12	0.21	2.4E-02	0	
FTN_0878		hypothetical protein	12	0.80	1.7E-01	0	
FTN_0530	mpl	UDP-N-acetylmuramate:L-alanyl-gamma-D-glutamyl- meso-diaminopimelate ligase	12	-0.48	1.6E-03	0	
FTN_1169		M20 family peptidase	12	-0.61	1.5E-01	0	
FTN_0493	mtn	5'-methylthioadenosine/S-adenosylhomocysteine nucleosidase	12	0.01	9.1E-01	0	Ubiquinone and other terpenoid-quinone biosynthesis;Cysteine and methionine metabolism;Metabolic pathways;Biosynthesis of secondary metabolites;Biosynthesis of amino acids
FTN_1782	rng	ribonuclease G	12	-0.19	3.6E-01	0	
FTN_0692	nadA	quinolinate synthetase	12	0.01	8.8E-01	0	Nicotinate and nicotinamide metabolism;Metabolic pathways
FTN_1107	metIQ	methionine uptake transporter (MUT) family protein, membrane and periplasmic protein	12	0.27	5.1E-03	0	ABC transporters
FTN_1128	dfp	P-pantothenate cysteine ligase/P-pantothenoylcysteine decarboxylase	12	-0.07	6.1E-01	0	Pantothenate and CoA biosynthesis;Metabolic pathways
FTN_0947	rlmN	Ribosomal RNA large subunit methyltransferase N	12	0.03	8.1E-01	0	
FTN_0381		hypothetical protein	12	1.90	1.1E-01	0	
FTN_0635		serine-type D-Ala-D-Ala carboxypeptidase	12	-0.20	2.2E-01	0	Peptidoglycan biosynthesis
FTN_1431	wbtA	dTDP-glucose 4,6-dehydratase	12	-0.39	5.3E-02	0	
FTN_0628	upp	Uracil phosphoribosyltransferase	12	0.39	2.9E-03	0	Pyrimidine metabolism;Metabolic pathways
FTN_1470	ispA	geranyl diphosphate synthase/farnesyl diphosphate synthase	12	0.26	2.6E-02	0	Terpenoid backbone biosynthesis;Metabolic pathways;Biosynthesis of secondary metabolites;Biosynthesis of antibiotics
FTN_1149	nagA	N-acetylglucosamine-6-phosphate deacetylase	12	-0.33	5.7E-03	0	Amino sugar and nucleotide sugar metabolism;Metabolic pathways;Biosynthesis of antibiotics
FTN_0509		hypothetical protein	12	0.06	7.1E-01	0	
FTN_0729	galU	UTP--glucose-1-phosphate uridylyltransferase	12	-0.10	3.8E-01	0	Pentose and glucuronate interconversions;Galactose metabolism;Starch and sucrose metabolism;Amino sugar and nucleotide sugar metabolism;Metabolic pathways;Biosynthesis of antibiotics
FTN_0966		hypothetical protein	12	-0.13	3.1E-01	0	
FTN_0071		LPS fatty acid acyltransferase	12	-0.25	2.6E-01	0	Lipopolysaccharide biosynthesis;Metabolic pathways
FTN_0529	pyrE	orotate phosphoribosyltransferase	12	-0.07	6.1E-01	0	Pyrimidine metabolism;Metabolic pathways
FTN_1145	era	GTP-binding protein Era	12	0.04	6.9E-01	0	
FTN_1778	trpE	anthranilate synthase component I	12	0.37	5.0E-02	0	Phenylalanine, tyrosine and tryptophan biosynthesis;Metabolic pathways;Biosynthesis of secondary metabolites;Biosynthesis of antibiotics;Biosynthesis of amino acids;Quorum sensing
FTN_1170		hypothetical protein	12	-0.31	6.8E-03	0	
FTN_0990		4Fe-4S ferredoxin, FAD dependent	12	0.08	6.5E-01	0	
FTN_0575		hypothetical protein	13	-0.17	2.8E-01	0	
FTN_0211	pcp	pyrrolidone carboxylylate peptidase	13	-0.20	3.0E-01	0	
FTN_0921		FKBP-type peptidyl-prolyl cis-trans isomerase	13	0.14	2.6E-01	0	
FTN_0239	rplC	50S ribosomal protein L3	13	0.02	8.4E-01	0	Ribosome
FTN_0558	lptD	LPS-assembly protein lptD	13	-0.02	8.7E-01	0	
FTN_0091		hypothetical protein	13	0.03	8.3E-01	0	
FTN_1603		pantothenate kinase	13	-0.03	6.9E-01	0	Pantothenate and CoA biosynthesis;Metabolic pathways
FTN_0471		NADPH-dependent FMN reductase	13	0.34	2.0E-01	0	
FTN_1390		Zn-dependent hydrolase	13	0.20	7.9E-02	0	
FTN_1209	cphB	cyanophycinase	13	-0.07	5.4E-01	0	
FTN_1740	trpA	tryptophan synthase subunit alpha	13	0.10	3.9E-01	0	Glycine, serine and threonine metabolism;Phenylalanine, tyrosine and tryptophan biosynthesis;Metabolic pathways;Biosynthesis of secondary metabolites;Biosynthesis of antibiotics;Biosynthesis of amino acids
FTN_1136	aroK	Shikimate kinase	13	0.04	6.1E-01	0	Phenylalanine, tyrosine and tryptophan biosynthesis;Metabolic pathways;Biosynthesis of secondary metabolites;Biosynthesis of antibiotics;Biosynthesis of amino acids
FTN_1658	hisS	Histidine--tRNA ligase	13	0.06	6.7E-01	0	Aminoacyl-tRNA biosynthesis
FTN_1268		hypothetical protein	13	0.53	4.8E-05	0	ABC transporters
FTN_1177	sbcB	exodeoxyribonuclease I	13	0.01	8.9E-01	0	Mismatch repair

FTN_0422	purE	N5-carboxyaminoimidazole ribonucleotide mutase	13	0.10	4.1E-01	0	Purine metabolism;Metabolic pathways;Biosynthesis of secondary metabolites;Biosynthesis of antibiotics
FTN_0019	pyrB	aspartate carbamoyltransferase	13	0.33	6.4E-02	0	Pyrimidine metabolism
FTN_1405		ABC transporter ATP-binding protein	13	0.04	6.9E-01	0	
FTN_1137	pilQ	Type IV pili secretin component	13	-0.04	8.4E-01	0	
FTN_0109		hypothetical protein	13	0.06	5.8E-01	0	
FTN_0786		hypothetical protein	13	-0.13	3.2E-01	0	
FTN_1151		hypothetical protein	13	-0.57	1.1E-03	0	
FTN_0686	galT	galactose-1-phosphate uridylyltransferase	13	-0.08	3.6E-01	0	Galactose metabolism;Amino sugar and nucleotide sugar metabolism;Metabolic pathways
FTN_1474	bglX	glycosy hydrolase family protein	13	0.44	1.5E-01	0	
FTN_1199		hypothetical protein	13	-0.03	7.8E-01	0	
FTN_1100	tgt	Queuine tRNA-ribosyltransferase	13	-0.04	7.8E-01	0	
FTN_0001	dnaA	Chromosomal replication initiator protein DnaA	13	-0.06	5.6E-01	0	Two-component system
FTN_0447	murA	UDP-N-acetylglucosamine 1-carboxyvinyltransferase	13	-0.27	4.5E-02	0	Amino sugar and nucleotide sugar metabolism;Peptidoglycan biosynthesis;Metabolic pathways
FTN_0022		histidine acid phosphatase	13	0.37	5.4E-03	0	
FTN_0798	rlmL	Ribosomal RNA large subunit methyltransferase L	13	-0.20	5.8E-02	0	
FTN_0519	glyQ	Glycine--tRNA ligase alpha subunit	13	0.17	3.5E-02	0	Aminoacyl-tRNA biosynthesis
FTN_0103		hypothetical protein	13	0.11	6.2E-01	0	
FTN_1731	pip	proline iminopeptidase	13	0.44	3.5E-02	0	Arginine and proline metabolism;Metabolic pathways
FTN_0417	folD	Bifunctional protein FolD [Includes: Methylenetetrahydrofolate dehydrogenase ; Methenyltetrahydrofolate cyclohydrolase]	13	0.12	1.9E-01	0	One carbon pool by folate;Metabolic pathways;Microbial metabolism in diverse environments;Carbon metabolism
FTN_1360	gltB	glutamate synthase domain-containing 2	13	-0.04	6.9E-01	0	
FTN_0616	rlmD	23S rRNA (uracil(1939)-C(5))-methyltransferase RlmD	13	-0.18	2.6E-01	0	
FTN_1594	uvrD	DNA helicase II	13	-0.55	8.1E-03	0	Nucleotide excision repair;Mismatch repair
FTN_1018		aldolase/adducin class II family protein	13	0.29	3.9E-02	0	
FTN_0819		6-hydroxymethyl-7,8-dihydropterin pyrophosphokinase/dihydropteroate synthase	13	0.20	1.1E-01	0	Folate biosynthesis;Metabolic pathways
FTN_1443		putative nicotinate phosphoribosyltransferase	13	-0.48	6.4E-03	0	Nicotinate and nicotinamide metabolism;Metabolic pathways
FTN_0044		hypothetical protein	14	-0.08	6.0E-01	0	
FTN_0949	rplI	50S ribosomal protein L9	14	-0.03	8.4E-01	0	Ribosome
FTN_0841		ThiJ/PfpI family protein	14	0.07	6.1E-01	0	
FTN_0514	pgm	phosphoglucomutase	14	0.20	2.1E-01	0	Glycolysis / Gluconeogenesis;Pentose phosphate pathway;Galactose metabolism;Purine metabolism;Starch and sucrose metabolism;Amino sugar and nucleotide sugar metabolism;Streptomycin biosynthesis;Metabolic pathways;Biosynthesis of secondary metabolites;Microbial metabolism in diverse environments;Biosynthesis of antibiotics
FTN_1499	trpS	tryptophanyl-tRNA synthetase	14	0.05	5.8E-01	0	Aminoacyl-tRNA biosynthesis
FTN_0891	ruvB	Holliday junction ATP-dependent DNA helicase RuvB	14	0.28	5.7E-03	0	Homologous recombination
FTN_0391		LemA-like protein	14	0.07	6.2E-01	0	
FTN_0253	rpsH	30S ribosomal protein S8	14	-0.04	6.8E-01	0	Ribosome
FTN_1064		PhoH family protein, putative ATPase	14	-0.03	7.7E-01	0	
FTN_0618		ROK family protein	14	-0.14	1.3E-01	0	
FTN_0545		glycosyl transferase, group 2	14	-0.09	5.3E-01	0	
FTN_0945	rsuA	16S rRNA pseudouridine synthase	14	0.12	3.3E-01	0	
FTN_0409	adhC	Zn-dependent alcohol dehydrogenase	14	0.13	4.9E-01	0	Glycolysis / Gluconeogenesis;Fatty acid degradation;Tyrosine metabolism;Chloroalkane and chloroalkene degradation;Naphthalene degradation;Methane metabolism;Metabolic pathways;Biosynthesis of secondary metabolites;Microbial metabolism in diverse environments;Biosynthesis of antibiotics;Carbon metabolism;Degradation of aromatic compounds
FTN_0513	glgB	1,4-alpha-glucan branching enzyme GlgB	14	0.43	2.4E-02	0	Starch and sucrose metabolism;Metabolic pathways;Biosynthesis of secondary metabolites
FTN_1479	fabZ	(3R)-hydroxymyristoyl-[acyl-carrier-protein] dehydratase	14	-0.03	7.3E-01	0	Fatty acid biosynthesis;Biotin metabolism;Metabolic pathways;Fatty acid metabolism
FTN_0088		LamB/YcsF family protein	14	0.04	8.3E-01	0	
FTN_0826		aldo/keto reductase family protein	14	0.21	2.2E-02	0	
FTN_0818		lipase/esterase	14	0.24	3.2E-02	0	Biotin metabolism;Metabolic pathways
FTN_0459	ubiB	2-octaprenylphenol hydroxylase	14	-0.02	8.9E-01	0	
FTN_0393		hypothetical protein	14	-0.51	1.9E-03	0	
FTN_0200		UDP-3-O-[3-fatty acid] glucosamine N-acyltransferase	14	-0.24	1.7E-01	0	Lipopolysaccharide biosynthesis;Metabolic pathways
FTN_0914	dnaG	DNA primase	14	-0.39	3.2E-02	0	DNA replication
FTN_0588		asparaginase	14	-0.23	1.5E-01	0	Alanine, aspartate and glutamate metabolism;Cyanoamino acid metabolism;Metabolic pathways;Biosynthesis of secondary metabolites
FTN_0704		type I restriction-modification system, subunit M (methyltransferase)	14	-0.18	2.9E-01	0	
FTN_0549	sspA	stringent starvation protein A	14	-0.25	4.6E-02	0	
FTN_1321	iglD	intracellular growth locus protein D	14	0.49	1.3E-02	0	

FTN_0632		dGTP triphosphohydrolase	14	0.43	2.2E-02	0	Purine metabolism;Metabolic pathways
FTN_1350	ligA	DNA ligase	14	-0.01	9.5E-01	0	DNA replication;Base excision repair;Nucleotide excision repair;Mismatch repair
FTN_1039	mfd	transcription-repair coupling factor	14	0.52	7.1E-02	0	Nucleotide excision repair
FTN_0920	ttcA1	tRNA 2-thiocytidine biosynthesis protein TtcA 1	14	0.09	4.8E-01	0	
FTN_1313		hypothetical protein	14	-0.81	2.6E-02	0	
FTN_1339	fabG	3-oxoacyl-(acyl-carrier-protein) reductase	15	-0.25	4.0E-01	0	Fatty acid biosynthesis;Biotin metabolism;Metabolic pathways;Biosynthesis of antibiotics;Fatty acid metabolism
FTN_1569	rplL	50S ribosomal protein L7/L12	15	-0.01	9.4E-01	0	Ribosome
FTN_1631	tpiA	Triosephosphate isomerase	15	-0.37	1.6E-03	0	Glycolysis / Gluconeogenesis;Fructose and mannose metabolism;Inositol phosphate metabolism;Metabolic pathways;Biosynthesis of secondary metabolites;Microbial metabolism in diverse environments;Biosynthesis of antibiotics;Carbon metabolism;Biosynthesis of amino acids
FTN_0580	trxB	thioredoxin reductase	15	-0.26	1.2E-02	0	Selenocompound metabolism
FTN_1109		rhodanese-like family protein	15	0.11	4.7E-01	0	
FTN_0444		membrane protein of unknown function	15	0.04	7.2E-01	0	
FTN_0358	rimO	Ribosomal protein S12 methylthiotransferase RimO	15	-0.03	7.4E-01	0	
FTN_0183		periplasmic solute binding family protein	15	0.20	1.1E-02	0	
FTN_1380		aspartate/tyrosine/aromatic aminotransferase	15	0.28	3.2E-02	0	
FTN_0063	ilvE	branched-chain amino acid aminotransferase protein (class IV)	15	0.10	4.4E-01	0	Cysteine and methionine metabolism;Valine, leucine and isoleucine degradation;Valine, leucine and isoleucine biosynthesis;Pantothenate and CoA biosynthesis;Metabolic pathways;Biosynthesis of secondary metabolites;Biosynthesis of antibiotics;2-Oxocarboxylic acid metabolism;Biosynthesis of amino acids
FTN_0412	recN	DNA repair protein	15	-0.05	7.1E-01	0	
FTN_0720		IclR family transcriptional regulator	15	-0.03	7.4E-01	0	
FTN_1072		beta-lactamase class A	15	-0.07	7.3E-01	0	Biosynthesis of antibiotics;beta-Lactam resistance
FTN_1171		hypothetical protein	15	-0.02	8.4E-01	0	
FTN_0229	pyrH	Uridylate kinase	15	-0.27	4.8E-02	0	Pyrimidine metabolism;Metabolic pathways
FTN_1372		hypothetical protein	15	0.05	5.0E-01	0	
FTN_1633	apt	Adenine phosphoribosyltransferase	15	-0.16	1.2E-01	0	Purine metabolism;Metabolic pathways
FTN_1391	naoX	uncharacterized NAD(FAD)-dependent dehydrogenase	15	0.26	1.5E-01	0	
FTN_1030	lipA	Lipoyl synthase	15	0.12	1.9E-01	0	Lipoic acid metabolism;Metabolic pathways
FTN_0161	ddl	D-alanine--D-alanine ligase	15	-0.08	7.5E-01	0	D-Alanine metabolism;Peptidoglycan biosynthesis;Metabolic pathways;Vancomycin resistance
FTN_0611	kdsA	2-dehydro-3-deoxyphosphooctonate aldolase	15	0.36	1.0E-03	0	Lipopolysaccharide biosynthesis;Metabolic pathways
FTN_0804	gshB	glutathione synthetase	15	-0.12	2.5E-01	0	Cysteine and methionine metabolism;Glutathione metabolism;Metabolic pathways
FTN_0165	lpxC	UDP-3-O-[3-hydroxymyristoyl] N-acetylglucosamine deacetylase	15	-0.12	1.6E-01	0	Lipopolysaccharide biosynthesis;Metabolic pathways
FTN_0166	dnaX	DNA polymerase III, gamma/tau subunits	15	-0.05	6.6E-01	0	DNA replication;Mismatch repair;Homologous recombination
FTN_1135	aroB	3-dehydroquinate synthase	15	-0.01	9.5E-01	0	Phenylalanine, tyrosine and tryptophan biosynthesis;Metabolic pathways;Biosynthesis of secondary metabolites;Biosynthesis of antibiotics;Biosynthesis of amino acids
FTN_1158		methyltransferase	15	-0.09	7.1E-01	0	
FTN_1418	manC	mannose-1-phosphate guanylyltransferase	15	-0.05	4.8E-01	0	Fructose and mannose metabolism;Amino sugar and nucleotide sugar metabolism;Metabolic pathways;Biosynthesis of secondary metabolites
FTN_1491		adenine specific DNA methylase	15	0.25	5.0E-02	0	
FTN_0282		hypothetical protein	15	0.51	8.3E-04	0	
FTN_1693		ATP-binding cassette (ABC) superfamily protein	15	-0.05	5.9E-01	0	
FTN_0896	dxs	1-deoxy-D-xylulose-5-phosphate synthase	15	0.17	9.3E-02	0	Thiamine metabolism;Terpenoid backbone biosynthesis;Metabolic pathways;Biosynthesis of secondary metabolites;Biosynthesis of antibiotics
FTN_1464	lepB	signal peptidase I	15	-0.29	4.2E-02	0	Protein export
FTN_0563	accB	acetyl-CoA carboxylase, biotin carboxy carrier protein subunit	16	0.07	4.4E-01	0	Fatty acid biosynthesis;Pyruvate metabolism;Propanoate metabolism;Metabolic pathways;Biosynthesis of secondary metabolites;Microbial metabolism in diverse environments;Biosynthesis of antibiotics;Carbon metabolism;Fatty acid metabolism
FTN_1606	msbA	lipid exporter (LipidE) family protein	16	-0.01	9.1E-01	0	ABC transporters
FTN_0973		AhpC/TSA family peroxiredoxin	16	0.07	5.1E-01	0	
FTN_1322	iglC	intracellular growth locus protein C	16	-0.03	9.5E-01	0	
FTN_0122	recA	Protein RecA	16	-0.24	5.2E-02	0	Homologous recombination
FTN_1710	obg	GTPase obg	16	0.13	1.2E-01	0	
FTN_0842	aroG	phospho-2-dehydro-3-deoxyheptonate aldolase	16	-0.15	1.1E-01	0	Phenylalanine, tyrosine and tryptophan biosynthesis;Metabolic pathways;Biosynthesis of secondary metabolites;Biosynthesis of antibiotics;Biosynthesis of amino acids;Quorum sensing
FTN_1609		membrane fusion protein	16	-0.12	1.9E-01	0	beta-Lactam resistance;Cationic antimicrobial peptide (CAMP) resistance
FTN_0043		hypothetical protein	16	-0.12	4.8E-01	0	
FTN_0893		hypothetical protein	16	0.51	1.8E-03	0	
FTN_1678	nuoC	NADH-quinone oxidoreductase subunit C	16	0.15	1.5E-01	0	Oxidative phosphorylation;Metabolic pathways
FTN_0739	potG	ATP-binding cassette putrescine uptake system, ATP-binding protein	16	-0.06	5.7E-01	0	ABC transporters

FTN_0330	minD	septum site-determining protein MinD	16	0.01	9.4E-01	0	
FTN_1249	serA	D-3-phosphoglycerate dehydrogenase	16	-0.29	1.7E-02	0	Glycine, serine and threonine metabolism;Cysteine and methionine metabolism;Methane metabolism;Metabolic pathways;Microbial metabolism in diverse environments;Biosynthesis of antibiotics;Carbon metabolism;Biosynthesis of amino acids
FTN_0348	ispH	4-hydroxy-3-methylbut-2-enyl diphosphate reductase	16	-0.13	3.0E-01	0	Terpenoid backbone biosynthesis;Metabolic pathways;Biosynthesis of secondary metabolites;Biosynthesis of antibiotics
FTN_0322		VacJ like lipoprotein	16	-0.25	9.9E-02	0	
FTN_0922	ispB	octaprenyl diphosphate synthase	16	-0.02	9.0E-01	0	Terpenoid backbone biosynthesis;Biosynthesis of secondary metabolites
FTN_0355	tolB	Protein tolB	16	0.11	3.2E-01	0	
FTN_0462	glk	glucose kinase (glucokinase)	16	0.21	6.1E-02	0	Glycolysis / Gluconeogenesis;Galactose metabolism;Starch and sucrose metabolism;Amino sugar and nucleotide sugar metabolism;Streptomycin biosynthesis;Metabolic pathways;Biosynthesis of secondary metabolites;Microbial metabolism in diverse environments;Biosynthesis of antibiotics;Carbon metabolism
FTN_0730	acs	acyl-coenzyme A synthetase/AMP-(fatty) acid ligases	16	-0.18	1.2E-01	0	Glycolysis / Gluconeogenesis;Pyruvate metabolism;Glyoxylate and dicarboxylate metabolism;Propanoate metabolism;Methane metabolism;Metabolic pathways;Biosynthesis of secondary metabolites;Microbial metabolism in diverse environments;Biosynthesis of antibiotics;Carbon metabolism
FTN_1593	oppA	ABC-type oligopeptide transport system, periplasmic component	16	0.10	4.3E-01	0	beta-Lactam resistance;ABC transporters;Quorum sensing
FTN_0958		AhpC/TSA family protein	16	-0.07	5.1E-01	0	
FTN_0854		hypothetical protein	16	-0.13	5.2E-01	0	
FTN_0242	rplB	50S ribosomal protein L2	16	-0.06	6.0E-01	0	Ribosome
FTN_1091	aroA	3-phosphoshikimate 1-carboxyvinyltransferase	16	-0.04	6.1E-01	0	Phenylalanine, tyrosine and tryptophan biosynthesis;Metabolic pathways;Biosynthesis of secondary metabolites;Biosynthesis of antibiotics;Biosynthesis of amino acids
FTN_1016		hypothetical protein	16	-0.34	3.6E-02	0	
FTN_0046		hypothetical protein	16	0.17	2.2E-01	0	
FTN_1477	lpxB	Lipid-A-disaccharide synthase	16	0.19	3.9E-02	0	Lipopolysaccharide biosynthesis;Metabolic pathways
FTN_1650	atpF	ATP synthase subunit b	16	0.00	9.8E-01	0	Oxidative phosphorylation;Metabolic pathways
FTN_1201	capB	capsule biosynthesis protein CapB	16	0.02	8.1E-01	0	
FTN_1738		metallocarboxypeptidase	16	0.08	3.9E-01	0	
FTN_1219	galE	UDP-glucose 4-epimerase	16	0.14	1.8E-01	0	Galactose metabolism;Amino sugar and nucleotide sugar metabolism;Metabolic pathways
FTN_1550	parE	DNA topoisomerase IV subunit B	16	0.17	1.6E-01	0	
FTN_0451		signal transduction protein	16	-0.07	6.1E-01	0	
FTN_1229		hypothetical protein	16	0.03	7.7E-01	0	
FTN_0047		hypothetical protein	16	0.10	6.5E-01	0	
FTN_0984		ABC transporter, ATP-binding protein	16	-0.14	4.3E-01	0	
FTN_1622	pilT	Type IV pili nucleotide-binding protein	16	0.16	4.8E-01	0	
FTN_1436	fadD	long chain fatty acid CoA ligase	16	0.68	1.1E-02	0	Fatty acid biosynthesis;Fatty acid degradation;Metabolic pathways;Fatty acid metabolism;Quorum sensing
FTN_1427	wbtD	glycosyl transferase, group 1	16	0.42	5.9E-03	0	
FTN_0263	rpsD	30S ribosomal protein S4	17	-0.12	3.8E-01	0	Ribosome
FTN_1430	wbtQ	aminotransferase	17	0.11	4.0E-01	0	
FTN_1449		hypothetical protein	17	0.29	9.9E-03	0	
FTN_0256	rpsE	30S ribosomal protein S5	17	-0.01	9.2E-01	0	Ribosome
FTN_0550	sohB	putative periplasmic protease	17	0.11	2.1E-01	0	
FTN_0815	bioB	Biotin synthase	17	-0.64	1.2E-01	0	Biotin metabolism;Metabolic pathways
FTN_1602	deoB	phosphopentomutase	17	-0.13	2.0E-01	0	Pentose phosphate pathway;Purine metabolism;Metabolic pathways
FTN_1719		formate dehydrogenase	17	-0.34	3.5E-02	0	Glyoxylate and dicarboxylate metabolism;Methane metabolism;Metabolic pathways;Microbial metabolism in diverse environments;Carbon metabolism
FTN_1739	trpB	Tryptophan synthase beta chain	17	0.19	1.8E-01	0	Glycine, serine and threonine metabolism;Phenylalanine, tyrosine and tryptophan biosynthesis;Metabolic pathways;Biosynthesis of secondary metabolites;Biosynthesis of antibiotics;Biosynthesis of amino acids
FTN_1346		inositol monophosphatase family protein	17	0.00	9.9E-01	0	Streptomycin biosynthesis;Inositol phosphate metabolism;Metabolic pathways;Biosynthesis of antibiotics
FTN_1439	fadA	acetyl-CoA acetyltransferase	17	-0.05	8.5E-01	0	Fatty acid degradation;Valine, leucine and isoleucine degradation;Geraniol degradation;Benzoate degradation;alpha-Linolenic acid metabolism;Metabolic pathways;Biosynthesis of secondary metabolites;Microbial metabolism in diverse environments;Biosynthesis of antibiotics;Fatty acid metabolism
FTN_0279		oxidoreductase	17	0.01	9.2E-01	0	
FTN_0746	alr	Alanine racemase	17	0.01	9.9E-01	0	D-Alanine metabolism;Metabolic pathways;Vancomycin resistance
FTN_0515	glgC	Glucose-1-phosphate adenylyltransferase	17	-0.35	9.1E-02	0	Starch and sucrose metabolism;Amino sugar and nucleotide sugar metabolism;Metabolic pathways;Biosynthesis of secondary metabolites
FTN_1087	cynT	carbonic anhydrase	17	0.14	2.0E-01	0	Nitrogen metabolism;Metabolic pathways
FTN_0851	sufB	cysteine desulfurase activator complex subunit SufB	17	0.01	9.3E-01	0	

FTN_1705		U32 family peptidase	17	-0.02	8.0E-01	0	
FTN_0343		aspartate aminotransferase	17	-0.09	5.6E-01	0	Alanine, aspartate and glutamate metabolism;Cysteine and methionine metabolism;Metabolic pathways;Biosynthesis of amino acids
FTN_0808		acetoacetate decarboxylase	17	-0.01	9.2E-01	0	Propanoate metabolism;Metabolic pathways
FTN_0771		protein-disulfide isomerase	18	0.03	7.4E-01	0	
FTN_0085	uspA	universal stress protein	18	0.01	9.4E-01	0	
FTN_0507	gcvPA	Probable glycine dehydrogenase [decarboxylating] subunit 1	18	0.05	7.3E-01	0	Glycine, serine and threonine metabolism;Glyoxylate and dicarboxylate metabolism;Metabolic pathways;Biosynthesis of secondary metabolites;Biosynthesis of antibiotics;Carbon metabolism
FTN_1004	ychF	GTP-dependent nucleic acid-binding protein EngD	18	-0.46	6.7E-04	0	
FTN_1451		hypothetical protein	18	0.08	6.4E-01	0	
FTN_1596	fimV	Type IV pili, pilus assembly protein	18	-0.08	4.5E-01	0	
FTN_0964		dehydrogenase	18	-0.07	6.7E-01	0	Lysine biosynthesis;Lysine degradation;Metabolic pathways;Biosynthesis of secondary metabolites;Biosynthesis of antibiotics;Biosynthesis of amino acids
FTN_1178		short-chain dehydrogenase	18	-0.01	9.5E-01	0	
FTN_1192		chitin-binding protein	18	-0.18	2.5E-01	0	
FTN_1545	prfA	Peptide chain release factor 1	18	-0.46	2.1E-04	0	
FTN_0423	purK	phosphoribosylaminoimidazole carboxylase ATPase subunit	18	0.12	2.3E-01	0	Purine metabolism;Metabolic pathways;Biosynthesis of secondary metabolites;Biosynthesis of antibiotics
FTN_1076	ispG	4-hydroxy-3-methylbut-2-en-1-yl diphosphate synthase	18	-0.04	6.9E-01	0	Terpenoid backbone biosynthesis;Metabolic pathways;Biosynthesis of secondary metabolites;Biosynthesis of antibiotics
FTN_0925		hypothetical protein	18	-0.18	2.3E-01	0	
FTN_1483	dxr	1-deoxy-D-xylulose 5-phosphate reductoisomerase	18	0.17	5.5E-02	0	Terpenoid backbone biosynthesis;Metabolic pathways;Biosynthesis of secondary metabolites;Biosynthesis of antibiotics
FTN_0962		hypothetical protein	18	0.41	2.6E-02	0	
FTN_1317		hypothetical protein	18	0.38	7.4E-02	0	
FTN_0277	gshA	glutamate-cysteine ligase	18	-0.07	4.5E-01	0	Cysteine and methionine metabolism;Glutathione metabolism;Metabolic pathways
FTN_1487		restriction endonuclease	18	0.05	7.2E-01	0	
FTN_0195	cyoA	cytochrome bo terminal oxidase subunit II	18	-0.23	4.0E-02	0	Oxidative phosphorylation;Metabolic pathways
FTN_0713	ostA2	organic solvent tolerance protein OstA	18	0.55	2.5E-02	0	
FTN_1518	relA	GDP pyrophosphokinase/GTP pyrophosphokinase	18	0.05	7.7E-01	0	Purine metabolism;Metabolic pathways
FTN_1570	rplJ	50S ribosomal protein L10	19	-0.09	2.9E-01	0	Ribosome
FTN_0172	glnA	glutamine synthetase	19	-0.01	9.6E-01	0	Arginine biosynthesis;Alanine, aspartate and glutamate metabolism;Glyoxylate and dicarboxylate metabolism;Nitrogen metabolism;Metabolic pathways;Microbial metabolism in diverse environments;Biosynthesis of amino acids;Two-component system
FTN_0963		NAD-dependent aldehyde dehydrogenase	19	0.04	7.7E-01	0	Glycolysis / Gluconeogenesis;Fatty acid degradation;Valine, leucine and isoleucine degradation;Lysine degradation;Arginine and proline metabolism;Histidine metabolism;Tryptophan metabolism;beta-Alanine metabolism;Glycerolipid metabolism;Pyruvate metabolism;Chloroalkane and chloroalkene degradation;Metabolic pathways;Biosynthesis of secondary metabolites;Microbial metabolism in diverse environments;Biosynthesis of antibiotics
FTN_0419	purM	phosphoribosylaminoimidazole synthetase	19	0.07	5.3E-01	0	Purine metabolism;Metabolic pathways;Biosynthesis of secondary metabolites;Biosynthesis of antibiotics
FTN_0141		ABC transporter, ATP-binding protein	19	-0.55	1.5E-04	0	
FTN_1465		two-component response regulator	19	0.06	4.5E-01	0	Two-component system;Quorum sensing
FTN_0839		hypothetical protein	19	0.08	7.3E-01	0	
FTN_1425	wbtF	NAD dependent epimerase	19	0.03	7.8E-01	0	Amino sugar and nucleotide sugar metabolism;Metabolic pathways
FTN_0073	yidC	Membrane protein insertase YidC	19	0.04	7.4E-01	0	Quorum sensing;Protein export;Bacterial secretion system
FTN_1636	sdhB	succinate dehydrogenase iron-sulfur subunit	19	0.00	9.9E-01	0	Citrate cycle (TCA cycle);Oxidative phosphorylation;Butanoate metabolism;Metabolic pathways;Biosynthesis of secondary metabolites;Microbial metabolism in diverse environments;Biosynthesis of antibiotics;Carbon metabolism
FTN_0663	pgi	Glucose-6-phosphate isomerase	19	-0.19	6.5E-02	0	Glycolysis / Gluconeogenesis;Pentose phosphate pathway;Starch and sucrose metabolism;Amino sugar and nucleotide sugar metabolism;Metabolic pathways;Biosynthesis of secondary metabolites;Microbial metabolism in diverse environments;Biosynthesis of antibiotics;Carbon metabolism
FTN_0781	talA	transaldolase B	19	0.01	8.8E-01	0	Pentose phosphate pathway;Metabolic pathways;Biosynthesis of secondary metabolites;Microbial metabolism in diverse environments;Biosynthesis of antibiotics;Carbon metabolism;Biosynthesis of amino acids
FTN_0021	carA	carbamoyl phosphate synthase small subunit	19	0.26	6.4E-02	0	Pyrimidine metabolism;Alanine, aspartate and glutamate metabolism;Metabolic pathways
FTN_1412	rpoA2	DNA-directed RNA polymerase subunit alpha 2	19	0.20	3.9E-02	0	RNA polymerase
FTN_1433		hypothetical protein	19	-0.01	9.1E-01	0	
FTN_0861		hypothetical protein	19	-0.14	5.5E-01	0	
FTN_1410	bfr	bacterioferritin	19	0.10	5.4E-01	0	Porphyrin and chlorophyll metabolism

FTN_1563	metK	S-adenosylmethionine synthase	19	0.18	1.6E-01	0	Cysteine and methionine metabolism;Metabolic pathways;Biosynthesis of secondary metabolites;Biosynthesis of amino acids
FTN_0633	katG	Catalase-peroxidase	19	0.12	3.3E-01	0	Phenylalanine metabolism;Tryptophan metabolism;Metabolic pathways;Biosynthesis of secondary metabolites
FTN_0883	pheS	Phenylalanine--tRNA ligase alpha subunit	19	0.26	2.1E-02	0	Aminoacyl-tRNA biosynthesis
FTN_0196	cyoB	cytochrome bo terminal oxidase subunit I	19	-0.13	2.6E-01	0	Oxidative phosphorylation;Metabolic pathways
FTN_0593	sucD	succinyl-CoA synthetase, alpha subunit	20	-0.02	8.3E-01	0	Citrate cycle (TCA cycle);Propanoate metabolism;C5-Branched dibasic acid metabolism;Metabolic pathways;Biosynthesis of secondary metabolites;Microbial metabolism in diverse environments;Biosynthesis of antibiotics;Carbon metabolism
FTN_1647	atpG	ATP synthase gamma chain	20	-0.01	8.8E-01	0	Oxidative phosphorylation;Metabolic pathways
FTN_0805	hemL	Glutamate-1-semialdehyde 2,1-aminomutase	20	-0.06	6.5E-01	0	Porphyrin and chlorophyll metabolism;Metabolic pathways;Biosynthesis of secondary metabolites;Microbial metabolism in diverse environments
FTN_0357	pal	OmpA family peptidoglycan-associated lipoprotein	20	-0.06	5.2E-01	0	
FTN_1703	tolC	outer membrane protein tolC precursor	20	-0.19	9.1E-02	0	beta-Lactam resistance;Cationic antimicrobial peptide (CAMP) resistance;Two-component system;Bacterial secretion system
FTN_1053		hypothetical protein	20	-0.02	8.1E-01	0	
FTN_1675	nuoF	NADH dehydrogenase I, F subunit	20	0.35	2.3E-03	0	Oxidative phosphorylation;Metabolic pathways
FTN_1056	clpX	ATP-dependent protease ATP-binding subunit ClpX	20	-0.13	1.8E-01	0	
FTN_0917		serine-type D-Ala-D-Ala carboxypeptidase	20	0.48	6.8E-04	0	Peptidoglycan biosynthesis
FTN_0553	hemB	delta-aminolevulinic acid dehydratase	20	0.12	4.7E-01	0	Porphyrin and chlorophyll metabolism;Metabolic pathways;Biosynthesis of secondary metabolites;Microbial metabolism in diverse environments
FTN_1182	mnmG	tRNA uridine 5-carboxymethylaminomethyl modification enzyme MnmG	20	-0.79	3.6E-02	0	
FTN_0897	guaA	GMP synthase [glutamine-hydrolyzing]	20	0.00	9.6E-01	0	Purine metabolism;Metabolic pathways
FTN_1106	metN	methionine uptake transporter (MUT) family protein	20	-0.28	1.2E-01	0	ABC transporters
FTN_0516	glgA	Glycogen synthase	20	-0.11	4.6E-01	0	Starch and sucrose metabolism;Metabolic pathways;Biosynthesis of secondary metabolites
FTN_1664	hemE	Uroporphyrinogen decarboxylase	20	0.00	9.8E-01	0	Porphyrin and chlorophyll metabolism;Metabolic pathways;Biosynthesis of secondary metabolites
FTN_0834	gor	glutathione reductase	20	-0.03	7.3E-01	0	Glutathione metabolism;Metabolic pathways
FTN_1198	spoT	GDP diphosphokinase/guanosine-3',5'-bis(diphosphate) 3'-diphosphatase	20	-0.05	6.1E-01	0	Purine metabolism;Metabolic pathways
FTN_0499	dnaE	DNA polymerase III alpha subunit	20	0.07	6.5E-01	0	DNA replication;Mismatch repair;Homologous recombination
FTN_0992	tyrS	tyrosyl-tRNA synthetase	20	-0.11	5.2E-01	0	Aminoacyl-tRNA biosynthesis
FTN_1159	ggt	gamma-glutamyltranspeptidase	20	-0.18	3.0E-01	0	Taurine and hypotaurine metabolism;Cyanoamino acid metabolism;Glutathione metabolism;Metabolic pathways
FTN_0492	parC	DNA topoisomerase IV subunit A	20	0.01	9.4E-01	0	
FTN_0756	fopA	OmpA family protein	21	-0.10	3.2E-01	0	
FTN_0337	fumA	fumerate hydratase	21	0.18	9.5E-02	0	Citrate cycle (TCA cycle);Pyruvate metabolism;Metabolic pathways;Biosynthesis of secondary metabolites;Microbial metabolism in diverse environments;Biosynthesis of antibiotics;Carbon metabolism
FTN_0310	cysS	Cysteine--tRNA ligase	21	-0.20	5.8E-02	0	Aminoacyl-tRNA biosynthesis
FTN_0780	pepB	cytosol aminopeptidase	21	0.13	2.4E-01	0	Glutathione metabolism;Metabolic pathways
FTN_0193	cydA	cytochrome bd-I terminal oxidase subunit I	21	-0.08	6.3E-01	0	Oxidative phosphorylation;Two-component system
FTN_1632	glmM	Phosphoglucosamine mutase	21	0.05	5.3E-01	0	Amino sugar and nucleotide sugar metabolism;Metabolic pathways;Biosynthesis of antibiotics
FTN_1008	prsA	ribose-phosphate pyrophosphokinase	21	0.02	8.2E-01	0	Pentose phosphate pathway;Purine metabolism;Metabolic pathways;Biosynthesis of secondary metabolites;Microbial metabolism in diverse environments;Biosynthesis of antibiotics;Carbon metabolism;Biosynthesis of amino acids
FTN_1261		hypothetical protein	21	0.01	9.6E-01	0	
FTN_0955	serC	phosphoserine aminotransferase	21	0.47	1.7E-04	0	Glycine, serine and threonine metabolism;Cysteine and methionine metabolism;Methane metabolism;Vitamin B6 metabolism;Metabolic pathways;Microbial metabolism in diverse environments;Biosynthesis of antibiotics;Carbon metabolism;Biosynthesis of amino acids
FTN_0900		hypothetical protein	21	-0.07	6.8E-01	0	
FTN_0455		CheB methylesterase/CheR methyltransferase	21	0.46	8.5E-03	0	Two-component system;Bacterial chemotaxis
FTN_0112	ribAB	3,4-dihydroxy-2-butanone 4-phosphate synthase/GTP cyclohydrolase II	21	0.07	7.8E-01	0	Riboflavin metabolism;Folate biosynthesis;Metabolic pathways;Biosynthesis of secondary metabolites
FTN_0270	pyrG	CTP synthase	22	0.08	4.3E-01	0	Pyrimidine metabolism;Metabolic pathways
FTN_0825		aldo/keto reductase family protein	22	0.09	3.1E-01	0	
FTN_0446	der	GTPase Der	22	0.07	4.8E-01	0	
FTN_0264	rpoA1	DNA-directed RNA polymerase subunit alpha 1	22	-0.21	3.0E-02	0	RNA polymerase
FTN_0484	glmU	Bifunctional protein GlmU [Includes: UDP-N-acetylglucosamine pyrophosphorylase ; Glucosamine-1-phosphate N-acetyltransferase]	22	0.05	5.6E-01	0	Amino sugar and nucleotide sugar metabolism;Metabolic pathways;Biosynthesis of antibiotics
FTN_0346		OmpA family protein	22	0.26	7.0E-02	0	
FTN_0425	prlC	oligopeptidase A	22	0.06	4.7E-01	0	
FTN_1337	fabH	3-oxoacyl-[acyl-carrier protein] synthase III	22	-0.19	1.0E-01	0	Fatty acid biosynthesis;Metabolic pathways;Fatty acid metabolism

FTN_0294	ftsK	cell division protein	22	-0.17	1.5E-01	0	
FTN_0126	pta	phosphate acetyltransferase	22	-0.07	4.7E-01	0	Taurine and hypotaurine metabolism;Pyruvate metabolism;Propanoate metabolism;Methane metabolism;Metabolic pathways;Microbial metabolism in diverse environments;Carbon metabolism
FTN_0517	glgP	glycogen phosphorylase	22	-0.04	6.9E-01	0	Starch and sucrose metabolism;Metabolic pathways;Biosynthesis of secondary metabolites
FTN_0040		hypothetical protein	22	-0.13	2.9E-01	0	
FTN_1597	prfC	Peptide chain release factor 3	22	-0.26	1.7E-02	0	
FTN_0131		hypothetical protein	22	0.12	6.9E-01	0	
FTN_0496	slt	soluble lytic murein transglycosylase	22	0.01	9.9E-01	0	
FTN_1319	pdpC	hypothetical protein	22	0.18	6.6E-01	0	
FTN_1571	rplA	50S ribosomal protein L1	23	-0.13	2.1E-01	0	Ribosome
FTN_1040	ilvC	ketol-acid reductoisomerase	23	-0.07	6.6E-01	0	Valine, leucine and isoleucine biosynthesis;Pantothenate and CoA biosynthesis;Metabolic pathways;Biosynthesis of secondary metabolites;Biosynthesis of antibiotics;2-Oxocarboxylic acid metabolism;Biosynthesis of amino acids
FTN_1338	fabD	malonyl-CoA:ACP transacylase	23	0.01	8.7E-01	0	Fatty acid biosynthesis;Metabolic pathways;Biosynthesis of antibiotics;Fatty acid metabolism
FTN_0595		hypothetical protein	23	0.20	4.6E-02	0	
FTN_0999	udhA	soluble pyridine nucleotide transhydrogenase	23	-0.05	5.5E-01	0	Nicotinate and nicotinamide metabolism;Metabolic pathways
FTN_0298	glpX	fructose 1,6-bisphosphatase II	23	0.07	4.9E-01	0	Glycolysis / Gluconeogenesis;Pentose phosphate pathway;Fructose and mannose metabolism;Methane metabolism;Metabolic pathways;Biosynthesis of secondary metabolites;Microbial metabolism in diverse environments;Biosynthesis of antibiotics;Carbon metabolism
FTN_0024	pyrC	dihydroorotase	23	0.13	2.9E-01	0	Pyrimidine metabolism;Metabolic pathways
FTN_0061	leuC	isopropylmalate isomerase large subunit	23	-0.08	6.9E-01	0	Valine, leucine and isoleucine biosynthesis;C5-Branched dibasic acid metabolism;Metabolic pathways;Biosynthesis of secondary metabolites;2-Oxocarboxylic acid metabolism;Biosynthesis of amino acids
FTN_0983		bifunctional gluaredoxin/ribonucleoside-diphosphate reductase subunit beta	23	0.11	2.8E-01	0	Purine metabolism;Pyrimidine metabolism;Metabolic pathways
FTN_1115	pilB	Type IV pili ATPase	23	0.01	9.6E-01	0	
FTN_0621	eno	Enolase	24	-0.19	2.6E-01	0	Glycolysis / Gluconeogenesis;Methane metabolism;Metabolic pathways;Biosynthesis of secondary metabolites;Microbial metabolism in diverse environments;Biosynthesis of antibiotics;Carbon metabolism;Biosynthesis of amino acids;RNA degradation
FTN_0163	ftsA	cell division protein FtsA	24	0.02	7.4E-01	0	
FTN_0594	sucC	Succinyl-CoA ligase [ADP-forming] subunit beta	24	0.02	8.1E-01	0	Citrate cycle (TCA cycle);Propanoate metabolism;C5-Branched dibasic acid metabolism;Metabolic pathways;Biosynthesis of secondary metabolites;Microbial metabolism in diverse environments;Biosynthesis of antibiotics;Carbon metabolism
FTN_0178	purA	Adenylosuccinate synthetase	24	-0.05	5.8E-01	0	Purine metabolism;Alanine, aspartate and glutamate metabolism;Metabolic pathways
FTN_0648	gpmI	2,3-bisphosphoglycerate-independent phosphoglycerate mutase	24	-0.20	9.9E-02	0	Glycolysis / Gluconeogenesis;Glycine, serine and threonine metabolism;Methane metabolism;Metabolic pathways;Biosynthesis of secondary metabolites;Microbial metabolism in diverse environments;Biosynthesis of antibiotics;Carbon metabolism;Biosynthesis of amino acids
FTN_1210		ribokinase	24	-0.08	3.7E-01	0	
FTN_1689	gatB	Aspartyl/glutamyl-tRNA(Asn/Gln) amidotransferase subunit B	24	-0.05	5.7E-01	0	Aminoacyl-tRNA biosynthesis;Metabolic pathways
FTN_1146		aspartate aminotransferase	24	0.01	9.2E-01	0	Arginine biosynthesis;Alanine, aspartate and glutamate metabolism;Cysteine and methionine metabolism;Arginine and proline metabolism;Tyrosine metabolism;Phenylalanine metabolism;Phenylalanine, tyrosine and tryptophan biosynthesis;Novobiocin biosynthesis;Metabolic pathways;Biosynthesis of secondary metabolites;Biosynthesis of antibiotics;2-Oxocarboxylic acid metabolism;Biosynthesis of amino acids
FTN_0116	ipdC	indolepyruvate decarboxylase	24	0.04	6.7E-01	0	Tryptophan metabolism;Metabolic pathways
FTN_0527	thrC	threonine synthase	24	0.09	2.6E-01	0	Glycine, serine and threonine metabolism;Vitamin B6 metabolism;Metabolic pathways;Biosynthesis of secondary metabolites;Microbial metabolism in diverse environments;Biosynthesis of amino acids
FTN_1649	atpH	ATP synthase subunit delta	24	0.09	3.4E-01	0	Oxidative phosphorylation;Metabolic pathways
FTN_1700	purF	amidophosphoribosyltransferase	24	-0.01	9.1E-01	0	Purine metabolism;Alanine, aspartate and glutamate metabolism;Metabolic pathways;Biosynthesis of secondary metabolites;Biosynthesis of antibiotics
FTN_0107	lepA	Elongation factor 4	24	0.03	7.6E-01	0	
FTN_1049		hypothetical protein	24	0.06	5.2E-01	0	
FTN_0601	pdxS	Pyridoxal biosynthesis lyase pdxS	25	0.03	8.2E-01	0	Vitamin B6 metabolism
FTN_1336	plsX	Phosphate acyltransferase	25	-0.02	7.9E-01	0	Glycerolipid metabolism;Metabolic pathways;Biosynthesis of secondary metabolites
FTN_1367		hypothetical protein	25	0.00	1.0E+00	0	

FTN_1493	aceF	dihydrolipoamide acetyltransferase	25	0.03	7.2E-01	0	Glycolysis / Gluconeogenesis;Citrate cycle (TCA cycle);Pyruvate metabolism;Metabolic pathways;Biosynthesis of secondary metabolites;Microbial metabolism in diverse environments;Biosynthesis of antibiotics;Carbon metabolism
FTN_1095	secD	preprotein translocase subunit SecD	25	-0.05	6.2E-01	0	Protein export;Bacterial secretion system
FTN_0227	rpsB	30S ribosomal protein S2	25	-0.16	1.8E-01	0	Ribosome
FTN_1063	miaB	(Dimethylallyl)adenosine tRNA methylthiotransferase MiaB	25	-0.09	3.2E-01	0	
FTN_0177	purH	bifunctional phosphoribosylaminoimidazolecarboxamide formyltransferase/IMP cyclohydrolase	25	0.01	9.3E-01	0	Purine metabolism;One carbon pool by folate;Metabolic pathways;Biosynthesis of secondary metabolites;Biosynthesis of antibiotics
FTN_0637	ugpQ	glycerophosphoryl diester phosphodiesterase	25	0.07	5.8E-01	0	Glycerophospholipid metabolism
FTN_0626	kbl	2-amino-3-ketobutyrate coenzyme A ligase	25	0.09	4.0E-01	0	Glycine, serine and threonine metabolism;Metabolic pathways
FTN_0062	leuA	2-isopropylmalate synthase	25	-0.15	1.6E-01	0	Valine, leucine and isoleucine biosynthesis;Pyruvate metabolism;Metabolic pathways;Biosynthesis of secondary metabolites;2-Oxocarboxylic acid metabolism;Biosynthesis of amino acids
FTN_0627	chiA	glycosyl hydrolase family chitinase	25	-0.27	5.4E-02	0	Amino sugar and nucleotide sugar metabolism;Metabolic pathways
FTN_1421	wbtH	glutamine amidotransferase/asparagine synthase	25	-0.35	6.6E-03	0	Alanine, aspartate and glutamate metabolism;Metabolic pathways;Biosynthesis of secondary metabolites;Biosynthesis of amino acids
FTN_0539	glnS	glutaminyl-tRNA synthetase	25	-0.14	2.0E-01	0	Aminoacyl-tRNA biosynthesis;Metabolic pathways
FTN_1377	proS	Proline--tRNA ligase	25	-0.09	3.3E-01	0	Aminoacyl-tRNA biosynthesis
FTN_1661	nusA	transcription elongation factor NusA	26	-0.05	6.5E-01	0	
FTN_1259	glyA	Pyridoxal-phosphate-dependent serine hydroxymethyltransferase	26	-0.13	2.1E-01	0	Glycine, serine and threonine metabolism;Cyanoamino acid metabolism;Glyoxylate and dicarboxylate metabolism;One carbon pool by folate;Methane metabolism;Metabolic pathways;Biosynthesis of secondary metabolites;Microbial metabolism in diverse environments;Biosynthesis of antibiotics;Carbon metabolism;Biosynthesis of amino acids
FTN_1228	fabI	enoyl-ACP reductase I	26	0.09	2.0E-01	0	Fatty acid biosynthesis;Biotin metabolism;Metabolic pathways;Biosynthesis of antibiotics;Fatty acid metabolism
FTN_0505	gcvT	Aminomethyltransferase	26	-0.05	6.1E-01	0	Glycine, serine and threonine metabolism;Glyoxylate and dicarboxylate metabolism;One carbon pool by folate;Metabolic pathways;Biosynthesis of secondary metabolites;Biosynthesis of antibiotics;Carbon metabolism
FTN_1677	nuoD	NADH-quinone oxidoreductase subunit D	26	0.09	3.1E-01	0	Oxidative phosphorylation;Metabolic pathways
FTN_1715	kdpD	two component regulator, sensor histidine kinase kdpD	26	-0.30	3.7E-01	0	Two-component system
FTN_0525	thrA	aspartate kinase I/homoserine dehydrogenase I	26	-0.30	5.8E-02	0	Glycine, serine and threonine metabolism;Monobactam biosynthesis;Cysteine and methionine metabolism;Lysine biosynthesis;Metabolic pathways;Biosynthesis of secondary metabolites;Microbial metabolism in diverse environments;Biosynthesis of antibiotics;Biosynthesis of amino acids
FTN_0604		AMP-binding protein	26	-0.03	7.3E-01	0	
FTN_1584	glpD	glycerol-3-phosphate dehydrogenase	26	-0.15	2.0E-01	0	Glycerophospholipid metabolism;Biosynthesis of secondary metabolites
FTN_1690	gatA	Glutamyl-tRNA(Gln) amidotransferase subunit A	26	-0.20	5.7E-02	0	Aminoacyl-tRNA biosynthesis;Metabolic pathways
FTN_1309	pdpA	hypothetical protein	26	0.20	4.4E-01	0	
FTN_1509	mutS	DNA mismatch repair protein mutS	26	0.07	6.0E-01	0	Mismatch repair
FTN_1637	sdhA	succinate dehydrogenase flavoprotein	27	0.09	3.7E-01	0	Citrate cycle (TCA cycle);Oxidative phosphorylation;Butanoate metabolism;Metabolic pathways;Biosynthesis of secondary metabolites;Microbial metabolism in diverse environments;Biosynthesis of antibiotics;Carbon metabolism
FTN_1332	gapA	glyceraldehyde-3-phosphate dehydrogenase/erythrose-4-phosphate dehydrogenase	27	0.05	7.8E-01	0	Glycolysis / Gluconeogenesis;Metabolic pathways;Biosynthesis of secondary metabolites;Microbial metabolism in diverse environments;Biosynthesis of antibiotics;Carbon metabolism;Biosynthesis of amino acids
FTN_0661	guaB	IMP dehydrogenase/GMP reductase	27	0.06	6.8E-01	0	Purine metabolism;Metabolic pathways;Biosynthesis of secondary metabolites
FTN_0251	rplE	50S ribosomal protein L5	27	-0.08	4.2E-01	0	Ribosome
FTN_1694	purB	adenylosuccinate lyase	27	0.04	5.8E-01	0	Purine metabolism;Alanine, aspartate and glutamate metabolism;Metabolic pathways;Biosynthesis of secondary metabolites;Biosynthesis of antibiotics
FTN_0647	serS	Serine--tRNA ligase	27	0.07	3.4E-01	0	Aminoacyl-tRNA biosynthesis
FTN_0064	ppdK	pyruvate phosphate dikinase	27	-0.08	5.2E-01	0	Pyruvate metabolism;Metabolic pathways;Microbial metabolism in diverse environments;Carbon metabolism
FTN_1770		bifunctional indole-3-glycerol phosphate synthase/phosphoribosylanthranilate isomerase	27	-0.05	7.0E-01	0	Phenylalanine, tyrosine and tryptophan biosynthesis;Metabolic pathways;Biosynthesis of secondary metabolites;Biosynthesis of antibiotics;Biosynthesis of amino acids
FTN_1644		hypothetical protein	27	-0.02	8.5E-01	0	
FTN_1461	rnr	ribonuclease R	27	-0.20	2.1E-01	0	RNA degradation
FTN_1043	ilvD	Dihydroxy-acid dehydratase	28	-0.11	1.5E-01	0	Valine, leucine and isoleucine biosynthesis;Pantothenate and CoA biosynthesis;Metabolic pathways;Biosynthesis of secondary metabolites;Biosynthesis of antibiotics;2-Oxocarboxylic acid metabolism;Biosynthesis of amino acids
FTN_1074		X-prolyl aminopeptidase 2	28	-0.10	4.5E-01	0	
FTN_0221	gltX	Glutamate--tRNA ligase	28	-0.02	8.2E-01	0	Porphyrin and chlorophyll metabolism;Aminoacyl-tRNA biosynthesis;Metabolic pathways;Biosynthesis of secondary metabolites;Microbial metabolism in diverse environments

FTN_0669	deoD	purine nucleoside phosphorylase	28	-0.09	3.7E-01	0	Purine metabolism;Pyrimidine metabolism;Nicotinate and nicotinamide metabolism;Metabolic pathways;Biosynthesis of secondary metabolites
FTN_0410		aspartate aminotransferase	28	0.00	9.6E-01	0	Arginine biosynthesis;Alanine, aspartate and glutamate metabolism;Cysteine and methionine metabolism;Arginine and proline metabolism;Tyrosine metabolism;Phenylalanine metabolism;Phenylalanine, tyrosine and tryptophan biosynthesis;Novobiocin biosynthesis;Metabolic pathways;Biosynthesis of secondary metabolites;Biosynthesis of antibiotics;2-Oxocarboxylic acid metabolism;Biosynthesis of amino acids
FTN_0002	dnaN	DNA polymerase III, beta subunit	28	0.11	1.4E-01	0	DNA replication;Mismatch repair;Homologous recombination
FTN_1717	kdpB	potassium-transporting ATPase B chain	28	-0.05	5.8E-01	0	Two-component system
FTN_0468	metG	Methionine--tRNA ligase	28	-0.14	1.3E-01	0	Selenocompound metabolism;Aminoacyl-tRNA biosynthesis;Metabolic pathways
FTN_0996	hslU	ATP-dependent protease ATPase subunit HslU	28	-0.20	3.2E-01	0	
FTN_0869		hypothetical protein	28	0.05	6.4E-01	0	
FTN_1492	lpdA	dihydrolipoamide dehydrogenase	29	0.00	9.7E-01	0	Glycolysis / Gluconeogenesis;Citrate cycle (TCA cycle);Glycine, serine and threonine metabolism;Valine, leucine and isoleucine degradation;Lysine degradation;Tryptophan metabolism;Pyruvate metabolism;Glyoxylate and dicarboxylate metabolism;Propanoate metabolism;Metabolic pathways;Biosynthesis of secondary metabolites;Microbial metabolism in diverse environments;Biosynthesis of antibiotics;Carbon metabolism
FTN_1768	pepN	aminopeptidase N	29	0.21	2.5E-01	0	Glutathione metabolism;Metabolic pathways
FTN_1172		hypothetical protein	29	0.22	2.8E-02	0	
FTN_1604	polA	DNA polymerase I	29	-0.25	3.9E-02	0	DNA replication;Base excision repair;Nucleotide excision repair;Homologous recombination
FTN_0980	mdh	Malate dehydrogenase	30	0.03	8.0E-01	0	Citrate cycle (TCA cycle);Cysteine and methionine metabolism;Pyruvate metabolism;Glyoxylate and dicarboxylate metabolism;Methane metabolism;Metabolic pathways;Biosynthesis of secondary metabolites;Microbial metabolism in diverse environments;Biosynthesis of antibiotics;Carbon metabolism
FTN_1329	fbaA	fructose-1,6-bisphosphate aldolase	30	0.07	6.0E-01	0	Glycolysis / Gluconeogenesis;Pentose phosphate pathway;Fructose and mannose metabolism;Methane metabolism;Metabolic pathways;Biosynthesis of secondary metabolites;Microbial metabolism in diverse environments;Biosynthesis of antibiotics;Carbon metabolism;Biosynthesis of amino acids
FTN_1331	pgk	Phosphoglycerate kinase	30	0.06	5.4E-01	0	Glycolysis / Gluconeogenesis;Metabolic pathways;Biosynthesis of secondary metabolites;Microbial metabolism in diverse environments;Biosynthesis of antibiotics;Carbon metabolism;Biosynthesis of amino acids
FTN_0690	deaD	DEAD-box subfamily ATP-dependent helicase	30	-0.10	5.7E-01	0	RNA degradation
FTN_0843	ffh	signal recognition particle GTPase	30	-0.14	1.3E-01	0	Quorum sensing;Protein export;Bacterial secretion system
FTN_1610		RND efflux transporter	30	0.17	6.7E-02	0	beta-Lactam resistance;Cationic antimicrobial peptide (CAMP) resistance
FTN_1426	wbtE	UDP-glucose/GDP-mannose dehydrogenase	30	0.12	3.0E-01	0	Amino sugar and nucleotide sugar metabolism;Metabolic pathways
FTN_1444		ornithine cyclodeaminase	30	0.22	5.3E-02	0	Arginine and proline metabolism;Metabolic pathways;Biosynthesis of secondary metabolites;Biosynthesis of antibiotics;Biosynthesis of amino acids
FTN_0750	sdaA	L-serine dehydratase	30	0.12	3.8E-01	0	Glycine, serine and threonine metabolism;Cysteine and methionine metabolism;Metabolic pathways;Biosynthesis of secondary metabolites;Biosynthesis of antibiotics;Carbon metabolism;Biosynthesis of amino acids
FTN_1042	ilvB	acetolactate synthase large subunit	30	-0.18	7.1E-02	0	Valine, leucine and isoleucine biosynthesis;Butanoate metabolism;C5-Branched dibasic acid metabolism;Pantothenate and CoA biosynthesis;Metabolic pathways;Biosynthesis of secondary metabolites;Biosynthesis of antibiotics;2-Oxocarboxylic acid metabolism;Biosynthesis of amino acids
FTN_1634	sucB	2-oxoglutarate dehydrogenase complex, E2 component, dihydrolipoyltranssuccinase	31	0.06	4.3E-01	0	Citrate cycle (TCA cycle);Lysine degradation;Tryptophan metabolism;Metabolic pathways;Biosynthesis of secondary metabolites;Microbial metabolism in diverse environments;Biosynthesis of antibiotics;Carbon metabolism
FTN_0443	maeA	malate dehydrogenase	31	0.27	3.3E-03	0	Pyruvate metabolism;Carbon metabolism;Two-component system
FTN_1585	glpK	Glycerol kinase	31	-0.08	5.1E-01	0	Glycerolipid metabolism;Metabolic pathways
FTN_0432	topA	DNA topoisomerase I	31	-0.20	1.1E-01	0	
FTN_1448		hypothetical protein	32	0.04	7.1E-01	0	
FTN_0907		D-alanyl-D-alanine carboxypeptidase	32	0.26	1.7E-02	0	Peptidoglycan biosynthesis;Metabolic pathways
FTN_0668	hflB	ATP-dependent metalloprotease	32	0.04	6.3E-01	0	
FTN_1762		putative ABC transporter ATP-binding protein	32	0.02	8.3E-01	0	
FTN_1484	gyrA	DNA gyrase, subunit A	32	0.04	5.7E-01	0	
FTN_1437	fadE	acyl-CoA dehydrogenase	32	0.58	1.3E-02	0	Fatty acid degradation;Valine, leucine and isoleucine degradation;Metabolic pathways;Biosynthesis of secondary metabolites;Biosynthesis of antibiotics;Fatty acid metabolism
FTN_0508	gcvPB	Probable glycine dehydrogenase [decarboxylating] subunit 2	32	0.13	5.7E-01	0	Glycine, serine and threonine metabolism;Glyoxylate and dicarboxylate metabolism;Metabolic pathways;Biosynthesis of secondary metabolites;Biosynthesis of antibiotics;Carbon metabolism
FTN_1648	atpA	ATP synthase subunit alpha	33	-0.07	4.0E-01	0	Oxidative phosphorylation;Metabolic pathways

FTN_0228	tsf	Elongation factor Ts	33	-0.25	8.5E-02	0	
FTN_1508	accA	Acetyl-coenzyme A carboxylase carboxyl transferase subunit alpha	33	0.00	9.8E-01	0	Fatty acid biosynthesis;Pyruvate metabolism;Propanoate metabolism;Metabolic pathways;Biosynthesis of secondary metabolites;Microbial metabolism in diverse environments;Biosynthesis of antibiotics;Carbon metabolism;Fatty acid metabolism
FTN_0485	glmS	glucosamine--fructose-6-phosphate aminotransferase	33	-0.15	1.5E-01	0	Alanine, aspartate and glutamate metabolism;Amino sugar and nucleotide sugar metabolism;Metabolic pathways;Biosynthesis of antibiotics
FTN_1157		GTP binding translational elongation factor Tu and G family protein	33	-0.13	3.8E-01	0	
FTN_0778	alaS	Alanine--tRNA ligase	33	-0.09	2.9E-01	0	Aminoacyl-tRNA biosynthesis
FTN_1330	pyk	pyruvate kinase	34	-0.02	8.8E-01	0	Glycolysis / Gluconeogenesis;Purine metabolism;Pyruvate metabolism;Metabolic pathways;Biosynthesis of secondary metabolites;Microbial metabolism in diverse environments;Biosynthesis of antibiotics;Carbon metabolism;Biosynthesis of amino acids
FTN_1044		hypothetical protein	34	-0.14	1.7E-01	0	Glycine, serine and threonine metabolism;Vitamin B6 metabolism;Metabolic pathways;Biosynthesis of secondary metabolites;Microbial metabolism in diverse environments;Biosynthesis of amino acids
FTN_1111		Mur ligase family protein	34	0.10	3.2E-01	0	
FTN_0034		hypothetical protein	34	0.13	4.2E-01	0	
FTN_1143		4Fe-4S ferredoxin, FAD dependent	34	0.08	5.6E-01	0	
FTN_0757		membrane protein of unknown function	34	-0.21	3.0E-01	0	
FTN_0911		alpha-glucosidase	34	0.19	3.9E-01	0	
FTN_0420		SAICAR synthetase/phosphoribosylamine-glycine ligase	34	-0.13	1.6E-01	0	Purine metabolism;Metabolic pathways;Biosynthesis of secondary metabolites;Biosynthesis of antibiotics
FTN_1341	fabF	beta-ketoacyl-ACP synthase II	35	-0.01	9.0E-01	0	Fatty acid biosynthesis;Biotin metabolism;Metabolic pathways;Fatty acid metabolism
FTN_1323	iglB	intracellular growth locus protein B	35	0.06	6.1E-01	0	
FTN_0168	lysU	lysyl-tRNA synthetase	35	0.07	5.6E-01	0	Aminoacyl-tRNA biosynthesis
FTN_1191	thrS	Threonine--tRNA ligase	35	-0.10	1.9E-01	0	Aminoacyl-tRNA biosynthesis
FTN_0666	uvrA	excinuclease ABC, subunit A	35	-0.16	4.3E-02	0	Nucleotide excision repair
FTN_1284	dnaK	Chaperone protein DnaK	36	-0.10	4.2E-01	0	RNA degradation
FTN_1640	gltA	citrate synthase	37	-0.19	2.4E-01	0	Citrate cycle (TCA cycle);Glyoxylate and dicarboxylate metabolism;Metabolic pathways;Biosynthesis of secondary metabolites;Microbial metabolism in diverse environments;Biosynthesis of antibiotics;Carbon metabolism;2-Oxocarboxylic acid metabolism;Biosynthesis of amino acids
FTN_1186	pepO	M13 family metallopeptidase	37	0.09	6.4E-01	0	
FTN_0564	accC	acetyl-CoA carboxylase, biotin carboxylase subunit	37	0.01	9.4E-01	0	Fatty acid biosynthesis;Pyruvate metabolism;Propanoate metabolism;Metabolic pathways;Biosynthesis of secondary metabolites;Microbial metabolism in diverse environments;Biosynthesis of antibiotics;Carbon metabolism;Fatty acid metabolism
FTN_0557	argS	Arginine--tRNA ligase	37	0.14	1.6E-01	0	Aminoacyl-tRNA biosynthesis
FTN_0649		4Fe-4S ferredoxin, FAD dependent	37	0.33	5.2E-03	0	
FTN_0609	pnp	Polyribonucleotide nucleotidyltransferase	38	-0.07	4.1E-01	0	RNA degradation
FTN_0600	gyrB	DNA gyrase subunit B	38	-0.03	7.3E-01	0	
FTN_1333	tktA	transketolase	38	-0.02	8.1E-01	0	Pentose phosphate pathway;Metabolic pathways;Biosynthesis of secondary metabolites;Microbial metabolism in diverse environments;Biosynthesis of antibiotics;Carbon metabolism;Biosynthesis of amino acids
FTN_0981	nrdA	ribonucleotide-diphosphate reductase subunit alpha	38	0.13	2.9E-01	0	Purine metabolism;Pyrimidine metabolism;Metabolic pathways
FTN_1416	rho	transcription termination factor Rho	39	-0.17	1.3E-01	0	RNA degradation
FTN_0736	glyS	glycyl-tRNA synthetase beta subunit	39	-0.11	3.6E-01	0	Aminoacyl-tRNA biosynthesis
FTN_0129	aspS	aspartyl-tRNA synthetase	39	0.01	9.1E-01	0	Aminoacyl-tRNA biosynthesis
FTN_1325	pdpD	hypothetical protein	39	0.51	3.3E-01	0	
FTN_0913	rpoD	RNA polymerase sigma-70 factor	40	0.01	9.1E-01	0	
FTN_0272	accD	acetyl-CoA carboxylase, carboxytransferase subunit beta	40	-0.09	3.8E-01	0	Fatty acid biosynthesis;Pyruvate metabolism;Propanoate metabolism;Metabolic pathways;Biosynthesis of secondary metabolites;Microbial metabolism in diverse environments;Biosynthesis of antibiotics;Carbon metabolism;Fatty acid metabolism
FTN_0870	leuS	Leucine--tRNA ligase	40	0.18	1.4E-01	0	Aminoacyl-tRNA biosynthesis
FTN_0441	ileS	Isoleucine--tRNA ligase	41	-0.02	8.6E-01	0	Aminoacyl-tRNA biosynthesis
FTN_1310	pdpB	hypothetical protein	41	0.34	1.7E-01	0	
FTN_0672	secA	Protein translocase subunit SecA	44	-0.12	2.9E-01	0	Quorum sensing;Protein export;Bacterial secretion system
FTN_1246	rne	ribonuclease E	45	-0.02	8.8E-01	0	RNA degradation
FTN_1058	tig	Trigger factor	45	-0.08	4.9E-01	0	
FTN_0266	htpG	Chaperone protein htpG	45	-0.07	5.5E-01	0	
FTN_1646	atpD	ATP synthase subunit beta	46	0.02	8.1E-01	0	Oxidative phosphorylation;Metabolic pathways
FTN_0159	rpsA	30S ribosomal protein S1	46	-0.12	3.3E-01	0	Ribosome
FTN_0660	pepA	cytosol aminopeptidase	46	0.04	6.7E-01	0	Glutathione metabolism;Metabolic pathways

FTN_1434	icd	isocitrate dehydrogenase	46	-0.02	8.6E-01	0	Citrate cycle (TCA cycle);Glutathione metabolism;Metabolic pathways;Biosynthesis of secondary metabolites;Microbial metabolism in diverse environments;Biosynthesis of antibiotics;Carbon metabolism;2-Oxocarboxylic acid metabolism;Biosynthesis of amino acids
FTN_1660	infB	Translation initiation factor IF-2	47	-0.03	7.6E-01	0	
FTN_1623	acnA	aconitate hydratase	49	0.03	7.0E-01	0	Citrate cycle (TCA cycle);Glyoxylate and dicarboxylate metabolism;Metabolic pathways;Biosynthesis of secondary metabolites;Microbial metabolism in diverse environments;Biosynthesis of antibiotics;Carbon metabolism;2-Oxocarboxylic acid metabolism;Biosynthesis of amino acids
FTN_0882	pheT	phenylalanyl-tRNA synthetase subunit beta	49	0.07	5.6E-01	0	Aminoacyl-tRNA biosynthesis
FTN_1438		fusion product of 3-hydroxacyl-CoA dehydrogenase and acyl-CoA-binding protein	49	0.46	5.4E-02	0	Fatty acid degradation;Benzoate degradation;Butanoate metabolism;Metabolic pathways;Microbial metabolism in diverse environments;Carbon metabolism;Fatty acid metabolism
FTN_1532	gdhA	glutamate dehydrogenase	50	0.03	6.4E-01	0	Arginine biosynthesis;Alanine, aspartate and glutamate metabolism;Nitrogen metabolism;Metabolic pathways;Microbial metabolism in diverse environments
FTN_1055	lon	DNA-binding, ATP-dependent protease La	50	0.01	9.4E-01	0	
FTN_0214	valS	valyl-tRNA synthetase	50	-0.25	1.6E-02	0	Aminoacyl-tRNA biosynthesis
FTN_1635	sucA	2-oxoglutarate dehydrogenase E1 component	52	-0.05	5.9E-01	0	Citrate cycle (TCA cycle);Metabolic pathways;Biosynthesis of secondary metabolites;Microbial metabolism in diverse environments;Biosynthesis of antibiotics;Carbon metabolism
FTN_1576	tuf	Elongation factor Tu	54	-0.15	2.4E-01	0	
FTN_1538	groL	60 kDa chaperonin	54	-0.03	8.2E-01	0	RNA degradation
FTN_1699	purL	phosphoribosylformylglycinamidine synthase	54	-0.06	6.4E-01	0	Purine metabolism;Metabolic pathways;Biosynthesis of secondary metabolites;Biosynthesis of antibiotics
FTN_1674	nuoG	NADH dehydrogenase subunit G	55	0.09	3.4E-01	0	Oxidative phosphorylation;Metabolic pathways
FTN_0715		hypothetical protein	59	-0.04	8.5E-01	0	
FTN_1743	clpB	chaperone clpB	59	0.18	1.7E-01	0	
FTN_0020	carB	carbamoyl phosphate synthase large subunit	59	0.17	2.7E-01	0	Pyrimidine metabolism;Alanine, aspartate and glutamate metabolism;Metabolic pathways
FTN_0164	ftsZ	cell division protein FtsZ	60	-0.06	4.5E-01	0	
FTN_0237	fusA	Elongation factor G	67	-0.09	4.0E-01	0	
FTN_1112	cphA	cyanophycin synthetase	67	0.01	9.2E-01	0	
FTN_1494	aceE	pyruvate dehydrogenase subunit E1	69	-0.02	7.9E-01	0	Glycolysis / Gluconeogenesis;Citrate cycle (TCA cycle);Pyruvate metabolism;Metabolic pathways;Biosynthesis of secondary metabolites;Microbial metabolism in diverse environments;Biosynthesis of antibiotics;Carbon metabolism
FTN_1131	putA	bifunctional proline dehydrogenase/pyrroline-5-carboxylate dehydrogenase	70	0.09	5.0E-01	0	Alanine, aspartate and glutamate metabolism;Arginine and proline metabolism;Metabolic pathways;Biosynthesis of secondary metabolites;Biosynthesis of antibiotics
FTN_0714		hypothetical protein	75	-0.05	8.4E-01	0	
FTN_1568	rpoB	DNA-directed RNA polymerase subunit beta	75	-0.01	9.4E-01	0	RNA polymerase
FTN_1567	rpoC	DNA-directed RNA polymerase subunit beta'	98	-0.03	8.0E-01	0	RNA polymerase

Supplementary Table 4. Table containing the 4,467 prokaryotic proteomes used for the bioinformatics analysis of LdcF.

Assembly number	Name of strain
000018105.1	Acaryochloris marina MBIC11017
000723785.1	Acetobacter pasteurianus 386B
001183745.1	Acetobacter pasteurianus Ab3
000010825.1	Acetobacter pasteurianus IFO 3283-01
000010945.1	Acetobacter pasteurianus IFO 3283-01-42C
000010845.1	Acetobacter pasteurianus IFO 3283-03
000010865.1	Acetobacter pasteurianus IFO 3283-07
000010965.1	Acetobacter pasteurianus IFO 3283-12
000010885.1	Acetobacter pasteurianus IFO 3283-22
000010905.1	Acetobacter pasteurianus IFO 3283-26
000010925.1	Acetobacter pasteurianus IFO 3283-32
001499615.1	Acetobacter senegalensis 108B
000247605.1	Acetobacterium woodii DSM 1030
000144695.1	Acetohalobium arabaticum DSM 5501
000967915.1	Acholeplasma brassicae
000018785.1	Acholeplasma laidlawii PG-8A
000953195.1	Acholeplasma oculi
000968055.1	Acholeplasma palmae J233
000165835.1	Achromobacter xylosoxidans A8
000758265.1	Achromobacter xylosoxidans C54
001051055.1	Achromobacter xylosoxidans MN001
000508285.1	Achromobacter xylosoxidans NBRC 15126 = ATCC 27061
001457475.1	Achromobacter xylosoxidans NCTC10807
000025305.1	Acidaminococcus fermentans DSM 20731
000230275.1	Acidaminococcus intestini RyC-MR95
000213215.1	Acidianus hospitalis W1
000144915.1	Acidilobus saccharovorans 345-15
000023265.1	Acidimicrobium ferrooxidans DSM 10331
000016725.1	Acidiphilium cryptum JF-5

000202835.1	Acidiphilium multivorum AIU301
000175575.2	Acidithiobacillus caldus ATCC 51756
000221025.1	Acidithiobacillus caldus SM-1
000214095.3	Acidithiobacillus ferrivorans SS3
000021485.1	Acidithiobacillus ferrooxidans ATCC 23270
000020825.1	Acidithiobacillus ferrooxidans ATCC 53993
000022565.1	Acidobacterium capsulatum ATCC 51196
000015025.1	Acidothermus cellulolyticus 11B
000176855.2	Acidovorax avenae subsp. avenae ATCC 19860
000015325.1	Acidovorax citrulli AAC00-1
000022305.1	Acidovorax ebreus TPSY
000015545.1	Acidovorax sp. JS42
000302535.1	Acidovorax sp. KKS102
000025665.1	Aciduliprofundum boonei T469
000327505.1	Aciduliprofundum sp. MAR08-339
000830055.1	Acinetobacter baumannii
000188215.1	Acinetobacter baumannii 1656-2
000814345.1	Acinetobacter baumannii 6200
000021245.1	Acinetobacter baumannii AB0057
000746645.1	Acinetobacter baumannii AB30
000021145.1	Acinetobacter baumannii AB307-0294
000746605.1	Acinetobacter baumannii AB31
000963815.1	Acinetobacter baumannii AB5075-UW
000695855.2	Acinetobacter baumannii AC29
000307975.2	Acinetobacter baumannii AC30
000018445.1	Acinetobacter baumannii ACICU
000015425.1	Acinetobacter baumannii ATCC 17978
001077675.1	Acinetobacter baumannii ATCC 17978-mff
000069245.1	Acinetobacter baumannii AYE
001077655.1	Acinetobacter baumannii Ab04-mff
000419385.1	Acinetobacter baumannii BJAB07104
000419405.1	Acinetobacter baumannii BJAB0715

000419425.1	Acinetobacter baumannii BJAB0868
000186665.4	Acinetobacter baumannii D1279779
001399655.1	Acinetobacter baumannii D36
000828935.1	Acinetobacter baumannii IOMTU433
000786735.1	Acinetobacter baumannii LAC-4
000187205.4	Acinetobacter baumannii MDR-TJ
000226275.1	Acinetobacter baumannii MDR-ZJ06
000828795.1	Acinetobacter baumannii NCGM 237
000761175.1	Acinetobacter baumannii Strain AbH12O-A2
000189735.2	Acinetobacter baumannii TCDC-AB0715
000302575.1	Acinetobacter baumannii TYTH-1
001026965.1	Acinetobacter baumannii XH386
000505685.2	Acinetobacter baumannii ZW85-1
001307195.1	Acinetobacter equi 114
001484935.1	Acinetobacter johnsonii XBB1
000814165.3	Acinetobacter nosocomialis 6411
000196795.1	Acinetobacter oleivorans DR1
000191145.1	Acinetobacter pittii PHEA-2
000046845.1	Acinetobacter sp. ADP1
001278715.1	Acinetobacter sp. TTH0-4
000801145.1	Actinobacillus equuli subsp. equuli
000016685.1	Actinobacillus pleuropneumoniae serovar 3 str. JL03
000015885.1	Actinobacillus pleuropneumoniae serovar 5b str. L20
000020405.1	Actinobacillus pleuropneumoniae serovar 7 str. AP76
001460855.1	Actinobacillus pleuropneumoniae serovar 8
000017245.1	Actinobacillus succinogenes 130Z
000739435.1	Actinobacillus suis ATCC 33415
001023575.1	Actinobacteria bacterium IMCC26256
001262055.1	Actinomyces meyeri W712
001278845.1	Actinomyces sp. oral taxon 414
000494755.1	Actinoplanes friuliensis DSM 7358
000284295.1	Actinoplanes missouriensis 431

000389965.1	Actinoplanes sp. N902-109
000237145.1	Actinoplanes sp. SE50/110
000023245.1	Actinosynnema mirum DSM 43827
000724605.1	Actinotignum schaalii CCUG 27420
000478885.1	Adlercreutzia equolifaciens DSM 19450
000219915.3	Advenella kashmirensis WT001
000521505.1	Advenella mimigardefordensis DPN7
000265385.1	Aequorivita sublithincola DSM 14238
000193205.1	Aerococcus urinae ACS-120-V-Col10a
000512185.1	Aeromonas hydrophila 4AK4
000963645.1	Aeromonas hydrophila AH10
000940915.1	Aeromonas hydrophila AL06-06
000633175.1	Aeromonas hydrophila AL09-71
000819505.1	Aeromonas hydrophila J-1
001455365.1	Aeromonas hydrophila JBN2301
000401555.1	Aeromonas hydrophila ML09-119
001019645.1	Aeromonas hydrophila NJ-35
000635955.1	Aeromonas hydrophila pc104A
000014805.1	Aeromonas hydrophila subsp. hydrophila ATCC 7966
000287215.3	Aeromonas media WS
000196395.1	Aeromonas salmonicida subsp. salmonicida A449
001447335.1	Aeromonas schubertii WL1483
000204115.1	Aeromonas veronii B565
000591035.1	Aeropyrum camini SY1 = JCM 12091
000011125.1	Aeropyrum pernix K1
001420915.1	Agarivorans gilvus WH0801
000241025.2	Aggregatibacter actinomycetemcomitans ANH9381
000146265.2	Aggregatibacter actinomycetemcomitans D11S-1
000163615.2	Aggregatibacter actinomycetemcomitans D7S-1
000604045.1	Aggregatibacter actinomycetemcomitans HK1651
000022985.1	Aggregatibacter aphrophilus NJ8700
001262035.1	Aggregatibacter aphrophilus W10433

000092025.1	Agrobacterium fabrum str. C58
000016265.1	Agrobacterium radiobacter K84
000192635.1	Agrobacterium sp. H13-3
000971565.1	Agrobacterium tumefaciens Ach5
000016285.1	Agrobacterium vitis S4
000020225.1	Akkermansia muciniphila ATCC BAA-835
000967305.2	Alcaligenes faecalis ZD02
000009365.1	Alcanivorax borkumensis SK2
000300005.1	Alcanivorax dieselolei B5
000299335.2	Alcanivorax pacificus W11-5
001010505.1	Alcanivorax sp. NBRC 101098
001310225.1	Algibacter alginolytica HZ-22
000166275.1	Algoriphagus machipongonensis PR1
000179015.2	Alicycliphilus denitrificans BC
000204645.1	Alicycliphilus denitrificans K601
000024285.1	Alicyclobacillus acidocaldarius subsp. acidocaldarius DSM 446
000219875.1	Alicyclobacillus acidocaldarius subsp. acidocaldarius Tc-4-1
000196495.1	Aliivibrio salmonicida LFI1238
000953695.1	Aliivibrio wodanis
000265365.1	Alistipes finegoldii DSM 17242
000014785.1	Alkalilimnicola ehrlichii MLHE-1
000016985.1	Alkaliphilus metalliredigens QYMF
000018325.1	Alkaliphilus oremlandii OhILAs
001255215.1	Alloactinosynnema sp. L-07
000025485.1	Allochromatium vinosum DSM 180
001008165.2	Altererythrobacter atlanticus 26DY36
001281485.1	Altererythrobacter epoxidivorans CGMCC 1.7731
001028625.1	Altererythrobacter marensis KCTC 22370
000934525.1	Alteromonas australica DE170
000730385.1	Alteromonas australica H 17
000172635.2	Alteromonas macleodii ATCC 27126
000300175.1	Alteromonas macleodii str. 'Balearic Sea AD45'

000299995.1	Alteromonas macleodii str. 'Black Sea 11'
000299955.1	Alteromonas macleodii str. 'English Channel 673'
000439475.1	Alteromonas mediterranea 615
000310085.1	Alteromonas mediterranea DE1
000020585.3	Alteromonas mediterranea Deep ecotype
000439495.1	Alteromonas mediterranea MED64
000439535.1	Alteromonas mediterranea U7
000439555.1	Alteromonas mediterranea U8
000439595.1	Alteromonas mediterranea UM4b
000439575.1	Alteromonas mediterranea UM7
000213655.1	Alteromonas sp. SN2
001433715.1	Alteromonas stellipolaris LMG 21856
000025885.1	Aminobacterium colombiense DSM 12261
000024605.1	Ammonifex degensii KC4
000307165.1	Amphibacillus xylanus NBRC 15112
000732925.1	Amycolatopsis japonica DSM 44213
000749465.2	Amycolatopsis lurida NRRL 2430
000454025.1	Amycolatopsis mediterranei RB
000220945.1	Amycolatopsis mediterranei S699
000196835.1	Amycolatopsis mediterranei U32
000739085.1	Amycolatopsis methanolica 239
000400635.2	Amycolatopsis orientalis HCCB10007
000214175.1	Amycolicicoccus subflavus DQS3-9A1
000317695.1	Anabaena cylindrica PCC 7122
000312705.1	Anabaena sp. 90
001277295.1	Anabaena sp. wa102
000204075.1	Anabaena variabilis ATCC 29413
000266925.1	Anaerobaculum mobile DSM 13181
000024105.1	Anaerococcus prevotii DSM 20548
000199675.1	Anaerolinea thermophila UNI-1
000022145.1	Anaeromyxobacter dehalogenans 2CP-1
000013385.1	Anaeromyxobacter dehalogenans 2CP-C

000017505.1	Anaeromyxobacter sp. Fw109-5
000020805.1	Anaeromyxobacter sp. K
000024505.1	Anaplasma centrale str. Israel
000020305.1	Anaplasma marginale str. Florida
000011945.1	Anaplasma marginale str. St. Maries
000013125.1	Anaplasma phagocytophilum str. HZ
000439755.1	Anaplasma phagocytophilum str. HZ2
000439775.1	Anaplasma phagocytophilum str. JM
000019045.1	Anoxybacillus flavithermus WK1
001187595.1	Anoxybacillus gonensis G2
000008625.1	Aquifex aeolicus VF5
000092365.1	Arcanobacterium haemolyticum DSM 20595
000008665.1	Archaeoglobus fulgidus DSM 4304
000734035.1	Archaeoglobus fulgidus DSM 8774
000025285.1	Archaeoglobus profundus DSM 5631
000385565.1	Archaeoglobus sulfaticallidus PM70-1
000194625.1	Archaeoglobus veneficus SNP6
001027285.1	Archangium gephyra DSM 2261
000215345.3	Arcobacter butzleri 7h1h
000284355.1	Arcobacter butzleri ED-1
000014025.1	Arcobacter butzleri RM4018
000092245.1	Arcobacter nitrofigilis DSM 7299
000284235.1	Arcobacter sp. L
000025965.1	Aromatoleum aromaticum EbN1
001189535.1	Arsenicicoccus sp. oral taxon 190
001445575.1	Arthrobacter alpinus ERGS4:06
001294625.1	Arthrobacter alpinus R3.8
000755585.2	Arthrobacter sp. A3
001281315.1	Arthrobacter sp. ERGS1:01
000196235.1	Arthrobacter sp. FB24
000950575.1	Arthrobacter sp. IHBB 11108
001281115.1	Arthrobacter sp. LS16

000785535.1	Arthrobacter sp. PAMC 25486
000294695.2	Arthrobacter sp. Rue61a
001454985.1	Arthrobacter sp. YC-RL1
000012225.1	Aster yellows witches'-broom phytoplasma AYWB
000175215.2	Asticcacaulis excentricus CB 48
000024225.1	Atopobium parvulum DSM 20469
001442755.1	Aureimonas sp. AU20
000061505.1	Azoarcus sp. BH72
001190925.1	Azoarcus sp. CIB
000349945.1	Azoarcus sp. KH32C
000010525.1	Azorhizobium caulinodans ORS 571
000632475.2	Azospirillum brasilense Az39
001315015.1	Azospirillum brasilense Sp7
000283655.1	Azospirillum lipoferum 4B
000010725.1	Azospirillum sp. B510
001305595.1	Azospirillum thiophilum BV-S
000817975.1	Azotobacter chroococcum NCIMB 8003
000380335.1	Azotobacter vinelandii CA
000380365.1	Azotobacter vinelandii CA6
000021045.1	Azotobacter vinelandii DJ
000494835.1	Bacillus amyloliquefaciens CC178
000196735.1	Bacillus amyloliquefaciens DSM 7
001023595.1	Bacillus amyloliquefaciens G341
000242855.2	Bacillus amyloliquefaciens IT-45
000835145.1	Bacillus amyloliquefaciens KHG19
000508265.1	Bacillus amyloliquefaciens LFB112
000833005.1	Bacillus amyloliquefaciens L-H15
000204275.1	Bacillus amyloliquefaciens LL3
000973485.1	Bacillus amyloliquefaciens L-S60
001483885.1	Bacillus amyloliquefaciens MBE1283
000195515.1	Bacillus amyloliquefaciens TA208
000221645.1	Bacillus amyloliquefaciens XH7

000262385.1	Bacillus amyloliquefaciens Y2
000742655.1	Bacillus anthracis 2000031021
000832965.1	Bacillus anthracis 2002013094
000875715.1	Bacillus anthracis A1144
000830095.1	Bacillus anthracis Ames A0462
000833065.1	Bacillus anthracis Ames_BA1004
000832665.1	Bacillus anthracis BA1015
000832725.1	Bacillus anthracis BA1035
000833125.1	Bacillus anthracis Canadian Bison
000747335.1	Bacillus anthracis Cvac02
000725325.1	Bacillus anthracis HYU01
000747375.1	Bacillus anthracis Han
000832465.1	Bacillus anthracis K3
000832505.1	Bacillus anthracis Ohio ACB
000832425.1	Bacillus anthracis PAK-1
000832585.1	Bacillus anthracis Pasteur
000832745.1	Bacillus anthracis RA3
000832565.1	Bacillus anthracis SK-102
000832445.1	Bacillus anthracis Vollum 1B
000022865.1	Bacillus anthracis str. A0248
000512835.1	Bacillus anthracis str. A16
000512775.1	Bacillus anthracis str. A16R
000007845.1	Bacillus anthracis str. Ames
000008445.1	Bacillus anthracis str. 'Ames Ancestor'
000021445.1	Bacillus anthracis str. CDC 684
000258885.1	Bacillus anthracis str. H9401
000583105.1	Bacillus anthracis str. SVA11
000008165.1	Bacillus anthracis str. Sterne
000833275.1	Bacillus anthracis str. Turkey32
000832785.1	Bacillus anthracis str. V770-NP-1R
000742895.1	Bacillus anthracis str. Vollum
000165925.1	Bacillus atrophaeus 1942

000830075.1	Bacillus atrophaeus NRS 1221A
000831065.1	Bacillus bombysepticus str. Wang
000177235.2	Bacillus cellulosilyticus DSM 2522
000022505.1	Bacillus cereus 03BB102
000832865.1	Bacillus cereus 03BB108
000789315.1	Bacillus cereus 03BB87
000832765.1	Bacillus cereus 3a
000021225.1	Bacillus cereus AH187
000021785.1	Bacillus cereus AH820
000008005.1	Bacillus cereus ATCC 10987
000007825.1	Bacillus cereus ATCC 14579
000832845.1	Bacillus cereus ATCC 4342
000021205.1	Bacillus cereus B4264
000832385.1	Bacillus cereus D17
000011625.1	Bacillus cereus E33L
000239195.1	Bacillus cereus F837/76
000832525.1	Bacillus cereus FM1
000978375.1	Bacillus cereus FORC_005
000292415.1	Bacillus cereus FRI-35
000724585.1	Bacillus cereus FT9
000832805.1	Bacillus cereus G9241
000021305.1	Bacillus cereus G9842
000283675.1	Bacillus cereus NC7401
001277915.1	Bacillus cereus NJ-W
000013065.1	Bacillus cereus Q1
000835185.1	Bacillus cereus S2-8
000143605.1	Bacillus cereus biovar anthracis str. CI
000737305.2	Bacillus clausii ENTPro
000009825.1	Bacillus clausii KSM-K16
000217835.1	Bacillus coagulans 2-6
000169195.2	Bacillus coagulans 36D1
000832905.1	Bacillus coagulans DSM 1 = ATCC 7050

000876545.1	Bacillus coagulans HM-08
001039495.1	Bacillus coagulans S-lac
000017425.1	Bacillus cytotoxicus NVH 391-98
000972245.2	Bacillus endophyticus Hbe603
000011145.1	Bacillus halodurans C-125
000473245.1	Bacillus infantis NRRL B-14911
000706725.1	Bacillus lehensis G1
000008425.1	Bacillus licheniformis DSM 13 = ATCC 14580
000025805.1	Bacillus megaterium DSM 319
000832985.1	Bacillus megaterium NBRC 15308 = ATCC 14581
001050455.1	Bacillus megaterium Q3
000025825.1	Bacillus megaterium QM B1551
000225265.1	Bacillus megaterium WSH-002
000724485.1	Bacillus methanolicus MGA3
000742855.1	Bacillus mycoides 219298
000832605.1	Bacillus mycoides ATCC 6462
000408885.1	Bacillus paralicheniformis ATCC 9945a
000876525.1	Bacillus paralicheniformis BL-09
000005825.2	Bacillus pseudofirmus OF4
000590455.1	Bacillus pumilus B6033
001191605.1	Bacillus pumilus GR-8
001431145.1	Bacillus pumilus NJ-M2
001431785.1	Bacillus pumilus NJ-V2
000017885.2	Bacillus pumilus SAFR-032
000972685.1	Bacillus pumilus W3
000093085.1	[Bacillus] selenitireducens MLS10
001050115.1	Bacillus smithii DSM 4216
000242895.3	Bacillus sp. 1NLA3E
000827045.1	Bacillus sp. BH072
000259365.1	Bacillus sp. JS
000978495.1	Bacillus sp. LM 4-2
000829195.1	Bacillus sp. OxB-1

000815145.1	Bacillus sp. Pc3
000800825.1	Bacillus sp. WP8
000747345.1	Bacillus sp. X1(2014)
000877815.1	Bacillus sp. YP1
000772125.1	Bacillus subtilis ATCC 13952
000772165.1	Bacillus subtilis ATCC 19217
000523045.1	Bacillus subtilis BEST7003
000328745.1	Bacillus subtilis BEST7613
000952895.1	Bacillus subtilis BS34A
000953615.1	Bacillus subtilis BS49
000186745.1	Bacillus subtilis BSn5
000772205.1	Bacillus subtilis Bs-916
000973605.1	Bacillus subtilis HJ5
000971925.1	Bacillus subtilis KCTC 1028
000497485.1	Bacillus subtilis PY79
000293765.1	Bacillus subtilis QB928
000782835.1	Bacillus subtilis SG6
000959025.1	Bacillus subtilis T30
001037985.1	Bacillus subtilis TO-A JPC
001015095.1	Bacillus subtilis UD1022
000338735.1	Bacillus subtilis XF-1
000209795.2	Bacillus subtilis subsp. natto BEST195
000816805.1	Bacillus subtilis subsp. spizizenii
000227465.1	Bacillus subtilis subsp. spizizenii TU-B-10
000146565.1	Bacillus subtilis subsp. spizizenii str. W23
000827065.1	Bacillus subtilis subsp. subtilis
000344745.1	Bacillus subtilis subsp. subtilis 6051-HGW
000009045.1	Bacillus subtilis subsp. subtilis str. 168
000699525.1	Bacillus subtilis subsp. subtilis str. AG1839
000349795.1	Bacillus subtilis subsp. subtilis str. BAB-1
000321395.1	Bacillus subtilis subsp. subtilis str. BSP1
000699465.1	Bacillus subtilis subsp. subtilis str. JH642

000706705.1	Bacillus subtilis subsp. subtilis str. OH 131.1
000227485.1	Bacillus subtilis subsp. subtilis str. RO-NN-1
000833085.1	Bacillus thuringiensis 97-27
000832885.1	Bacillus thuringiensis Al Hakam
000092165.1	Bacillus thuringiensis BMB171
000306745.1	Bacillus thuringiensis Bt407
001455345.1	Bacillus thuringiensis CTC
000835025.1	Bacillus thuringiensis HD1002
000832485.1	Bacillus thuringiensis HD1011
000832825.1	Bacillus thuringiensis HD571
000832925.1	Bacillus thuringiensis HD682
000292455.1	Bacillus thuringiensis HD-771
000292705.1	Bacillus thuringiensis HD-789
001182785.1	Bacillus thuringiensis HS18-1
000300475.1	Bacillus thuringiensis MC28
000774075.2	Bacillus thuringiensis XL6
000497525.2	Bacillus thuringiensis YBT-1518
001017635.1	Bacillus thuringiensis YC-10
001420855.1	Bacillus thuringiensis YWC2-8
000193355.1	Bacillus thuringiensis serovar chinensis CT-43
000190515.1	Bacillus thuringiensis serovar finitimus YBT-020
000803665.1	Bacillus thuringiensis serovar galleriae
001183785.1	Bacillus thuringiensis serovar indiana
000008505.1	[Bacillus thuringiensis] serovar konkukian str. 97-27
000717535.1	Bacillus thuringiensis serovar kurstaki str. HD-1
000338755.1	Bacillus thuringiensis serovar kurstaki str. HD73
000688795.1	Bacillus thuringiensis serovar kurstaki str. YBT-1520
000940785.1	Bacillus thuringiensis serovar morrisoni
000341665.1	Bacillus thuringiensis serovar thuringiensis str. IS5056
000015065.1	Bacillus thuringiensis str. Al Hakam
000496285.1	Bacillus toyonensis BCT-7112
000319475.1	Bacillus velezensis AS43.3

000283695.1	Bacillus velezensis CAU B946
000015785.1	Bacillus velezensis FZB42
000987825.1	Bacillus velezensis JJ-D34
000769555.1	Bacillus velezensis JS25R
000493375.1	Bacillus velezensis NAU-B3
000973585.1	Bacillus velezensis NJN-6
000685725.1	Bacillus velezensis SQR9
000583065.1	Bacillus velezensis TrigoCor1448
000455565.1	Bacillus velezensis UCMB5033
000341875.1	Bacillus velezensis UCMB5036
000455585.1	Bacillus velezensis UCMB5113
000284395.1	Bacillus velezensis YAU B9601-Y2
000988345.1	Bacillus velezensis YJ11-1-4
000018825.1	Bacillus weihenstephanensis KBAB4
000775975.1	Bacillus weihenstephanensis WSBC10204
000473305.1	Bacteroidales bacterium CF
001318345.1	Bacteroides cellulosilyticus WH2
000738045.1	Bacteroides dorei
000210835.1	Bacteroides fragilis 638R
001286525.1	Bacteroides fragilis BE1
000965785.1	Bacteroides fragilis BOB25
000025985.1	Bacteroides fragilis NCTC 9343
000009925.1	Bacteroides fragilis YCH46
000186225.1	Bacteroides helcogenes P 36-108
001314995.1	Bacteroides ovatus ATCC 8483
000190575.1	Bacteroides salanitronis DSM 18170
001314975.1	Bacteroides thetaiotaomicron 7330
000011065.1	Bacteroides thetaiotaomicron VPI-5482
000012825.1	Bacteroides vulgatus ATCC 8482
000512915.1	Barnesiella viscericola DSM 18177
001281405.1	Bartonella ancashensis 20.00
000341355.1	Bartonella australis Aust/NH1

000015445.1	Bartonella bacilliformis KC583
000253015.1	Bartonella clarridgeiae 73
000022725.1	Bartonella grahamii as4aup
000612965.1	Bartonella henselae BM1374163
000612765.1	Bartonella henselae BM1374165
001291465.1	Bartonella henselae MVT02
000046705.1	Bartonella henselae str. Houston-1
000294715.1	Bartonella quintana RM-11
000046685.1	Bartonella quintana str. Toulouse
000689355.1	Bartonella tribocorum BM1374166
000196435.1	Bartonella tribocorum CIP 105476
000341385.1	Bartonella vinsonii subsp. berkhoffii str. Winnie
000743945.1	Basilea psittacipulmonis DSM 24701
000013185.1	Baumannia cicadellinicola str. Hc (Homalodisca coagulata)
000691605.1	Bdellovibrio bacteriovorus 109J
000525675.1	Bdellovibrio bacteriovorus W
000317895.1	Bdellovibrio bacteriovorus str. Tiberius
000348725.1	Bdellovibrio exovorus JSS
001305575.1	Beggiatoa leptomitiformis D-402
000019845.1	Beijerinckia indica subsp. indica ATCC 9039
000265405.1	Belliella baltica DSM 15883
001029735.1	Berkelbacteria bacterium GW2011_GWE1_39_12
000023105.1	Beutenbergia cavernae DSM 12333
000521725.1	Bibersteinia trehalosi USDA-ARS-USMARC-188
000521745.1	Bibersteinia trehalosi USDA-ARS-USMARC-189
000521765.1	Bibersteinia trehalosi USDA-ARS-USMARC-190
000347595.1	Bibersteinia trehalosi USDA-ARS-USMARC-192
001263395.1	Bifidobacterium actinocoloniiforme DSM 22766
000737885.1	Bifidobacterium adolescentis 22L
000010425.1	Bifidobacterium adolescentis ATCC 15703
000817995.1	Bifidobacterium adolescentis BB23
001025155.1	Bifidobacterium angulatum DSM 20098 = JCM 7096

000817045.1	Bifidobacterium animalis
000695895.1	Bifidobacterium animalis RH
000260715.1	Bifidobacterium animalis subsp. animalis ATCC 25527
000818055.1	Bifidobacterium animalis subsp. lactis
000021425.1	Bifidobacterium animalis subsp. lactis AD011
000471945.1	Bifidobacterium animalis subsp. lactis ATCC 27673
000277325.1	Bifidobacterium animalis subsp. lactis B420
000025245.1	Bifidobacterium animalis subsp. lactis BB-12
000224965.2	Bifidobacterium animalis subsp. lactis BLC1
000277345.1	Bifidobacterium animalis subsp. lactis Bi-07
000022705.1	Bifidobacterium animalis subsp. lactis Bl-04
000414215.1	Bifidobacterium animalis subsp. lactis Bl12
000220885.1	Bifidobacterium animalis subsp. lactis CNCM I-2494
000022965.1	Bifidobacterium animalis subsp. lactis DSM 10140
000816205.1	Bifidobacterium animalis subsp. lactis KLDS2.0603
000092765.1	Bifidobacterium animalis subsp. lactis V9
000304215.1	Bifidobacterium asteroides PRL2011
001025135.1	Bifidobacterium bifidum ATCC 29521 = JCM 1255 = DSM 20456
001281345.1	Bifidobacterium bifidum BF3
000265095.1	Bifidobacterium bifidum BGN4
000165905.1	Bifidobacterium bifidum PRL2010
000164965.1	Bifidobacterium bifidum S17
000568955.1	Bifidobacterium breve 12L
000569055.1	Bifidobacterium breve 689b
000213865.1	Bifidobacterium breve ACS-071-V-Sch8b
001281425.1	Bifidobacterium breve BR3
001025175.1	Bifidobacterium breve DSM 20213 = JCM 1192
000568975.1	Bifidobacterium breve JCM 7017
000569015.1	Bifidobacterium breve JCM 7019
000569035.1	Bifidobacterium breve NCFB 2258
000569075.1	Bifidobacterium breve S27
000220135.1	Bifidobacterium breve UCC2003

001025195.1	Bifidobacterium catenulatum DSM 16992 = JCM 1194 = LMG 11043
000737865.1	Bifidobacterium coryneforme LMG18911
000024445.1	Bifidobacterium dentium Bd1
001042595.1	Bifidobacterium dentium JCM 1195 = DSM 20436
000706765.1	Bifidobacterium indicum LMG 11587 = DSM 20214
001042615.1	Bifidobacterium kashiwanohense JCM 15439 = DSM 21854
000800455.1	Bifidobacterium kashiwanohense PV20-2
000829295.1	Bifidobacterium longum 105-A
001293145.1	Bifidobacterium longum BG7
000730205.1	Bifidobacterium longum BXY01
000008945.1	Bifidobacterium longum DJO10A
000007525.1	Bifidobacterium longum NCC2705
001281305.1	Bifidobacterium longum subsp. infantis
000196575.1	Bifidobacterium longum subsp. infantis 157F
000020425.1	Bifidobacterium longum subsp. infantis ATCC 15697 = JCM 1222 = DSM 20088
001446255.1	Bifidobacterium longum subsp. longum
000166315.1	Bifidobacterium longum subsp. longum BBMN68
000772485.1	Bifidobacterium longum subsp. longum GT15
000196555.1	Bifidobacterium longum subsp. longum JCM 1217
000092325.1	Bifidobacterium longum subsp. longum JDM301
000219455.1	Bifidobacterium longum subsp. longum KACC 91563
001025215.1	Bifidobacterium pseudocatenulatum DSM 20438 = JCM 1200 = LMG 10505
000800475.1	Bifidobacterium pseudolongum PV8-2
001042635.1	Bifidobacterium scardovii JCM 12489 = DSM 13734
000347695.1	Bifidobacterium thermophilum RBL67
001459775.1	Blastochloris viridis
001402875.1	Blastochloris viridis ATCC 19567
000284015.1	Blastococcus saxobsidens DD2
000262715.1	Blattabacterium sp. (Blaberus giganteus)
000334405.1	Blattabacterium sp. (Blatta orientalis) str. Tarazona
000022605.2	Blattabacterium sp. (Blattella germanica) str. Bge
000236405.1	Blattabacterium sp. (Cryptocercus punctulatus) str. Cpu

000233435.1	Blattabacterium sp. (Mastotermes darwiniensis) str. MADAR
000471965.1	Blattabacterium sp. (Nauphoeta cinerea)
000348805.1	Blattabacterium sp. (Panesthia angustipennis spadica) str. BPAA
000093165.1	Blattabacterium sp. (Periplaneta americana) str. BPLAN
000973545.1	Blochmannia endosymbiont of Camponotus (Colobopsis) obliquus
000973505.1	Blochmannia endosymbiont of Polyrhachis (Hedomyrma) turneri
000070465.1	Bordetella avium 197N
000318015.1	Bordetella bronchiseptica 253
000317955.1	Bordetella bronchiseptica MO149
000829175.1	Bordetella bronchiseptica S798
001078275.1	Bordetella hinzii F582
001078295.1	Bordetella hinzii H568
000765395.1	Bordetella holmesii 44057
000612485.1	Bordetella holmesii ATCC 51541
000317935.1	Bordetella parapertussis Bpp5
000812165.1	Bordetella pertussis 137
000306945.1	Bordetella pertussis 18323
001307585.1	Bordetella pertussis B1838
001307605.1	Bordetella pertussis B1865
000193595.3	Bordetella pertussis B1917
001307625.1	Bordetella pertussis B3405
001307645.1	Bordetella pertussis B3582
001307665.1	Bordetella pertussis B3585
001307565.1	Bordetella pertussis B3621
001307525.1	Bordetella pertussis B3629
001307685.1	Bordetella pertussis B3640
001307705.1	Bordetella pertussis B3658
001307725.1	Bordetella pertussis B3913
001307745.1	Bordetella pertussis B3921
000212975.1	Bordetella pertussis CS
001013565.1	Bordetella pertussis D420
000067205.1	Bordetella petrii DSM 12804

000304735.1	Borrelia afzelii HLJ01
000962775.1	Borrelia afzelii K78
000222835.1	Borrelia afzelii PKo
000741005.1	Borrelia afzelii Tom3107
000008685.2	Borrelia burgdorferi B31
000444465.1	Borrelia burgdorferi CA382
000166655.2	Borrelia burgdorferi JD1
000166635.2	Borrelia burgdorferi N40
000021405.1	Borrelia burgdorferi ZS7
000019685.1	Borrelia duttonii Ly
000239475.1	Borrelia garinii BgVir
000300045.1	Borrelia garinii NMJW1
000691545.1	Borrelia garinii SZ
000956315.1	Borrelia hermsii CC1
000807295.1	Borrelia miyamotoi CT14D4
000445425.4	Borrelia miyamotoi LB-2001
000512145.2	Borrelia parkeri HR1
000019705.1	Borrelia recurrentis A1
000012085.1	Borrelia turicatae 91E135
000196215.1	Borreliella bavariensis PBi
000222305.1	Borreliella bissettii DN127
000808095.1	Borreliella chilensis VA1
000739475.1	Borreliella valaisiana Tom4006
000170955.2	Borreliella valaisiana VS116
000023405.1	Brachybacterium faecium DSM 4810
000022105.1	Brachyspira hyodysenteriae WA1
000223215.1	Brachyspira intermedia PWS/A
000092845.1	Brachyspira murdochii DSM 12563
000143725.1	Brachyspira pilosicoli 95/1000
000296575.1	Brachyspira pilosicoli B2904
000325665.1	Brachyspira pilosicoli P43/6/78
000011365.1	Bradyrhizobium diazoefficiens USDA 110

000807315.1	Bradyrhizobium japonicum E109
000284375.1	Bradyrhizobium japonicum USDA 6
000344805.1	Bradyrhizobium oligotrophicum S58
000015165.1	Bradyrhizobium sp. BTAi1
000026145.1	Bradyrhizobium sp. ORS 278
000284275.1	Bradyrhizobium sp. S23321
000010165.1	Brevibacillus brevis NBRC 100599
000219535.3	Brevibacillus laterosporus LMG 15441
000987865.1	[Brevibacterium] flavum ATCC 15168
001310255.1	Brevundimonas sp. DS20
000144605.1	Brevundimonas subvibrioides ATCC 15264
001296965.1	Brucella abortus 104M
000054005.1	Brucella abortus 2308
000740295.1	Brucella abortus 63 75
000238175.1	Brucella abortus A13334
001043295.1	Brucella abortus BAB8416
000740135.1	Brucella abortus BDW
000740155.1	Brucella abortus BER
000740315.1	Brucella abortus BFY
000740175.1	Brucella abortus NCTC 10505
000018725.1	Brucella abortus S19
000008145.1	Brucella abortus bv. 1 str. 9-941
000740375.1	Brucella abortus bv. 2 str. 86/8/59
000740215.1	Brucella abortus bv. 6 str. 870
000740195.1	Brucella abortus bv. 9 str. C68
000018525.1	Brucella canis ATCC 23365
000238195.1	Brucella canis HSK A52141
000740335.1	Brucella canis RM6/66
000691585.1	Brucella canis SVA13
000590795.1	Brucella ceti TE10759-12
000590815.1	Brucella ceti TE28753-12
001431745.1	Brucella melitensis 20236

000022625.1	Brucella melitensis ATCC 23457
000192725.1	Brucella melitensis M28
000192885.1	Brucella melitensis M5-90
000227645.1	Brucella melitensis NI
000007125.1	Brucella melitensis bv. 1 str. 16M
000740355.1	Brucella melitensis bv. 3 str. Ether
000022745.1	Brucella microti CCM 4915
000016845.1	Brucella ovis ATCC 25840
000740275.1	Brucella pinnipedialis 6/566
000221005.1	Brucella pinnipedialis B2/94
000007505.1	Brucella suis 1330
000740235.1	Brucella suis 513UK
000018905.1	Brucella suis ATCC 23445
000740435.1	Brucella suis BSP
000875695.1	Brucella suis Human/AR/US/1981
000236255.1	Brucella suis VBI22
000755105.1	Brucella suis ZW043
000755085.1	Brucella suis ZW046
000600055.1	Brucella suis bv. 1 str. S2
000698245.1	Brucella suis bv. 2
000740255.1	Brucella suis bv. 3 str. 686
001280225.1	Buchnera aphidicola (Aphis glycines)
000090965.1	Buchnera aphidicola BCc
000217635.1	Buchnera aphidicola (Cinara tujafilina)
000021085.1	Buchnera aphidicola str. 5A (Acyrthosiphon pisum)
000009605.1	Buchnera aphidicola str. APS (Acyrthosiphon pisum)
000225445.1	Buchnera aphidicola str. Ak (Acyrthosiphon kondoi)
000007725.1	Buchnera aphidicola str. Bp (Baizongia pistaciae)
000521585.1	Buchnera aphidicola str. F009 (Myzus persicae)
000521565.1	Buchnera aphidicola str. G002 (Myzus persicae)
000183305.1	Buchnera aphidicola str. JF98 (Acyrthosiphon pisum)
000183285.1	Buchnera aphidicola str. JF99 (Acyrthosiphon pisum)

000183225.1	Buchnera aphidicola str. LL01 (Acyrthosiphon pisum)
000174075.1	Buchnera aphidicola str. LSR1 (Acyrthosiphon pisum)
000007365.1	Buchnera aphidicola str. Sg (Schizaphis graminum)
000183245.1	Buchnera aphidicola str. TLW03 (Acyrthosiphon pisum)
000021065.1	Buchnera aphidicola str. Tuc7 (Acyrthosiphon pisum)
000521525.1	Buchnera aphidicola str. USDA (Myzus persicae)
000225465.1	Buchnera aphidicola str. Ua (Uroleucon ambrosiae)
000521545.1	Buchnera aphidicola str. W106 (Myzus persicae)
000203915.1	Burkholderia ambifaria AMMD
000019925.1	Burkholderia ambifaria MC40-6
000014085.1	Burkholderia cenocepacia AU 1054
000755725.1	Burkholderia cenocepacia DDS 22E-1
000764955.1	Burkholderia cenocepacia DWS 37E-2
000236215.4	Burkholderia cenocepacia H111
000203955.1	Burkholderia cenocepacia HI2424
000009485.1	Burkholderia cenocepacia J2315
000019505.1	Burkholderia cenocepacia MC0-3
001484665.1	Burkholderia cenocepacia ST32
001411495.1	Burkholderia cepacia ATCC 25416
000755805.1	Burkholderia cepacia DDS 7H-2
000292915.1	Burkholderia cepacia GG4
000974835.1	Burkholderia cepacia LO6
001029145.1	Burkholderia contaminans MS14
000959505.1	Burkholderia dolosa AU0158
000959725.1	Burkholderia gladioli ATCC 10248
000194745.1	Burkholderia gladioli BSR3
000022645.2	Burkholderia glumae BGR1
000960995.1	Burkholderia glumae LMG 2196 = ATCC 33617
000835205.1	Burkholderia glumae PG1
000012945.1	Burkholderia lata 383
000959405.1	Burkholderia mallei 11
000756025.2	Burkholderia mallei 2000031063

000959625.1	Burkholderia mallei 2002721276
000959165.1	Burkholderia mallei 2002734299
000959485.1	Burkholderia mallei 2002734306
000755865.1	Burkholderia mallei 23344
000755845.1	Burkholderia mallei 6
000011705.1	Burkholderia mallei ATCC 23344
000755885.1	Burkholderia mallei BMQ
000755785.1	Burkholderia mallei FMH 23344
000959465.1	Burkholderia mallei India86-567-2
000015605.1	Burkholderia mallei NCTC 10229
000015625.1	Burkholderia mallei NCTC 10247
000015465.1	Burkholderia mallei SAVP1
000018505.1	Burkholderia multivorans ATCC 17616
000959525.1	Burkholderia multivorans ATCC BAA-247
000756005.1	Burkholderia multivorans DDS 15A-1
000959365.1	Burkholderia oklahomensis C6786
000755985.1	Burkholderia oklahomensis EO147
001411805.1	Burkholderia plantarii ATCC 43733
000260515.1	Burkholderia pseudomallei 1026b
000015925.1	Burkholderia pseudomallei 1106a
000012785.1	Burkholderia pseudomallei 1710b
000959145.1	Burkholderia pseudomallei 406e
000756185.1	Burkholderia pseudomallei 576
000015905.1	Burkholderia pseudomallei 668
000959265.1	Burkholderia pseudomallei 7894
001277975.1	Burkholderia pseudomallei 982
000770535.1	Burkholderia pseudomallei A79A
000770455.1	Burkholderia pseudomallei B03
000755765.1	Burkholderia pseudomallei BDP
000756085.1	Burkholderia pseudomallei BGR
000294635.1	Burkholderia pseudomallei BPC006
000755825.1	Burkholderia pseudomallei BSR

001318245.1	Burkholderia pseudomallei Bp1651
000953095.1	Burkholderia pseudomallei C1
000755925.1	Burkholderia pseudomallei HBPUB10134a
000755905.1	Burkholderia pseudomallei HBPUB10303a
000770515.1	Burkholderia pseudomallei K42
000011545.1	Burkholderia pseudomallei K96243
000521645.1	Burkholderia pseudomallei MSHR146
000756165.1	Burkholderia pseudomallei MSHR1655
000959225.1	Burkholderia pseudomallei MSHR2543
000439695.1	Burkholderia pseudomallei MSHR305
000959205.1	Burkholderia pseudomallei MSHR491
000520895.1	Burkholderia pseudomallei MSHR511
000583835.1	Burkholderia pseudomallei MSHR520
000755965.1	Burkholderia pseudomallei MSHR5848
000756065.1	Burkholderia pseudomallei MSHR5855
000755945.1	Burkholderia pseudomallei MSHR5858
000770395.1	Burkholderia pseudomallei MSHR62
000959305.1	Burkholderia pseudomallei MSHR668
000959185.1	Burkholderia pseudomallei MSHR840
000756125.1	Burkholderia pseudomallei Mahidol-1106a
000511915.1	Burkholderia pseudomallei NAU20B-16
000764575.1	Burkholderia pseudomallei NAU35A-3
000511895.1	Burkholderia pseudomallei NCTC 13178
000494855.1	Burkholderia pseudomallei NCTC 13179
000959345.1	Burkholderia pseudomallei PB08298010
000757015.2	Burkholderia pseudomallei PHLS 112
000757035.2	Burkholderia pseudomallei Pasteur 52237
000770565.1	Burkholderia pseudomallei TSV202
000770495.1	Burkholderia pseudomallei TSV 48
000954175.1	Burkholderia pseudomallei vgh07
001277875.1	Burkholderia pseudomallei vgh16R
001277895.1	Burkholderia pseudomallei vgh16W

001028665.1	Burkholderia pyrrocinia DSM 10685
000959325.1	Burkholderia sp. 2002721687
000176935.2	Burkholderia sp. CCGE1001
000092885.1	Burkholderia sp. CCGE1002
000148685.1	Burkholderia sp. CCGE1003
001293045.1	Burkholderia sp. HB1
000262695.1	Burkholderia sp. KJ006
000402035.1	Burkholderia sp. RPE64
000828875.1	Burkholderia sp. RPE67
000236065.1	Burkholderia sp. YI23
000959425.1	Burkholderia thailandensis 2002721643
000567925.1	Burkholderia thailandensis 2002721723
000808035.2	Burkholderia thailandensis 2003015869
000959605.1	Burkholderia thailandensis 34
000765375.1	Burkholderia thailandensis E254
000012365.1	Burkholderia thailandensis E264
000567945.1	Burkholderia thailandensis E444
000567905.1	Burkholderia thailandensis H0587
000385525.1	Burkholderia thailandensis MSMB121
000764595.1	Burkholderia thailandensis MSMB59
000706745.1	Burkholderia thailandensis USAMRU Malaysia #20
000959245.1	Burkholderia ubonensis MSMB22
000016205.1	Burkholderia vietnamiensis G4
000959445.1	Burkholderia vietnamiensis LMG 10929
000828975.1	Burkholderiales bacterium GJ-E10
000145035.1	Butyrivibrio proteoclasticus B316
000007085.1	Caldanaerobacter subterraneus subsp. tengcongensis MB4
000022325.1	Caldicellulosiruptor bescii DSM 6725
000166355.1	Caldicellulosiruptor hydrothermalis 108
000166695.1	Caldicellulosiruptor kristjanssonii I77R1B
000166775.1	Caldicellulosiruptor kronotskyensis 2002
000193435.3	Caldicellulosiruptor lactoaceticus 6A

000145215.1	Caldicellulosiruptor obsidiansis OB47
000166335.1	Caldicellulosiruptor owensensis OL
000016545.1	Caldicellulosiruptor saccharolyticus DSM 8903
000281175.1	Caldilinea aerophila DSM 14535 = NBRC 104270
000284335.1	Caldisericum exile AZM16c01
000317795.1	Caldisphaera lagunensis DSM 15908
000183405.1	Calditerrivibrio nitroreducens DSM 19672
000018305.1	Caldivirga maquilingensis IC-167
000734895.2	Calothrix sp. 336/3
000317435.1	Calothrix sp. PCC 6303
000316575.1	Calothrix sp. PCC 7507
000494775.1	Campylobacter coli 15-537360
001305715.1	Campylobacter coli BFR-CA-9557
000465235.1	Campylobacter coli CVM N29710
000954195.1	Campylobacter coli FB1
001417635.1	Campylobacter coli HC2-48
001483845.1	Campylobacter coli OR12
000583755.1	Campylobacter coli RM1875
000583775.1	Campylobacter coli RM4661
000583795.1	Campylobacter coli RM5611
000017725.1	Campylobacter concisus 13826
001298465.1	Campylobacter concisus ATCC 33237
000017465.1	Campylobacter curvus 525.92
000759485.1	Campylobacter fetus subsp. fetus 04/554
000015085.1	Campylobacter fetus subsp. fetus 82-40
000814265.1	Campylobacter fetus subsp. testudinum
000495505.1	Campylobacter fetus subsp. testudinum 03-427
001484645.1	Campylobacter fetus subsp. testudinum Sp3
000759515.1	Campylobacter fetus subsp. venerealis 97/608
000512745.1	Campylobacter fetus subsp. venerealis cfvi03/293
000967135.1	Campylobacter fetus subsp. venerealis str. 84-112
001190745.1	Campylobacter gracilis ATCC 33236

000017585.1	Campylobacter hominis ATCC BAA-381
000736415.1	Campylobacter iguaniorum 1485E
001483985.1	Campylobacter iguaniorum 2463D
000816185.1	Campylobacter insulaenigrae NCTC 12927
000430385.1	Campylobacter jejuni 32488
000493495.1	Campylobacter jejuni 4031
001506205.1	Campylobacter jejuni CJ677CC002
001507085.1	Campylobacter jejuni CJ677CC008
001506325.1	Campylobacter jejuni CJ677CC010
001507265.1	Campylobacter jejuni CJ677CC012
001507005.1	Campylobacter jejuni CJ677CC013
001506725.1	Campylobacter jejuni CJ677CC014
001506385.1	Campylobacter jejuni CJ677CC016
001507125.1	Campylobacter jejuni CJ677CC024
001507185.1	Campylobacter jejuni CJ677CC026
001506585.1	Campylobacter jejuni CJ677CC032
001506605.1	Campylobacter jejuni CJ677CC033
001507205.1	Campylobacter jejuni CJ677CC034
001506345.1	Campylobacter jejuni CJ677CC036
001506745.1	Campylobacter jejuni CJ677CC039
001506885.1	Campylobacter jejuni CJ677CC040
001506405.1	Campylobacter jejuni CJ677CC041
001506965.1	Campylobacter jejuni CJ677CC047
001506785.1	Campylobacter jejuni CJ677CC052
001506985.1	Campylobacter jejuni CJ677CC058
001506565.1	Campylobacter jejuni CJ677CC059
001506905.1	Campylobacter jejuni CJ677CC061
001506545.1	Campylobacter jejuni CJ677CC062
001507145.1	Campylobacter jejuni CJ677CC064
001506265.1	Campylobacter jejuni CJ677CC073
001506825.1	Campylobacter jejuni CJ677CC078
001506765.1	Campylobacter jejuni CJ677CC085

001507225.1	Campylobacter jejuni CJ677CC086
001506445.1	Campylobacter jejuni CJ677CC092
001507065.1	Campylobacter jejuni CJ677CC094
001507245.1	Campylobacter jejuni CJ677CC095
001507025.1	Campylobacter jejuni CJ677CC100
001506185.1	Campylobacter jejuni CJ677CC519
001506705.1	Campylobacter jejuni CJ677CC520
001506285.1	Campylobacter jejuni CJ677CC521
001507045.1	Campylobacter jejuni CJ677CC522
001506845.1	Campylobacter jejuni CJ677CC523
001506365.1	Campylobacter jejuni CJ677CC524
001507165.1	Campylobacter jejuni CJ677CC525
001506305.1	Campylobacter jejuni CJ677CC526
001506805.1	Campylobacter jejuni CJ677CC527
001506665.1	Campylobacter jejuni CJ677CC528
001506505.1	Campylobacter jejuni CJ677CC529
001506465.1	Campylobacter jejuni CJ677CC530
001506525.1	Campylobacter jejuni CJ677CC531
001506485.1	Campylobacter jejuni CJ677CC532
001506945.1	Campylobacter jejuni CJ677CC533
001506225.1	Campylobacter jejuni CJ677CC534
001506425.1	Campylobacter jejuni CJ677CC535
001506245.1	Campylobacter jejuni CJ677CC536
001506625.1	Campylobacter jejuni CJ677CC537
001506685.1	Campylobacter jejuni CJ677CC538
001506925.1	Campylobacter jejuni CJ677CC539
001506865.1	Campylobacter jejuni CJ677CC540
001507105.1	Campylobacter jejuni CJ677CC541
001506645.1	Campylobacter jejuni CJ677CC542
001412295.1	Campylobacter jejuni CJM1cam
001457695.1	Campylobacter jejuni NCTC11351
000011865.1	Campylobacter jejuni RM1221

001314285.1	Campylobacter jejuni RM1285
000017485.1	Campylobacter jejuni subsp. doylei 269.97
000737085.1	Campylobacter jejuni subsp. jejuni
000468915.2	Campylobacter jejuni subsp. jejuni 00-2425
000466105.2	Campylobacter jejuni subsp. jejuni 00-2426
000466065.2	Campylobacter jejuni subsp. jejuni 00-2538
000466075.2	Campylobacter jejuni subsp. jejuni 00-2544
000017905.1	Campylobacter jejuni subsp. jejuni 81116
000015525.1	Campylobacter jejuni subsp. jejuni 81-176
000171795.2	Campylobacter jejuni subsp. jejuni CG8421
000772225.1	Campylobacter jejuni subsp. jejuni F38011
000025425.1	Campylobacter jejuni subsp. jejuni IA3902
000184085.1	Campylobacter jejuni subsp. jejuni ICDCCJ07001
000148705.1	Campylobacter jejuni subsp. jejuni M1
000304375.1	Campylobacter jejuni subsp. jejuni NCTC 11168-BN148
000830865.1	Campylobacter jejuni subsp. jejuni NCTC 11168-GSv
000830775.1	Campylobacter jejuni subsp. jejuni NCTC 11168-K12E5
000830805.1	Campylobacter jejuni subsp. jejuni NCTC 11168-Kf1
000009085.1	Campylobacter jejuni subsp. jejuni NCTC 11168 = ATCC 700819
000830825.1	Campylobacter jejuni subsp. jejuni NCTC 11168-mcK12E5
000830845.1	Campylobacter jejuni subsp. jejuni NCTC 11168-mfK12E5
000302555.3	Campylobacter jejuni subsp. jejuni PT14
000632435.1	Campylobacter jejuni subsp. jejuni R14
000184205.1	Campylobacter jejuni subsp. jejuni S3
000816385.1	Campylobacter lari CCUG 22395
000816365.1	Campylobacter lari NCTC 11845
000816405.1	Campylobacter lari RM16701
000816425.1	Campylobacter lari RM16712
000019205.1	Campylobacter lari RM2100
001017575.1	Campylobacter lari Slaughter Beach
000816225.1	Campylobacter lari subsp. concheus LMG 11760
000816785.1	Campylobacter peloridis LMG 23910

000816245.1	Campylobacter sp. RM16704
000816265.1	Campylobacter subantarcticus LMG 24374
000816305.1	Campylobacter subantarcticus LMG 24377
001190755.1	Campylobacter ureolyticus RIGS 9880
000816345.1	Campylobacter volucris LMG 24379
000024165.1	Candidatus Accumulibacter phosphatis clade IIA str. UW-1
000020565.1	Candidatus Amoebophilus asiaticus 5a2
000270205.1	Candidatus Arthromitus sp. SFB-mouse-Japan
000709435.1	Candidatus Arthromitus sp. SFB-mouse-NL
000284435.1	Candidatus Arthromitus sp. SFB-mouse-Yit
000283555.1	Candidatus Arthromitus sp. SFB-rat-Yit
000025125.1	Candidatus Atelocyanobacterium thalassa isolate ALOHA
000010645.1	Candidatus Azobacteroides pseudotrichonymphae genomovar. CFP2
000513475.1	Candidatus Babela massiliensis
000754265.1	Candidatus Baumannia cicadellinicola
001029675.1	Candidatus Beckwithbacteria bacterium GW2011_GWC1_49_16
000331065.1	Candidatus Blochmannia chromaiodes str. 640
000043285.1	Candidatus Blochmannia floridanus
000011745.1	Candidatus Blochmannia pennsylvanicus str. BPEN
000185985.2	Candidatus Blochmannia vafer str. BVAF
000743035.1	Candidatus Caedibacter acanthamoebae
001029775.1	Candidatus Campbellbacteria bacterium GW2011_OD1_34_28
001274515.1	Candidatus Carsonella ruddii
000287235.1	Candidatus Carsonella ruddii CE isolate Thao2000
000287255.1	Candidatus Carsonella ruddii CS isolate Thao2000
000441575.1	Candidatus Carsonella ruddii DC
000287275.1	Candidatus Carsonella ruddii HC isolate Thao2000
000287295.1	Candidatus Carsonella ruddii HT isolate Thao2000
000287315.1	Candidatus Carsonella ruddii PC isolate NHV
000010365.1	Candidatus Carsonella ruddii PV
000146065.1	Candidatus Cloacimonas acidaminovorans str. Evry
000018425.1	Candidatus Desulforudis audaxviator MP104C

000319385.1	Candidatus Endolissoclinum faulkneri L2
000510265.1	Candidatus Endolissoclinum faulkneri L5
000953435.1	Candidatus Evansia muelleri
000981565.1	Candidatus Filomicrobium marinum
000582535.1	Candidatus Hepatoplasma crinochetorum Av
000699475.1	Candidatus Hodgkinia cicadicola
000021505.1	Candidatus Hodgkinia cicadicola Dsem
000828515.1	Candidatus Ishikawaella capsulata Mpkobe
000340925.1	Candidatus Kinetoplastibacterium blastocrithidii TCC012E
000319245.1	Candidatus Kinetoplastibacterium blastocrithidii (ex Strigomonas culicis)
000340825.1	Candidatus Kinetoplastibacterium crithidii TCC036E
000319225.1	Candidatus Kinetoplastibacterium crithidii (ex Angomonas deanei ATCC 30255)
000340795.1	Candidatus Kinetoplastibacterium desouzaii TCC079E
000340905.1	Candidatus Kinetoplastibacterium galatii TCC219
000340865.1	Candidatus Kinetoplastibacterium oncopeltii TCC290E
000019605.1	Candidatus Korarchaeum cryptofilum OPF8
000014005.1	Candidatus Koribacter versatilis Ellin345
001021085.1	Candidatus Liberibacter africanus PTSAPSY
000496595.1	Candidatus Liberibacter americanus str. Sao Paulo
000590865.2	Candidatus Liberibacter asiaticus
000829355.1	Candidatus Liberibacter asiaticus str. Ishi-1
000346595.1	Candidatus Liberibacter asiaticus str. gxpsy
000023765.2	Candidatus Liberibacter asiaticus str. psy62
000183665.1	Candidatus Liberibacter solanacearum CLso-ZC1
000404225.1	Candidatus Methanomassiliicoccus intestinalis Issoire-Mx1
000300255.2	Candidatus Methanomethylophilus alvus Mx1201
000800805.1	Candidatus Methanoplasma termitum
000091165.1	Candidatus Methylomirabilis oxyfera
000981505.1	Candidatus Methylopumilus planktonicus
000953015.1	Candidatus Methylopumilus turicensis
000219355.1	Candidatus Midichloria mitochondrii IricVA
000219175.1	Candidatus Moranella endobia PCIT

000364725.1	Candidatus Moranella endobia PCVAL
000770195.1	Candidatus Mycoplasma girerdii
000281235.1	Candidatus Mycoplasma haemolamae str. Purdue
001447885.1	Candidatus Nasuia deltocephalinicola
000442605.1	Candidatus Nasuia deltocephalinicola str. NAS-ALF
000812185.1	Candidatus Nitrosopelagicus brevis
000956175.1	Candidatus Nitrosopumilus adriaticus
000299365.1	Candidatus Nitrosopumilus koreensis AR1
000875775.1	Candidatus Nitrosopumilus piranensis
000299395.1	Candidatus Nitrosopumilus sp. AR2
000730285.1	Candidatus Nitrososphaera evergladensis SR1
000303155.1	Candidatus Nitrososphaera gargensis Ga9.2
000955905.3	Candidatus Nitrosotenuis cloacae
001458695.1	Candidatus Nitrospira inopinata
001029715.1	Candidatus Pacebacteria bacterium GW2011_OP11-3_36_13
000478905.1	Candidatus Pantoea carbekii
000742835.1	Candidatus Paracaedibacter acanthamoebae
000195085.1	Candidatus Pelagibacter sp. IMCC9063
000012345.1	Candidatus Pelagibacter ubique HTCC1062
001430755.1	Candidatus Peribacter riflensis
000069925.1	Candidatus Phytoplasma australiense
000026205.1	Candidatus Phytoplasma mali
000953295.1	Candidatus Portiera aleyrodidarum
000292685.1	Candidatus Portiera aleyrodidarum BT-B-HRs
000298385.1	Candidatus Portiera aleyrodidarum BT-QVLC
000827855.1	Candidatus Portiera aleyrodidarum MED (Bemisia tabaci)
000349745.1	Candidatus Portiera aleyrodidarum TV
000441555.1	Candidatus Profftella armatura
000024465.1	Candidatus Puniceispirillum marinum IMCC1322
001273795.1	Candidatus Rickettsia amblyommii
000284055.1	Candidatus Rickettsia amblyommii str. GAT-30V
000093065.1	Candidatus Riesia pediculicola USDA

000015105.1	Candidatus Ruthia magnifica str. Cm (Calyptogena magnifica)
001029695.1	Candidatus Saccharibacteria bacterium GW2011_GWC2_44_17
000803625.1	Candidatus Saccharibacteria oral taxon TM7x
000392435.1	Candidatus Saccharimonas aalborgensis
000517405.1	Candidatus Sodalis pierantonius str. SOPE
000014905.1	Candidatus Solibacter usitatus Ellin6076
000754305.1	Candidatus Sulcia muelleri
000147035.1	Candidatus Sulcia muelleri CARI
000025785.1	Candidatus Sulcia muelleri DMIN
000017525.1	Candidatus Sulcia muelleri GWSS
000829155.1	Candidatus Sulcia muelleri PSPU
000022945.1	Candidatus Sulcia muelleri SMDSEM
000442635.1	Candidatus Sulcia muelleri str. Sulcia-ALF
000477435.1	Candidatus Symbiobacter mobilis CR
000828815.1	Candidatus Tachikawaea gelatinosa
001447805.1	Candidatus Tenderia electrophaga
001293165.1	Candidatus Thioglobus autotrophica
001281385.1	Candidatus Thioglobus singularis PS1
000412755.1	Candidatus Tremblaya phenacola PAVE
000219195.1	Candidatus Tremblaya princeps PCIT
000220965.1	Candidatus Tremblaya princeps PCVAL
000331975.1	Candidatus Uzinura diaspidicola str. ASNER
000010405.1	Candidatus Vesicomyosocius okutanii HA
001029755.1	Candidatus Woesebacteria bacterium GW2011_GWF1_31_35
001029635.1	Candidatus Wolfebacteria bacterium GW2011_GWB1_47_1
001318295.1	Candidatus Xiphinematobacter sp. Idaho Grape
000147015.1	Candidatus Zinderia insecticola CARI
000220625.1	Capnocytophaga canimorsus Cc5
000023285.1	Capnocytophaga ochracea DSM 7271
001278825.1	Capnocytophaga sp. oral taxon 323
000012865.1	Carboxydothermus hydrogenoformans Z-2901
000304455.1	Cardinium endosymbiont cEper1 of Encarsia pergandiella

000493735.1	Carnobacterium inhibens subsp. gilichinskyi
000317975.2	Carnobacterium maltaromaticum LMA28
000195575.1	Carnobacterium sp. 17-4
001483965.1	Carnobacterium sp. CP1
000612685.1	Castellaniella defragrans 65Phen
000024025.1	Catenulispora acidiphila DSM 44928
000006905.1	Caulobacter crescentus CB15
000022005.1	Caulobacter crescentus NA1000
001414055.1	Caulobacter henricii CB4
000092285.1	Caulobacter segnis ATCC 21756
000019145.1	Caulobacter sp. K31
000758305.1	Cedecea neteri M006
001506165.1	Cedecea neteri ND02
000758325.1	Cedecea neteri ND14a
000758345.1	Cedecea neteri ND14b
000757825.1	Cedecea neteri SSMD04
000819565.1	Celeribacter indicus P73
001308265.1	Celeribacter marinus IMCC 12053
000212695.1	Cellulomonas fimi ATCC 484
000092865.1	Cellulomonas flavigena DSM 20109
000218545.1	Cellulomonas gilvus ATCC 13127
000186265.1	Cellulophaga algicola DSM 14237
000468615.2	Cellulophaga baltica 18
000477035.2	Cellulophaga baltica NN016038
000190595.1	Cellulophaga lytica DSM 7489
000750195.1	Cellulophaga lytica HI1
000019225.1	Cellvibrio japonicus Ueda107
000520015.2	Chania multitudinisentens RB-25
000014245.1	Chelativorans sp. BNC1
001271345.1	Chelatococcus sp. CO-6
000024005.1	Chitinophaga pinensis DSM 2588
000952935.1	Chlamydia abortus AB7

000026025.1	Chlamydia abortus S26/3
000583875.1	Chlamydia avium 10DC88
000767405.1	Chlamydia muridarum Nigg3
000006685.1	Chlamydia muridarum str. Nigg
000772145.1	Chlamydia muridarum str. Nigg3
000830945.1	Chlamydia muridarum str. Nigg3 CMUT3-5
000709455.1	Chlamydia muridarum str. Nigg 2 MCR
000830965.1	Chlamydia muridarum str. Nigg CM972
000470825.1	Chlamydia pecorum P787
000470765.1	Chlamydia pecorum PV3056/3
000470805.1	Chlamydia pecorum W73
001007025.1	Chlamydia pneumoniae
000270405.1	Chlamydia psittaci 01DC11
000270425.1	Chlamydia psittaci 02DC15
000270445.1	Chlamydia psittaci 08DC60
000191925.1	Chlamydia psittaci 6BC
000298375.2	Chlamydia psittaci 84/55
000270385.1	Chlamydia psittaci C19/98
000298535.2	Chlamydia psittaci CP3
000298415.1	Chlamydia psittaci GR9
000298495.2	Chlamydia psittaci M56
000298435.2	Chlamydia psittaci MN
000338695.1	Chlamydia psittaci Mat116
000298555.2	Chlamydia psittaci NJ1
000298455.2	Chlamydia psittaci VS225
000298515.2	Chlamydia psittaci WC
000298475.2	Chlamydia psittaci WS/RT/E30
000590575.1	Chlamydia trachomatis
000068585.1	Chlamydia trachomatis 434/Bu
000173475.1	Chlamydia trachomatis 6276
000173495.1	Chlamydia trachomatis 6276s
000173515.1	Chlamydia trachomatis 70

000173535.1	Chlamydia trachomatis 70s
000226605.1	Chlamydia trachomatis A2497
000318525.2	Chlamydia trachomatis A/363
000318565.1	Chlamydia trachomatis A/7249
000012125.1	Chlamydia trachomatis A/HAR-13
000026925.1	Chlamydia trachomatis B/Jali20/OT
000026905.1	Chlamydia trachomatis B/TZ1A828/OT
000507225.1	Chlamydia trachomatis C/TW-3
001183765.1	Chlamydia trachomatis D/CS637/11
000092485.1	Chlamydia trachomatis D-EC
000093005.1	Chlamydia trachomatis D-LC
000008725.1	Chlamydia trachomatis D/UW-3/CX
000174055.1	Chlamydia trachomatis D(s)2923
000092745.1	Chlamydia trachomatis E/11023
000092665.1	Chlamydia trachomatis E/150
000318645.1	Chlamydia trachomatis E/Bour
001183805.1	Chlamydia trachomatis E/CS1025/11
000304495.1	Chlamydia trachomatis E/SW3
000590635.1	Chlamydia trachomatis F/11-96
001183825.1	Chlamydia trachomatis F/CS847/08
000304515.1	Chlamydia trachomatis F/SW4
000304535.1	Chlamydia trachomatis F/SW5
000092725.1	Chlamydia trachomatis G/11074
000092705.1	Chlamydia trachomatis G/11222
000092805.1	Chlamydia trachomatis G/9301
000092685.1	Chlamydia trachomatis G/9768
001183845.1	Chlamydia trachomatis Ia/CS190/96
000318765.1	Chlamydia trachomatis Ia/SotonIa1
000318785.1	Chlamydia trachomatis Ia/SotonIa3
000441775.1	Chlamydia trachomatis J/6276tet1
000318845.1	Chlamydia trachomatis L1/115
000318865.1	Chlamydia trachomatis L1/224

000318825.1	Chlamydia trachomatis L1/440/LN
000318885.1	Chlamydia trachomatis L2/25667R
000364785.1	Chlamydia trachomatis L2/434/Bu(f)
000364765.1	Chlamydia trachomatis L2/434/Bu(i)
000318985.1	Chlamydia trachomatis L2b/795
000318905.1	Chlamydia trachomatis L2b/8200/07
000971705.1	Chlamydia trachomatis L2b/CS19/08
000971725.1	Chlamydia trachomatis L2b/CS784/08
000068525.2	Chlamydia trachomatis L2b/UCH-1/proctitis
000318925.1	Chlamydia trachomatis L2b/UCH-2
000220105.1	Chlamydia trachomatis L2c
000175515.1	Chlamydia trachomatis L2tet1
000319105.1	Chlamydia trachomatis L3/404/LN
000441585.1	Chlamydia trachomatis RC-F/69
000441715.1	Chlamydia trachomatis RC-F(s)/342
000441635.1	Chlamydia trachomatis RC-F(s)/852
000441655.1	Chlamydia trachomatis RC-J/943
000441675.1	Chlamydia trachomatis RC-J/953
000441755.1	Chlamydia trachomatis RC-J/966
000441795.1	Chlamydia trachomatis RC-J/971
000441735.1	Chlamydia trachomatis RC-J(s)/122
000441815.1	Chlamydia trachomatis RC-L2/55
000441695.1	Chlamydia trachomatis RC-L2(s)/3
000441615.1	Chlamydia trachomatis RC-L2(s)/46
000210495.1	Chlamydia trachomatis Sweden2
000007605.1	Chlamydophila caviae GPIC
000009945.1	Chlamydophila felis Fe/C-56
000204135.1	Chlamydophila pecorum E58
000091085.1	Chlamydophila pneumoniae AR39
000008745.1	Chlamydophila pneumoniae CWL029
000011165.1	Chlamydophila pneumoniae J138
000024145.1	Chlamydophila pneumoniae LPCoLN

000007205.1	Chlamydophila pneumoniae TW-183
000226295.1	Chloracidobacterium thermophilum B
000020505.1	Chlorobaculum parvum NCIB 8327
000012585.1	Chlorobium chlorochromatii CaD3
000020465.1	Chlorobium limicola DSM 245
000012485.1	Chlorobium luteolum DSM 273
000020545.1	Chlorobium phaeobacteroides BS1
000015125.1	Chlorobium phaeobacteroides DSM 266
000016085.1	Chlorobium phaeovibrioides DSM 265
000006985.1	Chlorobium tepidum TLS
000021945.1	Chloroflexus aggregans DSM 9485
000018865.1	Chloroflexus aurantiacus J-10-fl
000022185.1	Chloroflexus sp. Y-400-fl
000020525.1	Chloroherpeton thalassium ATCC 35110
001189295.1	Chondromyces crocatus Cm c5
000007705.1	Chromobacterium violaceum ATCC 12472
000055785.1	Chromohalobacter salexigens DSM 3043
000317125.1	Chroococcidiopsis thermalis PCC 7203
001021975.1	Chryseobacterium gallinarum DSM 27622
001456155.1	Chryseobacterium sp. IHB B 17019
000829375.1	Chryseobacterium sp. StRB126
000981805.1	Citrobacter amalonaticus Y19
001022155.1	Citrobacter freundii CAV1321
001022275.1	Citrobacter freundii CAV1741
000648515.1	Citrobacter freundii CFNIH1
001281005.1	Citrobacter freundii P10159
000018045.1	Citrobacter koseri ATCC BAA-895
000027085.1	Citrobacter rodentium ICC168
001304795.1	Citromicrobium sp. JL477
001280205.1	Clavibacter michiganensis PF008
000958465.1	Clavibacter michiganensis subsp. insidiosus
000063485.1	Clavibacter michiganensis subsp. michiganensis NCPPB 382

000355695.1	Clavibacter michiganensis subsp. nebraskensis NCPPB 2581
000069225.1	Clavibacter michiganensis subsp. sepedonicus
001042715.1	Clostridium aceticum DSM 1496
000008765.1	Clostridium acetobutylicum ATCC 824
000218855.1	Clostridium acetobutylicum DSM 1731
000191905.1	Clostridium acetobutylicum EA 2018
000299355.1	[Clostridium] acidurici 9a
000484505.1	Clostridium autoethanogenum DSM 10061
000789395.1	Clostridium baratii str. Sullivan
000767745.1	Clostridium beijerinckii ATCC 35702
000016965.1	Clostridium beijerinckii NCIMB 8052
000577895.1	Clostridium bornimense M2/40
000827935.1	Clostridium botulinum
000829015.1	Clostridium botulinum 111
000789355.1	Clostridium botulinum 202F
000022765.1	Clostridium botulinum A2 str. Kyoto
000019545.1	Clostridium botulinum A3 str. Loch Maree
000017025.1	Clostridium botulinum A str. ATCC 19397
000063585.1	Clostridium botulinum A str. ATCC 3502
000017045.1	Clostridium botulinum A str. Hall
000019305.1	Clostridium botulinum B1 str. Okra
000204565.1	Clostridium botulinum BKT015925
000020165.1	Clostridium botulinum B str. Eklund 17B (NRP)
000020345.1	Clostridium botulinum Ba4 str. 657
000817935.1	Clostridium botulinum CDC_1436
000816945.1	Clostridium botulinum CDC_297
000020285.1	Clostridium botulinum E3 str. Alaska E43
000092345.1	Clostridium botulinum F str. 230613
000017065.1	Clostridium botulinum F str. Langeland
000253195.1	Clostridium botulinum H04402 065
000827955.1	Clostridium botulinum NCTC 8550
001465175.1	Clostridium butyricum JKY6D1

001456065.2	Clostridium butyricum KNU-L09
001038625.1	Clostridium carboxidivorans P7
000022065.1	[Clostridium] cellulolyticum H10
000953215.1	[Clostridium] cellulosi
000145275.1	Clostridium cellulovorans 743B
000237085.1	[Clostridium] clariflavum DSM 19732
000016505.1	Clostridium kluyveri DSM 555
000010265.1	Clostridium kluyveri NBRC 12016
000178835.2	Clostridium lentocellum DSM 5427
000143685.1	Clostridium ljungdahlii DSM 13528
000014125.1	Clostridium novyi NT
000389635.1	Clostridium pasteurianum BC1
000807175.1	Clostridium pasteurianum DSM 525 = ATCC 6013
000506785.2	Clostridium pasteurianum NRRL B-598
000013285.1	Clostridium perfringens ATCC 13124
001304735.1	Clostridium perfringens FORC_003
000013845.1	Clostridium perfringens SM101
000009685.1	Clostridium perfringens str. 13
000473995.1	Clostridium saccharobutylicum DSM 13864
000144625.1	[Clostridium] saccharolyticum WM1
000340885.1	Clostridium saccharoperbutylacetonicum N1-4(HMT)
000968375.1	Clostridium scatologenes ATCC 25775
000244875.1	Clostridium sp. BNL1100
000270305.1	Clostridium sp. SY8519
001020205.1	Clostridium sporogenes DSM 795
000973705.1	Clostridium sporogenes NCIMB 10696
000331995.1	[Clostridium] stercorarium subsp. stercorarium DSM 8532
000196455.1	[Clostridium] sticklandii DSM 519
000967115.1	Clostridium tetani 12124569
000007625.1	Clostridium tetani E88
000786695.1	Collimonas arenae Cal35
000221045.1	Collimonas fungivorans Ter331

000012325.1	Colwellia psychrerythraea 34H
001444365.1	Colwellia sp. MT41
000828895.1	Comamonadaceae bacterium A1
000828915.1	Comamonadaceae bacterium B1
000093145.2	Comamonas testosteroni CNB-2
001406795.1	Comamonas testosteroni P19
000739375.1	Comamonas testosteroni TK102
000025265.1	Conexibacter woesei DSM 14684
001305615.1	Confluentimicrobium sp. EMB200-NS6
000020945.1	Coprothermobacter proteolyticus DSM 5265
000025905.1	Coraliomargarita akajimensis DSM 45221
000255295.1	Corallococcus coralloides DSM 2259
000814825.1	Coriobacteriaceae bacterium 68-1-3
000195315.1	Coriobacterium glomerans PW2
000590555.1	Corynebacterium argentoratense DSM 44202
000732945.1	Corynebacterium atypicum R2070
000022905.1	Corynebacterium aurimucosum ATCC 700975
000344785.1	Corynebacterium callunae DSM 20147
000980815.1	Corynebacterium camporealensis DSM 44610
000550785.1	Corynebacterium casei LMG S-19264
001277995.1	Corynebacterium deserti GIMN1.010
000241895.1	Corynebacterium diphtheriae 241
000241875.1	Corynebacterium diphtheriae 31A
000241935.1	Corynebacterium diphtheriae BH8
000255175.1	Corynebacterium diphtheriae C7 (beta)
000255215.1	Corynebacterium diphtheriae CDCE 8392
000255235.1	Corynebacterium diphtheriae HC01
000255155.1	Corynebacterium diphtheriae HC02
000242775.1	Corynebacterium diphtheriae HC03
000255195.1	Corynebacterium diphtheriae HC04
000241915.1	Corynebacterium diphtheriae INCA 402
001457455.1	Corynebacterium diphtheriae NCTC11397

000255275.1	Corynebacterium diphtheriae PW8
000255255.1	Corynebacterium diphtheriae VA01
000767055.1	Corynebacterium doosanense CAU 212 = DSM 45436
000011305.1	Corynebacterium efficiens YS-314
001021025.1	Corynebacterium epidermidicanis DSM 45586
000525655.1	Corynebacterium falsenii DSM 44353
000742735.1	Corynebacterium glutamicum AR1
000011325.1	Corynebacterium glutamicum ATCC 13032
000742715.1	Corynebacterium glutamicum ATCC 21831
000828015.1	Corynebacterium glutamicum B253
001447865.1	Corynebacterium glutamicum CP
000382905.1	Corynebacterium glutamicum K051
000445015.1	Corynebacterium glutamicum MB001
000010225.1	Corynebacterium glutamicum R
000404145.1	Corynebacterium glutamicum SCgG1
000404185.1	Corynebacterium glutamicum SCgG2
000626675.1	Corynebacterium glyciniphilum AJ 3170
000341345.1	Corynebacterium halotolerans YIM 70093 = DSM 44683
000819445.1	Corynebacterium humireducens NBRC 106098 = DSM 45392
000739455.1	Corynebacterium imitans DSM 44264
000006605.1	Corynebacterium jeikeium K411
000023145.1	Corynebacterium kroppenstedtii DSM 44385
000980835.1	Corynebacterium kutscheri DSM 20755
001274895.1	Corynebacterium lactis RW2-5
000835165.1	Corynebacterium marinum DSM 44953
000442645.1	Corynebacterium maris DSM 45190
001020985.1	Corynebacterium mustelae DSM 45274
000144935.2	Corynebacterium pseudotuberculosis 1002
001433475.1	Corynebacterium pseudotuberculosis 1002B
000233735.1	Corynebacterium pseudotuberculosis 1/06-A
001017615.1	Corynebacterium pseudotuberculosis 12C
000972805.1	Corynebacterium pseudotuberculosis 226

000263755.2	Corynebacterium pseudotuberculosis 258
001047215.1	Corynebacterium pseudotuberculosis 262
000258385.1	Corynebacterium pseudotuberculosis 267
001026945.1	Corynebacterium pseudotuberculosis 29156
000259155.2	Corynebacterium pseudotuberculosis 31
000248375.1	Corynebacterium pseudotuberculosis 316
000241855.1	Corynebacterium pseudotuberculosis 3/99-5
000227175.1	Corynebacterium pseudotuberculosis 42/02-A
000730365.1	Corynebacterium pseudotuberculosis 48252
000144675.1	Corynebacterium pseudotuberculosis C231
000227605.1	Corynebacterium pseudotuberculosis CIP 52.97
000730405.1	Corynebacterium pseudotuberculosis CS_10
000265545.1	Corynebacterium pseudotuberculosis Cp162
001186445.1	Corynebacterium pseudotuberculosis E19
001481755.1	Corynebacterium pseudotuberculosis E56
000143705.1	Corynebacterium pseudotuberculosis FRC41
000730445.1	Corynebacterium pseudotuberculosis Ft_2193/67
000152065.1	Corynebacterium pseudotuberculosis I19
001481675.1	Corynebacterium pseudotuberculosis MEX25
001447295.1	Corynebacterium pseudotuberculosis N1
000255935.1	Corynebacterium pseudotuberculosis P54B96
001456175.1	Corynebacterium pseudotuberculosis PA01
000221625.1	Corynebacterium pseudotuberculosis PAT10
001481715.1	Corynebacterium pseudotuberculosis PO222/4-1
001298505.1	Corynebacterium pseudotuberculosis PO269-5
000814865.1	Corynebacterium pseudotuberculosis VD57
000177535.2	Corynebacterium resistens DSM 45100
000833575.1	Corynebacterium singulare IBS B52218
000755185.1	Corynebacterium sp. ATCC 6931
000418365.1	Corynebacterium terpenotabidum Y-11
001021045.1	Corynebacterium testudinoris DSM 44614
000968945.1	Corynebacterium ulcerans

000306825.1	Corynebacterium ulcerans 0102
000769635.1	Corynebacterium ulcerans 05146
001281445.1	Corynebacterium ulcerans 131001
000767645.1	Corynebacterium ulcerans 210931
000767415.1	Corynebacterium ulcerans 210932
000215645.1	Corynebacterium ulcerans 809
000215665.1	Corynebacterium ulcerans BR-AD22
000767685.1	Corynebacterium ulcerans FRC11
000499805.2	Corynebacterium ulcerans FRC58
000069945.1	Corynebacterium urealyticum DSM 7109
000338095.1	Corynebacterium urealyticum DSM 7111
000747315.1	Corynebacterium ureicelerivorans IMMIB RIV-2301
001021065.1	Corynebacterium uterequi DSM 45634
000179395.2	Corynebacterium variabile DSM 44702
000550805.1	Corynebacterium vitaeruminis DSM 20294
000019865.1	Coxiella burnetii CbuG_Q212
000019885.1	Coxiella burnetii CbuK_Q154
000017105.1	Coxiella burnetii Dugway 5J108-111
000018745.1	Coxiella burnetii RSA 331
000007765.1	Coxiella burnetii RSA 493
001077715.1	Coxiella-like endosymbiont CRt
000815025.1	Coxiella-like endosymbiont candidatus Clenella amblyommii
000317495.1	Crinalium epipsammum PCC 9333
000196315.1	Croceibacter atlanticus HTCC2559
001028705.1	Croceicoccus naphthovorans PQ-2
001277255.1	Cronobacter condimenti 1330
001277235.1	Cronobacter dublinensis subsp. dublinensis LMG 23823
001277195.1	Cronobacter muytjensii ATCC 51329
000982825.1	Cronobacter sakazakii ATCC 29544
000017665.1	Cronobacter sakazakii ATCC BAA-894
000504545.1	Cronobacter sakazakii CMCC 45402
000263215.1	Cronobacter sakazakii ES15

001277275.1	Cronobacter sakazakii NCTC 8155
000339015.1	Cronobacter sakazakii SP291
000027065.2	Cronobacter turicensis z3032
001277175.1	Cronobacter universalis NCTC 9529
000023845.1	Cryptobacterium curtum DSM 15641
000832305.1	Cupriavidus basilensis 4G11
001281465.1	Cupriavidus gilardii CR3
000196015.1	Cupriavidus metallidurans CH34
000219215.1	Cupriavidus necator N-1
000317675.1	Cyanobacterium aponinum PCC 10605
000317655.1	Cyanobacterium stanieri PCC 7202
000316515.1	Cyanobium gracile PCC 6307
000017845.1	Cyanothece sp. ATCC 51142
000021825.1	Cyanothece sp. PCC 7424
000022045.1	Cyanothece sp. PCC 7425
000147335.1	Cyanothece sp. PCC 7822
000021805.1	Cyanothece sp. PCC 8801
000024045.1	Cyanothece sp. PCC 8802
001050135.1	Cyclobacterium amurskyense KCTC 12363
000222485.1	Cyclobacterium marinum DSM 745
000299965.1	Cycloclasticus sp. P1
000442595.1	Cycloclasticus zancles 78-ME
000014145.1	Cytophaga hutchinsonii ATCC 33406
000317615.1	Dactylococcopsis salina PCC 8305
000012425.1	Dechloromonas aromatica RCB
000236665.1	Dechlorosoma suillum PS
000010985.1	Deferribacter desulfuricans SSM1
000953715.1	Defluviitoga tunisiensis
000512895.1	Dehalobacter restrictus DSM 9455
000305815.1	Dehalobacter sp. CF
000305775.1	Dehalobacter sp. DCA
000011905.1	Dehalococcoides mccartyi 195

000016705.1	Dehalococcoides mccartyi BAV1
000341695.1	Dehalococcoides mccartyi BTF08
000009025.1	Dehalococcoides mccartyi CBDB1
000830925.1	Dehalococcoides mccartyi CG1
000830905.1	Dehalococcoides mccartyi CG4
000830885.1	Dehalococcoides mccartyi CG5
000341655.1	Dehalococcoides mccartyi DCMB5
000025585.1	Dehalococcoides mccartyi GT
000499365.1	Dehalococcoides mccartyi GY50
000025025.1	Dehalococcoides mccartyi VS
001010485.1	Dehalococcoides sp. UCH007
000143165.1	Dehalogenimonas lykanthroporepellens BL-DC-9
001005265.1	Dehalogenimonas sp. WBC-2
000020685.1	Deinococcus deserti VCD115
000196275.1	Deinococcus geothermalis DSM 11300
000252445.1	Deinococcus gobiensis I-0
000186385.1	Deinococcus maricopensis DSM 21211
000317835.1	Deinococcus peraridilitoris DSM 19664
000190555.1	Deinococcus proteolyticus MRP
000008565.1	Deinococcus radiodurans R1
001007995.1	'Deinococcus soli' Cha et al. 2014
000800395.1	Deinococcus swuensis DY59
000018665.1	Delftia acidovorans SPH-1
000214395.1	Delftia sp. Cs1-4
000025725.1	Denitrovibrio acetiphilus DSM 12809
000725405.1	Dermacoccus nishinomiyaensis M25
000143965.1	Desulfarculus baarsii DSM 2075
000021905.1	Desulfatibacillum alkenivorans AK-01
000243155.3	Desulfitobacterium dehalogenans ATCC 51507
000243135.3	Desulfitobacterium dichloroeliminans LMG P-21439
000021925.1	Desulfitobacterium hafniense DCB-2
000010045.1	Desulfitobacterium hafniense Y51

000231405.3	Desulfitobacterium metallireducens DSM 15288
000195295.1	Desulfobacca acetoxidans DSM 11109
000020365.1	Desulfobacterium autotrophicum HRM2
000307105.1	Desulfobacula toluolica Tol2
000186885.1	Desulfobulbus propionicus DSM 2032
000341395.1	Desulfocapsa sulfexigens DSM 10523
000018405.1	Desulfococcus oleovorans Hxd3
000024325.1	Desulfohalobium retbaense DSM 5692
000023225.1	Desulfomicrobium baculatum DSM 4028
000266945.1	Desulfomonile tiedjei DSM 6799
000255115.3	Desulfosporosinus acidiphilus SJ4
000231385.3	Desulfosporosinus meridiei DSM 13257
000235605.1	Desulfosporosinus orientis DSM 765
000025945.1	Desulfotalea psychrophila LSv54
000024205.1	Desulfotomaculum acetoxidans DSM 771
000233715.3	Desulfotomaculum gibsoniae DSM 7213
000214705.1	Desulfotomaculum kuznetsovii DSM 6115
000214435.1	Desulfotomaculum nigrificans CO-1-SRB
000016165.1	Desulfotomaculum reducens MI-1
000215085.1	Desulfotomaculum ruminis DSM 2154
000176915.2	Desulfovibrio aespoeensis Aspo-2
000212675.2	Desulfovibrio africanus str. Walvis Bay
000012665.1	Desulfovibrio alaskensis G20
000189295.2	Desulfovibrio desulfuricans ND132
000022125.1	Desulfovibrio desulfuricans subsp. desulfuricans str. ATCC 27774
000010665.1	Desulfovibrio magneticus RS-1
000023445.1	Desulfovibrio salexigens DSM 2638
000015485.1	Desulfovibrio vulgaris DP4
000166115.1	Desulfovibrio vulgaris RCH1
000195755.1	Desulfovibrio vulgaris str. Hildenborough
000021385.1	Desulfovibrio vulgaris str. 'Miyazaki F'
000517565.1	Desulfurella acetivorans A63

000177635.2	Desulfurispirillum indicum S5
000092205.1	Desulfurivibrio alkaliphilus AHT 2
000191045.1	Desulfurobacterium thermolithotrophum DSM 11699
000231015.3	Desulfurococcus fermentans DSM 16532
000020905.1	Desulfurococcus kamchatkensis 1221n
000186365.1	Desulfurococcus mucosus DSM 2162
001278055.1	Desulfuromonas sp. WTL
001402915.1	Devosia sp. A16
001185205.1	Devosia sp. H5989
001260615.2	Devriesea agamarum IMP2
000015345.1	Dichelobacter nodosus VCS1703A
000023565.1	Dickeya chrysanthemi Ech1591
000147055.1	Dickeya dadantii 3937
000023545.1	Dickeya paradisiaca Ech703
000816045.1	Dickeya zeae EC1
000025065.1	Dickeya zeae Ech586
000020965.1	Dictyoglomus thermophilum H-6-12
000021645.1	Dictyoglomus turgidum DSM 6724
000018145.1	Dinoroseobacter shibae DFL 12 = DSM 16493
000212355.1	Dokdonia sp. 4H-3-7-5
000152925.3	Dokdonia sp. MED134
000626635.1	Draconibacterium orientale Draconibacterium orientale type strain FH5T
000023125.1	Dyadobacter fermentans DSM 18053
000725385.1	Dyella japonica A8
000632805.1	Dyella jiangningensis SBZ 3-12
000325705.1	Echinicola vietnamensis DSM 17526
000264765.2	Edwardsiella anguillarum ET080813
000022885.2	Edwardsiella ictaluri 93-146
000348565.1	Edwardsiella piscicida C07-087
000800725.2	Edwardsiella sp. EA181011
001186215.1	Edwardsiella sp. LADL05-105
000020865.1	Edwardsiella tarda EIB202

000146305.1	Edwardsiella tarda FL6-60
001011055.1	Edwardsiella tarda FL95-01
000024265.1	Eggerthella lenta DSM 2243
000270285.1	Eggerthella sp. YY7918
000012565.1	Ehrlichia canis str. Jake
000013145.1	Ehrlichia chaffeensis str. Arkansas
000632815.1	Ehrlichia chaffeensis str. Heartland
000632865.1	Ehrlichia chaffeensis str. Jax
000632885.1	Ehrlichia chaffeensis str. Liberty
000632905.1	Ehrlichia chaffeensis str. Osceola
000632925.1	Ehrlichia chaffeensis str. Saint Vincent
000632945.1	Ehrlichia chaffeensis str. Wakulla
000632965.1	Ehrlichia chaffeensis str. West Paces
000508225.1	Ehrlichia muris AS145
000050405.1	Ehrlichia ruminantium str. Gardel
000026005.1	Ehrlichia ruminantium str. Welgevonden
000632845.1	Ehrlichia sp. HF
000495935.2	Elizabethkingia anophelis NUHP1
001011675.1	Elizabethkingia meningoseptica FMS-007
000955665.1	Elizabethkingia sp. BM10
000020145.1	Elusimicrobium minutum Pei191
000263195.1	Emticicia oligotrophica DSM 17448
001027545.1	Endomicrobium proavitum Rsa215
001021995.1	[Enterobacter] aerogenes CAV1320
000334515.1	Enterobacter aerogenes EA1509E
000215745.1	Enterobacter aerogenes KCTC 2190
000807415.4	Enterobacter asburiae 35734
001022095.1	Enterobacter asburiae CAV1043
000632395.1	Enterobacter asburiae L1
000224675.1	Enterobacter asburiae LF7a
000814225.1	Enterobacter cloacae 34399
000814125.3	Enterobacter cloacae 34977

000807405.4	Enterobacter cloacae 34978
000807425.4	Enterobacter cloacae 34998
001022015.1	Enterobacter cloacae CAV1311
001022075.1	Enterobacter cloacae CAV1411
001022055.1	Enterobacter cloacae CAV1668
001022255.1	Enterobacter cloacae CAV1669
000724505.1	Enterobacter cloacae ECNIH2
000750225.1	Enterobacter cloacae ECNIH3
000784865.1	Enterobacter cloacae ECNIH4
000784905.1	Enterobacter cloacae ECNIH5
000750275.1	Enterobacter cloacae ECR091
000239975.1	Enterobacter cloacae EcWSU1
000770155.1	Enterobacter cloacae GGT036
000512375.1	Enterobacter cloacae P101
001029645.1	Enterobacter cloacae UW5
000025565.1	Enterobacter cloacae subsp. cloacae ATCC 13047
000286275.1	Enterobacter cloacae subsp. cloacae ENHKU01
000235765.3	Enterobacter cloacae subsp. dissolvens SDM
000164865.1	Enterobacter lignolyticus SCF1
000814205.1	Enterobacter sp. 34983
000016325.1	Enterobacter sp. 638
000801755.2	Enterobacter sp. E20
000410515.1	Enterobacter sp. R4-368
000330845.1	Enterobacteriaceae bacterium strain FGI 57
000157355.2	Enterococcus casseliflavus EC20
001267865.1	Enterococcus durans KLDS 6.0930
001267395.1	Enterococcus durans KLDS 6.0933
000211255.1	Enterococcus faecalis 62
000742975.1	Enterococcus faecalis ATCC 29212
000281195.1	Enterococcus faecalis D32
000550745.1	Enterococcus faecalis DENG1
000172575.2	Enterococcus faecalis OG1RF

000007785.1	Enterococcus faecalis V583
000317915.1	Enterococcus faecalis str. Symbioflor 1
001298485.1	Enterococcus faecium 64/3
000250945.1	Enterococcus faecium Aus0004
000444405.1	Enterococcus faecium Aus0085
000174395.2	Enterococcus faecium DO
000336405.1	Enterococcus faecium NRRL B-2354
000737555.1	Enterococcus faecium T110
001412695.1	Enterococcus faecium UW7606x64/3 TC1
000271405.2	Enterococcus hirae ATCC 9790
000504125.1	Enterococcus mundtii QU 25
001465115.1	Enterococcus silesiacus LMG 23085
000027205.1	Erwinia amylovora ATCC 49946
000091565.1	Erwinia amylovora CFBP1430
000513415.1	Erwinia amylovora LA635
000513395.1	Erwinia amylovora LA636
000513355.1	Erwinia amylovora LA637
000196615.1	Erwinia billingiae Eb661
000027265.1	Erwinia pyrifoliae Ep1/96
000165815.1	Erwinia sp. Ejp617
000026185.1	Erwinia tasmaniensis Et1/99
000404205.1	Erysipelothrix rhusiopathiae SY1027
000270085.1	Erysipelothrix rhusiopathiae str. Fujisawa
001077815.1	Erythrobacter atlanticus s21-N3
000013005.1	Erythrobacter litoralis HTCC2594
000512125.1	Escherichia albertii KF1
000801185.2	Escherichia coli
000027125.1	Escherichia coli 042
000829985.1	Escherichia coli 1303
001420955.1	Escherichia coli 2009C-3133
001420935.1	Escherichia coli 2012C-4227
000013305.1	Escherichia coli 536

000026245.1	Escherichia coli 55989
000814145.2	Escherichia coli 6409
000819645.1	Escherichia coli 789
000148365.1	Escherichia coli ABU 83972
001051135.1	Escherichia coli ACN001
000813165.1	Escherichia coli APEC IMT5155
000014845.1	Escherichia coli APEC O1
000332755.1	Escherichia coli APEC O78
000743255.1	Escherichia coli ATCC 25922
000019385.1	Escherichia coli ATCC 8739
000725265.1	Escherichia coli B7A
000009565.2	Escherichia coli BL21(DE3)
000023665.1	Escherichia coli 'BL21-Gold(DE3)pLysS AG'
000833145.1	Escherichia coli BL21 (TaKaRa)
000750555.1	Escherichia coli BW25113
000022345.1	Escherichia coli BW2952
000017985.1	Escherichia coli B str. REL606
000830035.1	Escherichia coli C41(DE3)
001039415.1	Escherichia coli C43(DE3)
001007915.1	Escherichia coli CFSAN029787
000007445.1	Escherichia coli CFT073
000971615.1	Escherichia coli CI5
001455385.1	Escherichia coli CQSW20
000023365.1	Escherichia coli DH1
001183645.1	Escherichia coli DH1Ec095
001183665.1	Escherichia coli DH1Ec104
001183685.1	Escherichia coli DH1Ec169
000831565.1	Escherichia coli ECC-1470
000784925.1	Escherichia coli ECONIH1
000800215.1	Escherichia coli ER2796
000210475.1	Escherichia coli ETEC H10407
001276585.1	Escherichia coli HB101

000017765.1	Escherichia coli HS
000026265.1	Escherichia coli IAI1
000026345.1	Escherichia coli IAI39
000025745.1	Escherichia coli IHE3034
000493755.1	Escherichia coli JJ1886
000800765.1	Escherichia coli K-12
000953515.1	Escherichia coli K-12 substr. HMS174
000952955.1	Escherichia coli K-12 substr. RV308
000725305.1	Escherichia coli KLY
000147855.3	Escherichia coli KO11FL
000284495.1	Escherichia coli LF82
000468515.1	Escherichia coli LY180
000931565.1	Escherichia coli MNCRE44
000214765.2	Escherichia coli NA114
001043215.1	Escherichia coli NCM3722
000714595.1	Escherichia coli Nissle 1917
000010745.1	Escherichia coli O103:H2 str. 12009
000299255.1	Escherichia coli O104:H4 str. 2009EL-2050
000299475.1	Escherichia coli O104:H4 str. 2009EL-2071
000299455.1	Escherichia coli O104:H4 str. 2011C-3493
000986765.1	Escherichia coli O104:H4 str. C227-11
000010765.1	Escherichia coli O111:H- str. 11128
000026545.1	Escherichia coli O127:H6 str. E2348/69
000017745.1	Escherichia coli O139:H28 str. E24377A
000671295.1	Escherichia coli O145:H28 str. RM12581
000662395.1	Escherichia coli O145:H28 str. RM12761
000520035.1	Escherichia coli O145:H28 str. RM13514
000520055.1	Escherichia coli O145:H28 str. RM13516
000827105.1	Escherichia coli O157:H16
001307215.1	Escherichia coli O157:H7
000021125.1	Escherichia coli O157:H7 str. EC4115
000732965.1	Escherichia coli O157:H7 str. EDL933

000730345.1	Escherichia coli O157:H7 str. SS17
000803705.1	Escherichia coli O157:H7 str. SS52
000008865.1	Escherichia coli O157:H7 str. Sakai
000022225.1	Escherichia coli O157:H7 str. TW14359
000285655.3	Escherichia coli O25b:H4-ST131
000091005.1	Escherichia coli O26:H11 str. 11368
000025165.1	Escherichia coli O55:H7 str. CB9615
000245515.1	Escherichia coli O55:H7 str. RM12579
000227625.1	Escherichia coli O7:K1 str. CE10
000183345.1	Escherichia coli O83:H1 str. NRG 857C
000257275.1	Escherichia coli P12b
000219515.3	Escherichia coli PCN033
001029125.1	Escherichia coli PCN061
000493595.1	Escherichia coli PMV-1
000801165.1	Escherichia coli RM9387
000800845.2	Escherichia coli RS218
000026285.1	Escherichia coli S88
000010385.1	Escherichia coli SE11
000010485.1	Escherichia coli SE15
000987875.1	Escherichia coli SEC470
001280325.1	Escherichia coli SF-088
001280385.1	Escherichia coli SF-166
001280405.1	Escherichia coli SF-173
001280345.1	Escherichia coli SF-468
000019645.1	Escherichia coli SMS-3-5
000988425.1	Escherichia coli SQ110
000988445.1	Escherichia coli SQ171
000988465.1	Escherichia coli SQ2203
000988355.1	Escherichia coli SQ37
000988385.1	Escherichia coli SQ88
000599665.1	Escherichia coli ST2747
000597845.1	Escherichia coli ST540

001485455.1	Escherichia coli ST648
000148605.1	Escherichia coli UM146
000220005.2	Escherichia coli UMNF18
000212715.2	Escherichia coli UMNK88
000013265.1	Escherichia coli UTI89
000968515.1	Escherichia coli VR50
000184185.1	Escherichia coli W
000262125.1	Escherichia coli Xuzhou21
001442495.1	Escherichia coli YD786
000019425.1	Escherichia coli str. K-12 substr. DH10B
000499485.1	Escherichia coli str. K-12 substr. MC4100
000350185.1	Escherichia coli str. K-12 substr. MDS42
000005845.2	Escherichia coli str. K-12 substr. MG1655
000010245.1	Escherichia coli str. K-12 substr. W3110
000233895.1	Escherichia coli str. 'clone D i14'
000233875.1	Escherichia coli str. 'clone D i2'
001469815.1	Escherichia coli uk_P46212
000026225.1	Escherichia fergusonii ATCC 35469
000178115.2	Ethanoligenens harbinense YUAN-3
000597865.1	Eubacterium acidaminophilum DSM 3953
000146185.1	[Eubacterium] eligens ATCC 27750
000152245.2	Eubacterium limosum KIST612
001481725.1	Eubacterium limosum SA11
000020605.1	[Eubacterium rectale] ATCC 33656
001189495.1	Eubacterium sulci ATCC 35585
000299435.1	Exiguobacterium antarcticum B7
000019905.1	Exiguobacterium sibiricum 255-15
000023045.1	Exiguobacterium sp. AT1b
000496635.1	Exiguobacterium sp. MH3
000953535.1	Fermentimonas caenicola
000148645.1	Ferrimonas balearica DSM 9799
000025505.1	Ferroglobus placidus DSM 10642

000152265.2	Ferroplasma acidarmanus fer1
000258425.1	Fervidicoccus fontis Kam940
000017545.1	Fervidobacterium nodosum Rt17-B1
000235405.3	Fervidobacterium pennivorans DSM 9078
000024665.1	Fibrobacter succinogenes subsp. succinogenes S85
000163895.2	Filifactor alocis ATCC 35896
000724625.1	Fimbriimonas ginsengisoli Gsoil 348
000010185.1	Finegoldia magna ATCC 29328
000597885.1	Flammeovirgaceae bacterium 311
000023725.1	Flavobacteriaceae bacterium 3519-10
000253275.1	Flavobacterium branchiophilum FL-15
000240075.2	Flavobacterium columnare ATCC 49512
000455605.1	Flavobacterium indicum GPTSA100-9 = DSM 17447
000016645.1	Flavobacterium johnsoniae UW101
000971785.1	Flavobacterium psychrophilum 4
000739395.1	Flavobacterium psychrophilum CSF259-93
000754365.1	Flavobacterium psychrophilum FPG101
000754405.1	Flavobacterium psychrophilum FPG3
000971575.1	Flavobacterium psychrophilum Flavobacterium psychrophilum strain 5
000064305.2	Flavobacterium psychrophilum JIP02/86
000971645.1	Flavobacterium psychrophilum PG2
001431805.1	Flavobacterium psychrophilum Z2
000831225.1	Flavobacterium psychrophilum phage resistant V3-5
000831185.1	Flavobacterium psychrophilum phage resistant V4-24
000831205.1	Flavobacterium psychrophilum phage resistant V4-33
000767095.1	Flavobacterium psychrophilum strain 950106-1/1
000265505.1	Flexibacter litoralis DSM 6794
000218625.1	Flexistipes sinusarabici DSM 4947
000194605.1	Fluviicola taffensis DSM 16823
000195535.1	Francisella cf. novicida Fx1
000195555.1	Francisella cf. tularensis subsp. novicida 3523
000815225.1	Francisella guangzhouensis

001042545.1	Francisella noatunensis subsp. orientalis
001042525.1	Francisella noatunensis subsp. orientalis FNO12
000505725.1	Francisella noatunensis subsp. orientalis LADL--07-285A
000262205.1	Francisella noatunensis subsp. orientalis str. Toba 04
000833255.1	Francisella philomiragia GA01-2794
000833315.1	Francisella philomiragia GA01-2801
000833195.1	Francisella philomiragia O#319-029
000833295.1	Francisella philomiragia O#319-036 [FSC 153]
000833215.1	Francisella philomiragia O#319-067
000833455.1	Francisella philomiragia subsp. philomiragia ATCC 25015
000019285.1	Francisella philomiragia subsp. philomiragia ATCC 25017
000764555.1	Francisella sp. FSC1006
000219045.1	Francisella sp. TX077308
000833235.1	Francisella tularensis subsp. holarctica
000313385.1	Francisella tularensis subsp. holarctica F92
000168775.2	Francisella tularensis subsp. holarctica FSC200
000017785.1	Francisella tularensis subsp. holarctica FTNF002-00
000009245.1	Francisella tularensis subsp. holarctica LVS
000014605.1	Francisella tularensis subsp. holarctica OSU18
000524575.1	Francisella tularensis subsp. holarctica PHIT-FT049
000018925.1	Francisella tularensis subsp. mediasiatica FSC147
000833355.1	Francisella tularensis subsp. novicida D9876
000833165.1	Francisella tularensis subsp. novicida F6168
000014645.1	Francisella tularensis subsp. novicida U112
000833475.1	Francisella tularensis subsp. tularensis
000009325.1	Francisella tularensis subsp. tularensis FSC198
001267475.1	Francisella tularensis subsp. tularensis MA00-2987
000023305.1	Francisella tularensis subsp. tularensis NE061598
000008985.1	Francisella tularensis subsp. tularensis SCHU S4
000248435.1	Francisella tularensis subsp. tularensis TI0902
000248415.1	Francisella tularensis subsp. tularensis TIGB03
001011135.1	Francisella tularensis subsp. tularensis WY-00W4114

000016105.1	Francisella tularensis subsp. tularensis WY96-3418
000978785.2	Francisella tularensis subsp. tularensis str. SCHU S4 substr. NR-28534
000978785.2	Francisella tularensis subsp. tularensis str. SCHU S4 substr. NR-28534
000058485.1	Frankia alni ACN14a
000013345.1	Frankia sp. CcI3
000018005.1	Frankia sp. EAN1pec
000166135.1	Frankia sp. EuI1c
000177615.2	Frankia symbiont of Datisca glomerata
000242255.3	Frateuria aurantia DSM 6220
000807275.1	Frischella perrara PEB0191
001455145.1	Fusobacterium hwasookii ChDC F174
001455085.1	Fusobacterium hwasookii ChDC F206
001455105.1	Fusobacterium hwasookii ChDC F300
001296085.1	Fusobacterium nucleatum subsp. animalis
000400875.1	Fusobacterium nucleatum subsp. animalis 4_8
000158275.2	Fusobacterium nucleatum subsp. animalis 7_1
001296185.1	Fusobacterium nucleatum subsp. nucleatum
000007325.1	Fusobacterium nucleatum subsp. nucleatum ATCC 25586
001296165.1	Fusobacterium nucleatum subsp. nucleatum ChDC F316
001433955.1	Fusobacterium nucleatum subsp. polymorphum
000163915.2	Fusobacterium nucleatum subsp. vincentii 3_1_27
000162235.2	Fusobacterium nucleatum subsp. vincentii 3_1_36A2
001296125.1	Fusobacterium nucleatum subsp. vincentii ChDC F8
000209675.1	Gallibacterium anatis UMN179
000145255.1	Gallionella capsiferriformans ES-2
000025205.1	Gardnerella vaginalis 409-05
001042655.1	Gardnerella vaginalis ATCC 14018 = JCM 11026
000159155.2	Gardnerella vaginalis ATCC 14019
000213955.1	Gardnerella vaginalis HMP9231
000317045.1	Geitlerinema sp. PCC 7407
000010305.1	Gemmatimonas aurantiaca T-27
000522985.1	Gemmatirosa kalamazoonesis KBS708

000827125.1	Geoalkalibacter subterraneus Red1
000009785.1	Geobacillus kaustophilus HTA426
001028085.1	Geobacillus sp. 12AMOR1
000092445.1	Geobacillus sp. C56-T3
000336445.1	Geobacillus sp. GHH01
000445995.2	Geobacillus sp. JF8
001191625.1	Geobacillus sp. LC300
000023385.1	Geobacillus sp. WCH70
000174795.2	Geobacillus sp. Y412MC52
000024705.1	Geobacillus sp. Y412MC61
000166075.1	Geobacillus sp. Y4.1MC1
001274575.1	Geobacillus stearothermophilus 10
000015745.1	Geobacillus thermodenitrificans NG80-2
000178395.2	Geobacillus thermoglucosidasius C56-YS93
001295365.1	Geobacillus thermoglucosidasius DSM 2542
000236605.1	Geobacillus thermoleovorans CCB_US3_UF5
000020725.1	Geobacter bemidjiensis Bem
000022265.1	Geobacter daltonii FRC-32
000020385.1	Geobacter lovleyi SZ
000012925.1	Geobacter metallireducens GS-15
000817955.1	Geobacter pickeringii G13
000175115.2	Geobacter sp. M18
000023645.1	Geobacter sp. M21
000210155.1	Geobacter sulfurreducens KN400
000007985.2	Geobacter sulfurreducens PCA
000016745.1	Geobacter uraniireducens Rf4
000025345.1	Geodermatophilus obscurus DSM 43160
000789255.1	Geoglobus acetivorans SBH6
001006045.1	Geoglobus ahangari 234
000599985.1	Gilliamella apicola wkB1
000226565.1	Glaciecola nitratireducens FR1064
000212335.1	Glaciecola sp. 4H-3-7+YE-5

000484535.1	Gloeobacter kilaueensis JS1
000011385.1	Gloeobacter violaceus PCC 7421
000317555.1	Gloeocapsa sp. PCC 7428
000021325.1	Gluconacetobacter diazotrophicus PA1 5
000011685.1	Gluconobacter oxydans 621H
000583855.1	Gluconobacter oxydans DSM 3504
000311765.1	Gluconobacter oxydans H24
001302565.1	Glutamicibacter arilaitensis KLBMP5180
000197735.1	Glutamicibacter arilaitensis Re117
000024785.1	Gordonia bronchialis DSM 43247
000247715.1	Gordonia polyisoprenivorans VH2
000143885.2	Gordonia sp. KTR9
001305675.1	Gordonia sp. QH-11
000060345.1	Gramella forsetii KT0803
000014285.1	Granulibacter bethesdensis CGDNIH1
000576185.1	Granulibacter bethesdensis CGDNIH2
000576085.1	Granulibacter bethesdensis CGDNIH3
000576145.1	Granulibacter bethesdensis CGDNIH4
000178955.2	Granulicella mallensis MP5ACTX8
000178975.2	Granulicella tundricola MP5ACTX9
000940805.1	Gynuella sunshinyii YC6258
000007945.1	Haemophilus ducreyi 35000HP
000767075.1	Haemophilus influenzae
000210875.1	Haemophilus influenzae 10810
000968335.1	Haemophilus influenzae 2019
000931575.1	Haemophilus influenzae 477
000931625.1	Haemophilus influenzae 723
000012185.1	Haemophilus influenzae 86-028NP
000931605.1	Haemophilus influenzae C486
000698365.1	Haemophilus influenzae CGSHiCZ412602
000197875.1	Haemophilus influenzae F3031
000200475.1	Haemophilus influenzae F3047

000465255.1	Haemophilus influenzae KR494
001457655.1	Haemophilus influenzae NCTC8143
000016465.1	Haemophilus influenzae PittEE
000016485.1	Haemophilus influenzae PittGG
000165575.1	Haemophilus influenzae R2846
000165525.1	Haemophilus influenzae R2866
000027305.1	Haemophilus influenzae Rd KW20
000210895.1	Haemophilus parainfluenzae T3T1
000742795.1	[Haemophilus] parasuis KL0318
000021885.1	Haemophilus parasuis SH0165
000740985.1	[Haemophilus] parasuis SH03
000439395.1	Haemophilus parasuis ZJ0906
000011785.1	Haemophilus somnus 129PT
000019405.1	Haemophilus somnus 2336
000597785.2	Hafnia alvei FB1
000012985.1	Hahella chejuensis KCTC 2396
000196895.1	Halalkalicoccus jeotgali B3
001011115.1	Halanaeroarchaeum sulfurireducens HSR2
001305655.1	Halanaeroarchaeum sulfurireducens M27-SA2
000166415.1	Halanaerobium hydrogeniformans missing
000165465.1	Halanaerobium praevalens DSM 2228
000024805.1	Haliangium ochraceum DSM 14365
000212735.1	Haliscomenobacter hydrossis DSM 1100
000223905.1	Haloarcula hispanica ATCC 33960
000504565.2	Haloarcula hispanica N601
000011085.1	Haloarcula marismortui ATCC 43049
000827835.1	Haloarcula sp. CBA1115
000284515.1	Halobacillus halophilus DSM 2266
000210915.2	Halobacteriovorax marinus SJ
001488575.1	Halobacterium hubeiense JI20-1
000006805.1	Halobacterium salinarum NRC-1
000069025.1	Halobacterium salinarum R1

000230955.3	Halobacterium sp. DL1
000328625.1	Halobacteroides halobius DSM 5150
001190965.1	Haloferax gibbonsii ARA6
000306765.2	Haloferax mediterranei ATCC 33500
000025685.1	Haloferax volcanii DS2
000172995.2	Halogeometricum borinquense DSM 11551
000023965.1	Halomicrobium mukohataei DSM 12286
000696485.1	Halomonas campaniensis LS21
000196875.1	Halomonas elongata DSM 2581
001431725.1	Halomonas huangheensis BJGMM-B45
000734975.2	Halomonas sp. KO116
000217715.1	Halopiger xanaduensis SH-6
000237865.1	Haloquadratum walsbyi C23
000009185.1	Haloquadratum walsbyi DSM 16790
000470655.1	Halorhabdus tiamatea SARL4B
000023945.1	Halorhabdus utahensis DSM 12940
000015585.1	Halorhodospira halophila SL1
000022205.1	Halorubrum lacusprofundi ATCC 49239
000517625.1	Halostagnicola larsenii XH-48
000025325.1	Haloterrigena turkmenica DSM 5511
000317635.1	Halothece sp. PCC 7418
000020485.1	Halothermothrix orenii H 168
000024765.1	Halothiobacillus neapolitanus c2
000328525.1	Halovivax ruber XH-70
000009305.1	Helicobacter acinonychis str. Sheeba
000259255.1	Helicobacter cetorum MIT 00-7128
000259275.1	Helicobacter cetorum MIT 99-5656
000349975.1	Helicobacter cinaedi CCUG 18818 = ATCC BAA-847
000284635.1	Helicobacter cinaedi PAGU611
000200595.1	Helicobacter felis ATCC 49179
000007905.1	Helicobacter hepaticus ATCC 51449
000091985.1	Helicobacter mustelae 12198

000192315.1	Helicobacter pylori 2017
000192335.1	Helicobacter pylori 2018
000008525.1	Helicobacter pylori 26695
000826985.1	Helicobacter pylori 26695-1
000829135.1	Helicobacter pylori 26695-1CH
000829115.1	Helicobacter pylori 26695-1CL
000827025.1	Helicobacter pylori 26695-1MET
001433495.1	Helicobacter pylori 29CaP
000178935.2	Helicobacter pylori 35A
000011725.1	Helicobacter pylori 51
000023805.1	Helicobacter pylori 52
001433515.1	Helicobacter pylori 7C
000213135.1	Helicobacter pylori 83
000148665.1	Helicobacter pylori 908
000315955.1	Helicobacter pylori Aklavik117
000317875.1	Helicobacter pylori Aklavik86
000091345.1	Helicobacter pylori B38
000196755.1	Helicobacter pylori B8
000498315.1	Helicobacter pylori BM012A
000685705.1	Helicobacter pylori BM012B
000498335.1	Helicobacter pylori BM012S
000685665.1	Helicobacter pylori BM013A
000685745.1	Helicobacter pylori BM013B
000148895.1	Helicobacter pylori Cuz20
000255955.1	Helicobacter pylori ELS37
000270005.1	Helicobacter pylori F16
000270025.1	Helicobacter pylori F30
000270045.1	Helicobacter pylori F32
000270065.1	Helicobacter pylori F57
000021165.1	Helicobacter pylori G27
000185205.1	Helicobacter pylori Gambia94/24
000013245.1	Helicobacter pylori HPAG1

000259235.1	Helicobacter pylori HUP-B14
000817025.1	Helicobacter pylori Hp238
000185185.1	Helicobacter pylori India7
000685625.1	Helicobacter pylori J166
000008785.1	Helicobacter pylori J99
000185225.1	Helicobacter pylori Lithuania75
000828955.1	Helicobacter pylori NY40
000348865.1	Helicobacter pylori OK113
000348885.1	Helicobacter pylori OK310
000021465.1	Helicobacter pylori P12
000277425.1	Helicobacter pylori PeCan18
000148875.1	Helicobacter pylori PeCan4
000224535.1	Helicobacter pylori Puno120
000224555.1	Helicobacter pylori Puno135
000307815.1	Helicobacter pylori Rif1
000307835.1	Helicobacter pylori Rif2
000148855.1	Helicobacter pylori SJM180
000224575.1	Helicobacter pylori SNT49
000148915.1	Helicobacter pylori Sat464
000277405.1	Helicobacter pylori Shi112
000277385.1	Helicobacter pylori Shi169
000277365.1	Helicobacter pylori Shi417
000020245.1	Helicobacter pylori Shi470
000590775.1	Helicobacter pylori SouthAfrica20
000185245.1	Helicobacter pylori SouthAfrica7
000392455.3	Helicobacter pylori UM032
000392515.3	Helicobacter pylori UM037
000392535.3	Helicobacter pylori UM066
000439295.2	Helicobacter pylori UM298
000392475.3	Helicobacter pylori UM299
000262655.1	Helicobacter pylori XZ274
000600045.1	Helicobacter pylori oki102

000600085.1	Helicobacter pylori oki112
000600125.1	Helicobacter pylori oki128
000600145.1	Helicobacter pylori oki154
000600165.1	Helicobacter pylori oki422
000600185.1	Helicobacter pylori oki673
000600205.1	Helicobacter pylori oki828
000600225.1	Helicobacter pylori oki898
000093185.1	Helicobacter pylori v225d
001460635.1	Helicobacter typhlonius
000019165.1	Heliobacterium modesticaldum Ice1
001267925.1	Herbaspirillum hiltneri N3
001483945.1	Herbaspirillum rubrisubalbicans M1
000143225.1	Herbaspirillum seropedicae SmR1
001040945.1	Herbaspirillum seropedicae Z67
001298655.2	Herbinix sp. SD1D
000026125.1	Herminiimonas arsenicoxydans
000018565.1	Herpetosiphon aurantiacus DSM 785
000194135.1	Hippea maritima DSM 10411
000023785.1	Hirschia baltica ATCC 49814
001011155.1	Hoeflea sp. IMCC20628
000010785.1	Hydrogenobacter thermophilus TK-6
000213785.1	Hydrogenobaculum sp. 3684
000341855.1	Hydrogenobaculum sp. HO
000215065.1	Hydrogenobaculum sp. SHO
000348765.2	Hydrogenobaculum sp. SN
000020785.1	Hydrogenobaculum sp. Y04AAS1
000737515.1	Hymenobacter sp. APR13
001280305.1	Hymenobacter sp. DG25A
000801315.1	Hymenobacter sp. DG25B
000576555.1	Hymenobacter swuensis DY53
000015145.1	Hyperthermus butylicus DSM 5456
000230975.3	Hyphomicrobium denitrificans 1NES1

000143145.1	Hyphomicrobium denitrificans ATCC 51888
000503895.1	Hyphomicrobium nitrativorans NL23
000253295.1	Hyphomicrobium sp. MC1
000013025.1	Hyphomonas neptunium ATCC 15444
000401175.1	Idiomarina loihiensis GSL 199
000008465.1	Idiomarina loihiensis L2TR
000258405.1	Ignavibacterium album JCM 16511
000017945.1	Ignicoccus hospitalis KIN4/I
001481685.1	Ignicoccus pacificus DSM 13166
000145985.1	Ignisphaera aggregans DSM 17230
000348785.1	Ilumatobacter coccineus YM16-304
000165505.1	Ilyobacter polytropus DSM 2926
001454945.1	Intestinimonas butyriciproducens AF211
000184685.1	Intrasporangium calvum DSM 43043
000215105.1	Isoptericola variabilis 225
000186345.1	Isosphaera pallida ATCC 43644
000013565.1	Jannaschia sp. CCS1
000723165.1	Janthinobacterium agaricidamnosum NBRC 102515 = DSM 9628
000013625.1	Janthinobacterium sp. Marseille
000818095.1	Jeotgalibacillus malaysiensis
000756715.2	Jeotgalicoccus sp. 13MG44_air
000024065.1	Jonesia denitrificans DSM 20603
000981765.1	Kangiella geojedonensis YCS-5
000024085.1	Kangiella koreensis DSM 16069
001399515.1	Ketogulonicigenium vulgare Hbe602
000223375.1	Ketogulonicigenium vulgare WSH-001
000164885.1	Ketogulonicigenium vulgare Y25
001302585.1	Kibdelosporangium phytohabitans KLBMP1111
000017305.1	Kineococcus radiotolerans SRS30216 = ATCC BAA-149
001458475.1	Kingella kingae KWG1
000269985.1	Kitasatospora setae KM-6054
000963575.1	Klebsiella michiganensis RC10

001022295.1	Klebsiella oxytoca CAV1099
001022115.1	Klebsiella oxytoca CAV1335
001022195.1	Klebsiella oxytoca CAV1374
000276705.2	Klebsiella oxytoca E718
000632415.1	Klebsiella oxytoca HKOPL1
000240325.1	Klebsiella oxytoca KCTC 1686
000714655.1	Klebsiella oxytoca KONIH1
000724525.1	Klebsiella oxytoca M1
000598005.1	Klebsiella pneumoniae 30660/NJST258_1
000597905.1	Klebsiella pneumoniae 30684/NJST258_2
000807395.3	Klebsiella pneumoniae 32192
000019565.1	Klebsiella pneumoniae 342
000814305.1	Klebsiella pneumoniae 34618
000406765.2	Klebsiella pneumoniae 500_1420
000364385.2	Klebsiella pneumoniae ATCC BAA-2146
001456135.1	Klebsiella pneumoniae CAV1193
001022175.1	Klebsiella pneumoniae CAV1344
001022035.1	Klebsiella pneumoniae CAV1392
001022235.1	Klebsiella pneumoniae CAV1596
000474015.1	Klebsiella pneumoniae CG43
000417225.2	Klebsiella pneumoniae DMC1097
000813205.1	Klebsiella pneumoniae HK787
001482345.1	Klebsiella pneumoniae J1
000445405.1	Klebsiella pneumoniae JM45
000220485.1	Klebsiella pneumoniae KCTC 2242
001307175.1	Klebsiella pneumoniae KP617
000968155.1	Klebsiella pneumoniae Kp52.145
001456055.1	Klebsiella pneumoniae KpN01
001456095.1	Klebsiella pneumoniae KpN06
001455995.1	Klebsiella pneumoniae MS6671
000764615.1	Klebsiella pneumoniae PMK1
000417265.2	Klebsiella pneumoniae UHKPC07

000417085.2	Klebsiella pneumoniae UHKPC33
000775955.1	Klebsiella pneumoniae XH209
000739495.1	Klebsiella pneumoniae carbapenem-resistant blaNDM-1
000742755.1	Klebsiella pneumoniae subsp. pneumoniae
000294365.1	Klebsiella pneumoniae subsp. pneumoniae 1084
000814805.1	Klebsiella pneumoniae subsp. pneumoniae 1158
000240185.2	Klebsiella pneumoniae subsp. pneumoniae HS11286
000714635.1	Klebsiella pneumoniae subsp. pneumoniae KP5-1
000281535.2	Klebsiella pneumoniae subsp. pneumoniae KPNIH1
000281435.2	Klebsiella pneumoniae subsp. pneumoniae KPNIH10
000714675.1	Klebsiella pneumoniae subsp. pneumoniae KPNIH24
000695935.1	Klebsiella pneumoniae subsp. pneumoniae KPNIH27
000717515.1	Klebsiella pneumoniae subsp. pneumoniae KPR0928
000512165.1	Klebsiella pneumoniae subsp. pneumoniae Kp13
000016305.1	Klebsiella pneumoniae subsp. pneumoniae MGH 78578
000009885.1	Klebsiella pneumoniae subsp. pneumoniae NTUH-K2044
000733255.1	Klebsiella pneumoniae subsp. pneumoniae PittNDM01
001461805.1	Klebsiella sp. G5
000025465.1	Klebsiella variicola At-22
000828055.1	Klebsiella variicola DSM 15968
000812205.1	Klebsiella variicola DX120E
001278905.1	Klebsiella variicola HKUOPLA
001022135.1	Kluyvera intermedia CAV1151
001482365.1	Kocuria flava HO-9041
001275345.1	Kocuria palustris MU14/1
000010285.1	Kocuria rhizophila DC2201
000182745.1	Komagataeibacter medellinensis NBRC 3288
000550765.1	Komagataeibacter xylinus E25
000300455.3	Kosakonia sacchari SP1
000023325.1	Kosmotoga olearia TBF 19.5.1
001027025.1	Kosmotoga pacifica SLHLJ1
000024345.1	Kribbella flavida DSM 17836

000525635.1	Kutzneria albida DSM 43870
000092905.1	Kyrpidia tusciae DSM 2912
000023925.1	Kytococcus sedentarius DSM 20547
001263205.1	Labilithrix luteola DSM 27648
000018685.1	Lachnoclostridium phytofermentans ISDg
001466725.1	Lacimicrobium alkaliphilum YelD216
000211855.3	Lacinutrix sp. 5H-3-7-4
001042405.1	Lactobacillus acetotolerans NBRC 13120
000191545.1	Lactobacillus acidophilus 30SC
000934625.1	Lactobacillus acidophilus FSI4
000389675.2	Lactobacillus acidophilus La-14
000011985.1	Lactobacillus acidophilus NCFM
000194115.1	Lactobacillus amylovorus GRL1118
000014465.1	Lactobacillus brevis ATCC 367
000359625.1	Lactobacillus brevis KB290
000298115.2	Lactobacillus buchneri CD034
000211375.1	Lactobacillus buchneri NRRL B-30929
000309565.2	Lactobacillus casei 12A
000194765.1	Lactobacillus casei BD-II
000026485.1	Lactobacillus casei BL23
000194785.1	Lactobacillus casei LC2W
000418515.1	Lactobacillus casei LOCK919
000318035.1	Lactobacillus casei W56
000019245.3	Lactobacillus casei str. Zhang
000829055.1	Lactobacillus casei subsp. casei ATCC 393
001469775.1	Lactobacillus delbrueckii subsp. bulgaricus
000191165.1	Lactobacillus delbrueckii subsp. bulgaricus 2038
000056065.1	Lactobacillus delbrueckii subsp. bulgaricus ATCC 11842 = JCM 1002
000014405.1	Lactobacillus delbrueckii subsp. bulgaricus ATCC BAA-365
000182835.1	Lactobacillus delbrueckii subsp. bulgaricus ND02
000466785.3	Lactobacillus fermentum 3872
000210515.1	Lactobacillus fermentum CECT 5716

000397165.1	Lactobacillus fermentum F-6
000010145.1	Lactobacillus fermentum IFO 3956
001314245.1	Lactobacillus gallinarum HFD4
000814885.1	Lactobacillus gasseri 130918
000014425.1	Lactobacillus gasseri ATCC 33323 = JCM 1131
001050475.1	Lactobacillus ginsenosidimutans EMML 3041
000831645.3	Lactobacillus heilongjiangensis DSM 28069
001308285.1	Lactobacillus helveticus CAUH18
000422165.1	Lactobacillus helveticus CNRZ32
000015385.1	Lactobacillus helveticus DPC 4571
000189515.1	Lactobacillus helveticus H10
000525715.1	Lactobacillus helveticus H9
000961015.1	Lactobacillus helveticus KLDS1.8701
001006025.1	Lactobacillus helveticus MB2-1
000165775.3	Lactobacillus helveticus R0052
000829395.1	Lactobacillus hokkaidonensis JCM 18461
000204985.1	Lactobacillus johnsonii DPC 6026
000091405.1	Lactobacillus johnsonii FI9785
000498675.1	Lactobacillus johnsonii N6.2
000008065.1	Lactobacillus johnsonii NCC 533
000214785.1	Lactobacillus kefiranofaciens ZW3
001050435.1	Lactobacillus koreensis 26-25
001314945.1	Lactobacillus kunkeei MP2
000248095.3	Lactobacillus mucosae LM1
000014525.1	Lactobacillus paracasei ATCC 334
001191565.1	Lactobacillus paracasei CAUH35
001244395.1	Lactobacillus paracasei L9
000582665.1	Lactobacillus paracasei N1115
000155515.2	Lactobacillus paracasei subsp. paracasei 8700:2
000829035.1	Lactobacillus paracasei subsp. paracasei JCM 8130
001443645.1	Lactobacillus paraplantarum L-ZS9
000412205.1	Lactobacillus plantarum 16

001278015.1	Lactobacillus plantarum 5-2
000931425.1	Lactobacillus plantarum B21
000604105.1	Lactobacillus plantarum DOMLa
001302645.1	Lactobacillus plantarum HFC8
000023085.1	Lactobacillus plantarum JDM1
001484005.1	Lactobacillus plantarum LZ95
000203855.3	Lactobacillus plantarum WCFS1
000338115.2	Lactobacillus plantarum ZJ316
001296095.1	Lactobacillus plantarum ZS2058
000392485.2	Lactobacillus plantarum subsp. plantarum P-8
000148815.2	Lactobacillus plantarum subsp. plantarum ST-III
000016825.1	Lactobacillus reuteri DSM 20016
000410995.1	Lactobacillus reuteri I5007
001046835.1	Lactobacillus reuteri IRT
000010005.1	Lactobacillus reuteri JCM 1112
000159455.2	Lactobacillus reuteri SD2112
000439275.1	Lactobacillus reuteri TD1
000233755.1	Lactobacillus rhamnosus ATCC 8530
000026505.1	Lactobacillus rhamnosus GG
000418475.1	Lactobacillus rhamnosus LOCK900
000418495.1	Lactobacillus rhamnosus LOCK908
000026525.1	Lactobacillus rhamnosus Lc 705
000224985.1	Lactobacillus ruminis ATCC 27782
000026065.1	Lactobacillus sakei subsp. sakei 23K
000143435.1	Lactobacillus salivarius CECT 5713
000758365.1	Lactobacillus salivarius JCM1046
000008925.1	Lactobacillus salivarius UCC118
001011095.1	Lactobacillus salivarius str. Ren
000225325.1	Lactobacillus sanfranciscensis TMW 1.1304
000761135.1	Lactobacillus sp. wkB8
000269925.1	Lactococcus garvieae ATCC 49156
000269945.1	Lactococcus garvieae Lg2

000761115.1	Lactococcus lactis AI06
000236475.1	Lactococcus lactis subsp. cremoris A76
000468955.1	Lactococcus lactis subsp. cremoris KW2
000009425.1	Lactococcus lactis subsp. cremoris MG1363
000143205.1	Lactococcus lactis subsp. cremoris NZ9000
000014545.1	Lactococcus lactis subsp. cremoris SK11
000312685.1	Lactococcus lactis subsp. cremoris UC509.9
000807375.1	Lactococcus lactis subsp. lactis
000192705.1	Lactococcus lactis subsp. lactis CV56
000344575.1	Lactococcus lactis subsp. lactis IO-1
000006865.1	Lactococcus lactis subsp. lactis Il1403
000025045.1	Lactococcus lactis subsp. lactis KF147
000479375.2	Lactococcus lactis subsp. lactis KLDS 4.0325
000478255.2	Lactococcus lactis subsp. lactis NCDO 2118
000981525.1	Lactococcus piscium MKFS47
000021025.1	Laribacter hongkongensis HLHK9
001293125.1	Lawsonella clevelandensis X1036
001281505.1	Lawsonella clevelandensis X1698
000331715.1	Lawsonia intracellularis N343
000055945.1	Lawsonia intracellularis PHE/MN1-00
000166395.1	Leadbetterella byssophila DSM 17132
000953135.1	Legionella fallonii LLAP-10
000953655.1	Legionella hackeliae ATCC35250
000091785.1	Legionella longbeachae NSW150
000512355.1	Legionella oakridgensis ATCC 33761 = DSM 21215
000092625.1	Legionella pneumophila 2300/99 Alcoy
000092545.1	Legionella pneumophila str. Corby
000048665.1	Legionella pneumophila str. Lens
000048645.1	Legionella pneumophila str. Paris
000306845.1	Legionella pneumophila subsp. pneumophila
000239175.1	Legionella pneumophila subsp. pneumophila ATCC 43290
000347615.1	Legionella pneumophila subsp. pneumophila LPE509

000277025.1	Legionella pneumophila subsp. pneumophila str. Hextuple_2q
000277065.1	Legionella pneumophila subsp. pneumophila str. Hextuple_3a
000008485.1	Legionella pneumophila subsp. pneumophila str. Philadelphia 1
000404245.1	Legionella pneumophila subsp. pneumophila str. Thunder Bay
000470775.1	Leifsonia xyli subsp. cynodontis DSM 46306
000007665.1	Leifsonia xyli subsp. xyli str. CTCB07
000511355.1	Leisingera methylohalidivorans DSM 14336
000316605.1	Leptolyngbya sp. PCC 7376
000017605.1	Leptospira biflexa serovar Patoc strain 'Patoc 1 (Ames)'
000017685.1	Leptospira biflexa serovar Patoc strain 'Patoc 1 (Paris)'
001444465.1	Leptospira borgpetersenii serovar Ballum
000013965.1	Leptospira borgpetersenii serovar Hardjo-bovis str. JB197
000013945.1	Leptospira borgpetersenii serovar Hardjo-bovis str. L550
001010765.1	Leptospira interrogans serovar Bratislava
000007685.1	Leptospira interrogans serovar Copenhageni str. Fiocruz L1-130
001293065.1	Leptospira interrogans serovar Hardjo str. Norma
000092565.1	Leptospira interrogans serovar Lai str. 56601
000231175.1	Leptospira interrogans serovar Lai str. IPAV
000941035.1	Leptospira interrogans serovar Linhai str. 56609
001047635.1	Leptospira interrogans serovar Manilae
000313175.2	Leptospira santarosai serovar Shermani str. LT 821
000299235.1	Leptospirillum ferriphilum ML-04
000695975.1	Leptospirillum ferriphilum YSK
000284315.1	Leptospirillum ferrooxidans C2-3
001186405.1	Leptospirillum sp. Group II 'CF-1'
000019785.1	Leptothrix cholodnii SP-6
000023905.1	Leptotrichia buccalis C-1013-b
001274535.1	Leptotrichia sp. oral taxon 212
000300135.1	Leuconostoc carnosum JB16
000026405.1	Leuconostoc citreum KM20
000298875.1	Leuconostoc gelidum JB7
000196855.1	Leuconostoc gelidum subsp. gasicomitatum LMG 18811

000092505.1	Leuconostoc kimchii IMSNU 11154
000512955.1	Leuconostoc mesenteroides KFRI-MG
001047695.1	Leuconostoc mesenteroides subsp. dextranicum
000014445.1	Leuconostoc mesenteroides subsp. mesenteroides ATCC 8293
000234825.3	Leuconostoc mesenteroides subsp. mesenteroides J18
000219785.1	Leuconostoc sp. C2
000325745.1	Liberibacter crescens BT-1
001412575.1	Limnohabitans sp. 103DPR2
001412535.1	Limnohabitans sp. 63ED37-2
000565155.1	Listeria ivanovii WSLC3009
000763515.1	Listeria ivanovii subsp. ivanovii
000252975.1	Listeria ivanovii subsp. ivanovii PAM 55
000763475.1	Listeria ivanovii subsp. londoniensis
000600015.1	Listeria monocytogenes
000258905.1	Listeria monocytogenes 07PF0776
000093125.2	Listeria monocytogenes 08-5578
000022925.1	Listeria monocytogenes 08-5923
000168695.2	Listeria monocytogenes 10403S
000577745.1	Listeria monocytogenes 6179
000307025.1	Listeria monocytogenes ATCC 19117
000438605.1	Listeria monocytogenes C1-387
000681515.1	Listeria monocytogenes CFSAN006122
001005985.1	Listeria monocytogenes CFSAN007956
001005925.1	Listeria monocytogenes CFSAN008100
001047715.1	Listeria monocytogenes CFSAN023463
000582845.1	Listeria monocytogenes EGD
000168575.2	Listeria monocytogenes FSL R2-561
000168595.2	Listeria monocytogenes Finland 1998
000021185.1	Listeria monocytogenes HCC23
000168635.2	Listeria monocytogenes J0161
000438665.1	Listeria monocytogenes J1-108
000195395.4	Listeria monocytogenes J1-220

000438705.2	Listeria monocytogenes J1776
000195435.4	Listeria monocytogenes J1816
000438725.2	Listeria monocytogenes J1817
000438745.2	Listeria monocytogenes J1926
000438645.1	Listeria monocytogenes J2-031
000438625.1	Listeria monocytogenes J2-064
001027085.1	Listeria monocytogenes L1846
001027065.1	Listeria monocytogenes L2074
001027165.1	Listeria monocytogenes L2624
001027125.1	Listeria monocytogenes L2625
001027245.1	Listeria monocytogenes L2626
001027205.1	Listeria monocytogenes L2676
000307085.1	Listeria monocytogenes L312
001188655.1	Listeria monocytogenes LM850658
000382925.1	Listeria monocytogenes La111
000746625.1	Listeria monocytogenes Lm60
001483425.1	Listeria monocytogenes Lm 3136
001483405.1	Listeria monocytogenes Lm 3163
001483445.1	Listeria monocytogenes Lm N1546
000218305.1	Listeria monocytogenes M7
000438685.2	Listeria monocytogenes N1-011A
000950775.1	Listeria monocytogenes N2306
000382945.1	Listeria monocytogenes N53-1
000800335.1	Listeria monocytogenes NTSN
000438585.1	Listeria monocytogenes R2-502
000613085.1	Listeria monocytogenes R479a
000307065.1	Listeria monocytogenes SLCC2376
000307615.1	Listeria monocytogenes SLCC2378
000307005.1	Listeria monocytogenes SLCC2479
000306905.1	Listeria monocytogenes SLCC2540
000307045.1	Listeria monocytogenes SLCC5850
000306985.1	Listeria monocytogenes SLCC7179

000568475.1	Listeria monocytogenes WSLC1001
000568935.1	Listeria monocytogenes WSLC1042
001454845.1	Listeria monocytogenes WSLC 1018
001454865.1	Listeria monocytogenes WSLC 1019
001454885.1	Listeria monocytogenes WSLC 1020
001454925.1	Listeria monocytogenes WSLC 1047
000197755.2	Listeria monocytogenes serotype 1/2b str. SLCC2755
000210815.2	Listeria monocytogenes serotype 1/2c str. SLCC2372
000209755.1	Listeria monocytogenes serotype 4a str. L99
000026705.1	Listeria monocytogenes serotype 4b str. CLIP 80459
000008285.1	Listeria monocytogenes serotype 4b str. F2365
000318055.1	Listeria monocytogenes serotype 4b str. LL195
000027145.1	Listeria seeligeri serovar 1/2b str. SLCC3954
000060285.1	Listeria welshimeri serovar 6b str. SLCC5334
001190945.1	Luteipulveratus mongoliensis MN07-A0370
000724775.3	Lysinibacillus fusiformis RB-21
000017965.1	Lysinibacillus sphaericus C3-41
000600105.1	Lysinibacillus varians GY32
001442745.1	Lysobacter antibioticus 76
001442535.1	Lysobacter antibioticus ATCC 29479
001442785.1	Lysobacter capsici 55
001442805.1	Lysobacter gummosus 3.2.11
000010585.1	Macrococcus caseolyticus JCSC5402
000025225.2	Mageeibacillus indolicus UPII9-5
000014865.1	Magnetococcus marinus MC-1
000968135.1	Magnetospira sp. QH-2
000513295.1	Magnetospirillum gryphiswaldense MSR-1 v2
000009985.1	Magnetospirillum magneticum AMB-1
000213255.1	Mahella australiensis 50-1 BON
000963635.1	Mannheimia haemolytica 89010807N
000422145.1	Mannheimia haemolytica D153
000427275.1	Mannheimia haemolytica D171

000422095.1	Mannheimia haemolytica D174
000376645.1	Mannheimia haemolytica M42548
000349765.1	Mannheimia haemolytica USDA-ARS-USMARC-183
000819525.1	Mannheimia haemolytica USDA-ARS-USMARC-184
000349785.1	Mannheimia haemolytica USDA-ARS-USMARC-185
000439735.1	Mannheimia haemolytica USMARC_2286
000007745.1	Mannheimia succiniciproducens MBEL55E
000521605.1	Mannheimia varigena USDA-ARS-USMARC-1261
000521655.1	Mannheimia varigena USDA-ARS-USMARC-1296
000521695.1	Mannheimia varigena USDA-ARS-USMARC-1312
000521685.1	Mannheimia varigena USDA-ARS-USMARC-1388
000153165.2	Maribacter sp. HTCC2170
000014745.1	Maricaulis maris MCS10
000224005.3	Marichromatium purpuratum 984
000195335.1	Marinithermus hydrothermalis DSM 14884
000255135.1	Marinitoga piezophila KA3
000166295.1	Marinobacter adhaerens HP15
000284615.1	Marinobacter hydrocarbonoclasticus ATCC 49840
000015365.1	Marinobacter hydrocarbonoclasticus VT8
001043175.1	Marinobacter psychrophilus 20041
000831005.1	Marinobacter salarius R9SW1
000830985.1	Marinobacter similis A3d10
000283275.1	Marinobacter sp. BSs20148
001266795.1	Marinobacter sp. CP1
000192865.1	Marinomonas mediterranea MMB-1
000214215.1	Marinomonas posidonica IVIA-Po-181
000017285.1	Marinomonas sp. MWYL1
001046955.1	Marinovum algicola DG 898
000183425.1	Marivirga tractuosa DSM 4126
000960975.1	Martelella endophytica YC6887
001191005.1	Massilia sp. NR 4-1
001412595.1	Massilia sp. WG5

001304715.1	Megasphaera elsdenii 14-14
000024425.1	Meiothermus ruber DSM 1279
000092125.1	Meiothermus silvanus DSM 9946
000279145.1	Melioribacter roseus P3M-2
000283975.1	Melissococcus plutonius DAT561
000008305.1	Mesoplasma florum L1
000479355.1	Mesoplasma florum W37
000230995.3	Mesorhizobium australicum WSM2073
000185905.1	Mesorhizobium ciceri biovar biserrulae WSM1271
000009625.1	Mesorhizobium loti MAFF303099
000176035.2	Mesorhizobium opportunistum WSM2075
000147715.3	Mesotoga prima MesG1.Ag.4.2
000204925.1	Metallosphaera cuprina Ar-4
001266695.1	Metallosphaera sedula ARS120-1
001266715.1	Metallosphaera sedula ARS120-2
001266655.1	Metallosphaera sedula ARS50-1
001266675.1	Metallosphaera sedula ARS50-2
000016605.1	Metallosphaera sedula DSM 5348
001266735.1	Metallosphaera sedula SARC-M1
000953115.1	Methanobacterium formicicum
000762265.1	Methanobacterium formicicum BRM9
000191585.1	Methanobacterium lacus AL-21
000214725.1	Methanobacterium paludis SWAN-1
000499765.1	Methanobacterium sp. MB1
001477655.1	Methanobrevibacter millerae SM9
000024185.1	Methanobrevibacter ruminantium M1
000016525.1	Methanobrevibacter smithii ATCC 35061
000404165.1	Methanobrevibacter sp. AbM4
000739065.1	Methanocaldococcus bathoardescens JH146
000023985.1	Methanocaldococcus fervens AG86
000092305.1	Methanocaldococcus infernus ME
000091665.1	Methanocaldococcus jannaschii DSM 2661

000025525.1	Methanocaldococcus sp. FS406-22
000024625.1	Methanocaldococcus vulcanius M7
000063445.1	Methanocella arvoryzae MRE50
000251105.1	Methanocella conradii HZ254
000011005.1	Methanocella paludicola SANAE
000013725.1	Methanococcoides burtonii DSM 6242
000970325.1	Methanococcoides methylutens MM1
000017185.1	Methanococcus aeolicus Nankai-3
000016125.1	Methanococcus maripaludis C5
000018485.1	Methanococcus maripaludis C6
000017225.1	Methanococcus maripaludis C7
000011585.1	Methanococcus maripaludis S2
000220645.1	Methanococcus maripaludis X1
000017165.1	Methanococcus vannielii SB
000006175.2	Methanococcus voltae A3
000015765.1	Methanocorpusculum labreanum Z
000304355.2	Methanoculleus bourgensis MS2
000015825.1	Methanoculleus marisnigri JR1
000196655.1	Methanohalobium evestigatum Z-7303
000025865.1	Methanohalophilus mahii DSM 5219
000147875.1	Methanolacinia petrolearia DSM 11571
000306725.1	Methanolobus psychrophilus R15
000328665.1	Methanomethylovorans hollandica DSM 15978
000007185.1	Methanopyrus kandleri AV19
000017625.1	Methanoregula boonei 6A8
000327485.1	Methanoregula formicica SMSP
000204415.1	Methanosaeta concilii GP6
000235565.1	Methanosaeta harundinacea 6Ac
000014945.1	Methanosaeta thermophila PT
000217995.1	Methanosalsum zhilinae DSM 4017
000007345.1	Methanosarcina acetivorans C2A
000970065.1	Methanosarcina barkeri 227

000970305.1	Methanosarcina barkeri 3
001027005.1	Methanosarcina barkeri CM1
000970025.1	Methanosarcina barkeri MS
000195895.1	Methanosarcina barkeri str. Fusaro
000969985.1	Methanosarcina barkeri str. Wiesmoor
000970285.1	Methanosarcina horonobensis HB-1 = JCM 15518
000970265.1	Methanosarcina lacustris Z-7289
000970245.1	Methanosarcina mazei C16
000007065.1	Methanosarcina mazei Go1
000970225.1	Methanosarcina mazei LYC
000970205.1	Methanosarcina mazei S-6
000970185.1	Methanosarcina mazei SarPi
000341715.1	Methanosarcina mazei Tuc01
000970165.1	Methanosarcina mazei WWM610
000970145.1	Methanosarcina siciliae C2J
000970125.1	Methanosarcina siciliae HI350
000970085.1	Methanosarcina siciliae T4/M
000969945.1	Methanosarcina sp. Kolksee
000970045.1	Methanosarcina sp. MTP4
000970005.1	Methanosarcina sp. WH1
000969965.1	Methanosarcina sp. WWM596
000969925.1	Methanosarcina thermophila CHTI-55
000969885.1	Methanosarcina thermophila TM-1
000969905.1	Methanosarcina vacuolata Z-761
000012545.1	Methanosphaera stadtmanae DSM 3091
000021965.1	Methanosphaerula palustris E1-9c
000013445.1	Methanospirillum hungatei JF-1
000145295.1	Methanothermobacter marburgensis str. Marburg
000828575.1	Methanothermobacter sp. CaT2
000008645.1	Methanothermobacter thermautotrophicus str. Delta H
000179575.2	Methanothermococcus okinawensis IH1
000166095.1	Methanothermus fervidus DSM 2088

000214415.1	Methanotorris igneus Kol 5
000953475.1	Methylacidiphilum fumariolicum SolV
000019665.1	Methylacidiphilum infernorum V4
000015725.1	Methylibium petroleiphilum PM1
000013705.1	Methylobacillus flagellatus KT
000022685.1	Methylobacterium extorquens AM1
000021845.1	Methylobacterium extorquens CM4
000083545.1	Methylobacterium extorquens DM4
000018845.1	Methylobacterium extorquens PA1
000022085.1	Methylobacterium nodulans ORS 2060
000757795.1	Methylobacterium oryzae CBMB20
000019945.1	Methylobacterium populi BJ001
000019725.1	Methylobacterium radiotolerans JCM 2831
000019365.1	Methylobacterium sp. 4-46
000828475.1	Methyloceanibacter caenitepidi Gela4
000021745.1	Methylocella silvestris BL2
000008325.1	Methylococcus capsulatus str. Bath
000304315.1	Methylocystis sp. SC2
000968535.1	Methylomicrobium alcaliphilum 20Z
000214665.1	Methylomonas methanica MC09
000260965.1	Methylophaga frappieri JAM7
000260985.2	Methylophaga nitratireducenticrescens JAM1
001183865.1	Methylophilus sp. TWE2
000023705.1	Methylotenera mobilis JLW8
000093025.1	Methylotenera versatilis 301
000023745.1	Methylovorus glucosetrophus SIP3-4
000183115.1	Methylovorus sp. MP688
000226315.1	Micavibrio aeruginosavorus ARL-13
000348745.1	Micavibrio aeruginosavorus EPB
001266755.1	Microbacterium sp. CGR1
001314225.1	Microbacterium sp. No. 7
000202635.1	Microbacterium testaceum StLB037

000023205.1	Micrococcus luteus NCTC 2665
000317515.1	Microcoleus sp. PCC 7113
000981785.1	Microcystis aeruginosa NIES-2549
000010625.1	Microcystis aeruginosa NIES-843
001264245.1	Microcystis panniformis FACHB-1757
000270245.1	Microlunatus phosphovorus NM-1
000145235.1	Micromonospora aurantiaca ATCC 27029
000177655.2	Micromonospora sp. L5
000196535.1	Mobiluncus curtisii ATCC 43063
000306785.1	Modestobacter marinus BC501
000755705.1	Mollicutes bacterium HR1
000013105.1	Moorella thermoacetica ATCC 39073
001267435.1	Moorella thermoacetica DSM 2955
001267405.1	Moorella thermoacetica DSM 521
000740455.1	Moraxella catarrhalis 25240
000766665.1	Moraxella catarrhalis ATCC 25239
000092265.1	Moraxella catarrhalis BBH18
000286435.2	Morganella morganii subsp. morganii KT
000953735.1	Moritella viscosa
000723505.1	Mucinivorans hirudinis
000963865.1	Muricauda lutaonensis CC-HSB-11
000224085.1	Muricauda ruestringensis DSM 13258
000770215.1	Mycobacterium abscessus 4529
000069185.1	Mycobacterium abscessus ATCC 19977
000770175.1	Mycobacterium abscessus DJO-44274
001430775.1	Mycobacterium abscessus NOV0213
001050395.1	Mycobacterium abscessus UC22
000758405.1	Mycobacterium abscessus subsp. bolletii
000758385.1	Mycobacterium abscessus subsp. bolletii 103
000445035.1	Mycobacterium abscessus subsp. bolletii 50594
000497265.2	Mycobacterium abscessus subsp. bolletii CCUG 48898 = JCM 15300
000277775.2	Mycobacterium abscessus subsp. bolletii str. GO 06

000253355.1	Mycobacterium africanum GM041182
000014985.1	Mycobacterium avium 104
000770235.1	Mycobacterium avium subsp. avium
000758285.1	Mycobacterium avium subsp. avium 2285 (R)
000831285.1	Mycobacterium avium subsp. avium 2285 (S)
000829075.1	Mycobacterium avium subsp. hominissuis TH135
000835225.1	Mycobacterium avium subsp. paratuberculosis
000007865.1	Mycobacterium avium subsp. paratuberculosis K-10
000390085.1	Mycobacterium avium subsp. paratuberculosis MAP4
001078615.1	Mycobacterium bovis 1595
000758245.1	Mycobacterium bovis BAA-935
001043255.1	Mycobacterium bovis BCG
001483905.1	Mycobacterium bovis BCG-1 (Russia)
000194075.3	Mycobacterium bovis BCG str. ATCC 35743
000338715.2	Mycobacterium bovis BCG str. Korea 1168P
000234725.1	Mycobacterium bovis BCG str. Mexico
000967285.1	Mycobacterium bovis BCG str. Moreau RDJ
000009445.1	Mycobacterium bovis BCG str. Pasteur 1173P2
000010685.1	Mycobacterium bovis BCG str. Tokyo 172
000253375.1	Mycobacterium canettii CIPT 140010059
000266905.1	Mycobacterium chubuense NBB4
001307545.1	Mycobacterium fortuitum CT6
000016365.1	Mycobacterium gilvum PYR-GCK
000184435.1	Mycobacterium gilvum Spyr1
001187505.1	Mycobacterium goodii X7B
000340435.3	Mycobacterium haemophilum ATCC 29548
000298095.1	Mycobacterium indicus pranii MTCC 9506
000767485.1	Mycobacterium intracellulare 1956
000277125.1	Mycobacterium intracellulare ATCC 13950
000277145.1	Mycobacterium intracellulare MOTT-02
000276825.1	Mycobacterium intracellulare MOTT-64
000831265.1	Mycobacterium kansasii 662

000831305.1	Mycobacterium kansasii 824
000157895.2	Mycobacterium kansasii ATCC 12478
000026685.1	Mycobacterium leprae Br4923
000026445.2	Mycobacterium liflandii 128FXT
000723425.2	Mycobacterium marinum E11
000018345.1	Mycobacterium marinum M
000317305.3	Mycobacterium neoaurum VKM Ac-1815D
000230895.3	Mycobacterium rhodesiae NBB3
000214155.1	Mycobacterium sinense JDM601
000767665.1	Mycobacterium smegmatis INHR1
000767705.1	Mycobacterium smegmatis INHR2
001457595.1	Mycobacterium smegmatis NCTC8159
000015005.1	Mycobacterium smegmatis str. MC2 155
001021385.1	Mycobacterium sp. EPa45
000016005.1	Mycobacterium sp. JLS
000328565.1	Mycobacterium sp. JS623
000015405.1	Mycobacterium sp. KMS
000014165.1	Mycobacterium sp. MCS
000262165.1	Mycobacterium sp. MOTT36Y
000416365.2	Mycobacterium sp. VKM Ac-1817D
000954155.1	Mycobacterium tuberculosis
000786505.1	Mycobacterium tuberculosis 49-02
000331445.1	Mycobacterium tuberculosis 7199-99
000756525.1	Mycobacterium tuberculosis 96075
000756545.1	Mycobacterium tuberculosis 96121
000572175.1	Mycobacterium tuberculosis BT1
000572155.1	Mycobacterium tuberculosis BT2
000389925.1	Mycobacterium tuberculosis CAS/NITR204
000270345.1	Mycobacterium tuberculosis CCDC5079
000270365.1	Mycobacterium tuberculosis CCDC5180
000008585.1	Mycobacterium tuberculosis CDC1551
000224435.1	Mycobacterium tuberculosis CTRI-2

000422125.1	Mycobacterium tuberculosis EAI5
000389945.1	Mycobacterium tuberculosis EAI5/NITR206
000016925.1	Mycobacterium tuberculosis F11
000016145.1	Mycobacterium tuberculosis H37Ra
000195955.2	Mycobacterium tuberculosis H37Rv
000827085.1	Mycobacterium tuberculosis H37RvSiena
000572125.1	Mycobacterium tuberculosis HKBS1
000698475.1	Mycobacterium tuberculosis K
000706665.1	Mycobacterium tuberculosis KIT87190
000023625.1	Mycobacterium tuberculosis KZN 1435
000154585.2	Mycobacterium tuberculosis KZN 4207
000154605.2	Mycobacterium tuberculosis KZN 605
000277085.1	Mycobacterium tuberculosis RGTB327
000277105.1	Mycobacterium tuberculosis RGTB423
001275565.1	Mycobacterium tuberculosis SCAID 187.0
000193185.2	Mycobacterium tuberculosis W-148
000738445.1	Mycobacterium tuberculosis ZMC13-264
000738475.1	Mycobacterium tuberculosis ZMC13-88
000364825.1	Mycobacterium tuberculosis str. Beijing/NITR203
000350205.1	Mycobacterium tuberculosis str. Erdman = ATCC 35801
000153685.2	Mycobacterium tuberculosis str. Haarlem
000389905.1	Mycobacterium tuberculosis str. Haarlem/NITR202
000828995.1	Mycobacterium tuberculosis str. Kurono
000013925.1	Mycobacterium ulcerans Agy99
000015305.1	Mycobacterium vanbaalenii PYR-1
000418535.2	Mycobacterium yongonense 05-1390
000089865.1	Mycoplasma agalactiae 5632
000063605.1	Mycoplasma agalactiae PG2
000020065.1	Mycoplasma arthritidis 158L3-1
000696015.1	Mycoplasma bovis CQ-W70
000270525.1	Mycoplasma bovis HB0801
000219375.1	Mycoplasma bovis Hubei-1

001043135.1	Mycoplasma bovis NM 2012
000183385.1	Mycoplasma bovis PG45
000524555.1	Mycoplasma bovoculi M165/69
000829335.1	Mycoplasma californicum HAZ160_1
000695835.1	Mycoplasma californicum ST-6
000828855.1	Mycoplasma canadense HAZ360_1
000988065.1	Mycoplasma canis LV
000012765.1	Mycoplasma capricolum subsp. capricolum ATCC 27343
000952915.1	Mycoplasma capricolum subsp. capripneumoniae
000835085.1	Mycoplasma capricolum subsp. capripneumoniae 87001
000025845.1	Mycoplasma crocodyli MP145
000328725.1	Mycoplasma cynos C142
000941075.1	Mycoplasma dispar ATCC 27140
000148625.1	Mycoplasma fermentans JER
000186005.1	Mycoplasma fermentans M64
000815065.1	Mycoplasma flocculare ATCC 27399
000965765.1	Mycoplasma gallinaceum B2096 8B
000286795.1	Mycoplasma gallisepticum CA06_2006.052-5-2P
000286775.1	Mycoplasma gallisepticum NC06_2006.080-5-2P
000286815.1	Mycoplasma gallisepticum NC08_2008.031-4-3P
000286695.1	Mycoplasma gallisepticum NC95_13295-2-2P
000286715.1	Mycoplasma gallisepticum NC96_1596-4-2P
000286735.1	Mycoplasma gallisepticum NY01_2001.047-5-1P
000211545.5	Mycoplasma gallisepticum S6
000286675.1	Mycoplasma gallisepticum VA94_7994-1-7P
000286755.1	Mycoplasma gallisepticum WI01_2001.043-13-2P
000025385.1	Mycoplasma gallisepticum str. F
000025365.1	Mycoplasma gallisepticum str. R(high)
000092585.1	Mycoplasma gallisepticum str. R(low)
000027325.1	Mycoplasma genitalium G37
000292505.1	Mycoplasma genitalium M2288
000292405.1	Mycoplasma genitalium M2321

000292445.1	Mycoplasma genitalium M6282
000292485.1	Mycoplasma genitalium M6320
000238995.1	Mycoplasma haemocanis str. Illinois
000186985.3	Mycoplasma haemofelis Ohio2
000200735.1	Mycoplasma haemofelis str. Langford 1
000947915.1	Mycoplasma hominis AF1
000085865.1	Mycoplasma hominis ATCC 23114
000767725.1	Mycoplasma hominis ATCC 27545
001017595.1	Mycoplasma hominis Sprott
000183185.1	Mycoplasma hyopneumoniae 168
000400855.1	Mycoplasma hyopneumoniae 168-L
000008405.1	Mycoplasma hyopneumoniae 232
000427215.1	Mycoplasma hyopneumoniae 7422
000008225.1	Mycoplasma hyopneumoniae 7448
000008205.1	Mycoplasma hyopneumoniae J
000496815.1	Mycoplasma hyorhinis DBS 1050
000241125.1	Mycoplasma hyorhinis GDL-1
000145705.1	Mycoplasma hyorhinis HUB-1
000211295.1	Mycoplasma hyorhinis MCLD
000313635.1	Mycoplasma hyorhinis SK76
000183365.1	Mycoplasma leachii PG50
000008365.1	Mycoplasma mobile 163K
000253075.1	Mycoplasma mycoides subsp. capri LC str. 95010
000023685.1	Mycoplasma mycoides subsp. capri str. GM12
000800785.1	Mycoplasma mycoides subsp. mycoides
000143865.1	Mycoplasma mycoides subsp. mycoides SC str. Gladysdale
000011445.1	Mycoplasma mycoides subsp. mycoides SC str. PG1
000508245.1	Mycoplasma ovis str. Michigan
000477415.1	Mycoplasma parvum str. Indiana
000011225.1	Mycoplasma penetrans HF-2
000387745.2	Mycoplasma pneumoniae 19294
000283755.1	Mycoplasma pneumoniae 309

001272715.1	Mycoplasma pneumoniae 39443
001272735.1	Mycoplasma pneumoniae 51494
001272755.1	Mycoplasma pneumoniae 54089
001272775.1	Mycoplasma pneumoniae 54524
001272795.1	Mycoplasma pneumoniae 85084
001272815.1	Mycoplasma pneumoniae 85138
000143945.1	Mycoplasma pneumoniae FH
001272855.1	Mycoplasma pneumoniae M1139
000027345.1	Mycoplasma pneumoniae M129
000331085.2	Mycoplasma pneumoniae M129-B7
001272875.1	Mycoplasma pneumoniae M2192
001272895.1	Mycoplasma pneumoniae M2592
000733995.1	Mycoplasma pneumoniae M29
001272915.1	Mycoplasma pneumoniae MAC
000319675.2	Mycoplasma pneumoniae PI 1428
000319655.2	Mycoplasma pneumoniae PO1
000224105.1	Mycoplasma putrefaciens KS1
000376625.1	Mycoplasma putrefaciens Mput9231
001484045.1	Mycoplasma sp. (ex Biomphalaria glabrata)
000203215.1	Mycoplasma suis KI3806
000179035.2	Mycoplasma suis str. Illinois
000008245.1	Mycoplasma synoviae 53
000969765.1	Mycoplasma synoviae ATCC 25204
000277795.1	Mycoplasma wenyonii str. Massachusetts
000875755.1	Mycoplasma yeatsii GM274B
001481655.1	Myroides odoratimimus PR63039
000833025.1	Myroides profundi D25
000807225.1	Myroides sp. A21
000988565.1	Myxococcus fulvus 124B02
000219105.1	Myxococcus fulvus HW-1
000280925.3	Myxococcus hansupus contaminant ex DSM 436
000331735.1	Myxococcus stipitatus DSM 14675

000012685.1	Myxococcus xanthus DK 1622
000024365.1	Nakamurella multipartita DSM 44233
000008085.1	Nanoarchaeum equitans Kin4-M
000020005.1	Natranaerobius thermophilus JW/NM-WN-LF
000025625.1	Natrialba magadii ATCC 43099
000230735.3	Natrinema pellirubrum DSM 15624
000281695.1	Natrinema sp. J7-2
000230715.3	Natronobacterium gregoryi SP2
000328685.1	Natronococcus occultus SP4
000591055.1	Natronomonas moolapensis 8.8.11
000026045.1	Natronomonas pharaonis DSM 2160
000021725.1	Nautilia profundicola AmH
000818035.1	Neisseria elongata subsp. glycolytica ATCC 29315
001047275.1	Neisseria gonorrhoeae 35/02
001047225.1	Neisseria gonorrhoeae FA19
001047255.1	Neisseria gonorrhoeae FA6140
000006845.1	Neisseria gonorrhoeae FA 1090
000156855.2	Neisseria gonorrhoeae MS11
000020105.1	Neisseria gonorrhoeae NCCP11945
000196295.1	Neisseria lactamica 020-06
000626595.1	Neisseria meningitidis
000014105.1	Neisseria meningitidis 053442
000026965.1	Neisseria meningitidis 8013
001029815.1	Neisseria meningitidis B6116/77
000009465.1	Neisseria meningitidis FAM18
000191425.1	Neisseria meningitidis G2136
000191445.1	Neisseria meningitidis H44/76
000816165.1	Neisseria meningitidis LNP21362
000191465.1	Neisseria meningitidis M01-240149
000191485.1	Neisseria meningitidis M01-240355
000191505.1	Neisseria meningitidis M04-240196
001029835.1	Neisseria meningitidis M0579

000800415.1	Neisseria meningitidis M10208
000800275.1	Neisseria meningitidis M7124
000008805.1	Neisseria meningitidis MC58
000800315.1	Neisseria meningitidis NM3682
000800355.1	Neisseria meningitidis NM3683
000800235.1	Neisseria meningitidis NM3686
000191525.1	Neisseria meningitidis NZ-05/33
000253215.1	Neisseria meningitidis WUE 2594
000009105.1	Neisseria meningitidis Z2491
000083565.1	Neisseria meningitidis alpha14
000152165.1	Neisseria meningitidis alpha710
000731295.1	Neorhizobium galegae HAMBI 1141
000731315.1	Neorhizobium galegae HAMBI 540
000632985.1	Neorickettsia helminthoeca str. Oregon
000022525.1	Neorickettsia risticii str. Illinois
000013165.1	Neorickettsia sennetsu str. Miyayama
000243115.3	Niabella soli DSM 19437
000246855.1	Niastella koreensis GR20-10
000186245.1	Nitratifractor salsuginis DSM 16511
000010325.1	Nitratiruptor sp. SB155-2
000013885.1	Nitrobacter hamburgensis X14
000012725.1	Nitrobacter winogradskyi Nb-255
000024725.1	Nitrosococcus halophilus Nc 4
000012805.1	Nitrosococcus oceani ATCC 19707
000143085.1	Nitrosococcus watsonii C-113
001007935.1	Nitrosomonas communis Nm2
000009145.1	Nitrosomonas europaea ATCC 19718
000014765.1	Nitrosomonas eutropha C91
000175095.2	Nitrosomonas sp. AL212
000219585.1	Nitrosomonas sp. Is79A3
001455205.1	Nitrosomonas ureae Nm10
000018465.1	Nitrosopumilus maritimus SCM1

000698785.1	Nitrososphaera viennensis EN76
000619905.2	Nitrosospira briensis C-128
000196355.1	Nitrosospira multiformis ATCC 25196
000196815.1	Nitrospira defluvii
001273775.1	Nitrospira moscoviensis NSP M-1
000250675.3	Nocardia brasiliensis ATCC 700358
000284035.1	Nocardia cyriacigeorgica GUH-2
000009805.1	Nocardia farcinica IFM 10152
001182745.2	Nocardia farcinica NCTC11134
000523235.1	Nocardia nova SH22a
000015265.1	Nocardioides sp. JS614
000294515.1	Nocardiopsis alba ATCC BAA-2165
000092985.1	Nocardiopsis dassonvillei subsp. dassonvillei DSM 43111
000332115.1	Nonlabens dokdonensis DSW-6
001430865.1	Nonlabens sp. MIC269
000196515.1	'Nostoc azollae' 0708
000020025.1	Nostoc punctiforme PCC 73102
000316625.1	Nostoc sp. PCC 7107
000009705.1	Nostoc sp. PCC 7120
000316645.1	Nostoc sp. PCC 7524
000013325.1	Novosphingobium aromaticivorans DSM 12444
000767465.1	Novosphingobium pentaromativorans US6-1
000253255.1	Novosphingobium sp. PP1Y
001267175.1	Oblitimonas alkaliphila B4199
001267195.1	Oblitimonas alkaliphila C6819
001267215.1	Oblitimonas alkaliphila C6918
001267235.1	Oblitimonas alkaliphila D2441
001267255.1	Oblitimonas alkaliphila D3318
001267275.1	Oblitimonas alkaliphila E1086
001267295.1	Oblitimonas alkaliphila E1148
000243075.1	Oceanimonas sp. GK1
000183745.1	Oceanithermus profundus DSM 14977

000011245.1	Oceanobacillus iheyensis HTE831
000017405.1	Ochrobactrum anthropi ATCC 49188
000742955.1	Ochrobactrum anthropi OAB
000155675.2	Octadecabacter antarcticus 307
000155735.2	Octadecabacter arcticus 238
001187845.1	Octadecabacter temperatus SB1
000190535.1	Odoribacter splanchnicus DSM 20712
000241055.1	Oenococcus kitaharae DSM 17330
000014385.1	Oenococcus oeni PSU-1
000967895.1	Oleispira antarctica RB-8
000218585.1	Oligotropha carboxidovorans OM4
000021365.1	Oligotropha carboxidovorans OM5
001189515.2	Olsenella sp. oral taxon 807
000143845.1	Olsenella uli DSM 7084
000009845.1	Onion yellows phytoplasma OY-M
000242935.3	Opitutaceae bacterium TAV5
000019965.1	Opitutus terrae PB90-1
000063545.1	Orientia tsutsugamushi str. Boryong
000010205.1	Orientia tsutsugamushi str. Ikeda
000265465.1	Ornithobacterium rhinotracheale DSM 15997
000756505.1	Ornithobacterium rhinotracheale ORT-UMN 88
000317105.1	Oscillatoria acuminata PCC 6304
000317475.1	Oscillatoria nigro-viridis PCC 7112
000283575.1	Oscillibacter valericigenes Sjm18-20
001262075.1	Ottowia sp. oral taxon 894
000236705.1	Owenweeksia hongkongensis DSM 17368
000014925.1	Paenarthrobacter aurescens TC1
000961095.1	Paenibacillus beijingensis DSM 24997
000758665.1	Paenibacillus borealis DSM 13188
001421015.1	Paenibacillus bovis BD3526
000993825.1	Paenibacillus durus ATCC 35681
000756615.1	Paenibacillus durus DSM 1735

000758705.1	Paenibacillus graminis DSM 15220
000511405.1	Paenibacillus larvae subsp. larvae DSM 25430
000250655.1	Paenibacillus mucilaginosus 3016
000258535.2	Paenibacillus mucilaginosus K02
000218915.1	Paenibacillus mucilaginosus KNP414
001465255.1	Paenibacillus naphthalenovorans 32O-Y
000758725.1	Paenibacillus odorifer DSM 15391
001272655.1	Paenibacillus peoriae HS311
000507205.2	Paenibacillus polymyxa CR1
000146875.2	Paenibacillus polymyxa E681
000237325.1	Paenibacillus polymyxa M1
000164985.2	Paenibacillus polymyxa SC2
000597985.1	Paenibacillus polymyxa SQR-21
000819665.1	Paenibacillus polymyxa Sb3-1
000981585.1	Paenibacillus riograndensis SBR5
000612505.1	Paenibacillus sabinae T27
001465275.1	Paenibacillus sp. 32O-W
000758525.1	Paenibacillus sp. FSL H7-0357
000758545.1	Paenibacillus sp. FSL H7-0737
000758565.1	Paenibacillus sp. FSL P4-0081
000758585.1	Paenibacillus sp. FSL R5-0345
000758605.1	Paenibacillus sp. FSL R5-0912
000758625.1	Paenibacillus sp. FSL R7-0273
000758645.1	Paenibacillus sp. FSL R7-0331
000949425.1	Paenibacillus sp. IHBB 10380
001447315.1	Paenibacillus sp. IHB B 3084
000023585.1	Paenibacillus sp. JDR-2
000024685.1	Paenibacillus sp. Y412MC10
000758685.1	Paenibacillus stellifer DSM 14472
000235585.1	Paenibacillus terrae HPL-003
000725425.1	Palaeococcus pacificus DY20341
000183135.1	Paludibacter propionicigenes WB4

001027265.1	Pandoraea apista AU2161
001465595.1	Pandoraea apista DSM 16535
001010785.1	Pandoraea apista TF80G25
000826965.3	Pandoraea apista TF81F4
001029105.1	Pandoraea faecigallinarum DSM 23572
001465545.1	Pandoraea norimbergensis DSM 11628
000972785.2	Pandoraea oxalativorans DSM 23570
000767615.2	Pandoraea pnomenusa
000590495.2	Pandoraea pnomenusa 3kgm
000604065.2	Pandoraea pnomenusa RB38
000815105.1	Pandoraea pulmonicola DSM 16583
000814845.1	Pandoraea sputorum DSM 21091
000934605.1	Pandoraea vervacti NS15
001484065.1	Pannonibacter phragmitetus 31801
000270125.1	Pantoea ananatis AJ13355
000025405.2	Pantoea ananatis LMG 20103
000283875.1	Pantoea ananatis LMG 5342
000233595.1	Pantoea ananatis PA13
000759475.1	Pantoea rwandensis ND04
000175935.2	Pantoea sp. At-9b
000784875.3	Pantoea sp. PSNIH1
000784965.1	Pantoea sp. PSNIH2
000148935.1	Pantoea vagans C9-1
000012845.1	Parabacteroides distasonis ATCC 8503
000522545.2	Paraburkholderia caribensis MBA4
001449005.1	Paraburkholderia caribensis MWAP64
000961515.1	Paraburkholderia fungorum ATCC BAA-463
000300095.1	Paraburkholderia phenoliruptrix BR3459a
000020045.1	Paraburkholderia phymatum STM815
000020125.1	Paraburkholderia phytofirmans PsJN
000198775.1	Paraburkholderia rhizoxinica HKI 454
000013645.1	Paraburkholderia xenovorans LB400

000253035.1	Parachlamydia acanthamoebae UV-7
000444995.1	Paracoccus aminophilus JCM 7686
000203895.1	Paracoccus denitrificans PD1222
000347635.1	Paraglaciecola psychrophila 170
000284415.2	Pararhodospirillum photometricum DSM 122
001042675.1	Parascardovia denticolens DSM 10105 = JCM 12538
000017565.1	Parvibaculum lavamentivorans DS-1
000800295.1	Parvimonas micra KCOM 1535 (=ChDC B708)
000152825.2	Parvularcula bermudensis HTCC2503
000234745.1	Pasteurella multocida 36950
000754275.1	Pasteurella multocida ATCC 43137
000973565.1	Pasteurella multocida OH1905
000973525.1	Pasteurella multocida subsp. multocida OH4807
000259545.1	Pasteurella multocida subsp. multocida str. 3480
000512395.1	Pasteurella multocida subsp. multocida str. HB03
000255915.1	Pasteurella multocida subsp. multocida str. HN06
000006825.1	Pasteurella multocida subsp. multocida str. Pm70
001477625.1	Paucibacter sp. KCTC 42545
000740965.1	Pectobacterium atrosepticum 21A
000696465.1	Pectobacterium atrosepticum JG10-08
000011605.1	Pectobacterium atrosepticum SCRI1043
000023605.1	Pectobacterium carotovorum subsp. carotovorum PC1
000294535.1	Pectobacterium carotovorum subsp. carotovorum PCC21
000769535.1	Pectobacterium carotovorum subsp. odoriferum
000260925.1	Pectobacterium sp. SCC3193
000024645.1	Pectobacterium wasabiae WPP163
000237995.2	Pediococcus claussenii ATCC BAA-344
000014505.1	Pediococcus pentosaceus ATCC 25745
000496265.1	Pediococcus pentosaceus SL4
000023825.1	Pedobacter heparinus DSM 2366
001412655.1	Pedobacter sp. PACM 27299
000230555.1	Pelagibacterium halotolerans B2

000012885.1	Pelobacter carbinolicus DSM 2380
000015045.1	Pelobacter propionicus DSM 2379
000020645.1	Pelodictyon phaeoclathratiforme BU-1
000271665.2	Pelosinus fermentans JBW45
000725345.1	Pelosinus sp. UFO1
000010565.1	Pelotomaculum thermopropionicum SI
000009205.1	Peptoclostridium difficile 630
000953275.1	Peptoclostridium difficile 630Derm
001077535.1	Peptoclostridium difficile ATCC 9689 = DSM 1296
000211235.1	Peptoclostridium difficile BI1
000085225.1	Peptoclostridium difficile CD196
000210435.1	Peptoclostridium difficile M120
001457575.1	Peptoclostridium difficile NCTC13307
001447175.1	Peptoclostridium difficile Z31
000952975.1	Peptoniphilus sp. 1-1
000021565.1	Persephonella marina EX-H1
001308105.1	Persicobacter sp. JZB09
000018605.1	Petrotoga mobilis SJ95
000154745.2	Phaeobacter gallaeciensis 2.10
000511385.1	Phaeobacter gallaeciensis DSM 26640
000154765.2	Phaeobacter inhibens DSM 17395
000017265.1	Phenylobacterium zucineum HLK1
000940995.1	Photobacterium gaetbulicola Gung47
000196475.1	Photorhabdus asymbiotica subsp. asymbiotica ATCC 43949
001010285.1	Photorhabdus temperata subsp. thracensis
000284115.1	Phycisphaera mikurensis NBRC 102666
000008265.1	Picrophilus torridus DSM 9790
000785495.1	Pimelobacter simplex VKM Ac-2033D
000025185.1	Pirellula staleyi DSM 6068
000300295.3	Piscirickettsia salmonis LF-89 = ATCC VR-1361
000756435.3	Piscirickettsia salmonis PM15972A1
000756415.3	Piscirickettsia salmonis PM32597B1

000092105.1	Planctopirus limnophila DSM 3776
000738435.1	Planktomarina temperata RCA23
001465835.1	Planococcus kocurii ATCC 43650
001465795.1	Planococcus rifietoensis M8
000785555.1	Planococcus sp. PAMC 21323
000180175.2	Plautia stali symbiont
000317025.1	Pleurocapsa sp. PCC 7327
000757785.1	Pluralibacter gergoviae FB2
000152945.2	Polaribacter sp. MED152
000015505.1	Polaromonas naphthalenivorans CJ2
000013865.1	Polaromonas sp. JS666
001017435.1	[Polyangium] brachysporum DSM 7029
000192745.1	Polymorphum gilvum SL003B-26A1
000973625.1	Polynucleobacter asymbioticus MWH-MoK4
000016345.1	Polynucleobacter asymbioticus QLW-P1DMWA-1
000019745.1	Polynucleobacter necessarius subsp. necessarius STIR1
000973725.1	Pontibacter korlensis X14-1T
000212375.1	Porphyromonas asaccharolytica DSM 20707
001314265.1	Porphyromonas gingivalis 381
001263815.1	Porphyromonas gingivalis A7436
001444325.1	Porphyromonas gingivalis A7A1-28
001274615.1	Porphyromonas gingivalis AJW4
000010505.1	Porphyromonas gingivalis ATCC 33277
000270225.1	Porphyromonas gingivalis TDC60
000007585.1	Porphyromonas gingivalis W83
001026985.1	Pragia fontium 24613
000242335.3	Prevotella dentalis DSM 3688
000193395.1	Prevotella denticola F0289
001444445.1	Prevotella enoeca F0113
001262015.1	Prevotella fusca JCM 17724
000261025.1	Prevotella intermedia 17
000144405.1	Prevotella melaninogenica ATCC 25845

000025925.1	Prevotella ruminicola 23
000163055.2	Prevotella sp. oral taxon 299 str. F0039
000015645.1	Prochlorococcus marinus str. AS9601
000018065.1	Prochlorococcus marinus str. MIT 9215
000015965.1	Prochlorococcus marinus str. MIT 9301
000015705.1	Prochlorococcus marinus str. MIT 9303
000012645.1	Prochlorococcus marinus str. MIT 9312
000011485.1	Prochlorococcus marinus str. MIT 9313
000015665.1	Prochlorococcus marinus str. MIT 9515
000015685.1	Prochlorococcus marinus str. NATL1A
000012465.1	Prochlorococcus marinus str. NATL2A
000007925.1	Prochlorococcus marinus subsp. marinus str. CCMP1375
000011465.1	Prochlorococcus marinus subsp. pastoris str. CCMP1986
000757845.1	Prochlorococcus sp. MIT 0604
000757865.1	Prochlorococcus sp. MIT 0801
000310065.1	Propionibacterium acidipropionici ATCC 4875
001441165.1	Propionibacterium acidipropionici CGMCC 1.2230
000213155.1	Propionibacterium acnes 266
000217615.1	Propionibacterium acnes 6609
000231215.1	Propionibacterium acnes ATCC 11828
000302515.1	Propionibacterium acnes C1
000376705.1	Propionibacterium acnes HL096PA1
001281065.1	Propionibacterium acnes KCOM 1861 (= ChDC B594)
000008345.1	Propionibacterium acnes KPA171202
001469655.1	Propionibacterium acnes PA_12_1_L1
001469565.1	Propionibacterium acnes PA_15_1_R1
001469615.1	Propionibacterium acnes PA_15_2_L1
001469595.1	Propionibacterium acnes PA_21_1_L1
000025765.1	Propionibacterium acnes SK137
000240035.1	Propionibacterium acnes TypeIA2 P.acn17
000240055.1	Propionibacterium acnes TypeIA2 P.acn31
000240015.1	Propionibacterium acnes TypeIA2 P.acn33

000709495.1	Propionibacterium acnes hdn-1
000367205.1	Propionibacterium avidum 44067
000940845.1	Propionibacterium freudenreichii subsp. freudenreichii
000091725.1	Propionibacterium freudenreichii subsp. shermanii CIRM-BIA1
000277715.1	Propionibacterium propionicum F0230a
000020625.1	Prosthecochloris aestuarii DSM 271
000444425.1	Proteus mirabilis BB2000
001281545.1	Proteus mirabilis CYPM1
000069965.1	Proteus mirabilis HI4320
001281565.1	Proteus vulgaris CYPV1
001499655.1	Protochlamydia naegleriophila KNic
000754345.1	Providencia stuartii ATCC 33672
000259175.1	Providencia stuartii MRSN 2154
000317065.1	Pseudanabaena sp. PCC 7367
000022025.1	Pseudarthrobacter chlorophenolicus A6
000189535.1	Pseudarthrobacter phenanthrenivorans Sphe3
001484605.1	Pseudarthrobacter sulfonivorans Ar51
000014225.1	Pseudoalteromonas atlantica T6c
000026085.1	Pseudoalteromonas haloplanktis TAC125
001455325.1	Pseudoalteromonas issachenkonii KCTC 12958
001444405.1	Pseudoalteromonas phenolica KCTC 12086
001482385.1	Pseudoalteromonas rubra SCSIO 6842
000310105.2	Pseudoalteromonas sp. Bsw20308
000788395.1	Pseudoalteromonas sp. OCN003
000184065.1	Pseudoalteromonas sp. SM9913
001465295.1	Pseudoalteromonas translucida KMM 520
000283535.1	Pseudogulbenkiania sp. NH8B
001444425.1	Pseudohongiella spirulinae KCTC 32221
001482325.1	Pseudomonas aeruginosa 12-4-4(59)
000220025.3	Pseudomonas aeruginosa AES-1R
000359505.1	Pseudomonas aeruginosa B136-33
000981825.1	Pseudomonas aeruginosa Carb01 63

001465155.1	Pseudomonas aeruginosa Cu1510
000271365.1	Pseudomonas aeruginosa DK2
001045685.1	Pseudomonas aeruginosa DSM 50071
000816985.1	Pseudomonas aeruginosa F22031
001077475.1	Pseudomonas aeruginosa F9676
000829885.1	Pseudomonas aeruginosa FRD1
000508765.1	Pseudomonas aeruginosa LES431
000026645.1	Pseudomonas aeruginosa LESB58
000226155.1	Pseudomonas aeruginosa M18
000504045.1	Pseudomonas aeruginosa MTB-1
000829275.1	Pseudomonas aeruginosa NCGM1900
000829255.1	Pseudomonas aeruginosa NCGM1984
000284555.1	Pseudomonas aeruginosa NCGM2.S1
001457615.1	Pseudomonas aeruginosa NCTC10332
000496605.2	Pseudomonas aeruginosa PA1
000496645.1	Pseudomonas aeruginosa PA1R
001293085.1	Pseudomonas aeruginosa PA1RG
000017205.1	Pseudomonas aeruginosa PA7
000168335.1	Pseudomonas aeruginosa PACS2
000006765.1	Pseudomonas aeruginosa PAO1
001291345.2	Pseudomonas aeruginosa PAO1_Orsay
000414035.1	Pseudomonas aeruginosa RP73
000510305.1	Pseudomonas aeruginosa SCV20265
000014625.1	Pseudomonas aeruginosa UCBPP-PA14
001447845.1	Pseudomonas aeruginosa VA-134
000473745.3	Pseudomonas aeruginosa VRFPA04
000524595.1	Pseudomonas aeruginosa YL84
000746525.1	Pseudomonas alkylphenolia KL28
000818015.1	Pseudomonas balearica DSM 6083
000585995.1	Pseudomonas brassicacearum DF41
001449085.1	Pseudomonas brassicacearum LBUM300
000194805.1	Pseudomonas brassicacearum subsp. brassicacearum NFM421

000698865.1	Pseudomonas chlororaphis PA23
000963835.1	Pseudomonas chlororaphis PCL1606
001023535.1	Pseudomonas chlororaphis UFB2
000761195.1	Pseudomonas chlororaphis subsp. aurantiaca
000517305.1	Pseudomonas cichorii JBC1
000759535.1	Pseudomonas cremoricolorata ND07
000349845.1	Pseudomonas denitrificans ATCC 13867
000026105.1	Pseudomonas entomophila L48
000262325.2	Pseudomonas fluorescens A506
000237065.1	Pseudomonas fluorescens F113
001307275.1	Pseudomonas fluorescens FW300-N2C3
001307155.1	Pseudomonas fluorescens FW300-N2E3
000968415.1	Pseudomonas fluorescens LBUM223
000293885.3	Pseudomonas fluorescens NCIMB 11764
000934565.1	Pseudomonas fluorescens PCL1751
000963495.1	Pseudomonas fluorescens PICF7
000012445.1	Pseudomonas fluorescens Pf0-1
000009225.1	Pseudomonas fluorescens SBW25
000730425.1	Pseudomonas fluorescens UK4
000213805.1	Pseudomonas fulva 12-X
000689415.1	Pseudomonas knackmussii B13
000257545.3	Pseudomonas mandelii JR-1
000204295.1	Pseudomonas mendocina NK-01
000016565.1	Pseudomonas mendocina ymp
000510285.1	Pseudomonas monteilii SB3078
000510325.1	Pseudomonas monteilii SB3101
000498975.2	Pseudomonas mosselii SJ10
000800255.1	Pseudomonas parafulva CRS01-1
000831585.1	Pseudomonas plecoglossicida NyZ12
000336465.1	Pseudomonas poae RE*1-1-14
000397205.1	Pseudomonas protegens CHA0
000828695.1	Pseudomonas protegens Cab57

000012265.1	Pseudomonas protegens Pf-5
000953455.1	Pseudomonas pseudoalcaligenes
000297075.2	Pseudomonas pseudoalcaligenes CECT 5344
000183645.1	Pseudomonas putida BIRD-1
000691565.1	Pseudomonas putida DLL-E4
000281215.1	Pseudomonas putida DOT-T1E
000016865.1	Pseudomonas putida F1
000019125.1	Pseudomonas putida GB-1
000410575.1	Pseudomonas putida H8234
000325725.1	Pseudomonas putida HB3267
000007565.1	Pseudomonas putida KT2440
000412675.1	Pseudomonas putida NBRC 14164
000264665.1	Pseudomonas putida ND6
000495455.2	Pseudomonas putida S12
000498395.3	Pseudomonas putida S13.1.2
000219705.1	Pseudomonas putida S16
000019445.1	Pseudomonas putida W619
000412695.1	Pseudomonas resinovorans NBRC 106553
000761155.1	Pseudomonas rhizosphaerae DSM 16299
000012205.1	Pseudomonas savastanoi pv. phaseolicola 1448A
000756775.2	Pseudomonas sp. 20_BN
001007005.1	Pseudomonas sp. CCOS 191
000511325.1	Pseudomonas sp. FGI182
001294575.1	Pseudomonas sp. L10.10
000931465.1	Pseudomonas sp. MRSN12121
000829415.1	Pseudomonas sp. StFLB209
000508205.1	Pseudomonas sp. TKP
000316175.1	Pseudomonas sp. UW4
000494915.1	Pseudomonas sp. VLB120
000661915.1	Pseudomonas stutzeri 19SMN4
000590475.1	Pseudomonas stutzeri 28a24
000013785.1	Pseudomonas stutzeri A1501

000219605.1	Pseudomonas stutzeri ATCC 17588 = LMG 11199
000267545.1	Pseudomonas stutzeri CCUG 29243
000279165.1	Pseudomonas stutzeri DSM 10701
000195105.1	Pseudomonas stutzeri DSM 4166
000327065.1	Pseudomonas stutzeri RCH2
001038645.1	Pseudomonas stutzeri SLG510A3-8
000452705.3	Pseudomonas syringae CC1557
001281365.1	Pseudomonas syringae UMAF0158
000648735.2	Pseudomonas syringae pv. actinidiae ICMP 18884
001482725.1	Pseudomonas syringae pv. lapsa
000988485.1	Pseudomonas syringae pv. syringae B301D
000012245.1	Pseudomonas syringae pv. syringae B728a
000988395.1	Pseudomonas syringae pv. syringae HS191
000007805.1	[Pseudomonas syringae] pv. tomato str. DC3000
001186335.1	Pseudomonas trivialis IHBB745
000196675.1	Pseudonocardia dioxanivorans CB1190
001294605.1	Pseudonocardia sp. AL041005-10
001420975.1	Pseudonocardia sp. EC080610-09
001420995.1	Pseudonocardia sp. EC080619-01
001294425.1	Pseudonocardia sp. EC080625-04
001294645.1	Pseudonocardia sp. HH130629-09
000190735.2	Pseudopedobacter saltans DSM 12145
000504085.1	Pseudothermotoga elfii DSM 9442 = NBRC 107921
000504105.1	Pseudothermotoga hypogea DSM 11164 = NBRC 106472
000017865.1	Pseudothermotoga lettingae TMO
000217815.1	Pseudothermotoga thermarum DSM 5069
000233915.4	Pseudoxanthomonas spadix BD-a59
000185965.1	Pseudoxanthomonas suwonensis 11-1
000972865.1	Pseudoxanthomonas suwonensis J1
000012305.1	Psychrobacter arcticus 273-4
000013905.1	Psychrobacter cryohalolentis K5
000418305.1	Psychrobacter sp. G

000016885.1	Psychrobacter sp. PRwf-1
001298525.1	Psychrobacter urativorans R310.10B
000153485.2	Psychroflexus torquis ATCC 700755
000015285.1	Psychromonas ingrahamii 37
000153405.2	Psychromonas sp. CNPT3
000209655.1	Pusillimonas sp. T7-7
000007225.1	Pyrobaculum aerophilum str. IM2
000016385.1	Pyrobaculum arsenaticum DSM 13514
000015805.1	Pyrobaculum calidifontis JCM 11548
000234805.1	Pyrobaculum ferrireducens 1860
000015205.1	Pyrobaculum islandicum DSM 4184
000019805.1	Pyrobaculum neutrophilum V24Sta
000247545.1	Pyrobaculum oguniense TE7
001189275.1	Pyrobaculum sp. WP30
000275605.1	Pyrococcus furiosus COM1
000007305.1	Pyrococcus furiosus DSM 3638
000011105.1	Pyrococcus horikoshii OT3
000211475.1	Pyrococcus sp. NA2
000263735.1	Pyrococcus sp. ST04
000215995.1	Pyrococcus yayanosii CH1
001412615.1	Pyrodictium delaneyi Su06
000223395.1	Pyrolobus fumarii 1A
000255535.1	Rahnella aquatilis HX2
000187705.1	Rahnella sp. Y9602
000009285.2	Ralstonia eutropha H16
000203875.1	Ralstonia eutropha JMP134
000954135.2	Ralstonia mannitolilytica SN82F48
000023425.1	Ralstonia pickettii 12D
000020205.1	Ralstonia pickettii 12J
000471925.1	Ralstonia pickettii DTP0602
000009125.1	Ralstonia solanacearum GMI1000
000283475.1	Ralstonia solanacearum PSI07

000215325.1	Ralstonia solanacearum Po82
001299555.1	Ralstonia solanacearum UY031
001267515.1	Ralstonia solanacearum YC45
000215705.1	Ramlibacter tataouinensis TTB310
000367425.1	Raoultella ornithinolytica B6
000829965.1	Raoultella ornithinolytica S12
001455225.1	Raoultella ornithinolytica Yangling I2
000875675.1	Rathayibacter toxicus 70137
001465855.1	Rathayibacter toxicus WAC3373
000018885.1	Renibacterium salmoninarum ATCC 33209
000092045.1	Rhizobium etli CFN 42
000020265.1	Rhizobium etli CIAT 652
000698845.1	Rhizobium etli bv. mimosae str. IE4771
000442435.1	Rhizobium etli bv. mimosae str. Mim1
000816125.1	Rhizobium etli bv. phaseoli str. IE4803
000816845.1	Rhizobium gallicum bv. gallicum R602
000520875.1	Rhizobium leguminosarum bv. trifolii CB782
000023185.1	Rhizobium leguminosarum bv. trifolii WSM1325
000517605.1	Rhizobium leguminosarum bv. trifolii WSM1689
000021345.1	Rhizobium leguminosarum bv. trifolii WSM2304
000009265.1	Rhizobium leguminosarum bv. viciae 3841
000499645.1	Rhizobium sp. IRBG74
000577275.1	Rhizobium sp. LPU83
000967425.1	Rhizobium sp. NT-26
000330885.1	Rhizobium tropici CIAT 899
000230695.3	Rhodanobacter denitrificans 2APBS1
000021865.1	Rhodobacter capsulatus SB 1003
000273405.1	Rhodobacter sphaeroides 2.4.1
000016405.1	Rhodobacter sphaeroides ATCC 17025
000015985.1	Rhodobacter sphaeroides ATCC 17029
000021005.1	Rhodobacter sphaeroides KD131
000212605.1	Rhodobacter sphaeroides WS8N

000982715.1	Rhodococcus aetherivorans IcdP1
000196695.1	Rhodococcus equi 103S
000975175.1	Rhodococcus erythropolis BG43
000454045.1	Rhodococcus erythropolis CCM2595
000010105.1	Rhodococcus erythropolis PR4
000696675.2	Rhodococcus erythropolis R138
000014565.1	Rhodococcus jostii RHA1
000010805.1	Rhodococcus opacus B4
000599545.1	Rhodococcus opacus PD630
000511305.1	Rhodococcus pyridinivorans SB3094
000954115.1	Rhodococcus sp. B7740
000934545.1	Rhodocyclaceae bacterium PG1-Ca6
000013605.1	Rhodoferax ferrireducens T118
000699505.1	Rhodoluna lacicola MWH-Ta8
000166055.1	Rhodomicrobium vannielii ATCC 17100
000014825.1	Rhodopseudomonas palustris BisA53
000013745.1	Rhodopseudomonas palustris BisB18
000013685.1	Rhodopseudomonas palustris BisB5
000177255.2	Rhodopseudomonas palustris DX-1
000013365.1	Rhodopseudomonas palustris HaA2
000020445.1	Rhodopseudomonas palustris TIE-1
000016185.1	Rhodospirillum centenum SW
000013085.1	Rhodospirillum rubrum ATCC 11170
000225955.1	Rhodospirillum rubrum F11
000024845.1	Rhodothermus marinus DSM 4252
000224745.1	Rhodothermus marinus SG0.5JP17-172
000023005.1	Rickettsia africae ESF-5
000018205.1	Rickettsia akari str. Hartford
000284155.1	Rickettsia australis str. Cutlack
000018245.1	Rickettsia bellii OSU 85-389
000012385.1	Rickettsia bellii RML369-C
000283915.1	Rickettsia canadensis str. CA410

000014345.1	Rickettsia canadensis str. McKiel
000007025.1	Rickettsia conorii str. Malish 7
000012145.1	Rickettsia felis URRWXCal2
000221205.1	Rickettsia heilongjiangensis 054
000255355.1	Rickettsia helvetica C9P9
000283595.1	Rickettsia japonica YH
000016625.1	Rickettsia massiliae MTU5
000499665.2	Rickettsia monacensis
000284175.1	Rickettsia montanensis str. OSU 85-930
000284195.1	Rickettsia parkeri str. Portsmouth
000021525.1	Rickettsia peacockii str. Rustic
000283995.1	Rickettsia philipii str. 364D
000367405.1	Rickettsia prowazekii str. Breinl
000277205.1	Rickettsia prowazekii str. BuV67-CWPP
000277165.1	Rickettsia prowazekii str. Chernikova
000277245.1	Rickettsia prowazekii str. GvV257
000277185.1	Rickettsia prowazekii str. Katsinyian
000363905.1	Rickettsia prowazekii str. NMRC Madrid E
000022785.1	Rickettsia prowazekii str. Rp22
000277265.1	Rickettsia prowazekii str. RpGvF24
001442475.1	Rickettsia rhipicephali HJ#5
000284075.1	Rickettsia rhipicephali str. 3-7-female6-CWPP
000283795.1	Rickettsia rickettsii str. Arizona
000283955.1	Rickettsia rickettsii str. Brazil
000283775.1	Rickettsia rickettsii str. Colombia
000283815.1	Rickettsia rickettsii str. Hino
000283935.1	Rickettsia rickettsii str. Hlp#2
000017445.3	Rickettsia rickettsii str. Iowa
000831545.1	Rickettsia rickettsii str. Morgan
000831525.1	Rickettsia rickettsii str. R
000018225.1	Rickettsia rickettsii str. 'Sheila Smith'
000166935.1	Rickettsia sibirica 246

000237845.1	Rickettsia slovaca 13-B
000252365.1	Rickettsia slovaca str. D-CWPP
000277305.1	Rickettsia typhi str. B9991CWPP
000277285.1	Rickettsia typhi str. TH1527
000008045.1	Rickettsia typhi str. Wilmington
000746585.2	Rickettsiales bacterium Ac37b
000183155.1	Riemerella anatipestifer ATCC 11845 = DSM 15868
000734055.1	Riemerella anatipestifer CH3
000295655.1	Riemerella anatipestifer RA-CH-1
000331695.1	Riemerella anatipestifer RA-CH-2
000191565.1	Riemerella anatipestifer RA-GD
001077795.1	Riemerella anatipestifer Yb2
000316665.1	Rivularia sp. PCC 7116
000024125.1	Robiginitalea biformata HTCC2501
001483865.1	Roseateles depolymerans KCTC 42856
000225345.1	Roseburia hominis A2-183
000590925.1	Roseibacterium elongatum DSM 19469
000017805.1	Roseiflexus castenholzii DSM 13941
000016665.1	Roseiflexus sp. RS-1
000014045.1	Roseobacter denitrificans OCh 114
000154785.2	Roseobacter litoralis Och 149
000164695.2	Rothia dentocariosa ATCC 17931
000011025.1	Rothia mucilaginosa DY-18
000165715.3	Rubinisphaera brasiliensis DSM 5305
000284255.1	Rubrivivax gelatinosus IL144
000661895.1	Rubrobacter radiotolerans RSPS-4
000014185.1	Rubrobacter xylanophilus DSM 9941
000011965.2	Ruegeria pomeroyi DSS-3
000014065.1	Ruegeria sp. TM1040
001078055.1	Rufibacter sp. DG31D
001310085.1	Rufibacter tibetensis 1351
000015865.1	Ruminiclostridium thermocellum ATCC 27405

000184925.1	Ruminiclostridium thermocellum DSM 1313
000179635.2	Ruminococcus albus 7 = DSM 20455
000723465.1	Ruminococcus bicirculans 80/3
000218895.1	Runella slithyformis DSM 19594
000023865.1	Saccharomonospora viridis DSM 43017
000013665.1	Saccharophagus degradans 2-40
000062885.1	Saccharopolyspora erythraea NRRL 2338
000328705.1	Saccharothrix espanaensis DSM 44229
000403645.1	Salinarchaeum sp. Harcht-Bsk1
000013045.1	Salinibacter ruber DSM 13855
000090405.1	Salinibacter ruber M8
001005905.1	Salinicoccus halodurans H3B36
000507245.1	Salinispira pacifica L21-RPul-D2
000018265.1	Salinispora arenicola CNS-205
000016425.1	Salinispora tropica CNB-440
000439255.1	Salmonella bongori N268-08
000252995.1	Salmonella bongori NCTC 12419
000709535.1	Salmonella bongori serovar 48:z41:-- str. RKS3044
000756465.1	Salmonella enterica subsp. arizonae serovar 62:z36:- str. RKS2983
000018625.1	Salmonella enterica subsp. arizonae serovar 62:z4,z23:-
001006525.1	Salmonella enterica subsp. enterica
000430165.1	Salmonella enterica subsp. enterica serovar 4,[5],12:i:- str. 08-1736
000487915.2	Salmonella enterica subsp. enterica serovar Abaetetuba str. ATCC 35640
000487615.2	Salmonella enterica subsp. enterica serovar Abony str. 0014
000503845.1	Salmonella enterica subsp. enterica serovar Agona str. 24249
000484195.2	Salmonella enterica subsp. enterica serovar Agona str. 460004 2-1
000020885.1	Salmonella enterica subsp. enterica serovar Agona str. SL483
001447095.1	Salmonella enterica subsp. enterica serovar Anatum
000487575.2	Salmonella enterica subsp. enterica serovar Anatum str. ATCC BAA-1592
000940895.1	Salmonella enterica subsp. enterica serovar Anatum str. CDC 06-0532
000963535.1	Salmonella enterica subsp. enterica serovar Anatum str. USDA-ARS-USMARC-1175
000988525.1	Salmonella enterica subsp. enterica serovar Anatum str. USDA-ARS-USMARC-1735

000439415.1	Salmonella enterica subsp. enterica serovar Bareilly str. CFSAN000189
000487775.2	Salmonella enterica subsp. enterica serovar Bredeney str. CFSAN001080
000742815.1	Salmonella enterica subsp. enterica serovar Choleraesuis
000487295.2	Salmonella enterica subsp. enterica serovar Choleraesuis str. ATCC 10708
000008105.1	Salmonella enterica subsp. enterica serovar Choleraesuis str. SC-B67
000430125.1	Salmonella enterica subsp. enterica serovar Cubana str. CFSAN002050
000020925.1	Salmonella enterica subsp. enterica serovar Dublin str. CT_02021853
000612325.1	Salmonella enterica subsp. enterica serovar Enteritidis
000335875.2	Salmonella enterica subsp. enterica serovar Enteritidis str. 18569
000280315.2	Salmonella enterica subsp. enterica serovar Enteritidis str. 77-1427
000329365.2	Salmonella enterica subsp. enterica serovar Enteritidis str. CDC_2010K_0968
000623755.1	Salmonella enterica subsp. enterica serovar Enteritidis str. EC20090135
000623775.1	Salmonella enterica subsp. enterica serovar Enteritidis str. EC20090193
000623795.1	Salmonella enterica subsp. enterica serovar Enteritidis str. EC20090332
000626155.1	Salmonella enterica subsp. enterica serovar Enteritidis str. EC20090531
000626275.2	Salmonella enterica subsp. enterica serovar Enteritidis str. EC20090641
000626255.1	Salmonella enterica subsp. enterica serovar Enteritidis str. EC20090698
000626135.1	Salmonella enterica subsp. enterica serovar Enteritidis str. EC20090884
000626215.1	Salmonella enterica subsp. enterica serovar Enteritidis str. EC20100101
000626115.1	Salmonella enterica subsp. enterica serovar Enteritidis str. EC20100103
000831045.1	Salmonella enterica subsp. enterica serovar Enteritidis str. EC20100130
000831025.1	Salmonella enterica subsp. enterica serovar Enteritidis str. EC20100134
000626235.1	Salmonella enterica subsp. enterica serovar Enteritidis str. EC20110221
000623175.1	Salmonella enterica subsp. enterica serovar Enteritidis str. EC20110223
000626315.1	Salmonella enterica subsp. enterica serovar Enteritidis str. EC20110353
000626175.1	Salmonella enterica subsp. enterica serovar Enteritidis str. EC20110354
000626295.1	Salmonella enterica subsp. enterica serovar Enteritidis str. EC20110355
000623375.1	Salmonella enterica subsp. enterica serovar Enteritidis str. EC20110356
000623355.1	Salmonella enterica subsp. enterica serovar Enteritidis str. EC20110357
000623335.1	Salmonella enterica subsp. enterica serovar Enteritidis str. EC20110358
000623315.1	Salmonella enterica subsp. enterica serovar Enteritidis str. EC20110359
000623295.1	Salmonella enterica subsp. enterica serovar Enteritidis str. EC20110360

000623275.1	Salmonella enterica subsp. enterica serovar Enteritidis str. EC20110361
000626375.1	Salmonella enterica subsp. enterica serovar Enteritidis str. EC20111095
000626355.1	Salmonella enterica subsp. enterica serovar Enteritidis str. EC20111174
000626335.1	Salmonella enterica subsp. enterica serovar Enteritidis str. EC20111175
000624395.2	Salmonella enterica subsp. enterica serovar Enteritidis str. EC20120002
000623195.2	Salmonella enterica subsp. enterica serovar Enteritidis str. EC20120005
000626195.1	Salmonella enterica subsp. enterica serovar Enteritidis str. EC20120008
000626395.1	Salmonella enterica subsp. enterica serovar Enteritidis str. EC20120009
000624155.1	Salmonella enterica subsp. enterica serovar Enteritidis str. EC20120916
000626415.1	Salmonella enterica subsp. enterica serovar Enteritidis str. EC20120929
000623055.2	Salmonella enterica subsp. enterica serovar Enteritidis str. EC20121175
000623075.1	Salmonella enterica subsp. enterica serovar Enteritidis str. EC20121176
000623095.1	Salmonella enterica subsp. enterica serovar Enteritidis str. EC20121178
000623115.1	Salmonella enterica subsp. enterica serovar Enteritidis str. EC20121179
000623135.1	Salmonella enterica subsp. enterica serovar Enteritidis str. EC20121180
000009505.1	Salmonella enterica subsp. enterica serovar Enteritidis str. P125109
000626475.1	Salmonella enterica subsp. enterica serovar Enteritidis str. SA20084644
000626495.1	Salmonella enterica subsp. enterica serovar Enteritidis str. SA20084824
000623155.1	Salmonella enterica subsp. enterica serovar Enteritidis str. SA20093266
000626515.1	Salmonella enterica subsp. enterica serovar Enteritidis str. SA20094177
000626535.1	Salmonella enterica subsp. enterica serovar Enteritidis str. SA20094301
000009525.1	Salmonella enterica subsp. enterica serovar Gallinarum str. 287/91
000462995.1	Salmonella enterica subsp. enterica serovar Gallinarum/pullorum str. CDC1983-67
000235545.1	Salmonella enterica subsp. enterica serovar Gallinarum/pullorum str. RKS5078
001441205.1	Salmonella enterica subsp. enterica serovar Heidelberg
000430105.1	Salmonella enterica subsp. enterica serovar Heidelberg str. 41578
000258365.1	Salmonella enterica subsp. enterica serovar Heidelberg str. B182
000505705.1	Salmonella enterica subsp. enterica serovar Heidelberg str. CFSAN002064
000430085.2	Salmonella enterica subsp. enterica serovar Heidelberg str. CFSAN002069
000020705.1	Salmonella enterica subsp. enterica serovar Heidelberg str. SL476
000953495.1	Salmonella enterica subsp. enterica serovar Infantis
000341425.1	Salmonella enterica subsp. enterica serovar Javiana str. CFSAN001992

000188955.5	Salmonella enterica subsp. enterica serovar Montevideo str. 507440-20
000940975.1	Salmonella enterica subsp. enterica serovar Montevideo str. USDA-ARS-USMARC-1903
000973665.1	Salmonella enterica subsp. enterica serovar Montevideo str. USDA-ARS-USMARC-1921
000973685.1	Salmonella enterica subsp. enterica serovar Newport
000272755.3	Salmonella enterica subsp. enterica serovar Newport str. CVM 21538
000272715.3	Salmonella enterica subsp. enterica serovar Newport str. CVM 21550
000272795.2	Salmonella enterica subsp. enterica serovar Newport str. CVM 21554
000272775.3	Salmonella enterica subsp. enterica serovar Newport str. CVM 22425
000272895.3	Salmonella enterica subsp. enterica serovar Newport str. CVM 22462
000272735.3	Salmonella enterica subsp. enterica serovar Newport str. CVM 22513
000272835.3	Salmonella enterica subsp. enterica serovar Newport str. CVM N1543
000272815.2	Salmonella enterica subsp. enterica serovar Newport str. CVM N18486
000016045.1	Salmonella enterica subsp. enterica serovar Newport str. SL254
000940935.1	Salmonella enterica subsp. enterica serovar Newport str. USDA-ARS-USMARC-1927
000442415.1	Salmonella enterica subsp. enterica serovar Newport str. USMARC-S3124.1
000486765.2	Salmonella enterica subsp. enterica serovar Panama str. ATCC 7378
000818075.1	Salmonella enterica subsp. enterica serovar Paratyphi A
000026565.1	Salmonella enterica subsp. enterica serovar Paratyphi A str. AKU_12601
000011885.1	Salmonella enterica subsp. enterica serovar Paratyphi A str. ATCC 9150
000018705.1	Salmonella enterica subsp. enterica serovar Paratyphi B str. SPB7
000018385.1	Salmonella enterica subsp. enterica serovar Paratyphi C str. RKS4594
000330485.2	Salmonella enterica subsp. enterica serovar Pullorum str. ATCC 9120
000444445.1	Salmonella enterica subsp. enterica serovar Pullorum str. S06004
000020745.1	Salmonella enterica subsp. enterica serovar Schwarzengrund str. CVM19633
001457675.1	Salmonella enterica subsp. enterica serovar Senftenberg
000486445.2	Salmonella enterica subsp. enterica serovar Sloterdijk str. ATCC 15791
000486405.2	Salmonella enterica subsp. enterica serovar Tennessee str. TXSC_TXSC08-19
001305815.1	Salmonella enterica subsp. enterica serovar Thompson
000486365.2	Salmonella enterica subsp. enterica serovar Thompson str. ATCC 8391
000473275.1	Salmonella enterica subsp. enterica serovar Thompson str. RM6836
001302605.1	Salmonella enterica subsp. enterica serovar Typhi
000195995.1	Salmonella enterica subsp. enterica serovar Typhi str. CT18

000245535.1	Salmonella enterica subsp. enterica serovar Typhi str. P-stx-12
000007545.1	Salmonella enterica subsp. enterica serovar Typhi str. Ty2
000385905.1	Salmonella enterica subsp. enterica serovar Typhi str. Ty21a
000636135.1	Salmonella enterica subsp. enterica serovar Typhimurium
000022165.1	Salmonella enterica subsp. enterica serovar Typhimurium str. 14028S
000252875.1	Salmonella enterica subsp. enterica serovar Typhimurium str. 798
000973645.1	Salmonella enterica subsp. enterica serovar Typhimurium str. CDC 2011K-0870
000027025.1	Salmonella enterica subsp. enterica serovar Typhimurium str. D23580
000493675.1	Salmonella enterica subsp. enterica serovar Typhimurium str. DT104
000493535.1	Salmonella enterica subsp. enterica serovar Typhimurium str. DT2
000828595.1	Salmonella enterica subsp. enterica serovar Typhimurium str. L-3553
000006945.1	Salmonella enterica subsp. enterica serovar Typhimurium str. LT2
000210855.2	Salmonella enterica subsp. enterica serovar Typhimurium str. SL1344
000188735.1	Salmonella enterica subsp. enterica serovar Typhimurium str. ST4/74
000283735.1	Salmonella enterica subsp. enterica serovar Typhimurium str. T000240
000380325.1	Salmonella enterica subsp. enterica serovar Typhimurium str. U288
000213635.1	Salmonella enterica subsp. enterica serovar Typhimurium str. UK-1
000941015.1	Salmonella enterica subsp. enterica serovar Typhimurium str. USDA-ARS-USMARC-1899
000430145.3	Salmonella enterica subsp. enterica serovar Typhimurium var. 5- str. CFSAN001921
001409155.1	Salmonella enterica subsp. enterica serovar Weltevreden
000737325.2	Sandaracinus amylolyticus DSM 53668
000024925.1	Sanguibacter keddieii DSM 10542
000250635.1	Saprospira grandis str. Lewin
001042695.1	Scardovia inopinata JCM 12537
000024405.1	Sebaldella termitidis ATCC 33386
001007875.1	Sedimenticola thiotaurini SIP-G1
001430825.1	Sediminicola sp. YIK13
000092825.1	Segniliparus rotundus DSM 44985
000284095.1	Selenomonas ruminantium subsp. lactilytica TAM6421
001189555.1	Selenomonas sp. oral taxon 478
000208405.1	Selenomonas sputigena ATCC 35185
001006005.1	Serratia fonticola DSM 4576

000422085.1	Serratia liquefaciens ATCC 27592
000975245.1	Serratia liquefaciens HUMV-21
001417865.1	Serratia marcescens B3R3
001022215.1	Serratia marcescens CAV1492
000330865.1	Serratia marcescens FGI94
001280365.1	Serratia marcescens RSC-14
000828775.1	Serratia marcescens SM39
001294565.1	Serratia marcescens SmUNAM836
000336425.1	Serratia marcescens WW4
000513215.1	Serratia marcescens subsp. marcescens Db11
000176835.2	Serratia plymuthica 4Rx13
000214235.1	Serratia plymuthica AS9
000478545.1	Serratia plymuthica RVH1
000438825.1	Serratia plymuthica S13
000018085.1	Serratia proteamaculans 568
000214195.1	Serratia sp. AS12
000214805.1	Serratia sp. AS13
000695995.1	Serratia sp. FS14
000747565.1	Serratia sp. SCBI
000238975.1	Serratia symbiotica str. 'Cinara cedri'
000015245.1	Shewanella amazonensis SB2B
000147735.3	Shewanella baltica BA175
000215895.1	Shewanella baltica OS117
000015845.1	Shewanella baltica OS155
000017325.1	Shewanella baltica OS185
000018765.1	Shewanella baltica OS195
000021665.1	Shewanella baltica OS223
000178875.2	Shewanella baltica OS678
000013765.1	Shewanella denitrificans OS217
000014705.1	Shewanella frigidimarina NCIMB 400
000019185.1	Shewanella halifaxensis HAW-EB4
000016065.1	Shewanella loihica PV-4

000146165.2	Shewanella oneidensis MR-1
000018285.1	Shewanella pealeana ATCC 700345
000014885.1	Shewanella piezotolerans WP3
000169215.2	Shewanella putrefaciens 200
000016585.1	Shewanella putrefaciens CN-32
000018025.1	Shewanella sediminis HAW-EB3
000203935.1	Shewanella sp. ANA-3
000014685.1	Shewanella sp. MR-4
000014665.1	Shewanella sp. MR-7
000015185.1	Shewanella sp. W3-18-1
000091325.1	Shewanella violacea DSS12
000019525.1	Shewanella woodyi ATCC 51908
001027225.1	Shigella boydii ATCC 9210
000020185.1	Shigella boydii CDC 3083-94
000012025.1	Shigella boydii Sb227
000497505.1	Shigella dysenteriae 1617
000012005.1	Shigella dysenteriae Sd197
000953035.1	Shigella flexneri
000022245.1	Shigella flexneri 2002017
000743955.1	Shigella flexneri 2003036
000007405.1	Shigella flexneri 2a str. 2457T
000006925.2	Shigella flexneri 2a str. 301
000013585.1	Shigella flexneri 5 str. 8401
001021855.1	Shigella flexneri G1663
000743995.1	Shigella flexneri Shi06HN006
000283715.1	Shigella sonnei 53G
000092525.1	Shigella sonnei Ss046
000262305.1	Shimwellia blattae DSM 4481 = NBRC 105725
000941055.1	Siansivirga zeaxanthinifaciens CC-SAMT-1
000025705.1	Sideroxydans lithotrophicus ES-1
000305785.2	Simiduia agarivorans SA1 = DSM 21679
000237205.1	Simkania negevensis Z

000242455.3	Singulisphaera acidiphila DSM 18658
000018545.1	Sinorhizobium fredii NGR234
000017145.1	Sinorhizobium medicae WSM419
000006965.1	Sinorhizobium meliloti 1021
000346065.1	Sinorhizobium meliloti 2011
000147795.3	Sinorhizobium meliloti AK83
000147775.3	Sinorhizobium meliloti BL225C
000320385.2	Sinorhizobium meliloti GR4
000747295.1	Sinorhizobium meliloti RMO17
000304415.1	Sinorhizobium meliloti Rm41
000218265.1	Sinorhizobium meliloti SM11
000023885.1	Slackia heliotrinireducens DSM 20476
000973085.1	Sneathia amnii SN35
000600005.1	Snodgrassella alvi wkB2
000010085.1	Sodalis glossinidius str. 'morsitans'
000517425.1	Sodalis praecaptivus HS1
000271325.1	Solibacillus silvestris StLB046
000242635.3	Solitalea canadensis DSM 3403
000418325.1	Sorangium cellulosum So0157-2
000067165.1	Sorangium cellulosum So ce56
000024985.1	Sphaerobacter thermophilus DSM 20745
000208385.1	Sphaerochaeta coccoides DSM 17374
000190435.1	Sphaerochaeta globosa str. Buddy
000236685.1	Sphaerochaeta pleomorpha str. Grapes
000192845.1	Sphingobacterium sp. 21
000747525.1	Sphingobacterium sp. ML3W
001456115.1	Sphingobium baderi DE-13
000147835.3	Sphingobium chlorophenolicum L-1
000091125.1	Sphingobium japonicum UT26S
000283515.1	Sphingobium sp. SYK-6
000943805.1	Sphingobium sp. YBL2
000935025.1	Sphingomonas hengshuiensis WHSC-8

000512205.2	Sphingomonas sanxanigenens DSM 19645 = NX02
000347675.2	Sphingomonas sp. MM-1
000764535.1	Sphingomonas taxi ATCC 55669
000016765.1	Sphingomonas wittichii RW1
000013985.1	Sphingopyxis alaskensis RB2256
000803645.1	Sphingopyxis fribergensis Kp5.2
001314325.1	Sphingopyxis macrogoltabida 203
001307295.1	Sphingopyxis macrogoltabida EY-1
001278035.1	Sphingopyxis sp. 113P3
000319575.2	Spiribacter salinus M19-40
000485905.1	Spiribacter sp. UAH-SP71
000242595.3	Spirochaeta africana DSM 8902
000143985.1	Spirochaeta smaragdinae DSM 11293
000147075.1	Spirochaeta thermophila DSM 6192
000184345.2	Spirochaeta thermophila DSM 6578
000500935.1	Spiroplasma apis B31
001029245.1	Spiroplasma atrichopogonis GNAT3597
001281045.1	Spiroplasma cantharicola CC-1
000400935.1	Spiroplasma chrysopicola DF-1
000565175.1	Spiroplasma culicicola AES-1
000439455.1	Spiroplasma diminutum CUAS-1
001029265.1	Spiroplasma eriocheiris DSM 21848
001274875.1	Spiroplasma kunkelii CR2-3x
001267155.1	Spiroplasma litorale TN-1
000517365.1	Spiroplasma mirum ATCC 29335
000565215.1	Spiroplasma sabaudiense Ar-1343
000400955.1	Spiroplasma syrphidicola EA-1
000439435.1	Spiroplasma taiwanense CT-1
001262715.1	Spiroplasma turonicum Tab4c
000024525.1	Spirosoma linguale DSM 74
000974425.1	Spirosoma radiotolerans DG5A
001010805.1	Spongiibacter sp. IMCC21906

000024545.1	Stackebrandtia nassauensis DSM 44728
000317575.1	Stanieria cyanosphaera PCC 7437
001442815.1	Staphylococcus agnetis 908
000236925.1	Staphylococcus argenteus MSHR1132
001278745.1	Staphylococcus aureus
000025145.2	Staphylococcus aureus 04-02981
000296595.1	Staphylococcus aureus 08BA02176
000746505.1	Staphylococcus aureus 2395 USA500
000597965.1	Staphylococcus aureus 502A
000815045.1	Staphylococcus aureus ATCC BAA1680
000418345.1	Staphylococcus aureus Bmb9393
001045795.2	Staphylococcus aureus CA12
001021895.1	Staphylococcus aureus CA15
000412775.1	Staphylococcus aureus CA-347
001045995.2	Staphylococcus aureus HUV05
000953255.1	Staphylococcus aureus ILRI_Eymole1/1
001021875.1	Staphylococcus aureus M121
001457495.1	Staphylococcus aureus NCTC13435
001457515.1	Staphylococcus aureus NCTC8532
000626615.1	Staphylococcus aureus NRS100
000009005.1	Staphylococcus aureus RF122
001465635.1	Staphylococcus aureus RIVM1295
001465675.1	Staphylococcus aureus RIVM1607
001465755.1	Staphylococcus aureus RIVM3897
001027045.1	Staphylococcus aureus RKI4
001281145.1	Staphylococcus aureus SA564
000695875.1	Staphylococcus aureus UA-S391_USA300
000568455.1	Staphylococcus aureus USA300-ISMMS1
001046095.2	Staphylococcus aureus V2200
000709475.1	Staphylococcus aureus XN108
001444345.1	Staphylococcus aureus XQ
000695215.1	Staphylococcus aureus subsp. aureus

000239235.1	Staphylococcus aureus subsp. aureus 11819-97
000160335.2	Staphylococcus aureus subsp. aureus 55/2053
000462955.1	Staphylococcus aureus subsp. aureus 6850
000463055.1	Staphylococcus aureus subsp. aureus CN1
000012045.1	Staphylococcus aureus subsp. aureus COL
001027105.1	Staphylococcus aureus subsp. aureus DSM 20231
000253135.1	Staphylococcus aureus subsp. aureus ECT-R 2
000210315.1	Staphylococcus aureus subsp. aureus ED133
000024585.1	Staphylococcus aureus subsp. aureus ED98
000284535.1	Staphylococcus aureus subsp. aureus HO 5096 0412
000017125.1	Staphylococcus aureus subsp. aureus JH1
000016805.1	Staphylococcus aureus subsp. aureus JH9
000144955.1	Staphylococcus aureus subsp. aureus JKD6159
000237265.1	Staphylococcus aureus subsp. aureus LGA251
000237125.1	Staphylococcus aureus subsp. aureus M013
000011525.1	Staphylococcus aureus subsp. aureus MRSA252
000011505.1	Staphylococcus aureus subsp. aureus MSSA476
000011265.1	Staphylococcus aureus subsp. aureus MW2
000010445.1	Staphylococcus aureus subsp. aureus Mu3
000009665.1	Staphylococcus aureus subsp. aureus Mu50
000009645.1	Staphylococcus aureus subsp. aureus N315
000013425.1	Staphylococcus aureus subsp. aureus NCTC 8325
000737615.1	Staphylococcus aureus subsp. aureus SA268
000470865.1	Staphylococcus aureus subsp. aureus SA40
000470845.1	Staphylococcus aureus subsp. aureus SA957
000382965.1	Staphylococcus aureus subsp. aureus ST228
000009585.1	Staphylococcus aureus subsp. aureus ST398
000828035.1	Staphylococcus aureus subsp. aureus ST772-MRSA-V
000204665.1	Staphylococcus aureus subsp. aureus T0131
000159535.2	Staphylococcus aureus subsp. aureus TCH60
000027045.1	Staphylococcus aureus subsp. aureus TW20
000013465.1	Staphylococcus aureus subsp. aureus USA300_FPR3757

000017085.1	Staphylococcus aureus subsp. aureus USA300_TCH1516
000245495.1	Staphylococcus aureus subsp. aureus VC40
000485885.1	Staphylococcus aureus subsp. aureus Z172
000145595.1	Staphylococcus aureus subsp. aureus str. JKD6008
000010465.1	Staphylococcus aureus subsp. aureus str. Newman
001028645.1	Staphylococcus capitis AYP1020
000009405.1	Staphylococcus carnosus subsp. carnosus TM300
000007645.1	Staphylococcus epidermidis ATCC 12228
000751035.1	Staphylococcus epidermidis PM221
000011925.1	Staphylococcus epidermidis RP62A
000759555.1	Staphylococcus epidermidis SEI
001432245.1	Staphylococcus equorum KS1039
000009865.1	Staphylococcus haemolyticus JCSC1435
000972725.1	Staphylococcus haemolyticus Sh29/312/L2
000816085.1	Staphylococcus hyicus ATCC 11249
000025085.1	Staphylococcus lugdunensis HKU09-01
000270465.1	Staphylococcus lugdunensis N920143
000494875.1	Staphylococcus pasteuri SP1
000478385.1	Staphylococcus pseudintermedius E140
000189495.1	Staphylococcus pseudintermedius ED99
000185885.1	Staphylococcus pseudintermedius HKU10-03
000010125.1	Staphylococcus saprophyticus subsp. saprophyticus ATCC 15305
001188855.1	Staphylococcus schleiferi
001188895.1	Staphylococcus schleiferi 2142-05
001188915.1	Staphylococcus schleiferi 2317-03
001188875.1	Staphylococcus schleiferi 5909-02
000332735.1	Staphylococcus warneri SG1
000953575.1	Staphylococcus xylosus C2a
000706685.1	Staphylococcus xylosus HKUOPL8
000709415.1	Staphylococcus xylosus SMQ-121
000092465.1	Staphylothermus hellenicus DSM 12710
000015945.1	Staphylothermus marinus F1

000092925.1	Starkeya novella DSM 506
001314305.1	Stenotrophomonas acidaminiphila ZAC14D2_NAIMI4_2
000284595.1	Stenotrophomonas maltophilia D457
001274655.1	Stenotrophomonas maltophilia ISMMS2
001274675.1	Stenotrophomonas maltophilia ISMMS2R
001274595.1	Stenotrophomonas maltophilia ISMMS3
000223885.1	Stenotrophomonas maltophilia JV3
000072485.1	Stenotrophomonas maltophilia K279a
000020665.1	Stenotrophomonas maltophilia R551-3
000165485.1	Stigmatella aurantiaca DW4/3-1
000397185.1	Strawberry lethal yellows phytoplasma (CPA) str. NZSb11
000024565.1	Streptobacillus moniliformis DSM 12112
000831105.1	Streptococcus agalactiae
000427035.1	Streptococcus agalactiae 09mas018883
000599965.1	Streptococcus agalactiae 138P
000636115.1	Streptococcus agalactiae 138spar
000007265.1	Streptococcus agalactiae 2603V/R
000012705.1	Streptococcus agalactiae A909
000782855.1	Streptococcus agalactiae CNCTC 10/84
000689235.1	Streptococcus agalactiae COH1
000967445.1	Streptococcus agalactiae Feb-22
000831145.1	Streptococcus agalactiae GBS1-NY
001266635.1	Streptococcus agalactiae GBS85147
001448985.1	Streptococcus agalactiae GBS ST-1
000299135.1	Streptococcus agalactiae GD201008-001
001190865.1	Streptococcus agalactiae GX026
001190885.1	Streptococcus agalactiae H002
001190805.1	Streptococcus agalactiae HN016
000427075.1	Streptococcus agalactiae ILRI005
000427055.1	Streptococcus agalactiae ILRI112
000730215.2	Streptococcus agalactiae NGBS061
000730255.1	Streptococcus agalactiae NGBS572

000302475.2	Streptococcus agalactiae SA20-06
001275545.2	Streptococcus agalactiae SG-M1
001026925.1	Streptococcus agalactiae SS1
000831165.1	Streptococcus anginosus
000463465.1	Streptococcus anginosus C1051
000463505.1	Streptococcus anginosus C238
001412635.1	Streptococcus anginosus J4211
000478925.1	Streptococcus anginosus subsp. whileyi MAS624
000463425.1	Streptococcus constellatus subsp. pharyngis C1050
000463395.1	Streptococcus constellatus subsp. pharyngis C232
000463445.1	Streptococcus constellatus subsp. pharyngis C818
000493775.1	Streptococcus dysgalactiae subsp. equisimilis 167
000317855.1	Streptococcus dysgalactiae subsp. equisimilis AC-2713
000188715.1	Streptococcus dysgalactiae subsp. equisimilis ATCC 12394
000010705.1	Streptococcus dysgalactiae subsp. equisimilis GGS_124
000307185.1	Streptococcus dysgalactiae subsp. equisimilis RE378
000026585.1	Streptococcus equi subsp. equi 4047
000026605.1	Streptococcus equi subsp. zooepidemicus
000219765.1	Streptococcus equi subsp. zooepidemicus ATCC 35246
000696505.1	Streptococcus equi subsp. zooepidemicus CY
000020765.1	Streptococcus equi subsp. zooepidemicus MGCS10565
001477575.1	Streptococcus gallolyticus ICDDRB-NRC-S1
000027185.1	Streptococcus gallolyticus UCN34
000270145.1	Streptococcus gallolyticus subsp. gallolyticus ATCC 43143
000203195.1	Streptococcus gallolyticus subsp. gallolyticus ATCC BAA-2069
001281105.1	Streptococcus gordonii KCOM 1506 (= ChDC B679)
000017005.1	Streptococcus gordonii str. Challis substr. CH1
001477615.1	Streptococcus infantarius ICDDRB-NRC-S5
000246835.1	Streptococcus infantarius subsp. infantarius CJ18
000648525.1	Streptococcus iniae ISET0901
000648555.1	Streptococcus iniae ISNO
000403625.1	Streptococcus iniae SF1

000831485.1	Streptococcus iniae YSFST01-82
000463355.1	Streptococcus intermedius B196
000463385.1	Streptococcus intermedius C270
000306805.1	Streptococcus intermedius JTH08
001296205.1	Streptococcus intermedius KCOM 1545
000441535.1	Streptococcus lutetiensis 033
000283635.1	Streptococcus macedonicus ACA-DC 198
000027165.1	Streptococcus mitis B6
001281025.1	Streptococcus mitis KCOM 1350 (= ChDC B183)
000271865.1	Streptococcus mutans GS-5
000284575.1	Streptococcus mutans LJ23
000091645.1	Streptococcus mutans NN2025
000007465.2	Streptococcus mutans UA159
000817065.1	Streptococcus mutans UA159-FR
000385925.1	Streptococcus oligofermentans AS 1.3089
000253155.1	Streptococcus oralis Uo5
000164675.2	Streptococcus parasanguinis ATCC 15912
000262145.1	Streptococcus parasanguinis FW213
000213825.1	Streptococcus parauberis KCTC 11537
000187935.2	Streptococcus parauberis NCFD 2020
000270165.1	Streptococcus pasteurianus ATCC 43144
000147095.1	Streptococcus pneumoniae 670-6B
000018965.1	Streptococcus pneumoniae 70585
000146975.1	Streptococcus pneumoniae AP200
000026665.1	Streptococcus pneumoniae ATCC 700669
000019985.1	Streptococcus pneumoniae CGSP14
000014365.1	Streptococcus pneumoniae D39
000019825.1	Streptococcus pneumoniae G54
000019265.1	Streptococcus pneumoniae Hungary19A-6
000210975.1	Streptococcus pneumoniae INV104
000210935.1	Streptococcus pneumoniae INV200
000018985.1	Streptococcus pneumoniae JJA

001457635.1	Streptococcus pneumoniae NCTC7465
000817005.1	Streptococcus pneumoniae NT_110_58
000210955.1	Streptococcus pneumoniae OXC141
000019005.1	Streptococcus pneumoniae P1031
000348705.1	Streptococcus pneumoniae PCS8235
000007045.1	Streptococcus pneumoniae R6
000211075.1	Streptococcus pneumoniae SPN032672
000211095.1	Streptococcus pneumoniae SPN033038
000210995.1	Streptococcus pneumoniae SPN034156
000211015.1	Streptococcus pneumoniae SPN034183
000211035.2	Streptococcus pneumoniae SPN994038
000211055.2	Streptococcus pneumoniae SPN994039
000180515.2	Streptococcus pneumoniae SPNA45
000251085.2	Streptococcus pneumoniae ST556
000196595.1	Streptococcus pneumoniae TCH8431/19A
000006885.1	Streptococcus pneumoniae TIGR4
000019025.1	Streptococcus pneumoniae Taiwan19F-14
000299015.1	Streptococcus pneumoniae gamPNI0373
000221985.1	Streptococcus pseudopneumoniae IS7493
001039695.2	Streptococcus pyogenes
000772245.1	Streptococcus pyogenes 1E1
001021955.1	Streptococcus pyogenes 5448
000767505.1	Streptococcus pyogenes 7F7
000307535.1	Streptococcus pyogenes A20
000993765.1	Streptococcus pyogenes AP1
000743015.1	Streptococcus pyogenes ATCC 19615
000230295.1	Streptococcus pyogenes Alab49
001014305.1	Streptococcus pyogenes D471
000772185.1	Streptococcus pyogenes HKU360
001051095.1	Streptococcus pyogenes HKU488
000275625.1	Streptococcus pyogenes HKU QMH11M0907901
000422045.1	Streptococcus pyogenes HSC5

001014285.1	Streptococcus pyogenes JRS4
000349925.2	Streptococcus pyogenes M1 476
000006785.2	Streptococcus pyogenes M1 GAS
000756485.1	Streptococcus pyogenes M23ND
001020185.1	Streptococcus pyogenes M28PF1
000013505.1	Streptococcus pyogenes MGAS10270
000011665.1	Streptococcus pyogenes MGAS10394
000013545.1	Streptococcus pyogenes MGAS10750
000250905.1	Streptococcus pyogenes MGAS15252
000250925.1	Streptococcus pyogenes MGAS1882
000013525.1	Streptococcus pyogenes MGAS2096
000007425.1	Streptococcus pyogenes MGAS315
000011765.2	Streptococcus pyogenes MGAS5005
000012165.1	Streptococcus pyogenes MGAS6180
000007285.1	Streptococcus pyogenes MGAS8232
000013485.1	Streptococcus pyogenes MGAS9429
001267805.1	Streptococcus pyogenes NGAS322
001019695.1	Streptococcus pyogenes NGAS327
001019675.1	Streptococcus pyogenes NGAS596
001267845.1	Streptococcus pyogenes NGAS638
001019635.1	Streptococcus pyogenes NGAS743
000018125.1	Streptococcus pyogenes NZ131
000011285.1	Streptococcus pyogenes SSI-1
001023495.1	Streptococcus pyogenes STAB10015
000732385.1	Streptococcus pyogenes STAB901
000732425.1	Streptococcus pyogenes STAB902
000009385.1	Streptococcus pyogenes str. Manfredo
000305335.1	Streptococcus salivarius 57.I
000253335.1	Streptococcus salivarius CCHSS3
000253315.1	Streptococcus salivarius JIM8777
000785515.1	Streptococcus salivarius NCTC 8618
000014205.1	Streptococcus sanguinis SK36

000479335.1	Streptococcus sp. I-G2
000479315.1	Streptococcus sp. I-P16
000688775.1	Streptococcus sp. VT 162
000168355.3	Streptococcus suis 05HAS68
000014305.1	Streptococcus suis 05ZYH33
000732355.1	Streptococcus suis 6407
000014325.1	Streptococcus suis 98HAH33
000233575.1	Streptococcus suis A7
000026745.1	Streptococcus suis BM407
000231905.1	Streptococcus suis D12
000231885.1	Streptococcus suis D9
000018185.1	Streptococcus suis GZ1
000186405.1	Streptococcus suis JS14
001272635.1	Streptococcus suis NSUI002
000091905.1	Streptococcus suis P1/7
000294495.1	Streptococcus suis S735
000344765.1	Streptococcus suis SC070731
000026725.1	Streptococcus suis SC84
000231865.1	Streptococcus suis SS12
000231925.1	Streptococcus suis ST1
000204625.1	Streptococcus suis ST3
000494895.1	Streptococcus suis T15
000390245.1	Streptococcus suis TL13
000471985.1	Streptococcus suis YB51
000993745.1	Streptococcus suis ZY05719
000698885.1	Streptococcus thermophilus ASCC 1275
000011845.1	Streptococcus thermophilus CNRZ1066
000253395.1	Streptococcus thermophilus JIM 8232
000014485.1	Streptococcus thermophilus LMD-9
000011825.1	Streptococcus thermophilus LMG 18311
001280285.1	Streptococcus thermophilus MN-BM-A01
001008015.1	Streptococcus thermophilus MN-BM-A02

000262675.1	Streptococcus thermophilus MN-ZLW-002
000182875.1	Streptococcus thermophilus ND03
000971665.1	Streptococcus thermophilus SMQ-301
000009545.1	Streptococcus uberis 0140J
000695235.1	Streptomyces albulus NK660
000963515.1	Streptomyces albulus ZPM
000827005.1	Streptomyces albus DSM 41398
000359525.1	Streptomyces albus J1074
001267885.1	Streptomyces ambofaciens ATCC 23877
000009765.1	Streptomyces avermitilis MA-4680 = NBRC 14893
000092385.1	Streptomyces bingchenggensis BCW-1
000237305.1	Streptomyces cattleya NRRL 8057 = DSM 46488
000444875.1	Streptomyces collinus Tu 365
000931445.1	Streptomyces cyaneogriseus subsp. noncyanogenus
000385945.1	Streptomyces fulvissimus DSM 40593
000761215.1	Streptomyces glaucescens GLA.O
000261345.2	Streptomyces globisporus C-1027
000010605.1	Streptomyces griseus subsp. griseus NBRC 13350
000245355.1	Streptomyces hygroscopicus subsp. jinggangensis 5008
000340845.1	Streptomyces hygroscopicus subsp. jinggangensis TL01
001447075.1	Streptomyces hygroscopicus subsp. limoneus
001013905.1	Streptomyces leeuwenhoekii type strain (C34 = DSM 42122 = NRRL B-24963)
000739105.1	Streptomyces lividans TK24
000952035.1	Streptomyces lydicus A02
000176115.2	Streptomyces pratensis ATCC 33331
001278075.1	Streptomyces pristinaespiralis HCCB 10218
000091305.1	Streptomyces scabiei 87.22
001484705.1	Streptomyces sp. 4F
000816025.1	Streptomyces sp. 769
001278095.1	Streptomyces sp. CFMR 7
001011035.1	Streptomyces sp. CNQ-509
001484565.1	Streptomyces sp. CdTB01

000412265.2	Streptomyces sp. Mg1
000364805.1	Streptomyces sp. PAMC26508
000177195.2	Streptomyces sp. SirexAA-E
001406115.1	Streptomyces venezuelae ATCC 15439
000830005.1	Streptomyces vietnamensis GIMV4.0001
000147815.3	Streptomyces violaceusniger Tu 4113
000993785.2	Streptomyces xiamenensis MCCC 1A01550
000024865.1	Streptosporangium roseum DSM 43021
000237975.1	Sulfobacillus acidophilus DSM 10332
000219855.1	Sulfobacillus acidophilus TPY
000012285.1	Sulfolobus acidocaldarius DSM 639
000340315.1	Sulfolobus acidocaldarius N8
000338775.1	Sulfolobus acidocaldarius Ron12/I
000508305.1	Sulfolobus acidocaldarius SUSAZ
000189575.1	Sulfolobus islandicus HVE10/4
000364745.1	Sulfolobus islandicus LAL14/1
000022385.1	Sulfolobus islandicus L.S.2.15
000022405.1	Sulfolobus islandicus M.14.25
000022425.1	Sulfolobus islandicus M.16.27
000022445.1	Sulfolobus islandicus M.16.4
000189555.1	Sulfolobus islandicus REY15A
000022465.1	Sulfolobus islandicus Y.G.57.14
000022485.1	Sulfolobus islandicus Y.N.15.51
000024745.1	Sulfolobus solfataricus 98/2
000968395.1	Sulfolobus solfataricus 98/2 SULC
000007005.1	Sulfolobus solfataricus P2
000968435.1	Sulfolobus solfataricus SULA
000968355.1	Sulfolobus solfataricus SULB
000011205.1	Sulfolobus tokodaii str. 7
000297055.2	Sulfuricella denitrificans skB26
000183725.1	Sulfuricurvum kujiense DSM 16994
000021545.1	Sulfurihydrogenibium azorense Az-Fu1

000020325.1	Sulfurihydrogenibium sp. YO3AOP1
000147355.1	Sulfurimonas autotrophica DSM 16294
000012965.1	Sulfurimonas denitrificans DSM 1251
000242915.2	Sulfurimonas gotlandica GD1
000828635.1	Sulfuritalea hydrogenivorans sk43H
000265295.1	Sulfurospirillum barnesii SES-3
000024885.1	Sulfurospirillum deleyianum DSM 6946
000568815.1	Sulfurospirillum multivorans DSM 12446
000987835.1	Sulfurovum lithotrophicum ATCC BAA-797
000010345.1	Sulfurovum sp. NBC37-1
000009905.1	Symbiobacterium thermophilum IAM 14863
000010065.1	Synechococcus elongatus PCC 6301
000012525.1	Synechococcus elongatus PCC 7942
000014585.1	Synechococcus sp. CC9311
000012625.1	Synechococcus sp. CC9605
000012505.1	Synechococcus sp. CC9902
000013225.1	Synechococcus sp. JA-2-3B'a(2-13)
000013205.1	Synechococcus sp. JA-3-3Ab
000737535.1	Synechococcus sp. KORDI-100
000737575.1	Synechococcus sp. KORDI-49
000737595.1	Synechococcus sp. KORDI-52
000316685.1	Synechococcus sp. PCC 6312
000019485.1	Synechococcus sp. PCC 7002
000317085.1	Synechococcus sp. PCC 7502
000063525.1	Synechococcus sp. RCC307
000817325.1	Synechococcus sp. UTEX 2973
000063505.1	Synechococcus sp. WH 7803
001182765.1	Synechococcus sp. WH 8103
000161795.2	Synechococcus sp. WH 8109
000478825.2	Synechocystis sp. PCC 6714
000009725.1	Synechocystis sp. PCC 6803
000284135.1	Synechocystis sp. PCC 6803 substr. GT-I

000284215.1	Synechocystis sp. PCC 6803 substr. PCC-N
000284455.1	Synechocystis sp. PCC 6803 substr. PCC-P
000014965.1	Syntrophobacter fumaroxidans MPOB
000190635.1	Syntrophobotulus glycolicus DSM 8271
000014725.1	Syntrophomonas wolfei subsp. wolfei str. Goettingen G311
000092405.1	Syntrophothermus lipocalidus DSM 12680
000013405.1	Syntrophus aciditrophicus SB
000238215.1	Tannerella forsythia 92A2
000953635.1	Tatlockia micdadei ATCC33218
000226625.1	Taylorella asinigenitalis MCE3
000276685.1	Taylorella equigenitalis ATCC 35865
000185745.1	Taylorella equigenitalis MCE9
001483385.1	Tenacibaculum dicentrarchi AY7486TD
000213235.1	Tepidanaerobacter acetatoxydans Re1
000023025.1	Teredinibacter turnerae T7901
000725365.1	Terribacillus aidingensis MP602
000265425.1	Terriglobus roseus DSM 18391
000179915.2	Terriglobus saanensis SP1PR4
000283615.1	Tetragenococcus halophilus NBRC 12172
000355675.1	Thalassolituus oleivorans MIL-1
000582555.1	Thalassolituus oleivorans R6-15
000300235.2	Thalassospira xiamenensis M-5 = DSM 17429
000021765.1	Thauera sp. MZ1T
000802205.2	Thaumarchaeota archaeon MY3
000305935.1	Thermacetogenium phaeum DSM 12270
000184705.1	Thermaerobacter marianensis DSM 12885
000024905.1	Thermanaerovibrio acidaminovorans DSM 6589
000092945.1	Thermincola potens JR
000175295.2	Thermoanaerobacter brockii subsp. finnii Ako-1
000025645.1	Thermoanaerobacter italicus Ab9
000763575.1	Thermoanaerobacter kivui LKT-1
000092965.1	Thermoanaerobacter mathranii subsp. mathranii str. A3

000019085.1	Thermoanaerobacter pseudethanolicus ATCC 33223
000148425.1	Thermoanaerobacter sp. X513
000019065.1	Thermoanaerobacter sp. X514
000147695.3	Thermoanaerobacter wiegelii Rt8.B1
000307585.1	Thermoanaerobacterium saccharolyticum JW/SL-YS485
000145615.1	Thermoanaerobacterium thermosaccharolyticum DSM 571
000328545.1	Thermoanaerobacterium thermosaccharolyticum M0795
000189775.3	Thermoanaerobacterium xylanolyticum LX-11
000227705.3	Thermobacillus composti KWC4
000025005.1	Thermobaculum terrenum ATCC BAA-798
000012405.1	Thermobifida fusca YX
000092645.1	Thermobispora bispora DSM 43833
001433455.1	Thermococcus barophilus CH5
000151105.2	Thermococcus barophilus MP
000265525.1	Thermococcus cleftensis CL1
000769655.1	Thermococcus eurythermalis A501
000022365.1	Thermococcus gammatolerans EJ3
000009965.1	Thermococcus kodakarensis KOD1
000246985.3	Thermococcus litoralis DSM 5473
000585495.1	Thermococcus nautili 30-1
000018365.1	Thermococcus onnurineus NA1
000517445.1	Thermococcus paralvinellae ES1
000022545.1	Thermococcus sibiricus MM 739
001484685.1	Thermococcus sp. 2319x1
000221185.1	Thermococcus sp. 4557
000151205.2	Thermococcus sp. AM4
000025605.1	Thermocrinis albus DSM 14484
000512735.1	Thermocrinis ruber DSM 23557
000217795.1	Thermodesulfatator indicus DSM 15286
000734015.1	Thermodesulfobacterium commune DSM 2178
000215975.1	Thermodesulfobacterium geofontis OPF15
000212395.1	Thermodesulfobium narugense DSM 14796

000020985.1	Thermodesulfovibrio yellowstonii DSM 11347
000813245.1	Thermofilum carboxyditrophus 1505
000015225.1	Thermofilum pendens Hrk 5
000993805.1	Thermofilum sp. 1807-2
000446015.1	Thermofilum sp. 1910b
000264495.1	Thermogladius cellulolyticus 1633
000021685.1	Thermomicrobium roseum DSM 5159
000024385.1	Thermomonospora curvata DSM 43183
000011185.1	Thermoplasma volcanium GSS1
000350305.1	Thermoplasmatales archaeon BRNA1
000253055.1	Thermoproteus tenax Kra 1
000193375.1	Thermoproteus uzoniensis 768-20
000144645.1	Thermosediminibacter oceani DSM 16646
000021285.1	Thermosipho africanus TCF52B
000016905.1	Thermosipho melanesiensis BI429
000092185.1	Thermosphaera aggregans DSM 11486
000011345.1	Thermosynechococcus elongatus BP-1
000828655.1	Thermotoga caldifontis AZM44c09
000008545.1	Thermotoga maritima MSB8
000978575.1	Thermotoga maritima Tma100
000978535.1	Thermotoga maritima Tma200
000025105.1	Thermotoga naphthophila RKU-10
000018945.1	Thermotoga neapolitana DSM 4359
000016785.1	Thermotoga petrophila RKU-1
000828675.1	Thermotoga profunda AZM34c06
000789335.1	Thermotoga sp. 2812B
000789375.1	Thermotoga sp. Cell2
000019625.1	Thermotoga sp. RQ2
000832145.1	Thermotoga sp. RQ7
000185805.1	Thermovibrio ammonificans HB-1
000233775.1	Thermovirga lienii DSM 17291
001399775.1	Thermus aquaticus Y51MC23

000309885.1	Thermus oshimai JL-2
000187005.1	Thermus scotoductus SA-01
000236585.1	Thermus sp. CCB_US3_UF1
000008125.1	Thermus thermophilus HB27
000091545.1	Thermus thermophilus HB8
000258245.1	Thermus thermophilus JL-18
000214845.1	Thermus thermophilus SG0.5JP17-16
000227665.3	Thioalkalimicrobium aerophilum AL3
000214825.1	Thioalkalimicrobium cyclicum ALM1
000321415.2	Thioalkalivibrio nitratireducens DSM 14787
000227685.3	Thioalkalivibrio paradoxus ARh 1
000025545.1	Thioalkalivibrio sp. K90mix
000021985.1	Thioalkalivibrio sulfidiphilus HL-EbGr7
001020955.1	Thioalkalivibrio versutus D301
000012745.1	Thiobacillus denitrificans ATCC 25259
000227745.3	Thiocystis violascens DSM 198
000327045.1	Thioflavicoccus mobilis 8321
000828615.1	Thiolapillus brandeum
000012605.1	Thiomicrospira crunogena XCL-2
000092605.1	Thiomonas intermedia K12
000828835.1	Thioploca ingrica
000264455.2	Tistrella mobilis KA081020-065
000023065.1	Tolumonas auensis DSM 9187
000214355.1	Treponema azotonutricium ZAS-9
000212415.1	Treponema brennaborense DSM 12168
000219725.1	Treponema caldarium DSM 7334
000008185.1	Treponema denticola ATCC 35405
000008605.1	Treponema pallidum subsp. pallidum str. Nichols
000604125.1	Treponema pallidum subsp. pallidum str. Sea 81-4
000217655.1	Treponema paraluiscuniculi Cuniculi A
000447675.1	Treponema pedis str. T A4
000214375.1	Treponema primitia ZAS-2

000755145.1	Treponema putidum OMZ 758 (ATCC 700334)
000775995.1	Treponema sp. OMZ 838
000195275.1	Treponema succinifaciens DSM 2489
000014265.1	Trichodesmium erythraeum IMS101
000007485.1	Tropheryma whipplei str. Twist
000092425.1	Truepera radiovictrix DSM 17093
001281085.1	Trueperella pyogenes 2012CQ-ZSH
000612055.1	Trueperella pyogenes TP6375
000816065.1	Trueperella pyogenes TP8
000092225.1	Tsukamurella paurometabola DSM 20162
000266885.1	Turneriella parva DSM 21527
000828735.1	Ureaplasma parvum serovar 3
000019345.1	Ureaplasma parvum serovar 3 str. ATCC 27815
000006625.1	Ureaplasma parvum serovar 3 str. ATCC 700970
000021265.1	Ureaplasma urealyticum serovar 10 str. ATCC 33699
000463015.1	Variovorax paradoxus B4
000184745.1	Variovorax paradoxus EPS
000023345.1	Variovorax paradoxus S110
000024945.1	Veillonella parvula DSM 2008
000015565.1	Verminephrobacter eiseniae EF01-2
000972765.1	Verrucomicrobia bacterium IMCC26134
001017655.1	Verrucomicrobia bacterium L21-Fru-AB
000172155.1	Verrucomicrobium spinosum DSM 4136 = JCM 18804
000204155.1	Verrucosispora maris AB-18-032
001469735.1	Vibrio alginolyticus ATCC 33787
000354175.2	Vibrio alginolyticus NBRC 15630 = ATCC 17749
000786425.1	Vibrio anguillarum
000217675.1	Vibrio anguillarum 775
000462975.1	Vibrio anguillarum M3
000024825.1	Vibrio antiquarius EX25
000017705.1	Vibrio campbellii ATCC BAA-1116
000765415.1	Vibrio cholerae

000969265.1	Vibrio cholerae 10432-62
000969235.1	Vibrio cholerae 1154-74
000275645.1	Vibrio cholerae H1
000250855.1	Vibrio cholerae IEC224
000195065.1	Vibrio cholerae LMA3984-4
000021605.1	Vibrio cholerae M66-2
000022585.1	Vibrio cholerae MJ-1236
000829215.1	Vibrio cholerae MS6
000963555.1	Vibrio cholerae O1 biovar El Tor
000006745.1	Vibrio cholerae O1 biovar El Tor str. N16961
000166455.2	Vibrio cholerae O1 str. 2010EL-1786
001318185.1	Vibrio cholerae O1 str. KW3
000016245.1	Vibrio cholerae O395
001045415.1	Vibrio cholerae TSY216
000763535.2	Vibrio coralliilyticus
000772065.1	Vibrio coralliilyticus RE98
000011805.1	Vibrio fischeri ES114
000184325.1	Vibrio furnissii NCTC 11218
000801275.1	Vibrio nigripulchritudo
000328405.1	Vibrio parahaemolyticus BB22OP
001433415.1	Vibrio parahaemolyticus FORC_004
001304775.1	Vibrio parahaemolyticus FORC_006
001244315.1	Vibrio parahaemolyticus FORC_008
000430425.1	Vibrio parahaemolyticus O1:K33 str. CDC_K4557
000430405.1	Vibrio parahaemolyticus O1:Kuk str. FDA_R31
000196095.1	Vibrio parahaemolyticus RIMD 2210633
000568495.1	Vibrio parahaemolyticus UCM-V493
000241385.1	Vibrio sp. EJY3
000091465.1	Vibrio tasmaniensis LGP32
000772105.1	Vibrio tubiashii ATCC 19109
000746665.1	Vibrio vulnificus 93U204
000039765.1	Vibrio vulnificus CMCP6

001433435.1	Vibrio vulnificus FORC_009
000186585.1	Vibrio vulnificus MO6-24/O
000009745.1	Vibrio vulnificus YJ016
000725285.1	Virgibacillus sp. SK37
000148385.1	Vulcanisaeta distributa DSM 14429
000190315.1	Vulcanisaeta moutnovskia 768-28
001263175.1	Vulgatibacter incomptus DSM 27710
000092785.1	Waddlia chondrophila WSU 86-1044
000189415.1	Weeksella virosa DSM 16922
000732905.1	Weissella ceti WS08
000750535.1	Weissella ceti WS105
000750515.1	Weissella ceti WS74
001308145.1	Weissella cibaria CH2
000219805.1	Weissella koreensis KACC 15510
001187785.1	Wenzhouxiangella marina KCTC 42284
000008885.1	Wigglesworthia glossinidia endosymbiont of Glossina brevipalpis
000247565.1	Wigglesworthia glossinidia endosymbiont of Glossina morsitans morsitans (Yale colony)
000828715.1	Winogradskyella sp. PG-2
000953315.1	Wolbachia
000829315.1	Wolbachia endosymbiont of Cimex lectularius
000008025.1	Wolbachia endosymbiont of Drosophila melanogaster
000376605.1	Wolbachia endosymbiont of Drosophila simulans wHa
000376585.1	Wolbachia endosymbiont of Drosophila simulans wNo
000306885.1	Wolbachia endosymbiont of Onchocerca ochengi
000530755.1	Wolbachia endosymbiont of Onchocerca volvulus str. Cameroon
000008385.1	Wolbachia endosymbiont strain TRS of Brugia malayi
000022285.1	Wolbachia sp. wRi
000017645.1	Xanthobacter autotrophicus Py2
000087965.1	Xanthomonas albilineans GPE PC73
000348585.1	Xanthomonas axonopodis Xac29-1
000007165.1	Xanthomonas axonopodis pv. citri str. 306
000225915.1	Xanthomonas axonopodis pv. citrumelo F1

000972745.1	Xanthomonas campestris 17
001186415.1	Xanthomonas campestris pv. campestris
000012105.1	Xanthomonas campestris pv. campestris str. 8004
000007145.1	Xanthomonas campestris pv. campestris str. ATCC 33913
000070605.1	Xanthomonas campestris pv. campestris str. B100
000221965.1	Xanthomonas campestris pv. raphani 756C
001028285.2	Xanthomonas citri pv. citri
000961155.1	Xanthomonas citri subsp. citri
000816885.1	Xanthomonas citri subsp. citri A306
000349225.1	Xanthomonas citri subsp. citri Aw12879
000961175.1	Xanthomonas citri subsp. citri UI6
000009165.1	Xanthomonas euvesicatoria 85-10
000969685.1	Xanthomonas fuscans subsp. fuscans str. 4834-R
001466505.1	Xanthomonas oryzae pv. oryzae
000007385.1	Xanthomonas oryzae pv. oryzae KACC 10331
000010025.1	Xanthomonas oryzae pv. oryzae MAFF 311018
000948075.1	Xanthomonas oryzae pv. oryzae PXO86
000019585.2	Xanthomonas oryzae pv. oryzae PXO99A
000940825.1	Xanthomonas oryzae pv. oryzicola
000168315.3	Xanthomonas oryzae pv. oryzicola BLS256
000815185.1	Xanthomonas sacchari R1
001021935.1	Xanthomonas translucens pv. undulosa
000973125.1	Xenorhabdus bovienii CS03
000027225.1	Xenorhabdus bovienii SS-2004
000968195.1	Xenorhabdus doucetiae FRM16
000953355.1	Xenorhabdus nematophila AN6/1
000968175.1	Xenorhabdus poinarii G6
000024965.1	Xylanimonas cellulosilytica DSM 15894
000006725.1	Xylella fastidiosa 9a5c
000019325.1	Xylella fastidiosa M12
000019765.1	Xylella fastidiosa M23
000698825.1	Xylella fastidiosa MUL0034

000007245.1	Xylella fastidiosa Temecula1
000148405.1	Xylella fastidiosa subsp. fastidiosa GB514
000698805.1	Xylella fastidiosa subsp. sandyi Ann-1
000834395.1	Yersinia aldovae 670-83
001047675.1	Yersinia aleksiciae 159
000834735.1	Yersinia enterocolitica 2516-87
000987925.1	Yersinia enterocolitica FORC_002
001305635.1	Yersinia enterocolitica KNG22703
000597945.1	Yersinia enterocolitica LC20
000834195.1	Yersinia enterocolitica WA
000009345.1	Yersinia enterocolitica subsp. enterocolitica 8081
000192105.1	Yersinia enterocolitica subsp. palearctica 105.5R(r)
000253175.1	Yersinia enterocolitica subsp. palearctica Y11
000968115.1	Yersinia enterocolitica (type O:5) str. YE53/03
000834215.1	Yersinia frederiksenii Y225
000834515.1	Yersinia intermedia Y228
000834865.1	Yersinia kristensenii Y231
001188695.1	Yersinia pestis 1412
001188935.1	Yersinia pestis 1413
001188715.1	Yersinia pestis 1522
001188815.1	Yersinia pestis 2944
001188795.1	Yersinia pestis 3067
001188775.1	Yersinia pestis 3770
001188755.1	Yersinia pestis 8787
000222975.1	Yersinia pestis A1122
000018805.1	Yersinia pestis Angola
000013825.1	Yersinia pestis Antiqua
000009065.1	Yersinia pestis CO92
000022805.1	Yersinia pestis D106004
000022825.1	Yersinia pestis D182038
000834775.1	Yersinia pestis Dodson
000834495.1	Yersinia pestis El Dorado

000834275.1	Yersinia pestis Harbin35
000006645.1	Yersinia pestis KIM10+
000013805.1	Yersinia pestis Nepal516
000834885.1	Yersinia pestis Nicholisk 41
000834235.1	Yersinia pestis PBM19
000016445.1	Yersinia pestis Pestoides F
000834985.1	Yersinia pestis Pestoides G
000834335.1	Yersinia pestis Shasta
000022845.1	Yersinia pestis Z176003
000186725.1	Yersinia pestis biovar Medievalis str. Harbin 35
000007885.1	Yersinia pestis biovar Microtus str. 91001
000834925.1	Yersinia pestis str. Pestoides B
000834435.1	Yersinia pseudotuberculosis 1
000750315.1	Yersinia pseudotuberculosis ATCC 6904
000834415.1	Yersinia pseudotuberculosis EP2/+
000016945.1	Yersinia pseudotuberculosis IP 31758
000047365.1	Yersinia pseudotuberculosis IP 32953
000834355.1	Yersinia pseudotuberculosis MD67
000020085.1	Yersinia pseudotuberculosis PB1/+
000019465.1	Yersinia pseudotuberculosis YPIII
000834945.1	Yersinia pseudotuberculosis str. PA3606
000834455.1	Yersinia rohdei YRA
000964565.1	Yersinia ruckeri Big Creek 74
000834255.1	Yersinia ruckeri YRB
000582515.1	Yersinia similis Y_sim_228
000973105.1	Zobellia galactanivorans DsiJT
000023465.1	Zunongwangia profunda SM-A87
000175255.2	Zymomonas mobilis subsp. mobilis ATCC 10988
000277755.1	Zymomonas mobilis subsp. mobilis ATCC 29191
000024245.1	Zymomonas mobilis subsp. mobilis NCIMB 11163
000576125.1	Zymomonas mobilis subsp. mobilis NRRL B-12526
000007105.1	Zymomonas mobilis subsp. mobilis ZM4 = ATCC 31821

000498655.1	Zymomonas mobilis subsp. mobilis str. CP4 = NRRL B-14023
000218875.1	Zymomonas mobilis subsp. pomaceae ATCC 29192
000299095.1	alpha proteobacterium HIMB5
000299115.1	alpha proteobacterium HIMB59
000830275.1	archaeon GW2011_AR10
000830315.1	archaeon GW2011_AR20
001443605.1	bacterium L21-Spi-D4
000342265.1	beta proteobacterium CB
001029795.1	candidate division Kazan bacterium GW2011_GWA1_50_15
001007975.1	candidate division SR1 bacterium Aalborg_AAW-1
000829235.1	cyanobacterium endosymbiont of Epithemia turgida isolate EtSB Lake Yunoko
000730245.1	endosymbiont of Acanthamoeba sp. UWC8
000709555.1	endosymbiont of Llaveia axin axin
000801295.1	endosymbiont of unidentified scaly snail isolate Monju
000198515.1	gamma proteobacterium HdN1
000224475.1	halophilic archaeon DL31
000287335.1	secondary endosymbiont of Ctenarytaina eucalypti
000287355.1	secondary endosymbiont of Heteropsylla cubana
000503875.1	sediment metagenome
000831405.1	symbiont metagenome
000474035.1	synthetic Escherichia coli C321.deltaA
000146025.1	uncultured Termite group 1 bacterium phylotype Rs-D17

Supplementary Table 5. Table containing identified AAT-fold decarboxylase sequences.

Taxonomy ID	Assembly number	Species	Accession number
1000561	000220025.3	Paeruginosa	ALT72174.1
1000561	000220025.3	Paeruginosa	ALT72540.1
1001534	000248435.1	Ftularensis	WP_003020112.1
1001542	000248415.1	Ftularensis	WP_003020112.1
1001582	000204275.1	Bamyloliquefa	WP_013350743.1
1001582	000204275.1	Bamyloliquefa	WP_014470448.1
1001585	000204295.1	Pmendocina	WP_013715345.1
1001714	000497265.2	Mabscessus	WP_005137308.1
1001989	000219515.3	Ecoli	AKK46781.1
1001989	000219515.3	Ecoli	AKK49751.1
1001989	000219515.3	Ecoli	AKK51043.1
1001989	000219515.3	Ecoli	AKK51069.1
1002809	000271325.1	Ssilvestris	WP_008403458.1
1002809	000271325.1	Ssilvestris	WP_041865812.1
1003191	000486405.2	Senterica	WP_000839724.1
1003191	000486405.2	Senterica	WP_000978689.1
1003191	000486405.2	Senterica	WP_000992186.1
1003191	000486405.2	Senterica	WP_001021043.1
1003191	000486405.2	Senterica	WP_001100652.1
1003191	000486405.2	Senterica	WP_001292402.1
1003195	000237305.1	Scattleya	WP_014140810.1
1004785	000299995.1	Amacleodii	WP_014999137.1
1004787	000300175.1	Amacleodii	WP_014979908.1
1004788	000299955.1	Amacleodii	WP_014977117.1
1004952	000231905.1	Ssuis	WP_014638289.1
1005048	000221045.1	Cfungivorans	WP_041741452.1
1006007	000225265.1	Bmegaterium	WP_014462072.1
1006007	000225265.1	Bmegaterium	WP_028414107.1
1006543	000204665.1	Saureus	WP_000812832.1
1006551	000240325.1	Koxytoca	WP_004848492.1
1006551	000240325.1	Koxytoca	WP_014226846.1
1006551	000240325.1	Koxytoca	WP_014228008.1
1006598	000478545.1	Splymuthica	WP_006327522.1
1006598	000478545.1	Splymuthica	WP_037433640.1
1007105	000209655.1	Psp	WP_013743231.1
1007676	001050475.1	Lginsenosidim	WP_048706237.1
1008297	000252875.1	Senterica	WP_000839739.1
1008297	000252875.1	Senterica	WP_000978690.1
1008297	000252875.1	Senterica	WP_001100652.1
1008297	000252875.1	Senterica	WP_001292402.1

1008459	000226625.1	Tasinigenital	WP_014112054.1
1009846	000292915.1	Bcepacia	WP_014896349.1
1009846	000292915.1	Bcepacia	WP_014898692.1
1010834	000572125.1	Mtuberculosis	WP_003899363.1
1010835	000572155.1	Mtuberculosis	X1
1010836	000572175.1	Mtuberculosis	WP_003899363.1
101510	000014565.1	Rjostii	WP_007297490.1
1016998	000018705.1	Senterica	WP_000839733.1
1016998	000018705.1	Senterica	WP_000978691.1
1016998	000018705.1	Senterica	WP_001021052.1
1016998	000018705.1	Senterica	WP_001100652.1
1016998	000018705.1	Senterica	WP_001292402.1
1017264	000212975.1	Bpertussis	WP_003808393.1
1028307	000215745.1	Eaerogenes	YP_004590711.1
1028307	000215745.1	Eaerogenes	YP_004590857.1
1028307	000215745.1	Eaerogenes	YP_004592539.1
1028307	000215745.1	Eaerogenes	YP_004592843.1
1028799	000245495.1	Saureus	WP_000812832.1
1029718	000270205.1	Carthromitus	WP_005805101.1
1029718	000270205.1	Carthromitus	WP_007442964.1
1029979	000330485.2	Senterica	WP_000978691.1
1029979	000330485.2	Senterica	WP_001021054.1
1029979	000330485.2	Senterica	WP_001100652.1
1029979	000330485.2	Senterica	WP_001292402.1
1029983	000487615.2	Senterica	WP_000839733.1
1029983	000487615.2	Senterica	WP_000978694.1
1029983	000487615.2	Senterica	WP_001021052.1
1029983	000487615.2	Senterica	WP_001100652.1
1029983	000487615.2	Senterica	WP_001292402.1
1030009	000218305.1	Lmonocytogene	WP_012582337.1
1031711	000215325.1	Rsolanacearum	WP_014616404.1
1032480	000270245.1	Mphosphovorus	WP_013861653.1
1032480	000270245.1	Mphosphovorus	WP_013864385.1
1033813	000214765.2	Ecoli	AEG34998.1
1033813	000214765.2	Ecoli	AEG35451.1
1033813	000214765.2	Ecoli	AEG37856.1
1033813	000214765.2	Ecoli	AEG39145.1
1033813	000214765.2	Ecoli	AEG39163.1
1033837	000214785.1	Lkefiranofaci	WP_013854165.1
1033991	000520875.1	Rleguminosaru	WP_025416941.1
1033991	000520875.1	Rleguminosaru	WP_025416942.1
1033991	000520875.1	Rleguminosaru	WP_025419142.1
1034809	000270465.1	Slugdunensis	WP_002460725.1

1034809	000270465.1	Slugdunensis	WP_002479477.1
1034836	000221645.1	Bamyloliquefa	WP_013350743.1
1034836	000221645.1	Bamyloliquefa	WP_014470448.1
1035377	000222975.1	Ypestis	WP_002210002.1
1035377	000222975.1	Ypestis	WP_002210805.1
1036672	000219915.3	Akashmirensis	X1
1036673	000218915.1	Pmucilaginosu	WP_013917650.1
1036673	000218915.1	Pmucilaginosu	WP_013921265.1
103690	000009705.1	Nsp	WP_010999013.1
1037409	000284375.1	Bjaponicum	WP_014493537.1
1037409	000284375.1	Bjaponicum	WP_014496637.1
1037911	000262325.2	Pfluorescens	WP_014718259.1
103855	001078275.1	Bhinzii	WP_029578266.1
103855	001078295.1	Bhinzii	WP_029578266.1
1041504	000283555.1	Carthromitus	WP_014094806.1
1041504	000283555.1	Carthromitus	WP_014095272.1
1041809	000284435.1	Carthromitus	WP_005805101.1
1041809	000284435.1	Carthromitus	WP_007442964.1
1042156	000270305.1	Csp	WP_013977075.1
1042163	000219535.3	Blaterosporus	AIG24465.1
1042163	000219535.3	Blaterosporus	AIG27859.1
1042876	000219705.1	Pputida	WP_013971954.1
1042876	000219705.1	Pputida	WP_024087408.1
1042878	000219215.1	Cnecator	WP_013957766.1
1045856	000239975.1	Ecloacae	WP_014168717.1
1045856	000239975.1	Ecloacae	WP_014169153.1
1045856	000239975.1	Ecloacae	WP_041162848.1
1048245	000253375.1	Mcanettii	WP_003899363.1
1048254	000986765.1	Ecoli	WP_000839766.1
1048254	000986765.1	Ecoli	WP_000978647.1
1048254	000986765.1	Ecoli	WP_001020973.1
1048254	000986765.1	Ecoli	WP_001295383.1
1048254	000986765.1	Ecoli	WP_001297242.1
1048260	000299235.1	Lferriphilum	WP_014960850.1
1048689	000245515.1	Ecoli	WP_000839816.1
1048689	000245515.1	Ecoli	WP_000978640.1
1048689	000245515.1	Ecoli	WP_001020954.1
1048689	000245515.1	Ecoli	WP_001292415.1
1048689	000245515.1	Ecoli	WP_001295383.1
1048834	000219875.1	Aacidocaldari	WP_014463054.1
1048834	000219875.1	Aacidocaldari	WP_041695065.1
1049565	000220485.1	Kpneumoniae	WP_002889384.1
1049565	000220485.1	Kpneumoniae	WP_002892486.1

1049565	000220485.1	Kpneumoniae	WP_004900586.1
1050617	000220005.2	Ecoli	AEJ54845.1
1050617	000220005.2	Ecoli	AEJ55315.1
1050617	000220005.2	Ecoli	AEJ58297.1
1050617	000220005.2	Ecoli	AEJ59522.1
1050617	000220005.2	Ecoli	AEJ59538.1
1051632	000219855.1	Sacidophilus	WP_013985131.1
1051650	000309565.2	Lcasei	WP_003564627.1
1051974	000576185.1	Gbethesdensis	WP_011631230.1
1052004	000576085.1	Gbethesdensis	WP_025286072.1
1052095	000576145.1	Gbethesdensis	WP_025319693.1
105219	000954135.2	Rmannitolilyt	AJW45905.1
1052585	000227465.1	Bsubtilis	WP_014112287.1
1052585	000227465.1	Bsubtilis	WP_014113664.1
1052588	000227485.1	Bsubtilis	WP_014475563.1
1052588	000227485.1	Bsubtilis	WP_014476699.1
1052684	000237325.1	Ppolymyxa	WP_013368657.1
1052684	000237325.1	Ppolymyxa	WP_013371896.1
1054460	000221985.1	Spseudopneumo	WP_000732150.1
1055524	000236215.4	Bcenocepacia	CDN61025.1
1055524	000236215.4	Bcenocepacia	CDN62791.1
1055524	000236215.4	Bcenocepacia	CDN62792.1
1064551	000473275.1	Senterica	WP_000978690.1
1064551	000473275.1	Senterica	WP_001021052.1
1064551	000473275.1	Senterica	WP_001100652.1
1064551	000473275.1	Senterica	WP_001292402.1
1064551	000473275.1	Senterica	WP_017465457.1
1068978	000739085.1	Amethanolica	WP_017982109.1
1072459	000227625.1	Ecoli	WP_000040180.1
1072459	000227625.1	Ecoli	WP_000839780.1
1072459	000227625.1	Ecoli	WP_000978629.1
1072459	000227625.1	Ecoli	WP_001021033.1
1072459	000227625.1	Ecoli	WP_001296667.1
1072685	000743945.1	Bpsittacipulm	WP_038499081.1
1073571	000981585.1	Priograndensi	CQR51066.1
1073571	000981585.1	Priograndensi	CQR55269.1
1073999	001277255.1	Ccondimenti	WP_007666643.1
1073999	001277255.1	Ccondimenti	WP_007674953.1
1073999	001277255.1	Ccondimenti	WP_007676785.1
1074000	001277175.1	Cuniversalis	WP_007697437.1
1074000	001277175.1	Cuniversalis	WP_007701740.1
1074252	000284535.1	Saureus	WP_000812832.1
1074467	000831645.3	Lheilongjiang	WP_041501004.1

1074919	000382965.1	Saureus	WP_000812829.1
1075089	000234745.1	Pmultocida	WP_014326116.1
1076550	000759475.1	Prwandensis	WP_038644888.1
1076550	000759475.1	Prwandensis	WP_038648899.1
1078773	001483945.1	Hrubrisubalbi	WP_017453986.1
1080228	000284135.1	Ssp	WP_010871419.1
1080229	000284215.1	Ssp	WP_010871419.1
1080230	000284455.1	Ssp	WP_010871419.1
1081093	000235545.1	Senterica	WP_000978691.1
1081093	000235545.1	Senterica	WP_001021054.1
1081093	000235545.1	Senterica	WP_001100652.1
1081093	000235545.1	Senterica	WP_001292399.1
1087440	000281535.2	Kpneumoniae	WP_002889384.1
1087440	000281535.2	Kpneumoniae	WP_002892486.1
1087440	000281535.2	Kpneumoniae	WP_004151766.1
1087448	000299435.1	Eantarcticum	WP_014969080.1
1087448	000299435.1	Eantarcticum	WP_014970808.1
1088720	000233755.1	Lrhamnosus	WP_005689694.1
1089456	000284555.1	Paeruginosa	WP_003087961.1
1089456	000284555.1	Paeruginosa	WP_014603687.1
1089553	000305935.1	Tphaeum	WP_015051903.1
1091041	000242855.2	Bamyloliquefa	WP_015388285.1
1091041	000242855.2	Bamyloliquefa	WP_015388791.1
1091500	000277085.1	Mtuberculosis	AFE17368.1
1093787	000271365.1	Paeruginosa	WP_003087961.1
1093787	000271365.1	Paeruginosa	WP_031655407.1
1093790	000275645.1	Vcholerae	WP_001019827.1
1093790	000275645.1	Vcholerae	WP_001086685.1
1094170	000281435.2	Kpneumoniae	WP_002889384.1
1094170	000281435.2	Kpneumoniae	WP_002892486.1
1094170	000281435.2	Kpneumoniae	WP_004151766.1
1094508	000307585.1	Tsaccharolyti	WP_014757572.1
1094508	000307585.1	Tsaccharolyti	WP_014757684.1
1094508	000307585.1	Tsaccharolyti	WP_014758510.1
1095774	000233595.1	Pananatis	WP_014605104.1
1095774	000233595.1	Pananatis	WP_014606547.1
1097668	000236065.1	Bsp	WP_014190694.1
1104326	000235765.3	Ecloacae	AFM58576.1
1104326	000235765.3	Ecloacae	AFM59024.1
1104326	000235765.3	Ecloacae	AFM61522.1
110662	000012625.1	Ssp	WP_011364581.1
1110693	000350185.1	Ecoli	WP_000040195.1
1110693	000350185.1	Ecoli	WP_000978651.1

1110693	000350185.1	Ecoli	WP_001020973.1
1110693	000350185.1	Ecoli	WP_001295383.1
1110693	000350185.1	Ecoli	WP_001326492.1
1111068	000236605.1	Gthermoleovor	WP_011230565.1
1111068	000236605.1	Gthermoleovor	WP_025039043.1
1112204	000247715.1	Gpolyisopreni	WP_006370623.1
1114958	000283695.1	Bvelezensis	WP_014304189.1
1114958	000283695.1	Bvelezensis	WP_014304948.1
1116375	000241385.1	Vsp	WP_014234789.1
1116375	000241385.1	Vsp	WP_014234790.1
1116391	000250655.1	Pmucilaginosu	WP_014371513.1
1116391	000250655.1	Pmucilaginosu	WP_014372601.1
1117647	000305785.2	Sagarivorans	WP_016389371.1
111780	000317575.1	Scyanosphaera	WP_015192159.1
111781	000316605.1	Lsp	WP_015133912.1
1118959	000237125.1	Saureus	WP_000812840.1
1121088	000832905.1	Bcoagulans	WP_029141723.1
1121088	000832905.1	Bcoagulans	WP_029141899.1
1121308	001077535.1	Pdifficile	AKP41941.1
1121308	001077535.1	Pdifficile	AKP44470.1
1121335	000331995.1	Cstercorarium	WP_015358917.1
1121335	000331995.1	Cstercorarium	WP_015359462.1
1123015	001045685.1	Paeruginosa	WP_003087961.1
1123015	001045685.1	Paeruginosa	WP_003159884.1
1123016	000818015.1	Pbalearica	WP_043219989.1
1123519	000279165.1	Pstutzeri	WP_014852913.1
1123523	000239235.1	Saureus	WP_000812837.1
1123862	000512165.1	Kpneumoniae	WP_002889384.1
1123862	000512165.1	Kpneumoniae	WP_002892486.1
1123862	000512165.1	Kpneumoniae	WP_025367889.1
1123863	000283875.1	Pananatis	WP_014606547.1
1123863	000283875.1	Pananatis	WP_015699572.1
1123863	000283875.1	Pananatis	WP_015701049.1
1124917	000484195.2	Senterica	WP_000839737.1
1124917	000484195.2	Senterica	WP_000978690.1
1124917	000484195.2	Senterica	WP_000992186.1
1124917	000484195.2	Senterica	WP_001021045.1
1124917	000484195.2	Senterica	WP_001100652.1
1124917	000484195.2	Senterica	WP_001292402.1
1124936	000430105.1	Senterica	WP_000839725.1
1124936	000430105.1	Senterica	WP_000978689.1
1124936	000430105.1	Senterica	WP_001021052.1
1124936	000430105.1	Senterica	WP_001100652.1

1124936	000430105.1	Senterica	WP_001292402.1
1124961	000486765.2	Senterica	WP_000978690.1
1124961	000486765.2	Senterica	WP_001021044.1
1124961	000486765.2	Senterica	WP_001100652.1
1124961	000486765.2	Senterica	WP_001292402.1
1124961	000486765.2	Senterica	WP_023212702.1
1124983	000397205.1	Pprotegens	WP_015635813.1
1124991	000286435.2	Mmorganii	WP_004238543.1
1124991	000286435.2	Mmorganii	WP_015422912.1
1125630	000240185.2	Kpneumoniae	AEW59631.1
1125630	000240185.2	Kpneumoniae	AEW60007.1
1125630	000240185.2	Kpneumoniae	AEW63166.1
1126011	000258905.1	Lmonocytogene	WP_012681468.1
1126211	000262385.1	Bamyloliquefa	WP_014416716.1
1126211	000262385.1	Bamyloliquefa	WP_014417652.1
1126833	000961095.1	Pbeijingensis	WP_045670334.1
1126833	000961095.1	Pbeijingensis	WP_045671838.1
1127134	000284035.1	Ncyriacigeorg	WP_014350450.1
1127744	000259365.1	Bsp	WP_014662472.1
1127744	000259365.1	Bsp	WP_014663836.1
1128398	000299355.1	Cacidurici	WP_014966327.1
1128398	000299355.1	Cacidurici	WP_014966358.1
1128398	000299355.1	Cacidurici	WP_041701716.1
1130804	000251085.2	Spneumoniae	WP_000661006.1
1131442	000723425.2	Mmarinum	WP_012393949.1
1131462	000305815.1	Dsp	WP_015042284.1
1131462	000305815.1	Dsp	WP_015044044.1
1132496	000255915.1	Pmultocida	WP_014391207.1
1132507	000245535.1	Senterica	WP_000978690.1
1132507	000245535.1	Senterica	WP_001021059.1
1132507	000245535.1	Senterica	WP_001100652.1
1133671	001011135.1	Ftularensis	WP_003020112.1
1133849	000250675.3	Nbrasiliensis	AFU01781.1
1133850	000245355.1	Shygroscopicu	WP_014676795.1
1133852	000299455.1	Ecoli	YP_006777644.1
1133852	000299455.1	Ecoli	YP_006780311.1
1133852	000299455.1	Ecoli	YP_006780792.1
1133852	000299455.1	Ecoli	YP_006781292.1
1133852	000299455.1	Ecoli	YP_006781307.1
1133853	000299475.1	Ecoli	WP_000839766.1
1133853	000299475.1	Ecoli	WP_000978647.1
1133853	000299475.1	Ecoli	WP_001020973.1
1133853	000299475.1	Ecoli	WP_001295383.1

1133853	000299475.1	Ecoli	WP_001297242.1
1134456	000250855.1	Vcholerae	WP_001019827.1
1134456	000250855.1	Vcholerae	WP_001086685.1
1134687	000963575.1	Kmichiganensi	WP_045781411.1
1134687	000963575.1	Kmichiganensi	WP_045783945.1
1134782	000299255.1	Ecoli	WP_000839766.1
1134782	000299255.1	Ecoli	WP_000978647.1
1134782	000299255.1	Ecoli	WP_001020973.1
1134782	000299255.1	Ecoli	WP_001295383.1
1134782	000299255.1	Ecoli	WP_001297242.1
1136873	000971925.1	Bsubtilis	WP_003243352.1
1136873	000971925.1	Bsubtilis	WP_003244780.1
1138308	000263215.1	Csakazakii	WP_012125704.1
1138308	000263215.1	Csakazakii	WP_014728051.1
1138382	000277145.1	Mintracellula	WP_009955898.1
1138382	000277145.1	Mintracellula	WP_014382302.1
1138383	000276825.1	Mintracellula	WP_008255597.1
1138383	000276825.1	Mintracellula	WP_014385281.1
1138871	000418535.2	Myongonense	AGP63333.1
1138871	000418535.2	Myongonense	AGP64743.1
1138877	000331445.1	Mtuberculosis	WP_003899363.1
1140	000012525.1	Selongatus	WP_011377691.1
1145276	000600105.1	Lvarians	WP_024362198.1
1145276	000600105.1	Lvarians	WP_025218324.1
114615	000026145.1	Bsp	WP_011927730.1
1147	000478825.2	Ssp	WP_028947653.1
1147129	000305775.1	Dsp	WP_015042284.1
1147129	000305775.1	Dsp	WP_015044044.1
1147130	000512395.1	Pmultocida	WP_014326116.1
1147161	000344745.1	Bsubtilis	WP_003243352.1
1147161	000344745.1	Bsubtilis	WP_003244780.1
1147786	000257545.3	Pmandelii	WP_010459804.1
1148	000009725.1	Ssp	WP_010871419.1
1150475	000341875.1	Bvelezensis	WP_015416648.1
1150475	000341875.1	Bvelezensis	WP_015417400.1
1150476	000455585.1	Bvelezensis	WP_007409666.1
1150476	000455585.1	Bvelezensis	WP_020955207.1
1151116	000255535.1	Raquatilis	WP_013576854.1
1151116	000255535.1	Raquatilis	WP_015689430.1
1155777	000284395.1	Bvelezensis	WP_014416716.1
1155777	000284395.1	Bvelezensis	WP_014417652.1
1156913	000400635.2	Aorientalis	WP_016336488.1
1157951	000259175.1	Pstuartii	WP_004923888.1

1157951	000259175.1	Pstuartii	WP_014656883.1
1159083	000348705.1	Spneumoniae	WP_000661011.1
1159554	001277235.1	Cdublinensis	WP_007709569.1
1159554	001277235.1	Cdublinensis	WP_007718137.1
1159554	001277235.1	Cdublinensis	WP_007728046.1
1159613	001277195.1	Cmuytjensii	WP_038864107.1
1159613	001277195.1	Cmuytjensii	WP_038864673.1
1159613	001277195.1	Cmuytjensii	WP_038867748.1
1160717	000258365.1	Senterica	WP_000839725.1
1160717	000258365.1	Senterica	WP_000978689.1
1160717	000258365.1	Senterica	WP_001021052.1
1160717	000258365.1	Senterica	WP_001100652.1
1160717	000258365.1	Senterica	WP_001292402.1
1160720	001433715.1	Astellipolari	WP_057790397.1
1160721	000723465.1	Rbicirculans	WP_046439735.1
1160721	000723465.1	Rbicirculans	WP_051706626.1
1162668	000284315.1	Lferrooxidans	WP_014449684.1
1163389	000262205.1	Fnoatunensis	WP_014715045.1
1163399	000284595.1	Smaltophilia	WP_014647525.1
1163617	000297055.2	Sdenitrifican	WP_009204871.1
1166016	000260925.1	Psp	WP_014700965.1
1166130	000410515.1	Esp	WP_020455189.1
1166130	000410515.1	Esp	WP_020456618.1
1168287	000262165.1	Msp	WP_008255597.1
1168287	000262165.1	Msp	WP_008258230.1
1170562	000317435.1	Csp	WP_015196752.1
1171376	000380325.1	Senterica	WP_000839738.1
1171376	000380325.1	Senterica	WP_000978695.1
1171376	000380325.1	Senterica	WP_001021054.1
1171376	000380325.1	Senterica	WP_001100652.1
1171376	000380325.1	Senterica	WP_001292402.1
1172205	000765395.1	Bholmesii	WP_005015296.1
1172205	000765395.1	Bholmesii	WP_005015527.1
1173022	000317495.1	Cepipsammum	WP_015205041.1
1173025	000317045.1	Gsp	WP_041268428.1
1173026	000317555.1	Gsp	WP_015187115.1
1173027	000317515.1	Msp	WP_015183795.1
1173263	000317085.1	Ssp	WP_015169506.1
1173427	000439415.1	Senterica	WP_000978690.1
1173427	000439415.1	Senterica	WP_001021054.1
1173427	000439415.1	Senterica	WP_001100652.1
1173427	000439415.1	Senterica	WP_001292402.1
1173427	000439415.1	Senterica	WP_020839028.1

1176492	000960995.1	Bglumae	WP_012734936.1
1176492	000960995.1	Bglumae	WP_015876723.1
1179773	000328705.1	Sespanaensis	WP_015099625.1
1179773	000328705.1	Sespanaensis	WP_015102759.1
1179773	000328705.1	Sespanaensis	WP_041317975.1
118163	000317025.1	Psp	WP_015141848.1
1182590	000297075.2	Ppseudoalcali	WP_003461428.1
1183438	000484535.1	Gkilaueensis	WP_051382630.1
1185650	000770175.1	Mabscessus	WP_005058249.1
1185650	000770215.1	Mabscessus	WP_005058249.1
1191061	000276705.2	Koxytoca	WP_004848492.1
1191061	000276705.2	Koxytoca	WP_014839541.1
1191062	001406795.1	Ctestosteroni	WP_046460760.1
1192196	000321395.1	Bsubtilis	WP_003244780.1
1192197	000271665.2	Pfermentans	WP_045820656.1
1193292	000294365.1	Kpneumoniae	WP_002889384.1
1193292	000294365.1	Kpneumoniae	WP_002892486.1
1193292	000294365.1	Kpneumoniae	WP_014906933.1
1193576	000463055.1	Saureus	WP_020978038.1
1194085	000470865.1	Saureus	WP_000812840.1
1194154	000487775.2	Senterica	AHW21355.1
1194154	000487775.2	Senterica	AHW22583.1
1194154	000487775.2	Senterica	AHW23098.1
1194154	000487775.2	Senterica	AHW24571.1
1194154	000487775.2	Senterica	AHW25080.1
1194526	000332735.1	Swarneri	WP_002465708.1
1194971	001011095.1	Lsalivarius	WP_047035011.1
1195464	000300475.1	Bthuringiensi	WP_000084886.1
1195464	000300475.1	Bthuringiensi	WP_001072283.1
1196325	000281215.1	Pputida	YP_006534749.1
1196835	000267545.1	Pstutzeri	WP_014820124.1
1197719	000439255.1	Sbongori	WP_000978697.1
1197719	000439255.1	Sbongori	WP_001100654.1
1197719	000439255.1	Sbongori	WP_001292414.1
1197719	000439255.1	Sbongori	WP_020842715.1
1197719	000439255.1	Sbongori	WP_020845214.1
1198114	000178975.2	Gtundricola	WP_013581383.1
119856	000833475.1	Ftularensis	WP_003020112.1
119857	000833235.1	Ftularensis	WP_003018000.1
1198627	000277775.2	Mabscessus	WP_005058249.1
1198676	000271865.1	Smutans	WP_014834840.1
119912	000742815.1	Senterica	WP_001539477.1
119912	000742815.1	Senterica	WP_023234833.1

119912	000742815.1	Senterica	WP_023235145.1
119912	000742815.1	Senterica	WP_024148683.1
119912	000742815.1	Senterica	WP_038394036.1
1199150	000714635.1	Kpneumoniae	AIE00322.1
1199150	000714635.1	Kpneumoniae	AIE01858.1
1199150	000714635.1	Kpneumoniae	AIE02136.1
1199187	000390085.1	Mavium	WP_003872520.1
1199187	000390085.1	Mavium	WP_003872624.1
1201010	000470845.1	Saureus	WP_000812840.1
1202450	000340435.3	Mhaemophilum	AKN16760.1
1202450	000340435.3	Mhaemophilum	AKN17391.1
1203460	000340845.1	Shygroscopicu	WP_014676795.1
1204342	000523045.1	Bsubtilis	WP_003243352.1
1204342	000523045.1	Bsubtilis	WP_003244780.1
1204343	000328745.1	Bsubtilis	BAM48958.1
1204343	000328745.1	Bsubtilis	BAM50496.1
1204343	000328745.1	Bsubtilis	BAM52109.1
1206780	000338715.2	Mbovis	WP_003899363.1
1207075	000316175.1	Psp	WP_015094585.1
1207075	000316175.1	Psp	WP_015095372.1
1207075	000316175.1	Psp	WP_015095547.1
1208611	000487915.2	Senterica	WP_000978690.1
1208611	000487915.2	Senterica	WP_001100652.1
1208611	000487915.2	Senterica	WP_023214799.1
1208611	000487915.2	Senterica	WP_023262937.1
1208611	000487915.2	Senterica	WP_023262962.1
1208658	000317955.1	Bbronchisepti	WP_003816357.1
1208660	000317935.1	Bparapertussi	YP_006894817.1
1208918	000340825.1	Ckinetoplasti	WP_041572063.1
1208919	000340795.1	Ckinetoplasti	WP_015396059.1
1208920	000340865.1	Ckinetoplasti	WP_015396803.1
1208921	000340905.1	Ckinetoplasti	WP_015389297.1
1208922	000340925.1	Ckinetoplasti	WP_015237812.1
1208923	000319245.1	Ckinetoplasti	WP_015237812.1
1209989	000213235.1	Tacetatoxydan	WP_013777148.1
1209989	000213235.1	Tacetatoxydan	WP_013779501.1
1211025	000286275.1	Ecloacae	WP_014882641.1
1211025	000286275.1	Ecloacae	WP_014883022.1
1211025	000286275.1	Ecloacae	WP_014885213.1
1211579	000412675.1	Pputida	WP_016498738.1
1211705	000328405.1	Vparahaemolyt	WP_005481757.1
1211705	000328405.1	Vparahaemolyt	WP_011106546.1
1212491	000953135.1	Lfallonii	WP_045094554.1

1214242	000444875.1	Scollinus	WP_020937797.1
1215087	000495455.2	Pputida	X1
1215088	000325725.1	Pputida	WP_015271062.1
1215914	000318035.1	Lcasei	WP_014952000.1
1215914	000318035.1	Lcasei	WP_014952001.1
1216932	000577895.1	Cbornimense	CDM67326.1
1216932	000577895.1	Cbornimense	CDM69239.1
1216976	000508285.1	Axylosoxidans	YP_008925927.1
1216976	000508285.1	Axylosoxidans	YP_008927152.1
1217721	000725385.1	Djaponica	WP_019465080.1
1217737	000292705.1	Bthuringiensi	WP_000084903.1
1217737	000292705.1	Bthuringiensi	WP_001073008.1
1217984	000292415.1	Bcereus	WP_000084887.1
1217984	000292415.1	Bcereus	WP_001075280.1
1218175	000292455.1	Bthuringiensi	WP_000084895.1
1218175	000292455.1	Bthuringiensi	WP_001073007.1
1218933	000294535.1	Pcarotovorum	WP_014914377.1
1219076	000354175.2	Valginolyticu	WP_005481757.1
1220533	000293765.1	Bsubtilis	WP_003243352.1
1220533	000293765.1	Bsubtilis	WP_003244780.1
1221328	000699525.1	Bsubtilis	WP_003243352.1
1221328	000699525.1	Bsubtilis	WP_003244780.1
1221522	000293885.3	Pfluorescens	AKV08491.1
1221522	000293885.3	Pfluorescens	AKV09100.1
1221524	000454025.1	Amediterranei	WP_013226148.1
1221524	000454025.1	Amediterranei	WP_013229207.1
1223802	000828635.1	Shydrogenivor	WP_041100603.1
1225181	000714675.1	Kpneumoniae	WP_002889384.1
1225181	000714675.1	Kpneumoniae	WP_002892486.1
1225181	000714675.1	Kpneumoniae	WP_004151766.1
1225522	000462995.1	Senterica	WP_000978691.1
1225522	000462995.1	Senterica	WP_001021054.1
1225522	000462995.1	Senterica	WP_001100652.1
1225522	000462995.1	Senterica	WP_001292402.1
1225788	000319475.1	Bvelezensis	WP_015239017.1
1225788	000319475.1	Bvelezensis	WP_015239787.1
1226298	000829035.1	Lparacasei	WP_003660671.1
1228987	000829235.1	cendosymbiont	WP_044106803.1
1229205	000300095.1	Pphenoliruptr	WP_013587492.1
1229492	000296595.1	Saureus	WP_000812828.1
1229671	000829075.1	Mavium	WP_023868764.1
1229671	000829075.1	Mavium	WP_023869511.1
1229785	000294635.1	Bpseudomallei	WP_004191939.1

1230340	000318055.1	Lmonocytogene	WP_010959076.1
1230587	000304415.1	Smeliloti	WP_014989906.1
1230587	000304415.1	Smeliloti	WP_014989907.1
1231072	000967115.1	Ctetani	WP_023437041.1
1231072	000967115.1	Ctetani	WP_023439256.1
1232394	000313385.1	Ftularensis	WP_003018000.1
1232554	000699465.1	Bsubtilis	WP_003243352.1
1232554	000699465.1	Bsubtilis	WP_003244780.1
1232724	000298095.1	Mindicus	WP_008255597.1
1232724	000298095.1	Mindicus	WP_008258230.1
1233100	000338735.1	Bsubtilis	WP_014479695.1
1233100	000338735.1	Bsubtilis	WP_015382555.1
1233873	000336445.1	Gsp	WP_011230565.1
1233873	000336445.1	Gsp	WP_015373697.1
1234142	000382945.1	Lmonocytogene	YP_007608304.1
1234679	000317975.2	Cmaltaromatic	WP_015077659.1
1235461	000320385.2	Smeliloti	WP_015242485.1
1235461	000320385.2	Smeliloti	WP_015242486.1
1235834	000300455.3	Ksacchari	AHJ75169.1
1235834	000300455.3	Ksacchari	AHJ76612.1
1239307	000517425.1	Spraecaptivus	WP_025420957.1
1239307	000517425.1	Spraecaptivus	WP_025421123.1
1241582	000567925.1	Bthailandensi	WP_009892476.1
1241583	000494855.1	Bpseudomallei	WP_004191939.1
1241616	001027105.1	Saureus	WP_000812832.1
1243618	000626315.1	Senterica	WP_000978691.1
1243618	000626315.1	Senterica	WP_001021050.1
1243618	000626315.1	Senterica	WP_001100652.1
1243618	000626315.1	Senterica	WP_001292402.1
1243619	000626175.1	Senterica	WP_000839732.1
1243619	000626175.1	Senterica	WP_000978691.1
1243619	000626175.1	Senterica	WP_001021050.1
1243619	000626175.1	Senterica	WP_001100652.1
1243619	000626175.1	Senterica	WP_001292402.1
1243620	000626295.1	Senterica	WP_000839732.1
1243620	000626295.1	Senterica	WP_000978691.1
1243620	000626295.1	Senterica	WP_001021050.1
1243620	000626295.1	Senterica	WP_001100652.1
1243620	000626295.1	Senterica	WP_001292402.1
1243621	000623375.1	Senterica	WP_000839732.1
1243621	000623375.1	Senterica	WP_000978691.1
1243621	000623375.1	Senterica	WP_001021050.1
1243621	000623375.1	Senterica	WP_001100652.1

1243621	000623375.1	Senterica	WP_001292402.1
1244085	000474015.1	Kpneumoniae	WP_002892486.1
1244085	000474015.1	Kpneumoniae	WP_004149820.1
1244085	000474015.1	Kpneumoniae	WP_004194933.1
1244111	000623355.1	Senterica	WP_000839732.1
1244111	000623355.1	Senterica	WP_000978691.1
1244111	000623355.1	Senterica	WP_001021050.1
1244111	000623355.1	Senterica	WP_001100652.1
1244111	000623355.1	Senterica	WP_001292402.1
1244112	000623335.1	Senterica	WP_000839732.1
1244112	000623335.1	Senterica	WP_000978691.1
1244112	000623335.1	Senterica	WP_001100652.1
1244112	000623335.1	Senterica	WP_001292402.1
1244112	000623335.1	Senterica	WP_038397931.1
1244113	000623315.1	Senterica	WP_000839732.1
1244113	000623315.1	Senterica	WP_000978691.1
1244113	000623315.1	Senterica	WP_001021050.1
1244113	000623315.1	Senterica	WP_001100652.1
1244113	000623315.1	Senterica	WP_001292402.1
1244118	000623295.1	Senterica	WP_000839732.1
1244118	000623295.1	Senterica	WP_000978691.1
1244118	000623295.1	Senterica	WP_001021050.1
1244118	000623295.1	Senterica	WP_001100652.1
1244118	000623295.1	Senterica	WP_001292402.1
1244119	000623275.1	Senterica	WP_000839732.1
1244119	000623275.1	Senterica	WP_000978691.1
1244119	000623275.1	Senterica	WP_001021050.1
1244119	000623275.1	Senterica	WP_001100652.1
1244119	000623275.1	Senterica	WP_001292402.1
1244120	000626375.1	Senterica	WP_000839732.1
1244120	000626375.1	Senterica	WP_000978691.1
1244120	000626375.1	Senterica	WP_001021050.1
1244120	000626375.1	Senterica	WP_001100652.1
1244120	000626375.1	Senterica	WP_001292402.1
1244121	000626335.1	Senterica	WP_000839732.1
1244121	000626335.1	Senterica	WP_000978691.1
1244121	000626335.1	Senterica	WP_001021050.1
1244121	000626335.1	Senterica	WP_001100652.1
1244121	000626335.1	Senterica	WP_001292402.1
1244122	000626355.1	Senterica	WP_000839732.1
1244122	000626355.1	Senterica	WP_000978691.1
1244122	000626355.1	Senterica	WP_001021050.1
1244122	000626355.1	Senterica	WP_001100652.1

1244122	000626355.1	Senterica	WP_001292402.1
1245469	000344805.1	Boligotrophic	WP_015665899.1
1245471	000412695.1	Presinovorans	WP_016493540.1
1245474	000800215.1	Ecoli	WP_000040195.1
1245474	000800215.1	Ecoli	WP_000978651.1
1245474	000800215.1	Ecoli	WP_001020973.1
1245474	000800215.1	Ecoli	WP_001295383.1
1245474	000800215.1	Ecoli	WP_001326492.1
1246301	000463015.1	Vparadoxus	WP_021007410.1
1246626	000706725.1	Blehensis	WP_038484823.1
1246626	000706725.1	Blehensis	WP_038485807.1
1246626	000706725.1	Blehensis	WP_051667296.1
1247649	000612485.1	Bholmesii	WP_005015296.1
1247726	000521505.1	Amimigardefor	WP_025374028.1
1248823	000671295.1	Ecoli	WP_000839810.1
1248823	000671295.1	Ecoli	WP_000978649.1
1248823	000671295.1	Ecoli	WP_001021032.1
1248823	000671295.1	Ecoli	WP_001295383.1
1248902	000520035.1	Ecoli	WP_000839810.1
1248902	000520035.1	Ecoli	WP_000978649.1
1248902	000520035.1	Ecoli	WP_001021032.1
1248902	000520035.1	Ecoli	WP_001295383.1
1248903	000662395.1	Ecoli	WP_000839810.1
1248903	000662395.1	Ecoli	WP_000978649.1
1248903	000662395.1	Ecoli	WP_001021032.1
1248903	000662395.1	Ecoli	WP_001295383.1
1248915	000520055.1	Ecoli	WP_000839810.1
1248915	000520055.1	Ecoli	WP_000978649.1
1248915	000520055.1	Ecoli	WP_001021032.1
1248915	000520055.1	Ecoli	WP_001295383.1
1249468	000511895.1	Bpseudomallei	WP_004191939.1
1249471	000521645.1	Bpseudomallei	WP_004191939.1
1249472	000959225.1	Bpseudomallei	WP_004191939.1
1249473	000959205.1	Bpseudomallei	WP_004191939.1
1249474	000520895.1	Bpseudomallei	WP_004191939.1
1249475	000959185.1	Bpseudomallei	WP_004191939.1
1249476	000511915.1	Bpseudomallei	WP_004191939.1
1249477	000764575.1	Bpseudomallei	WP_004191939.1
1249615	000698475.1	Mtuberculosis	WP_003899363.1
1249634	000330865.1	Smarcescens	WP_015673544.1
1249634	000330865.1	Smarcescens	WP_015673810.1
1249658	000959345.1	Bpseudomallei	WP_004191939.1
1249659	000583835.1	Bpseudomallei	WP_004191939.1

1249660	000959425.1	Bthailandensi	WP_009892476.1
1249661	000385525.1	Bthailandensi	WP_015602470.1
1249662	000567945.1	Bthailandensi	WP_009892476.1
1249663	000567905.1	Bthailandensi	WP_009892476.1
1249664	000959605.1	Bthailandensi	WP_009892476.1
1249665	000765375.1	Bthailandensi	WP_009892476.1
1249667	000706745.1	Bthailandensi	WP_009892476.1
1249668	000959245.1	Bubonensis	WP_042586066.1
1250339	000764595.1	Bthailandensi	WP_009892476.1
1259844	000511325.1	Psp	WP_025339933.1
1261127	000981805.1	Camalonaticus	WP_046475836.1
1261127	000981805.1	Camalonaticus	WP_046494034.1
1261127	000981805.1	Camalonaticus	WP_046497192.1
1261127	000981805.1	Camalonaticus	WP_046497676.1
1261129	000717535.1	Bthuringiensi	WP_001075296.1
1261129	000717535.1	Bthuringiensi	WP_042969788.1
1262449	000807175.1	Cpasteurianum	WP_003440309.1
1262449	000807175.1	Cpasteurianum	WP_003443643.1
1262462	000968115.1	Yenterocoliti	WP_005175347.1
1262462	000968115.1	Yenterocoliti	WP_046050275.1
1262462	000968115.1	Yenterocoliti	WP_046050279.1
1262462	000968115.1	Yenterocoliti	WP_046050355.1
1262470	001267925.1	Hhiltneri	WP_053200590.1
1263871	000364385.2	Kpneumoniae	WP_002889384.1
1263871	000364385.2	Kpneumoniae	WP_002892486.1
1263871	000364385.2	Kpneumoniae	WP_004151766.1
1266717	000478385.1	Spseudinterme	WP_037543982.1
1266738	000444425.1	Pmirabilis	WP_020945294.1
1266738	000444425.1	Pmirabilis	WP_041701401.1
1266845	000493735.1	Cinhibens	WP_023176701.1
1266925	000619905.2	Nbriensis	WP_025040599.1
1267562	001281465.1	Cgilardii	WP_053821152.1
1267577	000319225.1	Ckinetoplasti	WP_041572063.1
1267753	000341425.1	Senterica	WP_000978690.1
1267753	000341425.1	Senterica	WP_001021044.1
1267753	000341425.1	Senterica	WP_001100652.1
1267753	000341425.1	Senterica	WP_001292402.1
1267753	000341425.1	Senterica	WP_001527964.1
1268072	000612505.1	Psabinae	WP_025332566.1
1268072	000612505.1	Psabinae	WP_025334631.1
1271862	000430145.3	Senterica	AGQ66689.1
1271862	000430145.3	Senterica	AGQ67048.1
1271862	000430145.3	Senterica	AGQ68562.1

1271862	000430145.3	Senterica	AGQ68969.1
1271862	000430145.3	Senterica	AGQ70848.1
1271863	000430125.1	Senterica	WP_000978690.1
1271863	000430125.1	Senterica	WP_001021045.1
1271863	000430125.1	Senterica	WP_001292402.1
1271863	000430125.1	Senterica	WP_020899093.1
1271863	000430125.1	Senterica	WP_020899203.1
1271864	000430085.2	Senterica	WP_000839725.1
1271864	000430085.2	Senterica	WP_000978689.1
1271864	000430085.2	Senterica	WP_001021052.1
1271864	000430085.2	Senterica	WP_001100652.1
1271864	000430085.2	Senterica	WP_001292402.1
1273687	000416365.2	Msp	WP_003884938.1
1274814	000332755.1	Ecoli	WP_000839748.1
1274814	000332755.1	Ecoli	WP_000978647.1
1274814	000332755.1	Ecoli	WP_001020973.1
1274814	000332755.1	Ecoli	WP_001295383.1
1274814	000332755.1	Ecoli	WP_001372249.1
1276282	000494875.1	Spasteuri	WP_023374600.1
1276647	000390245.1	Ssuis	WP_015647048.1
1278073	000331735.1	Mstipitatus	WP_063639755.1
1279007	000496605.2	Paeruginosa	WP_003087961.1
1279007	000496605.2	Paeruginosa	WP_023086414.1
1279008	000496645.1	Paeruginosa	WP_003087961.1
1279008	000496645.1	Paeruginosa	WP_023086414.1
1279365	000338755.1	Bthuringiensi	WP_000084904.1
1279365	000338755.1	Bthuringiensi	WP_001075296.1
1280	000597965.1	Saureus	WP_000812829.1
1280	000626615.1	Saureus	WP_000812832.1
1280	000695875.1	Saureus	WP_000812827.1
1280	000709475.1	Saureus	AID38972.1
1280	000746505.1	Saureus	WP_000812832.1
1280	000815045.1	Saureus	WP_000812827.1
1280	000953255.1	Saureus	WP_000812850.1
1280	001021875.1	Saureus	WP_000812827.1
1280	001021895.1	Saureus	WP_000812827.1
1280	001027045.1	Saureus	WP_047928173.1
1280	001045795.2	Saureus	WP_000812827.1
1280	001045995.2	Saureus	WP_000812827.1
1280	001046095.2	Saureus	WP_000812827.1
1280	001278745.1	Saureus	WP_000812829.1
1280	001281145.1	Saureus	WP_000812829.1
1280	001444345.1	Saureus	WP_031927772.1

1280	001457495.1	Saureus	WP_000812837.1
1280	001457515.1	Saureus	WP_000812832.1
1280	001465635.1	Saureus	WP_000812828.1
1280	001465675.1	Saureus	WP_000812828.1
1280	001465755.1	Saureus	WP_000812828.1
1280380	000737535.1	Ssp	WP_051847368.1
1280938	000359505.1	Paeruginosa	WP_003087961.1
1280938	000359505.1	Paeruginosa	WP_014603687.1
1282	000759555.1	Sepidermidis	WP_001832221.1
1282357	000743955.1	Sflexneri	WP_000978635.1
1282357	000743955.1	Sflexneri	WP_001020991.1
1282357	000743955.1	Sflexneri	WP_005085661.1
1282358	000743995.1	Sflexneri	WP_000978635.1
1282358	000743995.1	Sflexneri	WP_001020991.1
1282358	000743995.1	Sflexneri	WP_005085661.1
1283	000972725.1	Shaemolyticus	WP_016931269.1
1283330	000380335.1	Avinelandii	WP_012701511.1
1283331	000380365.1	Avinelandii	WP_012701511.1
1284	000816085.1	Shyicus	WP_039647147.1
1284798	000417225.2	Kpneumoniae	WP_002889384.1
1284798	000417225.2	Kpneumoniae	WP_002892486.1
1284798	000417225.2	Kpneumoniae	WP_004151766.1
1284802	000417085.2	Kpneumoniae	WP_002889384.1
1284802	000417085.2	Kpneumoniae	WP_002892486.1
1284802	000417085.2	Kpneumoniae	WP_004151766.1
1284804	000417265.2	Kpneumoniae	WP_002889384.1
1284804	000417265.2	Kpneumoniae	WP_002892486.1
1284804	000417265.2	Kpneumoniae	WP_004151766.1
1286170	000367425.1	Rornithinolyt	WP_004858936.1
1286170	000367425.1	Rornithinolyt	WP_015585100.1
1286170	000367425.1	Rornithinolyt	WP_015585737.1
1286171	000597865.1	Eacidaminophi	WP_025434596.1
1286171	000597865.1	Eacidaminophi	WP_025434632.1
1286171	000597865.1	Eacidaminophi	WP_038602829.1
1286404	000341665.1	Bthuringiensi	WP_000084899.1
1286404	000341665.1	Bthuringiensi	WP_001073005.1
1286640	000346065.1	Smeliloti	WP_010967353.1
1286640	000346065.1	Smeliloti	WP_015445418.1
1288	000706685.1	Sxylosus	WP_038678827.1
1288	000709415.1	Sxylosus	WP_042363336.1
1288	000953575.1	Sxylosus	WP_047172872.1
1288122	000348565.1	Episcicida	WP_012847161.1
1288122	000348565.1	Episcicida	WP_012847625.1

1288122	000348565.1	Episcicida	WP_015462316.1
1288295	000577745.1	Lmonocytogene	WP_031669215.1
1288394	000401555.1	Ahydrophila	WP_016349881.1
1289591	000696675.2	Rerythropolis	WP_029256704.1
1292358	000835145.1	Bamyloliquefa	WP_007409666.1
1292358	000835145.1	Bamyloliquefa	WP_020955207.1
1294143	000349845.1	Pdenitrifican	WP_015476902.1
1295	001188855.1	Sschleiferi	WP_050346102.1
1295	001188875.1	Sschleiferi	WP_050331813.1
1295	001188895.1	Sschleiferi	WP_050331813.1
1295	001188915.1	Sschleiferi	WP_050331813.1
1298917	000444445.1	Senterica	WP_000978691.1
1298917	000444445.1	Senterica	WP_001021054.1
1298917	000444445.1	Senterica	WP_001292402.1
1298917	000444445.1	Senterica	WP_020936842.1
1299044	000505705.1	Senterica	WP_000839725.1
1299044	000505705.1	Senterica	WP_000978689.1
1299044	000505705.1	Senterica	WP_001021052.1
1299044	000505705.1	Senterica	WP_001100652.1
1299044	000505705.1	Senterica	WP_001292402.1
1299325	000758385.1	Mabscessus	WP_005111050.1
1299326	000831265.1	Mkansasii	WP_023372340.1
1299326	000831265.1	Mkansasii	WP_023373082.1
1299328	000831305.1	Mkansasii	WP_023372340.1
1299328	000831305.1	Mkansasii	WP_023373082.1
1299329	000831285.1	Mavium	WP_009977695.1
1299329	000831285.1	Mavium	WP_011724548.1
1299330	000758285.1	Mavium	WP_009977695.1
1299330	000758285.1	Mavium	WP_038430418.1
1299331	000767485.1	Mintracellula	WP_036458648.1
1299331	000767485.1	Mintracellula	WP_038536717.1
1301098	000689415.1	Pknackmussii	WP_043249262.1
1301098	000689415.1	Pknackmussii	WP_043251261.1
1302650	000349795.1	Bsubtilis	WP_014479695.1
1302650	000349795.1	Bsubtilis	WP_015482688.1
1303024	000445035.1	Mabscessus	WP_005058249.1
13035	000317615.1	Dsalina	WP_015230502.1
1304279	000389905.1	Mtuberculosis	X1
1304890	000973565.1	Pmultocida	WP_005716647.1
1304922	000406765.2	Kpneumoniae	WP_002889384.1
1304922	000406765.2	Kpneumoniae	WP_002892486.1
1304922	000406765.2	Kpneumoniae	WP_004151766.1
1306400	000364825.1	Mtuberculosis	WP_003899363.1

1306414	000422125.1	Mtuberculosis	WP_003904863.1
1306417	000755945.1	Bpseudomallei	WP_004191939.1
1306418	000755925.1	Bpseudomallei	WP_004191939.1
1306419	000755905.1	Bpseudomallei	WP_004191939.1
1306420	000755965.1	Bpseudomallei	WP_004191939.1
1306421	000756065.1	Bpseudomallei	WP_004191939.1
1307427	001296125.1	Fnucleatum	ALF20036.1
1307428	001296165.1	Fnucleatum	WP_029598527.1
1307442	001455145.1	Fhwasookii	WP_029493265.1
1307443	001455085.1	Fhwasookii	WP_029491153.1
1307444	001455105.1	Fhwasookii	WP_029491153.1
1307839	001443605.1	bl21spid4	WP_057954844.1
1308980	000632415.1	Koxytoca	WP_004848492.1
1308980	000632415.1	Koxytoca	WP_014226846.1
1308980	000632415.1	Koxytoca	WP_014228008.1
1310114	000389925.1	Mtuberculosis	YP_007962417.1
1310115	000389945.1	Mtuberculosis	WP_015631450.1
1311757	001051135.1	Ecoli	WP_000839748.1
1311757	001051135.1	Ecoli	WP_001020973.1
1311757	001051135.1	Ecoli	WP_001295383.1
1311757	001051135.1	Ecoli	WP_001372249.1
1311757	001051135.1	Ecoli	WP_048943316.1
1313	000817005.1	Spneumoniae	WP_016399315.1
1313	001255215.1	Asp	WP_053039567.1
1313	001457635.1	Spneumoniae	WP_000661011.1
1314884	000389675.2	Lacidophilus	WP_003547166.1
1315283	001465295.1	Ptranslucida	WP_058372989.1
1316933	000418475.1	Lrhamnosus	WP_005711611.1
1318466	000968055.1	Apalmae	WP_026654684.1
1318634	000418495.1	Lrhamnosus	WP_005689694.1
1318635	000418515.1	Lcasei	WP_003564627.1
1321369	000418345.1	Saureus	WP_000812832.1
1322345	000743255.1	Ecoli	WP_000839820.1
1322345	000743255.1	Ecoli	WP_000978663.1
1322345	000743255.1	Ecoli	WP_001021003.1
1322345	000743255.1	Ecoli	WP_001296667.1
1322345	000743255.1	Ecoli	WP_001524296.1
1323661	000412775.1	Saureus	WP_000812839.1
1323664	000522545.2	Pcaribensis	WP_036003465.1
1327989	000695995.1	Ssp	WP_004931974.1
1327989	000695995.1	Ssp	WP_004931982.1
1327989	000695995.1	Ssp	WP_044029859.1
1327989	000695995.1	Ssp	WP_044031971.1

1328314	000817975.1	Achroococcum	WP_039803738.1
1328324	000695935.1	Kpneumoniae	WP_002889384.1
1328324	000695935.1	Kpneumoniae	WP_002892486.1
1328324	000695935.1	Kpneumoniae	WP_004185624.1
1328325	000717515.1	Kpneumoniae	WP_002889384.1
1328325	000717515.1	Kpneumoniae	WP_002892486.1
1328325	000717515.1	Kpneumoniae	WP_004151766.1
1328859	000730345.1	Ecoli	WP_000839815.1
1328859	000730345.1	Ecoli	WP_000978640.1
1328859	000730345.1	Ecoli	WP_001020954.1
1328859	000730345.1	Ecoli	WP_001295383.1
1328859	000730345.1	Ecoli	WP_038431160.1
1329907	000813165.1	Ecoli	WP_000839776.1
1329907	000813165.1	Ecoli	WP_000978670.1
1329907	000813165.1	Ecoli	WP_001296667.1
1329907	000813165.1	Ecoli	WP_001362902.1
1330043	000831065.1	Bbombysepticu	WP_000084897.1
1330043	000831065.1	Bbombysepticu	WP_001075294.1
1330457	000803705.1	Ecoli	WP_000839815.1
1330457	000803705.1	Ecoli	WP_000978640.1
1330457	000803705.1	Ecoli	WP_001020954.1
1330457	000803705.1	Ecoli	WP_001292415.1
1330457	000803705.1	Ecoli	WP_001295383.1
1331671	000410575.1	Pputida	WP_016488236.1
1333534	000993825.1	Pdurus	WP_046723086.1
1333534	000993825.1	Pdurus	WP_046723765.1
1333848	000648515.1	Cfreundii	WP_038633637.1
1333848	000648515.1	Cfreundii	WP_038636438.1
1333848	000648515.1	Cfreundii	WP_038637430.1
1333849	000750275.1	Ecloacae	WP_022647114.1
1333849	000750275.1	Ecloacae	WP_022649374.1
1333849	000750275.1	Ecloacae	WP_038415690.1
1333850	000724505.1	Ecloacae	WP_015571121.1
1333850	000724505.1	Ecloacae	WP_015572729.1
1333850	000724505.1	Ecloacae	WP_032620254.1
1333851	000750225.1	Ecloacae	WP_022647114.1
1333851	000750225.1	Ecloacae	WP_022649374.1
1333851	000750225.1	Ecloacae	WP_038415690.1
1333852	000714655.1	Koxytoca	WP_004848492.1
1333852	000714655.1	Koxytoca	WP_014226846.1
1333852	000714655.1	Koxytoca	WP_014228008.1
1334187	000465255.1	Hinfluenzae	WP_021034546.1
1334564	000828775.1	Smarcescens	WP_038878804.1

1334564	000828775.1	Smarcescens	WP_039567427.1
1334564	000828775.1	Smarcescens	WP_041037622.1
1334564	000828775.1	Smarcescens	WP_041038110.1
1334565	000582845.1	Lmonocytogene	WP_014601195.1
1334629	000988565.1	Mfulvus	WP_063789775.1
1334632	000963495.1	Pfluorescens	WP_010210911.1
1335307	000439695.1	Bpseudomallei	WP_004191939.1
1335916	000468515.1	Ecoli	WP_000839749.1
1335916	000468515.1	Ecoli	WP_000978651.1
1335916	000468515.1	Ecoli	WP_001021030.1
1335916	000468515.1	Ecoli	WP_001295383.1
1335916	000468515.1	Ecoli	WP_001297242.1
13373	000755785.1	Bmallei	WP_004191939.1
13373	000755845.1	Bmallei	WP_024900677.1
13373	000755865.1	Bmallei	WP_004191939.1
13373	000755885.1	Bmallei	WP_004191939.1
13373	000756025.2	Bmallei	AIO80392.1
13373	000959165.1	Bmallei	WP_004191939.1
13373	000959405.1	Bmallei	WP_004191939.1
13373	000959465.1	Bmallei	WP_004191939.1
13373	000959485.1	Bmallei	WP_024900677.1
13373	000959625.1	Bmallei	WP_004191939.1
1337936	000734895.2	Csp	WP_035156405.1
1338032	000430425.1	Vparahaemolyt	WP_005481757.1
1338032	000430425.1	Vparahaemolyt	WP_011106546.1
1338034	000430405.1	Vparahaemolyt	WP_005481757.1
1338034	000430405.1	Vparahaemolyt	WP_011106546.1
1338518	000455565.1	Bvelezensis	WP_007409666.1
1338518	000455565.1	Bvelezensis	WP_007409917.1
1340851	000414035.1	Paeruginosa	WP_003087961.1
1340851	000414035.1	Paeruginosa	WP_016561700.1
1341656	000978785.2	Ftularensis	WP_003020112.1
1341692	000484505.1	Cautoethanoge	WP_013236828.1
1341692	000484505.1	Cautoethanoge	WP_013240050.1
1343064	000828035.1	Saureus	WP_000812832.1
134537	000961515.1	Pfungorum	WP_046566445.1
1345695	000473995.1	Csaccharobuty	WP_022744075.1
1345695	000473995.1	Csaccharobuty	WP_022748984.1
1345697	000445995.2	Gsp	WP_020959190.1
1345697	000445995.2	Gsp	WP_023817403.1
1345702	001188815.1	Ypestis	WP_002210002.1
1345702	001188815.1	Ypestis	WP_002210805.1
1345703	001188695.1	Ypestis	WP_002210002.1

1345704	001188935.1	Ypestis	WP_002210002.1
1345705	001188715.1	Ypestis	WP_002210002.1
1345706	001188795.1	Ypestis	WP_002210002.1
1345707	001188775.1	Ypestis	WP_002210002.1
1345708	001188755.1	Ypestis	WP_002210002.1
134601	001187505.1	Mgoodii	WP_049744266.1
1346614	000422085.1	Sliquefaciens	WP_020824970.1
1346614	000422085.1	Sliquefaciens	WP_020828344.1
1346614	000422085.1	Sliquefaciens	WP_020828350.1
1346614	000422085.1	Sliquefaciens	WP_020828616.1
134676	000237145.1	Asp	WP_014691690.1
134676	000237145.1	Asp	WP_043514437.1
1348623	000832985.1	Bmegaterium	WP_013056060.1
1348623	000832985.1	Bmegaterium	WP_034652899.1
1348623	000832985.1	Bmegaterium	WP_034654916.1
1348660	000438825.1	Splymuthica	WP_006327522.1
1348660	000438825.1	Splymuthica	WP_006327912.1
1349767	000723165.1	Jagaricidamno	WP_038491775.1
1349819	001442755.1	Asp	WP_061974322.1
1350461	000817325.1	Ssp	WP_011377691.1
1354030	000512375.1	Ecloacae	WP_025203245.1
1354030	000512375.1	Ecloacae	WP_025204662.1
1354030	000512375.1	Ecloacae	WP_025205149.1
1354304	000968175.1	Xpoinarii	WP_045958736.1
135461	000827065.1	Bsubtilis	WP_003243352.1
135461	000827065.1	Bsubtilis	WP_003244780.1
1355100	000493755.1	Ecoli	WP_000839781.1
1355100	000493755.1	Ecoli	WP_000978629.1
1355100	000493755.1	Ecoli	WP_001021005.1
1355100	000493755.1	Ecoli	WP_001296667.1
1355100	000493755.1	Ecoli	WP_001531487.1
1356861	000505725.1	Fnoatunensis	WP_014715045.1
135735	000972245.2	Bendophyticus	WP_019393000.1
135735	000972245.2	Bendophyticus	WP_040059935.1
1358422	001029125.1	Ecoli	WP_000671170.1
1358422	001029125.1	Ecoli	WP_000978651.1
1358422	001029125.1	Ecoli	WP_001020950.1
1358422	001029125.1	Ecoli	WP_001295383.1
1358422	001029125.1	Ecoli	WP_001741343.1
1358422	001029125.1	Ecoli	WP_032298507.1
1366050	000471925.1	Rpickettii	WP_022536352.1
1367477	000473245.1	Binfantis	WP_009791103.1
1367477	000473245.1	Binfantis	WP_035401349.1

1367847	000444995.1	Paminophilus	WP_020952439.1
1368166	000737615.1	Saureus	WP_000812840.1
1374	001465835.1	Pkocurii	WP_058384823.1
1374	001465835.1	Pkocurii	WP_058386804.1
137722	000010725.1	Asp	WP_012978210.1
1379159	000632805.1	Djiangningens	WP_038618574.1
1379159	000632805.1	Djiangningens	WP_051595365.1
1380908	000445405.1	Kpneumoniae	WP_002889384.1
1380908	000445405.1	Kpneumoniae	WP_002892486.1
1380908	000445405.1	Kpneumoniae	WP_004151766.1
138119	000010045.1	Dhafniense	WP_005808143.1
138119	000010045.1	Dhafniense	WP_005815018.1
1382510	000709535.1	Sbongori	WP_000978697.1
1382510	000709535.1	Sbongori	WP_001100654.1
1382510	000709535.1	Sbongori	WP_038390535.1
1382510	000709535.1	Sbongori	WP_038392601.1
1382700	000493595.1	Ecoli	WP_000839776.1
1382700	000493595.1	Ecoli	WP_000978670.1
1382700	000493595.1	Ecoli	WP_001021000.1
1382700	000493595.1	Ecoli	WP_001296667.1
1382700	000493595.1	Ecoli	WP_001362902.1
1384061	000498395.3	Pputida	AJQ51128.1
1385727	000493375.1	Bvelezensis	WP_017417709.1
1385727	000493375.1	Bvelezensis	WP_022552467.1
1385755	000474035.1	sescherichia	AGX32367.1
1385755	000474035.1	sescherichia	AGX32817.1
1385755	000474035.1	sescherichia	AGX34958.1
1385755	000474035.1	sescherichia	AGX36028.1
1385755	000474035.1	sescherichia	AGX36043.1
1386967	000756465.1	Senterica	WP_000978611.1
1386967	000756465.1	Senterica	WP_001100646.1
1386967	000756465.1	Senterica	WP_038396532.1
1386967	000756465.1	Senterica	WP_038396918.1
1386967	000756465.1	Senterica	WP_038397652.1
1388763	000498975.2	Pmosselii	WP_023629445.1
1390	000833005.1	Bamyloliquefa	WP_043866770.1
1390	000833005.1	Bamyloliquefa	WP_043867068.1
1390	000973485.1	Bamyloliquefa	WP_043866770.1
1390	000973485.1	Bamyloliquefa	WP_043867068.1
1390	001023595.1	Bamyloliquefa	WP_007409666.1
1390	001023595.1	Bamyloliquefa	WP_007409917.1
1390	001483885.1	Bamyloliquefa	WP_014304189.1
1390	001483885.1	Bamyloliquefa	WP_048367222.1

1390363	001042525.1	Fnoatunensis	WP_014715045.1
1392	000725325.1	Banthracis	WP_000084880.1
1392	000725325.1	Banthracis	WP_001075286.1
1392	000742655.1	Banthracis	WP_000084880.1
1392	000742655.1	Banthracis	WP_001075263.1
1392	000747335.1	Banthracis	WP_000084880.1
1392	000747335.1	Banthracis	WP_001075286.1
1392	000747375.1	Banthracis	WP_000084880.1
1392	000747375.1	Banthracis	WP_001075286.1
1392	000830095.1	Banthracis	WP_000084880.1
1392	000830095.1	Banthracis	WP_001075286.1
1392	000832425.1	Banthracis	WP_000084880.1
1392	000832425.1	Banthracis	WP_001075286.1
1392	000832445.1	Banthracis	WP_000084880.1
1392	000832445.1	Banthracis	WP_001075286.1
1392	000832465.1	Banthracis	WP_000084880.1
1392	000832465.1	Banthracis	WP_001075286.1
1392	000832505.1	Banthracis	WP_000084880.1
1392	000832505.1	Banthracis	WP_001075286.1
1392	000832565.1	Banthracis	WP_000084880.1
1392	000832565.1	Banthracis	WP_001075286.1
1392	000832585.1	Banthracis	WP_000084880.1
1392	000832585.1	Banthracis	WP_001075286.1
1392	000832665.1	Banthracis	WP_000084880.1
1392	000832665.1	Banthracis	WP_001075286.1
1392	000832725.1	Banthracis	WP_000084880.1
1392	000832725.1	Banthracis	WP_001075286.1
1392	000832745.1	Banthracis	WP_000084880.1
1392	000832745.1	Banthracis	WP_001075286.1
1392	000832965.1	Banthracis	WP_000084880.1
1392	000832965.1	Banthracis	WP_001075263.1
1392	000833065.1	Banthracis	WP_000084880.1
1392	000833065.1	Banthracis	WP_001075286.1
1392	000833125.1	Banthracis	WP_000084880.1
1392	000833125.1	Banthracis	WP_001075286.1
1392	000875715.1	Banthracis	WP_000084880.1
1392	000875715.1	Banthracis	WP_001075286.1
1392005	000739375.1	Ctestosteroni	WP_043372148.1
1392476	000462955.1	Saureus	WP_020977008.1
1392499	000814805.1	Kpneumoniae	WP_002889384.1
1392499	000814805.1	Kpneumoniae	WP_002892486.1
1392499	000814805.1	Kpneumoniae	WP_004900586.1
1392500	000813205.1	Kpneumoniae	WP_002892486.1

1392500	000813205.1	Kpneumoniae	WP_004149820.1
1392500	000813205.1	Kpneumoniae	WP_004194933.1
1392837	000583105.1	Banthracis	WP_000084880.1
1392837	000583105.1	Banthracis	WP_025441071.1
1396	000724585.1	Bcereus	WP_000084889.1
1396	000724585.1	Bcereus	WP_042874151.1
1396	000724585.1	Bcereus	WP_042876191.1
1396	000789315.1	Bcereus	WP_001980832.1
1396	000789315.1	Bcereus	WP_033692457.1
1396	000832525.1	Bcereus	WP_033692457.1
1396	000832525.1	Bcereus	WP_042515820.1
1396	000832765.1	Bcereus	WP_000084880.1
1396	000832765.1	Bcereus	WP_001075276.1
1396	000835185.1	Bcereus	WP_000084880.1
1396	000835185.1	Bcereus	WP_001075276.1
1396	000978375.1	Bcereus	WP_000084899.1
1396	000978375.1	Bcereus	WP_006920522.1
1396	001277915.1	Bcereus	WP_000084899.1
1396	001277915.1	Bcereus	WP_053563254.1
1398	000876545.1	Bcoagulans	WP_014095608.1
1398	000876545.1	Bcoagulans	WP_017550884.1
1398	001039495.1	Bcoagulans	WP_014095608.1
1398	001039495.1	Bcoagulans	WP_017550884.1
1399115	000496635.1	Esp	WP_023466516.1
1399115	000496635.1	Esp	WP_023468847.1
1400868	000473745.3	Paeruginosa	AID84551.1
1400868	000473745.3	Paeruginosa	AIL00160.1
1401659	000504545.1	Csakazakii	WP_007781867.1
1401659	000504545.1	Csakazakii	WP_023897989.1
1401659	000504545.1	Csakazakii	WP_023899385.1
1403053	000812165.1	Bpertussis	WP_003808393.1
1403312	000814885.1	Lgasseri	X1
1403539	000952035.1	Slydicus	WP_046924194.1
1403831	000499485.1	Ecoli	WP_000040195.1
1403831	000499485.1	Ecoli	WP_000978651.1
1403831	000499485.1	Ecoli	WP_001020973.1
1403831	000499485.1	Ecoli	WP_001295383.1
1403831	000499485.1	Ecoli	WP_001326492.1
1404258	000706705.1	Bsubtilis	WP_014479695.1
1404258	000706705.1	Bsubtilis	WP_029726395.1
1405	000742855.1	Bmycoides	WP_018780882.1
1405	000742855.1	Bmycoides	WP_018783057.1
1405	000832605.1	Bmycoides	WP_002128878.1

1405	000832605.1	Bmycoides	WP_002130200.1
1406	000819665.1	Ppolymyxa	WP_013368657.1
1406	000819665.1	Ppolymyxa	WP_013371896.1
1406314	000733255.1	Kpneumoniae	WP_002892486.1
1406314	000733255.1	Kpneumoniae	WP_004194933.1
1406314	000733255.1	Kpneumoniae	WP_043906894.1
1406860	000503845.1	Senterica	WP_000839737.1
1406860	000503845.1	Senterica	WP_000978690.1
1406860	000503845.1	Senterica	WP_000992186.1
1406860	000503845.1	Senterica	WP_001021045.1
1406860	000503845.1	Senterica	WP_001100652.1
1406860	000503845.1	Senterica	WP_001292402.1
1406863	000485885.1	Saureus	WP_000812832.1
1407062	000513415.1	Eamylovora	WP_004155423.1
1407062	000513415.1	Eamylovora	WP_004159429.1
1407063	000513395.1	Eamylovora	WP_004155423.1
1407063	000513395.1	Eamylovora	WP_004159429.1
1407064	000513355.1	Eamylovora	WP_004155423.1
1407064	000513355.1	Eamylovora	WP_004159429.1
1408	000590455.1	Bpumilus	WP_025206597.1
1408	000590455.1	Bpumilus	WP_035392179.1
1408	000972685.1	Bpumilus	WP_025206597.1
1408	000972685.1	Bpumilus	WP_046343453.1
1408	001191605.1	Bpumilus	WP_046343453.1
1408	001191605.1	Bpumilus	WP_050826831.1
1408	001431145.1	Bpumilus	WP_057078760.1
1408	001431145.1	Bpumilus	WP_057079595.1
1408	001431785.1	Bpumilus	WP_057078760.1
1408	001431785.1	Bpumilus	WP_057079595.1
1408186	000498675.1	Ljohnsonii	WP_023600088.1
1408272	000508765.1	Paeruginosa	WP_003113592.1
1408272	000508765.1	Paeruginosa	WP_016253674.1
1408283	000817935.1	Cbotulinum	WP_003360141.1
1408283	000817935.1	Cbotulinum	WP_003363053.1
1408285	000816945.1	Cbotulinum	AJD27092.1
1408285	000816945.1	Cbotulinum	AJD28601.1
1409	000952895.1	Bsubtilis	WP_003243352.1
1409	000952895.1	Bsubtilis	WP_003244780.1
1412451	000623175.1	Senterica	WP_000839732.1
1412451	000623175.1	Senterica	WP_000978691.1
1412451	000623175.1	Senterica	WP_001021050.1
1412451	000623175.1	Senterica	WP_001100652.1
1412451	000623175.1	Senterica	WP_001292402.1

1412453	000626275.2	Senterica	WP_000839732.1
1412453	000626275.2	Senterica	WP_000978691.1
1412453	000626275.2	Senterica	WP_001021050.1
1412453	000626275.2	Senterica	WP_001100652.1
1412453	000626275.2	Senterica	WP_001292402.1
1412454	000626255.1	Senterica	WP_000839732.1
1412454	000626255.1	Senterica	WP_000978691.1
1412454	000626255.1	Senterica	WP_001021050.1
1412454	000626255.1	Senterica	WP_001100652.1
1412454	000626255.1	Senterica	WP_001292402.1
1412455	000626235.1	Senterica	WP_000839732.1
1412455	000626235.1	Senterica	WP_000978691.1
1412455	000626235.1	Senterica	WP_001021050.1
1412455	000626235.1	Senterica	WP_001100652.1
1412455	000626235.1	Senterica	WP_001292402.1
1412457	000626215.1	Senterica	WP_000839732.1
1412457	000626215.1	Senterica	WP_000978691.1
1412457	000626215.1	Senterica	WP_001021050.1
1412457	000626215.1	Senterica	WP_001100652.1
1412457	000626215.1	Senterica	WP_001292402.1
1412458	000626115.1	Senterica	WP_000839732.1
1412458	000626115.1	Senterica	WP_000978691.1
1412458	000626115.1	Senterica	WP_001021050.1
1412458	000626115.1	Senterica	WP_001100652.1
1412458	000626115.1	Senterica	WP_001292402.1
1412459	000623775.1	Senterica	WP_000839732.1
1412459	000623775.1	Senterica	WP_000978691.1
1412459	000623775.1	Senterica	WP_001021050.1
1412459	000623775.1	Senterica	WP_001100652.1
1412459	000623775.1	Senterica	WP_001292402.1
1412460	000623755.1	Senterica	WP_000839732.1
1412460	000623755.1	Senterica	WP_000978691.1
1412460	000623755.1	Senterica	WP_001021050.1
1412460	000623755.1	Senterica	WP_001100652.1
1412460	000623755.1	Senterica	WP_001292402.1
1412461	000623795.1	Senterica	WP_000839732.1
1412461	000623795.1	Senterica	WP_000978691.1
1412461	000623795.1	Senterica	WP_001021050.1
1412461	000623795.1	Senterica	WP_001100652.1
1412461	000623795.1	Senterica	WP_001292402.1
1412462	000626155.1	Senterica	WP_000839732.1
1412462	000626155.1	Senterica	WP_000978691.1
1412462	000626155.1	Senterica	WP_001021050.1

1412462	000626155.1	Senterica	WP_001100652.1
1412462	000626155.1	Senterica	WP_001292402.1
1412463	000626135.1	Senterica	WP_000839732.1
1412463	000626135.1	Senterica	WP_000978691.1
1412463	000626135.1	Senterica	WP_001021050.1
1412463	000626135.1	Senterica	WP_001100652.1
1412463	000626135.1	Senterica	WP_001292402.1
1412464	000831045.1	Senterica	WP_000839732.1
1412464	000831045.1	Senterica	WP_000978691.1
1412464	000831045.1	Senterica	WP_001021050.1
1412464	000831045.1	Senterica	WP_001100652.1
1412464	000831045.1	Senterica	WP_001292402.1
1412465	000831025.1	Senterica	WP_000839732.1
1412465	000831025.1	Senterica	WP_000978691.1
1412465	000831025.1	Senterica	WP_001021050.1
1412465	000831025.1	Senterica	WP_001100652.1
1412465	000831025.1	Senterica	WP_001292402.1
1412466	000624395.2	Senterica	WP_000839732.1
1412466	000624395.2	Senterica	WP_000978691.1
1412466	000624395.2	Senterica	WP_001021050.1
1412466	000624395.2	Senterica	WP_001100652.1
1412466	000624395.2	Senterica	WP_001292402.1
1412468	000623195.2	Senterica	WP_000839732.1
1412468	000623195.2	Senterica	WP_000978691.1
1412468	000623195.2	Senterica	WP_001021050.1
1412468	000623195.2	Senterica	WP_001100652.1
1412468	000623195.2	Senterica	WP_001292402.1
1412470	000626195.1	Senterica	WP_000839732.1
1412470	000626195.1	Senterica	WP_000978691.1
1412470	000626195.1	Senterica	WP_001021050.1
1412470	000626195.1	Senterica	WP_001100652.1
1412470	000626195.1	Senterica	WP_001292402.1
1412471	000626395.1	Senterica	X1
1412475	000623155.1	Senterica	WP_000839732.1
1412475	000623155.1	Senterica	WP_000978691.1
1412475	000623155.1	Senterica	WP_001021050.1
1412475	000623155.1	Senterica	WP_001100652.1
1412475	000623155.1	Senterica	WP_001292402.1
1412478	000626495.1	Senterica	AHV62514.1
1412478	000626495.1	Senterica	AHV62713.1
1412478	000626495.1	Senterica	AHV63021.1
1412478	000626495.1	Senterica	AHV63078.1
1412478	000626495.1	Senterica	AHV63979.1

1412479	000626515.1	Senterica	AHV65531.1
1412479	000626515.1	Senterica	AHV65969.1
1412479	000626515.1	Senterica	AHV66836.1
1412479	000626515.1	Senterica	AHV67909.1
1412479	000626515.1	Senterica	AHV68244.1
1412480	000626475.1	Senterica	AHV56762.1
1412480	000626475.1	Senterica	AHV58258.1
1412480	000626475.1	Senterica	AHV58537.1
1412480	000626475.1	Senterica	AHV58906.1
1412480	000626475.1	Senterica	AHV59431.1
1412505	000626535.1	Senterica	AHV69277.1
1412505	000626535.1	Senterica	AHV69326.1
1412505	000626535.1	Senterica	AHV70562.1
1412505	000626535.1	Senterica	AHV72306.1
1412505	000626535.1	Senterica	AHV72758.1
1412570	000624155.1	Senterica	WP_000839732.1
1412570	000624155.1	Senterica	WP_000978691.1
1412570	000624155.1	Senterica	WP_001021050.1
1412570	000624155.1	Senterica	WP_001100652.1
1412570	000624155.1	Senterica	WP_001292402.1
1412592	000623055.2	Senterica	AHN98317.1
1412592	000623055.2	Senterica	AHN98977.2
1412592	000623055.2	Senterica	AHN99273.1
1412592	000623055.2	Senterica	AHN99437.1
1412592	000623055.2	Senterica	AHO00481.1
1412593	000623075.1	Senterica	AHO03847.1
1412593	000623075.1	Senterica	AHO04182.1
1412593	000623075.1	Senterica	AHO04214.1
1412593	000623075.1	Senterica	AHO04643.1
1412593	000623075.1	Senterica	AHO05057.1
1412593	000623075.1	Senterica	AHO05515.1
1412595	000623095.1	Senterica	AHO08669.1
1412595	000623095.1	Senterica	AHO09063.1
1412595	000623095.1	Senterica	AHO09586.1
1412595	000623095.1	Senterica	AHO09900.1
1412595	000623095.1	Senterica	AHO10540.1
1412595	000623095.1	Senterica	AHO10930.1
1412595	000623095.1	Senterica	AHO11601.1
1412596	000623115.1	Senterica	WP_000839732.1
1412596	000623115.1	Senterica	WP_001100652.1
1412596	000623115.1	Senterica	WP_001292402.1
1412596	000623115.1	Senterica	WP_052647739.1
1412596	000623115.1	Senterica	WP_052648206.1

1412597	000623135.1	Senterica	AHO18676.1
1412597	000623135.1	Senterica	AHO19114.1
1412597	000623135.1	Senterica	AHO19565.1
1412597	000623135.1	Senterica	AHO19865.1
1412597	000623135.1	Senterica	AHO20962.1
1412618	000626415.1	Senterica	WP_000839732.1
1412618	000626415.1	Senterica	WP_000978691.1
1412618	000626415.1	Senterica	WP_001021050.1
1412618	000626415.1	Senterica	WP_001100652.1
1412618	000626415.1	Senterica	WP_001292402.1
1412898	000494835.1	Bamyloliquefa	WP_011996166.1
1412898	000494835.1	Bamyloliquefa	WP_012117476.1
1413214	000597985.1	Ppolymyxa	WP_016822391.1
1413214	000597985.1	Ppolymyxa	WP_025363638.1
1415165	000508265.1	Bamyloliquefa	WP_024084790.1
1415165	000508265.1	Bamyloliquefa	WP_024085263.1
1415166	000523235.1	Nnova	WP_025349808.1
1415167	000497485.1	Bsubtilis	WP_003243352.1
1415167	000497485.1	Bsubtilis	WP_003244780.1
1415629	000504045.1	Paeruginosa	WP_003087961.1
1415629	000504045.1	Paeruginosa	WP_023875601.1
1415630	000508205.1	Psp	WP_024075108.1
1415774	000789355.1	Cbotulinum	WP_035784106.1
1415774	000789355.1	Cbotulinum	WP_035785269.1
1415775	000789395.1	Cbaratii	WP_039310919.1
1415784	000496285.1	Btoyonensis	WP_000084877.1
1415784	000496285.1	Btoyonensis	WP_001072283.1
1416914	000590495.2	Ppnomenusa	WP_023597105.1
1416915	001019645.1	Ahydrophila	WP_016349881.1
1418107	000635955.1	Ahydrophila	WP_016349881.1
1418110	000633175.1	Ahydrophila	WP_016349881.1
1419584	000819505.1	Ahydrophila	WP_016349881.1
1419814	000688775.1	Ssp	WP_044020797.1
1420012	000598005.1	Kpneumoniae	WP_002889384.1
1420012	000598005.1	Kpneumoniae	WP_002892486.1
1420012	000598005.1	Kpneumoniae	WP_004151766.1
1420013	000597905.1	Kpneumoniae	WP_002889384.1
1420013	000597905.1	Kpneumoniae	WP_002892486.1
1420013	000597905.1	Kpneumoniae	WP_004151766.1
1420599	000828695.1	Pprotegens	WP_041118482.1
1420885	000829215.1	Vcholerae	WP_001019826.1
1420885	000829215.1	Vcholerae	WP_001086685.1
1421338	000632395.1	Easburiae	WP_029740736.1

1421338	000632395.1	Easburiae	WP_029741180.1
1421338	000632395.1	Easburiae	WP_029741194.1
1423	000772125.1	Bsubtilis	WP_013350743.1
1423	000772125.1	Bsubtilis	WP_014470448.1
1423	000772165.1	Bsubtilis	WP_007409666.1
1423	000772165.1	Bsubtilis	WP_038463551.1
1423	000772205.1	Bsubtilis	WP_007409666.1
1423	000772205.1	Bsubtilis	WP_007409917.1
1423	000782835.1	Bsubtilis	WP_017694801.1
1423	000782835.1	Bsubtilis	WP_038428260.1
1423	000953615.1	Bsubtilis	WP_003243352.1
1423	000953615.1	Bsubtilis	WP_003244780.1
1423	000959025.1	Bsubtilis	WP_003218293.1
1423	000959025.1	Bsubtilis	WP_003221301.1
1423	001015095.1	Bsubtilis	WP_014479695.1
1423	001015095.1	Bsubtilis	WP_047181964.1
1423	001037985.1	Bsubtilis	WP_003226777.1
1423	001037985.1	Bsubtilis	WP_003244780.1
1423138	000685725.1	Bvelezensis	WP_015239787.1
1423138	000685725.1	Bvelezensis	WP_038456660.1
1426	001295365.1	Gthermoglucos	WP_003252032.1
1426	001295365.1	Gthermoglucos	WP_042385725.1
1427342	000510305.1	Paeruginosa	WP_003087961.1
1427342	000510305.1	Paeruginosa	WP_003159884.1
1427366	000816045.1	Dzeae	WP_016942161.1
1427516	000786505.1	Mtuberculosis	WP_003899363.1
1428	000774075.2	Bthuringiensi	ALL20333.1
1428	000832485.1	Bthuringiensi	WP_000084880.1
1428	000832485.1	Bthuringiensi	WP_001075274.1
1428	000832825.1	Bthuringiensi	WP_000084880.1
1428	000832825.1	Bthuringiensi	WP_001075278.1
1428	000832925.1	Bthuringiensi	WP_000084880.1
1428	000832925.1	Bthuringiensi	WP_042511790.1
1428	000833085.1	Bthuringiensi	WP_000084883.1
1428	000833085.1	Bthuringiensi	WP_001075273.1
1428	001017635.1	Bthuringiensi	WP_000084904.1
1428	001017635.1	Bthuringiensi	WP_001075296.1
1428	001182785.1	Bthuringiensi	WP_016080448.1
1428	001182785.1	Bthuringiensi	WP_050842384.1
1428	001420855.1	Bthuringiensi	WP_000084904.1
1428	001420855.1	Bthuringiensi	WP_001075296.1
1428	001455345.1	Bthuringiensi	WP_060629168.1
1428	001455345.1	Bthuringiensi	WP_060632071.1

1428454	000506785.2	Cpasteurianum	WP_012060567.1
1429044	000568495.1	Vparahaemolyt	WP_005481757.1
1429044	000568495.1	Vparahaemolyt	WP_011106546.1
1429244	000507205.2	Ppolymyxa	WP_013310938.1
1429244	000507205.2	Ppolymyxa	WP_023986423.1
1432056	000521605.1	Mvarigena	WP_025236159.1
1432652	000524575.1	Ftularensis	WP_003018000.1
1433144	001318185.1	Vcholerae	WP_001019827.1
1433144	001318185.1	Vcholerae	WP_001086685.1
1433287	000521655.1	Mvarigena	WP_025217104.1
1434214	000521695.1	Mvarigena	WP_025247603.1
1434215	000521685.1	Mvarigena	WP_025342700.1
1435044	000510285.1	Pmonteilii	WP_024086652.1
1435044	000510285.1	Pmonteilii	WP_024087408.1
1435046	001021855.1	Sflexneri	WP_000978662.1
1435046	001021855.1	Sflexneri	WP_001021013.1
1435046	001021855.1	Sflexneri	WP_005099260.1
1435058	000510325.1	Pmonteilii	WP_024086652.1
1435058	000510325.1	Pmonteilii	WP_024087408.1
1435365	000770495.1	Bpseudomallei	WP_004191939.1
1435461	000725305.1	Ecoli	WP_000040195.1
1435461	000725305.1	Ecoli	WP_000978651.1
1435461	000725305.1	Ecoli	WP_001020973.1
1435461	000725305.1	Ecoli	WP_001326492.1
1435461	000725305.1	Ecoli	WP_038430655.1
1435984	000770395.1	Bpseudomallei	WP_004191939.1
1437453	001013905.1	Sleeuwenhoeki	WP_029387594.1
1437823	000953355.1	Xnematophila	WP_013184234.1
1437824	000612685.1	Cdefragrans	WP_043684195.1
1437838	000613085.1	Lmonocytogene	WP_009930436.1
1437856	000827085.1	Mtuberculosis	WP_003899363.1
1439852	000770515.1	Bpseudomallei	WP_004191939.1
1439854	000770455.1	Bpseudomallei	WP_038761313.1
1439855	000770535.1	Bpseudomallei	WP_038761313.1
1440052	000512125.1	Ealbertii	WP_025237379.1
1440052	000512125.1	Ealbertii	WP_025237832.1
1440052	000512125.1	Ealbertii	WP_025238067.1
1441	000940785.1	Bthuringiensi	WP_000084903.1
1441	000940785.1	Bthuringiensi	WP_001073008.1
1441628	000695975.1	Lferriphilum	WP_014960850.1
1441629	000517305.1	Pcichorii	WP_051427759.1
1441930	000520015.2	Cmultitudinis	WP_024914264.1
1443113	000597945.1	Yenterocoliti	AHM72292.1

1443113	000597945.1	Yenterocoliti	AHM72467.1
1443113	000597945.1	Yenterocoliti	AHM75974.1
1445606	000828995.1	Mtuberculosis	WP_003899363.1
1446494	000582665.1	Lparacasei	WP_016365836.1
1446792	000815145.1	Bsp	WP_015417400.1
1446792	000815145.1	Bsp	WP_039253044.1
1448140	000524595.1	Paeruginosa	WP_003087961.1
1448140	000524595.1	Paeruginosa	WP_003159884.1
1449088	000583065.1	Bvelezensis	WP_025284078.1
1449088	000583065.1	Bvelezensis	WP_025284683.1
1449091	001042545.1	Fnoatunensis	WP_014715045.1
1449752	000751035.1	Sepidermidis	WP_001832221.1
1449752	000751035.1	Sepidermidis	WP_002494725.1
1449978	000959445.1	Bvietnamiensi	WP_011885706.1
1449978	000959445.1	Bvietnamiensi	WP_014725479.1
1449979	000832785.1	Banthracis	WP_000084880.1
1449979	000832785.1	Banthracis	WP_001075286.1
1450527	000833355.1	Ftularensis	WP_003035692.1
1452	000830075.1	Batrophaeus	WP_003328608.1
1452	000830075.1	Batrophaeus	WP_003329126.1
1452722	001050455.1	Bmegaterium	WP_028412631.1
1452722	001050455.1	Bmegaterium	WP_028414107.1
1452727	000833275.1	Banthracis	WP_000084880.1
1452727	000833275.1	Banthracis	WP_001075286.1
1452728	000833165.1	Ftularensis	WP_003035692.1
1452729	000835025.1	Bthuringiensi	WP_000084903.1
1452729	000835025.1	Bthuringiensi	WP_001073008.1
1453352	001430825.1	Ssp	WP_062059271.1
1453429	000025125.1	Catelocyanoba	WP_012953747.1
1453495	000834395.1	Yaldovae	WP_042546717.1
1453495	000834395.1	Yaldovae	WP_042547648.1
1453495	000834395.1	Yaldovae	WP_042548274.1
1453496	000597785.2	Halvei	WP_025798582.1
1453496	000597785.2	Halvei	WP_025798687.1
1453496	000597785.2	Halvei	WP_025800207.1
1453496	000597785.2	Halvei	WP_025801092.1
1453496	000597785.2	Halvei	WP_025801823.1
1453496	000597785.2	Halvei	WP_038503077.1
1453990	000973605.1	Bsubtilis	WP_014479695.1
1453990	000973605.1	Bsubtilis	WP_046340193.1
1454377	000834215.1	Yfrederikseni	WP_038630974.1
1454377	000834215.1	Yfrederikseni	WP_038636767.1
1454382	000832385.1	Bcereus	WP_000084880.1

1454382	000832385.1	Bcereus	WP_001075278.1
1454585	000988525.1	Senterica	WP_000978690.1
1454585	000988525.1	Senterica	WP_001100652.1
1454585	000988525.1	Senterica	WP_001292402.1
1454585	000988525.1	Senterica	WP_023243687.1
1454585	000988525.1	Senterica	WP_024155456.1
1454590	000963535.1	Senterica	WP_000978690.1
1454590	000963535.1	Senterica	WP_001100652.1
1454590	000963535.1	Senterica	WP_001292402.1
1454590	000963535.1	Senterica	WP_023243687.1
1454590	000963535.1	Senterica	WP_023244110.1
1454592	000940895.1	Senterica	WP_000978690.1
1454592	000940895.1	Senterica	WP_001100652.1
1454592	000940895.1	Senterica	WP_001292402.1
1454592	000940895.1	Senterica	WP_023243687.1
1454592	000940895.1	Senterica	WP_024155456.1
1454603	000940975.1	Senterica	WP_000839722.1
1454603	000940975.1	Senterica	WP_000978689.1
1454603	000940975.1	Senterica	WP_001021042.1
1454603	000940975.1	Senterica	WP_001100656.1
1454603	000940975.1	Senterica	WP_001292402.1
1454606	000973665.1	Senterica	WP_000839722.1
1454606	000973665.1	Senterica	WP_000978689.1
1454606	000973665.1	Senterica	WP_001021042.1
1454606	000973665.1	Senterica	WP_001100656.1
1454606	000973665.1	Senterica	WP_001292402.1
1454620	000940935.1	Senterica	WP_000839738.1
1454620	000940935.1	Senterica	WP_001021051.1
1454620	000940935.1	Senterica	WP_001100652.1
1454620	000940935.1	Senterica	WP_001292402.1
1454620	000940935.1	Senterica	WP_044810996.1
1454627	000973685.1	Senterica	WP_000839735.1
1454627	000973685.1	Senterica	WP_000978691.1
1454627	000973685.1	Senterica	WP_001021052.1
1454627	000973685.1	Senterica	WP_001100652.1
1454627	000973685.1	Senterica	WP_001292402.1
1454639	000941015.1	Senterica	WP_000839738.1
1454639	000941015.1	Senterica	WP_000978690.1
1454639	000941015.1	Senterica	WP_001021054.1
1454639	000941015.1	Senterica	WP_001100652.1
1454639	000941015.1	Senterica	WP_001292402.1
1454642	000973645.1	Senterica	WP_000839738.1
1454642	000973645.1	Senterica	WP_000978690.1

1454642	000973645.1	Senterica	WP_001021054.1
1454642	000973645.1	Senterica	WP_001100652.1
1454642	000973645.1	Senterica	WP_001292402.1
1457187	000568475.1	Lmonocytogene	WP_014601195.1
1457188	000568935.1	Lmonocytogene	WP_010959076.1
1457190	000565155.1	Livanovii	WP_014093876.1
1458206	000973585.1	Bvelezensis	WP_024084790.1
1458206	000973585.1	Bvelezensis	WP_024085263.1
1458279	000568455.1	Saureus	WP_000812827.1
1458425	000828895.1	Cbacterium	WP_045531334.1
1458426	000828915.1	Cbacterium	WP_045535419.1
1458465	001304715.1	Melsdenii	WP_027895148.1
1458465	001304715.1	Melsdenii	WP_054336204.1
1460371	000749465.2	Alurida	AJK59049.1
1461582	000756715.2	Jsp	CEA02602.1
1468409	000959325.1	Bsp	WP_006024902.1
146891	000015645.1	Pmarinus	WP_011818621.1
1469502	000828975.1	Bbacterium	WP_045473203.1
1479	001050115.1	Bsmithii	WP_048622322.1
1479	001050115.1	Bsmithii	WP_048623067.1
1484157	000784965.1	Psp	WP_038624192.1
1484157	000784965.1	Psp	WP_038628025.1
1484158	000784875.3	Psp	AIX49223.1
1484158	000784875.3	Psp	AIX50325.1
1491	000827935.1	Cbotulinum	WP_003373549.1
1491	000827935.1	Cbotulinum	WP_012451323.1
1491	000827955.1	Cbotulinum	WP_003373549.1
1491	000827955.1	Cbotulinum	WP_012451323.1
1491	000829015.1	Cbotulinum	WP_003386794.1
1491	000829015.1	Cbotulinum	WP_011947924.1
1492	001456065.2	Cbutyricum	WP_024039157.1
1492	001456065.2	Cbutyricum	WP_043853492.1
1492	001465175.1	Cbutyricum	WP_024039157.1
1492	001465175.1	Cbutyricum	WP_043853492.1
149539	000612325.1	Senterica	WP_000839732.1
149539	000612325.1	Senterica	WP_000978691.1
149539	000612325.1	Senterica	WP_001021050.1
149539	000612325.1	Senterica	WP_001100652.1
149539	000612325.1	Senterica	WP_001292402.1
1496	000953275.1	Pdifficile	WP_011860979.1
1496	000953275.1	Pdifficile	WP_011861997.1
1496	001447175.1	Pdifficile	WP_003437158.1
1496	001457575.1	Pdifficile	WP_011860979.1

1496	001457575.1	Pdifficile	WP_011861997.1
1499686	000756775.2	Psp	CDZ93440.1
1501268	000757845.1	Psp	WP_042850563.1
1501269	000757865.1	Psp	WP_038653504.1
1502	001304735.1	Cperfringens	WP_060669644.1
1502	001304735.1	Cperfringens	WP_060671151.1
150340	000024825.1	Vantiquarius	WP_012841314.1
150340	000024825.1	Vantiquarius	WP_041853108.1
1508404	000818095.1	Jmalaysiensis	WP_039806124.1
1508404	000818095.1	Jmalaysiensis	WP_039813425.1
1508644	000709435.1	Carthromitus	WP_005805101.1
1508644	000709435.1	Carthromitus	WP_007442964.1
1509	000973705.1	Csporogenes	WP_033058174.1
1509	000973705.1	Csporogenes	WP_033061095.1
1509	001020205.1	Csporogenes	WP_033058174.1
1509	001020205.1	Csporogenes	WP_033061095.1
1511	000196455.1	Csticklandii	WP_013360267.1
1511	000196455.1	Csticklandii	WP_013361042.1
1519377	001191625.1	Gsp	WP_050368466.1
152297	001455325.1	Pissachenkoni	WP_058154909.1
1525	001267405.1	Mthermoacetic	WP_051498625.1
1525	001267405.1	Mthermoacetic	WP_053095127.1
1525	001267435.1	Mthermoacetic	WP_051498625.1
1525	001267435.1	Mthermoacetic	WP_053095127.1
1526927	000785555.1	Psp	WP_038704367.1
1526927	000785555.1	Psp	WP_038705286.1
1536769	000758565.1	Psp	WP_039297911.1
1536769	000758565.1	Psp	WP_042132674.1
1536770	000758585.1	Psp	WP_042123076.1
1536770	000758585.1	Psp	WP_042126789.1
1536771	000758605.1	Psp	WP_039297911.1
1536771	000758605.1	Psp	WP_042231398.1
1536772	000758625.1	Psp	WP_039872522.1
1536772	000758625.1	Psp	WP_063837750.1
1536773	000758645.1	Psp	WP_042171804.1
1536773	000758645.1	Psp	WP_042175810.1
1536774	000758525.1	Psp	WP_038584320.1
1536774	000758525.1	Psp	WP_038590413.1
1536775	000758545.1	Psp	WP_042126789.1
1536775	000758545.1	Psp	WP_042183804.1
1547445	000764555.1	Fsp	WP_040010298.1
1548	000968375.1	Cscatologenes	WP_029161158.1
1548	000968375.1	Cscatologenes	WP_029162078.1

155864	000732965.1	Ecoli	WP_000839815.1
155864	000732965.1	Ecoli	WP_000978640.1
155864	000732965.1	Ecoli	WP_001020954.1
155864	000732965.1	Ecoli	WP_001292415.1
155864	000732965.1	Ecoli	WP_001295383.1
1560339	000801755.2	Esp	WP_039260581.1
1560339	000801755.2	Esp	WP_039261294.1
1560339	000801755.2	Esp	WP_039262954.1
1565605	000934545.1	Rbacterium	AJP48120.1
1565991	000747345.1	Bsp	WP_038538230.1
1565991	000747345.1	Bsp	WP_038540692.1
1566358	000949425.1	Psp	WP_044876209.1
1566358	000949425.1	Psp	WP_044880364.1
1570330	000827045.1	Bsp	WP_014416716.1
1570330	000827045.1	Bsp	WP_040221709.1
1574141	000877815.1	Bsp	WP_003226777.1
1574141	000877815.1	Bsp	WP_014479695.1
157783	000759535.1	Pcremoricolor	WP_049870512.1
1578828	000800725.2	Esp	AKM46197.1
1578828	000800725.2	Esp	AKM46835.1
1578828	000800725.2	Esp	AKM46837.1
1578828	000800725.2	Esp	AKM48692.1
1579	000934625.1	Lacidophilus	WP_003547166.1
1579	001469775.1	Ldelbrueckii	WP_003621775.1
1581557	000981505.1	Cmethylopumil	WP_046487832.1
1581680	000953015.1	Cmethylopumil	WP_045750867.1
1587	000961015.1	Lhelveticus	WP_052541562.1
1587	001006025.1	Lhelveticus	WP_046814123.1
1587	001006025.1	Lhelveticus	WP_046814465.1
1587	001308285.1	Lhelveticus	WP_054607391.1
1587	001308285.1	Lhelveticus	WP_054607569.1
158822	000757825.1	Cneteri	WP_038474637.1
158822	000757825.1	Cneteri	WP_038475417.1
158822	000757825.1	Cneteri	WP_038478940.1
158822	000758305.1	Cneteri	WP_039287677.1
158822	000758305.1	Cneteri	WP_039290441.1
158822	000758325.1	Cneteri	WP_039297941.1
158822	000758325.1	Cneteri	WP_039300555.1
158822	000758345.1	Cneteri	WP_038661636.1
158822	001506165.1	Cneteri	WP_039327238.1
158822	001506165.1	Cneteri	WP_039331194.1
158878	000009665.1	Saureus	WP_000812829.1
158879	000009645.1	Saureus	WP_000812829.1

159087	000012425.1	Daromatica	WP_011286344.1
1597	001191565.1	Lparacasei	WP_016377870.1
1597	001244395.1	Lparacasei	WP_003584633.1
1604	000191545.1	Lacidophilus	WP_013641833.1
160488	000007565.1	Pputida	X1
160799	000758665.1	Pborealis	WP_042209898.1
160799	000758665.1	Pborealis	WP_042212797.1
1611770	000931465.1	Psp	WP_044462840.1
1616788	001421015.1	Pbovis	WP_060531710.1
1616788	001421015.1	Pbovis	WP_060535746.1
1620392	001010805.1	Ssp	WP_047011638.1
1620421	001011155.1	Hsp	WP_047030402.1
1620421	001011155.1	Hsp	WP_047030645.1
162209	001465255.1	Pnaphthalenov	WP_062407783.1
162209	001465255.1	Pnaphthalenov	WP_062410318.1
1624	000758365.1	Lsalivarius	WP_034982265.1
1628753	000978495.1	Bsp	WP_003244780.1
1628753	000978495.1	Bsp	WP_046380714.1
1629723	001028085.1	Gsp	WP_044746532.1
1629723	001028085.1	Gsp	WP_047818078.1
1638788	001264245.1	Mpanniformis	WP_052277229.1
1639	000382925.1	Lmonocytogene	YP_007605159.1
1639	000438585.1	Lmonocytogene	WP_003727667.1
1639	000438605.1	Lmonocytogene	WP_014602264.1
1639	000438625.1	Lmonocytogene	WP_009927970.1
1639	000438645.1	Lmonocytogene	WP_023548283.1
1639	000438665.1	Lmonocytogene	WP_010959076.1
1639	000438685.2	Lmonocytogene	WP_003727667.1
1639	000438705.2	Lmonocytogene	WP_003724994.1
1639	000438725.2	Lmonocytogene	WP_003724994.1
1639	000438745.2	Lmonocytogene	WP_003724994.1
1639	000600015.1	Lmonocytogene	WP_010959076.1
1639	000681515.1	Lmonocytogene	WP_003724994.1
1639	000746625.1	Lmonocytogene	WP_009930436.1
1639	000800335.1	Lmonocytogene	WP_010959076.1
1639	000950775.1	Lmonocytogene	WP_012681468.1
1639	001005925.1	Lmonocytogene	WP_046811270.1
1639	001005985.1	Lmonocytogene	WP_014602264.1
1639	001027065.1	Lmonocytogene	WP_047933464.1
1639	001027085.1	Lmonocytogene	WP_014601195.1
1639	001027125.1	Lmonocytogene	WP_047938222.1
1639	001027165.1	Lmonocytogene	WP_009927970.1
1639	001027205.1	Lmonocytogene	WP_014601195.1

1639	001027245.1	Lmonocytogene	WP_014601195.1
1639	001047715.1	Lmonocytogene	WP_048667153.1
1639	001188655.1	Lmonocytogene	WP_012582337.1
1639	001454845.1	Lmonocytogene	WP_010959076.1
1639	001454865.1	Lmonocytogene	WP_003728581.1
1639	001454885.1	Lmonocytogene	WP_003728581.1
1639	001454925.1	Lmonocytogene	WP_003727667.1
1639	001483405.1	Lmonocytogene	WP_026750065.1
1639	001483425.1	Lmonocytogene	WP_031665378.1
1639	001483445.1	Lmonocytogene	WP_009930436.1
1641812	000981785.1	Maeruginosa	WP_046662473.1
1647413	001277295.1	Asp	WP_053539417.1
164756	000014165.1	Msp	WP_011557657.1
164757	000016005.1	Msp	WP_011854294.1
1648923	000876525.1	Bparalichenif	WP_020449790.1
1648923	000876525.1	Bparalichenif	WP_020451250.1
1649184	001278095.1	Ssp	WP_053562202.1
1649877	001007005.1	Psp	WP_046854822.1
1650654	001186215.1	Esp	WP_034163233.1
1650654	001186215.1	Esp	WP_034164754.1
1650654	001186215.1	Esp	WP_034165623.1
1650654	001186215.1	Esp	WP_038631389.1
1650654	001186215.1	Esp	WP_049648767.1
1652545	001454985.1	Asp	WP_047120015.1
1658672	001262075.1	Osp	WP_050716547.1
1660083	001186405.1	Lsp	WP_014960850.1
1662285	001183865.1	Msp	WP_049638317.1
166314	000161795.2	Ssp	WP_045172713.1
167539	000007925.1	Pmarinus	NP_875504.1
167542	000015665.1	Pmarinus	WP_011820481.1
167546	000015965.1	Pmarinus	WP_011863142.1
167555	000015685.1	Pmarinus	WP_011824078.1
1678028	001191005.1	Msp	WP_050408085.1
1678128	001412535.1	Lsp	WP_062404607.1
1678129	001412575.1	Lsp	WP_055360731.1
1678678	001293045.1	Bsp	WP_006048516.1
1679721	001298655.2	Hsp	WP_058258218.1
1695218	001465275.1	Psp	WP_062489700.1
1695218	001465275.1	Psp	WP_062492451.1
1696072	001266755.1	Msp	WP_053096374.1
169760	000758685.1	Pstellifer	WP_038692819.1
169760	000758685.1	Pstellifer	WP_038695464.1
170187	000006885.1	Spneumoniae	WP_000661000.1

1707785	001412595.1	Msp	WP_047825934.1
171101	000007045.1	Spneumoniae	NP_358410.1
1715259	001461805.1	Ksp	WP_013365210.1
1715259	001461805.1	Ksp	WP_013367100.1
1715259	001461805.1	Ksp	WP_062740052.1
1715259	001461805.1	Ksp	WP_062740402.1
1715259	001461805.1	Ksp	WP_062741960.1
1725411	001484565.1	Ssp	WP_058921186.1
1725411	001484565.1	Ssp	WP_058927332.1
1727164	001412655.1	Psp	WP_062547163.1
1736	001481725.1	Elimosum	WP_058696285.1
1751294	001484705.1	Ssp	WP_058915517.1
176279	000011925.1	Sepidermidis	WP_001832221.1
176280	000007645.1	Sepidermidis	NP_765857.1
1765	000758245.1	Mbovis	WP_003899363.1
1765	001078615.1	Mbovis	WP_003899363.1
1765	001483905.1	Mbovis	WP_003899363.1
1768242	001477625.1	Psp	WP_058719753.1
1768242	001477625.1	Psp	WP_058720126.1
1768242	001477625.1	Psp	WP_058720128.1
1770	000835225.1	Mavium	WP_003872520.1
1770	000835225.1	Mavium	WP_003872624.1
1772	000767665.1	Msmegmatis	WP_011726793.1
1772	000767705.1	Msmegmatis	WP_011726793.1
1772	001457595.1	Msmegmatis	WP_058124982.1
1773	000706665.1	Mtuberculosis	WP_003899363.1
1773	000738445.1	Mtuberculosis	WP_003899363.1
1773	000738475.1	Mtuberculosis	WP_003899363.1
1773	000756525.1	Mtuberculosis	WP_003899363.1
1773	000756545.1	Mtuberculosis	WP_003903932.1
1773	000954155.1	Mtuberculosis	WP_003899363.1
1773	001275565.1	Mtuberculosis	WP_003899363.1
177416	000008985.1	Ftularensis	YP_169451.1
179408	000317475.1	Onigroviridis	WP_015174541.1
180850	001183785.1	Bthuringiensi	WP_000084897.1
180850	001183785.1	Bthuringiensi	WP_001075293.1
187410	000006645.1	Ypestis	WP_002210002.1
187410	000006645.1	Ypestis	WP_002210805.1
189423	000147095.1	Spneumoniae	WP_000661011.1
189425	000758705.1	Pgraminis	WP_025708247.1
189425	000758705.1	Pgraminis	WP_042265802.1
189426	000758725.1	Podorifer	WP_036686120.1
189426	000758725.1	Podorifer	WP_038567999.1

189918	000015405.1	Msp	WP_011557657.1
190304	000007325.1	Fnucleatum	NP_603398.1
1907	000761215.1	Sglaucescens	WP_043497752.1
1907	000761215.1	Sglaucescens	WP_043497961.1
191292	000982715.1	Raetherivoran	WP_006944120.1
192	000632475.2	Abrasilense	AIB15315.1
195102	000009685.1	Cperfringens	WP_003461164.1
195102	000009685.1	Cperfringens	WP_011010913.1
195103	000013285.1	Cperfringens	WP_003461164.1
195103	000013285.1	Cperfringens	WP_011591136.1
195253	000316685.1	Ssp	WP_015125655.1
196162	000015265.1	Nsp	WP_011756798.1
196600	000009745.1	Vvulnificus	WP_011079948.1
196600	000009745.1	Vvulnificus	WP_011151494.1
196620	000011265.1	Saureus	WP_000812834.1
197221	000011345.1	Telongatus	NP_682656.1
198094	000007845.1	Banthracis	NP_842596.1
198094	000007845.1	Banthracis	NP_846410.1
198107	001190925.1	Asp	WP_050416790.1
198214	000006925.2	Sflexneri	NP_706131.1
198214	000006925.2	Sflexneri	NP_708736.2
198214	000006925.2	Sflexneri	NP_709824.2
198215	000007405.1	Sflexneri	WP_000978635.1
198215	000007405.1	Sflexneri	WP_001020991.1
198215	000007405.1	Sflexneri	WP_005085661.1
198467	001187595.1	Agonensis	WP_019418064.1
198467	001187595.1	Agonensis	WP_035064022.1
198628	000147055.1	Ddadantii	WP_013316699.1
199310	000007445.1	Ecoli	WP_000671170.1
199310	000007445.1	Ecoli	WP_000839794.1
199310	000007445.1	Ecoli	WP_000978663.1
199310	000007445.1	Ecoli	WP_001021003.1
199310	000007445.1	Ecoli	WP_001296667.1
199310	000007445.1	Ecoli	WP_001524296.1
200450	001186335.1	Ptrivialis	WP_049708678.1
200991	001465795.1	Prifietoensis	WP_058382248.1
200991	001465795.1	Prifietoensis	WP_058383463.1
202751	000763515.1	Livanovii	WP_014093876.1
202752	000763475.1	Livanovii	WP_038409250.1
203119	000015865.1	Rthermocellum	WP_011838304.1
203119	000015865.1	Rthermocellum	WP_023062647.1
203122	000013665.1	Sdegradans	WP_041324778.1
203124	000014265.1	Terythraeum	WP_011610990.1

203124	000014265.1	Terythraeum	WP_011611060.1
204773	000026125.1	Harsenicoxyda	WP_011870570.1
205914	000011785.1	Hsomnus	WP_011609161.1
205914	000011785.1	Hsomnus	WP_011609719.1
205922	000012445.1	Pfluorescens	WP_011333617.1
208596	000195575.1	Csp	WP_013709927.1
208963	000014625.1	Paeruginosa	WP_003087961.1
208963	000014625.1	Paeruginosa	WP_016254137.1
208964	000006765.1	Paeruginosa	NP_250037.1
208964	000006765.1	Paeruginosa	NP_250509.1
209261	000007545.1	Senterica	WP_000978690.1
209261	000007545.1	Senterica	WP_001021059.1
209261	000007545.1	Senterica	WP_001100652.1
211586	000146165.2	Soneidensis	NP_715954.1
212717	000007625.1	Ctetani	WP_011098536.1
212717	000007625.1	Ctetani	WP_011100413.1
213554	000696485.1	Hcampaniensis	WP_038477757.1
214092	000009065.1	Ypestis	YP_002346006.1
214092	000009065.1	Ypestis	YP_002346237.1
215689	000165815.1	Esp	WP_012669239.1
215689	000165815.1	Esp	WP_041474237.1
216142	000761155.1	Prhizosphaera	WP_043189012.1
216591	000009485.1	Bcenocepacia	WP_006488824.1
216591	000009485.1	Bcenocepacia	WP_006488848.1
216591	000009485.1	Bcenocepacia	WP_006491304.1
216592	000027125.1	Ecoli	WP_000978620.1
216592	000027125.1	Ecoli	WP_001020966.1
216592	000027125.1	Ecoli	WP_001295383.1
216592	000027125.1	Ecoli	WP_014639294.1
216594	000018345.1	Mmarinum	WP_012393949.1
216595	000009225.1	Pfluorescens	CAY48880.1
216597	000210855.2	Senterica	WP_000839739.1
216597	000210855.2	Senterica	WP_000978690.1
216597	000210855.2	Senterica	WP_001021054.1
216597	000210855.2	Senterica	WP_001100652.1
216597	000210855.2	Senterica	WP_001292402.1
216599	000283715.1	Ssonnei	WP_000134206.1
216599	000283715.1	Ssonnei	WP_000424603.1
216599	000283715.1	Ssonnei	WP_000978652.1
216599	000283715.1	Ssonnei	WP_001021017.1
216599	000283715.1	Ssonnei	WP_005141237.1
216599	000283715.1	Ssonnei	WP_014334061.1
216895	000039765.1	Vvulnificus	WP_011079948.1

216895	000039765.1	Vvulnificus	WP_011082127.1
218491	000011605.1	Patrosepticum	WP_011092567.1
218493	000252995.1	Sbongori	WP_000839714.1
218493	000252995.1	Sbongori	WP_000978697.1
218493	000252995.1	Sbongori	WP_001020947.1
218493	000252995.1	Sbongori	WP_001100654.1
218493	000252995.1	Sbongori	WP_001292414.1
218495	000009545.1	Suberis	WP_015911647.1
219334	000829055.1	Lcasei	WP_039639106.1
220341	000195995.1	Senterica	NP_454842.1
220341	000195995.1	Senterica	NP_457089.1
220341	000195995.1	Senterica	NP_458595.1
220664	000012265.1	Pprotegens	WP_011061575.1
221109	000011245.1	Oiheyensis	WP_011064437.1
222523	000008005.1	Bcereus	WP_000084887.1
222523	000008005.1	Bcereus	WP_001075281.1
223926	000196095.1	Vparahaemolyt	NP_799269.1
223926	000196095.1	Vparahaemolyt	NP_801145.1
224308	000009045.1	Bsubtilis	NP_387908.1
224308	000009045.1	Bsubtilis	NP_389346.1
224911	000011365.1	Bdiazoefficie	NP_769817.1
224911	000011365.1	Bdiazoefficie	NP_770114.1
226900	000007825.1	Bcereus	NP_829932.1
226900	000007825.1	Bcereus	NP_833681.1
228400	000019405.1	Hsomnus	WP_012341278.1
229193	000007885.1	Ypestis	WP_002210002.1
229193	000007885.1	Ypestis	WP_002210805.1
231023	000264665.1	Pputida	WP_029611831.1
2325	000763575.1	Tkivui	WP_049684307.1
2325	000763575.1	Tkivui	WP_049685049.1
232721	000015545.1	Asp	WP_011805918.1
2342	000517405.1	Csodalis	WP_025246423.1
2342	000517405.1	Csodalis	WP_025246771.1
234831	000184065.1	Psp	WP_013465293.1
235909	000009785.1	Gkaustophilus	WP_011229539.1
235909	000009785.1	Gkaustophilus	WP_011230565.1
237609	000746525.1	Palkylphenoli	WP_038613015.1
240292	000204075.1	Avariabilis	WP_011318944.1
243160	000011705.1	Bmallei	YP_102485.1
243243	000014985.1	Mavium	WP_009977695.1
243243	000014985.1	Mavium	WP_011724548.1
243277	000006745.1	Vcholerae	NP_229937.1
243277	000006745.1	Vcholerae	NP_233445.1

243365	000007705.1	Cviolaceum	WP_011137587.1
243365	000007705.1	Cviolaceum	WP_043595977.1
244366	000812205.1	Kvariicola	WP_008806477.1
244366	000812205.1	Kvariicola	WP_012968785.1
244366	000812205.1	Kvariicola	WP_012968903.1
244366	000828055.1	Kvariicola	WP_008806477.1
244366	000828055.1	Kvariicola	WP_022064710.1
244366	000828055.1	Kvariicola	WP_032691340.1
244366	001278905.1	Kvariicola	WP_002892486.1
244366	001278905.1	Kvariicola	WP_004204462.1
244366	001278905.1	Kvariicola	WP_017899763.1
246194	000012865.1	Chydrogenofor	WP_011342997.1
246195	000015345.1	Dnodosus	WP_011927774.1
246196	000015005.1	Msmegmatis	YP_884680.1
246432	001432245.1	Sequorum	WP_056935225.1
247156	000009805.1	Nfarcinica	WP_011207217.1
251221	000011385.1	Gviolaceus	NP_926433.1
251229	000317125.1	Cthermalis	WP_015156492.1
256701	001302565.1	Garilaitensis	WP_060702516.1
257314	000008065.1	Ljohnsonii	WP_011162617.1
260799	000008165.1	Banthracis	YP_026315.1
260799	000008165.1	Banthracis	YP_030124.1
261292	000219585.1	Nsp	X1
261591	000742895.1	Banthracis	WP_000084880.1
261591	000742895.1	Banthracis	WP_001075286.1
261594	000008445.1	Banthracis	WP_000084880.1
261594	000008445.1	Banthracis	WP_001075286.1
262316	000007865.1	Mavium	WP_003872520.1
262316	000007865.1	Mavium	WP_003872624.1
262543	000019905.1	Esibiricum	WP_012368935.1
262543	000019905.1	Esibiricum	WP_012370876.1
262728	000165525.1	Hinfluenzae	WP_005651071.1
263819	001047675.1	Yaleksiciae	WP_048616444.1
263819	001047675.1	Yaleksiciae	WP_048617490.1
263819	001047675.1	Yaleksiciae	WP_048619059.1
264198	000203875.1	Reutropha	WP_011296876.1
264445	001447075.1	Shygroscopicu	WP_058083286.1
264732	000013105.1	Mthermoacetic	YP_428923.1
264732	000013105.1	Mthermoacetic	YP_431095.1
265072	000013705.1	Mflagellatus	WP_011479280.1
265669	000008285.1	Lmonocytogene	WP_010959076.1
266264	000196015.1	Cmetalliduran	WP_008646295.1
266265	000013645.1	Pxenovorans	WP_011487262.1

266265	000013645.1	Pxenovorans	WP_011493889.1
266834	000006965.1	Smeliloti	NP_435606.1
266834	000006965.1	Smeliloti	NP_435607.4
266940	000017305.1	Kradiotoleran	WP_012085527.1
267608	000009125.1	Rsolanacearum	WP_011002288.1
269084	000010065.1	Selongatus	WP_011243135.1
269482	000016205.1	Bvietnamiensi	WP_011882700.1
269482	000016205.1	Bvietnamiensi	WP_011885706.1
269801	000832805.1	Bcereus	WP_001980832.1
269801	000832805.1	Bcereus	WP_033692457.1
271848	000012365.1	Bthailandensi	WP_009892476.1
272123	000317695.1	Acylindrica	WP_015212884.1
272123	000317695.1	Acylindrica	WP_015217169.1
272558	000011145.1	Bhalodurans	WP_010896225.1
272558	000011145.1	Bhalodurans	WP_010898791.1
272560	000011545.1	Bpseudomallei	YP_107631.1
272562	000008765.1	Cacetobutylic	NP_346938.1
272562	000008765.1	Cacetobutylic	NP_348954.1
272563	000009205.1	Pdifficile	YP_001087362.1
272563	000009205.1	Pdifficile	YP_001090072.1
272564	000021925.1	Dhafniense	WP_005815018.1
272564	000021925.1	Dhafniense	WP_015942617.1
272567	001274575.1	Gstearothermo	WP_011230565.1
272567	001274575.1	Gstearothermo	WP_012820430.1
272620	000016305.1	Kpneumoniae	WP_002889384.1
272620	000016305.1	Kpneumoniae	WP_002892486.1
272620	000016305.1	Kpneumoniae	WP_004151766.1
272621	000011985.1	Lacidophilus	YP_193877.1
272630	000022685.1	Mextorquens	WP_003605432.1
272843	000006825.1	Pmultocida	WP_005716647.1
273036	000009005.1	Saureus	WP_000812845.1
273068	000007085.1	Csubterraneus	WP_011024604.1
273068	000007085.1	Csubterraneus	WP_011025395.1
273123	000047365.1	Ypseudotuberc	WP_011191987.1
273123	000047365.1	Ypseudotuberc	WP_011192986.1
273526	000513215.1	Smarcescens	WP_025304020.1
273526	000513215.1	Smarcescens	WP_025304026.1
273526	000513215.1	Smarcescens	WP_025304259.1
273526	000513215.1	Smarcescens	WP_025305086.1
278992	001267885.1	Sambofaciens	WP_053126122.1
278992	001267885.1	Sambofaciens	WP_053126985.1
279010	000008425.1	Blicheniformi	WP_009328574.1
279010	000008425.1	Blicheniformi	WP_011197464.1

279058	000786695.1	Carenae	WP_038490817.1
279808	000009865.1	Shaemolyticus	WP_011276780.1
28031	000724775.3	Lfusiformis	WP_036122008.1
28031	000724775.3	Lfusiformis	WP_036123694.1
28037	001281025.1	Smitis	WP_060628327.1
28072	000316645.1	Nsp	WP_015141628.1
28095	000959725.1	Bgladioli	WP_013699054.1
28110	000833195.1	Fphilomiragia	WP_004288241.1
28110	000833215.1	Fphilomiragia	WP_004288241.1
28110	000833255.1	Fphilomiragia	WP_044525739.1
28110	000833295.1	Fphilomiragia	WP_042524534.1
28110	000833315.1	Fphilomiragia	WP_004288241.1
281309	000008505.1	Bthuringiensi	YP_034383.1
281309	000008505.1	Bthuringiensi	YP_038026.1
28141	000982825.1	Csakazakii	WP_007900483.1
28141	000982825.1	Csakazakii	WP_029038990.1
28141	000982825.1	Csakazakii	WP_029039292.1
28141	001277275.1	Csakazakii	WP_004385449.1
28141	001277275.1	Csakazakii	WP_004387170.1
28141	001277275.1	Csakazakii	WP_015386340.1
28150	001457675.1	Senterica	WP_000839730.1
28150	001457675.1	Senterica	WP_000978689.1
28150	001457675.1	Senterica	WP_001021045.1
28150	001457675.1	Senterica	WP_001100652.1
28150	001457675.1	Senterica	WP_023165931.1
28152	000834865.1	Ykristensenii	WP_038630974.1
28152	000834865.1	Ykristensenii	WP_038636767.1
282458	000011505.1	Saureus	WP_000812850.1
282459	000011525.1	Saureus	WP_000812834.1
283699	000310105.2	Psp	WP_033023881.1
28450	000755765.1	Bpseudomallei	WP_004191939.1
28450	000755825.1	Bpseudomallei	WP_004191939.1
28450	000756085.1	Bpseudomallei	WP_004191939.1
28450	000756125.1	Bpseudomallei	WP_004191939.1
28450	000756165.1	Bpseudomallei	WP_004191939.1
28450	000757015.2	Bpseudomallei	WP_004191939.1
28450	000953095.1	Bpseudomallei	WP_004191939.1
28450	000954175.1	Bpseudomallei	WP_004191939.1
28450	000959305.1	Bpseudomallei	WP_004191939.1
28450	001277875.1	Bpseudomallei	WP_004191939.1
28450	001277895.1	Bpseudomallei	WP_004191939.1
28450	001277975.1	Bpseudomallei	WP_004191939.1
28450	001318245.1	Bpseudomallei	WP_004191939.1

287	000816985.1	Paeruginosa	WP_003087961.1
287	000816985.1	Paeruginosa	WP_023131185.1
287	000829255.1	Paeruginosa	WP_003087961.1
287	000829255.1	Paeruginosa	WP_014603687.1
287	000829275.1	Paeruginosa	WP_003087961.1
287	000829275.1	Paeruginosa	WP_014603687.1
287	000829885.1	Paeruginosa	WP_003087961.1
287	000829885.1	Paeruginosa	WP_029771068.1
287	000981825.1	Paeruginosa	WP_003087961.1
287	000981825.1	Paeruginosa	WP_029771068.1
287	001077475.1	Paeruginosa	WP_003087961.1
287	001077475.1	Paeruginosa	WP_003159884.1
287	001291345.2	Paeruginosa	WP_003113592.1
287	001291345.2	Paeruginosa	WP_003159884.1
287	001293085.1	Paeruginosa	WP_003087961.1
287	001293085.1	Paeruginosa	WP_023086414.1
287	001447845.1	Paeruginosa	WP_003087961.1
287	001447845.1	Paeruginosa	WP_016561700.1
287	001457615.1	Paeruginosa	WP_003087961.1
287	001457615.1	Paeruginosa	WP_003159884.1
287	001465155.1	Paeruginosa	ALS10388.1
287	001465155.1	Paeruginosa	ALS10820.1
287	001482325.1	Paeruginosa	WP_003087961.1
287	001482325.1	Paeruginosa	WP_058149893.1
288000	000015165.1	Bsp	WP_012043265.1
288681	000011625.1	Bcereus	WP_000084884.1
288681	000011625.1	Bcereus	WP_001072957.1
289380	000013845.1	Cperfringens	WP_011591613.1
289380	000013845.1	Cperfringens	WP_045009338.1
290338	000018045.1	Ckoseri	WP_012133971.1
290338	000018045.1	Ckoseri	WP_012135080.1
290338	000018045.1	Ckoseri	WP_024130539.1
290339	000017665.1	Csakazakii	WP_012123774.1
290339	000017665.1	Csakazakii	WP_012125352.1
290339	000017665.1	Csakazakii	WP_012125704.1
290402	000016965.1	Cbeijerinckii	WP_012060311.1
290402	000016965.1	Cbeijerinckii	WP_012060567.1
291112	000196475.1	Pasymbiotica	WP_015833467.1
292	000755805.1	Bcepacia	WP_006497175.1
292	000755805.1	Bcepacia	WP_034202537.1
292	000755805.1	Bcepacia	WP_034202538.1
292	000974835.1	Bcepacia	WP_035971895.1
292415	000012745.1	Tdenitrifican	WP_011312618.1

292459	000009905.1	Sthermophilum	WP_011197372.1
292459	000009905.1	Sthermophilum	WP_011197381.1
292563	000317655.1	Cstanieri	WP_015222681.1
292564	000316515.1	Cgracile	WP_015107883.1
29338	000803665.1	Bthuringiensi	WP_000084904.1
29338	000803665.1	Bthuringiensi	WP_001075296.1
29343	000953215.1	Ccellulosi	WP_034836634.1
29343	000953215.1	Ccellulosi	WP_034837210.1
29343	000953215.1	Ccellulosi	WP_052659850.1
293826	000016985.1	Ametalliredig	WP_011971233.1
293826	000016985.1	Ametalliredig	WP_011971297.1
294	000730425.1	Pfluorescens	WP_038441535.1
294	000934565.1	Pfluorescens	WP_010210911.1
294	000968415.1	Pfluorescens	WP_046072321.1
294	001307155.1	Pfluorescens	WP_054597137.1
29410	001182765.1	Ssp	WP_049691943.1
29471	000696465.1	Patrosepticum	WP_039290475.1
29471	000740965.1	Patrosepticum	WP_039290475.1
29485	000834455.1	Yrohdei	WP_004713723.1
29485	000834455.1	Yrohdei	WP_004716005.1
29486	000834255.1	Yruckeri	WP_038240777.1
29486	000834255.1	Yruckeri	WP_042527160.1
29486	000834255.1	Yruckeri	WP_042528058.1
29486	000964565.1	Yruckeri	WP_004720425.1
29486	000964565.1	Yruckeri	WP_038240777.1
29486	000964565.1	Yruckeri	WP_045844523.1
295319	000011885.1	Senterica	WP_000839743.1
295319	000011885.1	Senterica	WP_000978696.1
295319	000011885.1	Senterica	WP_001021039.1
295319	000011885.1	Senterica	WP_001100647.1
295319	000011885.1	Senterica	WP_001292406.1
296591	000013865.1	Psp	WP_011483332.1
300267	000012005.1	Sdysenteriae	YP_404612.1
300267	000012005.1	Sdysenteriae	YP_405499.1
300268	000012025.1	Sboydii	WP_000040185.1
300268	000012025.1	Sboydii	WP_000424603.1
300268	000012025.1	Sboydii	WP_000978676.1
300269	000092525.1	Ssonnei	WP_000134207.1
300269	000092525.1	Ssonnei	WP_000424603.1
300269	000092525.1	Ssonnei	WP_000978652.1
300269	000092525.1	Ssonnei	WP_001021016.1
300269	000092525.1	Ssonnei	WP_005141237.1
300269	000092525.1	Ssonnei	WP_014334061.1

303	000691565.1	Pputida	WP_024087408.1
303	000691565.1	Pputida	WP_049870805.1
305	001267515.1	Rsolanacearum	AKZ25882.1
305	001299555.1	Rsolanacearum	WP_013205426.1
309798	000020945.1	Cproteolyticu	WP_012543982.1
309801	000021685.1	Troseum	WP_052294080.1
311402	000016285.1	Avitis	WP_015916838.1
312153	000016345.1	Pasymbioticus	WP_011902494.1
312309	000011805.1	Vfischeri	YP_205440.1
315730	000018825.1	Bweihenstepha	WP_002142966.1
315730	000018825.1	Bweihenstepha	WP_012260163.1
315749	000017425.1	Bcytotoxicus	WP_011983169.1
315749	000017425.1	Bcytotoxicus	WP_012095118.1
315750	000017885.2	Bpumilus	WP_012008638.1
315750	000017885.2	Bpumilus	WP_012009828.1
316	000590475.1	Pstutzeri	WP_025241981.1
316	000661915.1	Pstutzeri	WP_038659874.1
316	001038645.1	Pstutzeri	WP_013982902.1
316055	000014825.1	Rpalustris	WP_011664579.1
316275	000196495.1	Asalmonicida	WP_012550963.1
316275	000196495.1	Asalmonicida	WP_012552282.1
316278	000063525.1	Ssp	WP_011935199.1
316279	000012505.1	Ssp	WP_011360167.1
316385	000019425.1	Ecoli	WP_000040195.1
316385	000019425.1	Ecoli	WP_000978642.1
316385	000019425.1	Ecoli	WP_001020973.1
316385	000019425.1	Ecoli	WP_001295383.1
316385	000019425.1	Ecoli	WP_001326492.1
316401	000210475.1	Ecoli	WP_000040192.1
316401	000210475.1	Ecoli	WP_000978651.1
316401	000210475.1	Ecoli	WP_001021017.1
316401	000210475.1	Ecoli	WP_001295383.1
316401	000210475.1	Ecoli	WP_014640006.1
316407	000010245.1	Ecoli	BAA35349.1
316407	000010245.1	Ecoli	BAA77861.2
316407	000010245.1	Ecoli	BAE77028.1
316407	000010245.1	Ecoli	BAE78119.1
316407	000010245.1	Ecoli	BAE78134.1
316435	000714595.1	Ecoli	WP_000839794.1
316435	000714595.1	Ecoli	WP_000978663.1
316435	000714595.1	Ecoli	WP_001021003.1
316435	000714595.1	Ecoli	WP_001296667.1
316435	000714595.1	Ecoli	WP_001524296.1

317936	000316625.1	Nsp	WP_015113730.1
319224	000016585.1	Sputrefaciens	WP_011791051.1
319705	000758405.1	Mabscessus	WP_005111050.1
320372	000012785.1	Bpseudomallei	WP_004191939.1
320373	000015905.1	Bpseudomallei	WP_004191939.1
320388	000015465.1	Bmallei	WP_004191939.1
320389	000015625.1	Bmallei	WP_004191939.1
32049	000019485.1	Ssp	WP_012306781.1
32051	000063505.1	Ssp	WP_011933443.1
321314	000008105.1	Senterica	WP_001539058.1
321314	000008105.1	Senterica	WP_001539477.1
321314	000008105.1	Senterica	WP_001540636.1
321314	000008105.1	Senterica	WP_001540897.1
321314	000008105.1	Senterica	WP_001541324.1
321327	000013205.1	Ssp	WP_011429884.1
321332	000013225.1	Ssp	WP_011432136.1
321956	000014405.1	Ldelbrueckii	WP_003618812.1
321956	000014405.1	Ldelbrueckii	WP_003621775.1
321967	000014525.1	Lparacasei	YP_806869.1
322710	000021045.1	Avinelandii	WP_012701511.1
323848	000196355.1	Nmultiformis	WP_041352407.1
324057	000023585.1	Psp	WP_012772014.1
324057	000023585.1	Psp	WP_015845390.1
324831	000014425.1	Lgasseri	WP_011679015.1
325240	000015845.1	Sbaltica	WP_011848112.1
326297	000015245.1	Samazonensis	WP_011761441.1
326423	000015785.1	Bvelezensis	WP_011996166.1
326423	000015785.1	Bvelezensis	WP_012117476.1
326425	000422165.1	Lhelveticus	WP_020829009.1
326425	000422165.1	Lhelveticus	WP_020829153.1
326442	000026085.1	Phaloplanktis	WP_011327780.1
329726	000018105.1	Amarina	WP_012163092.1
330	000953455.1	Ppseudoalcali	WP_003461428.1
331111	000017745.1	Ecoli	WP_000839764.1
331111	000017745.1	Ecoli	WP_000978647.1
331111	000017745.1	Ecoli	WP_001020977.1
331111	000017745.1	Ecoli	WP_001295383.1
331111	000017745.1	Ecoli	WP_024184889.1
331112	000017765.1	Ecoli	WP_000839756.1
331112	000017765.1	Ecoli	WP_000978647.1
331112	000017765.1	Ecoli	WP_001020973.1
331112	000017765.1	Ecoli	WP_001295383.1
331271	000014085.1	Bcenocepacia	WP_011545955.1

331271	000014085.1	Bcenocepacia	WP_011548052.1
331271	000014085.1	Bcenocepacia	WP_041489406.1
331272	000203955.1	Bcenocepacia	WP_011545955.1
331272	000203955.1	Bcenocepacia	WP_011548052.1
331272	000203955.1	Bcenocepacia	WP_041489406.1
331978	000757035.2	Bpseudomallei	WP_004191939.1
334406	000283675.1	Bcereus	WP_000084879.1
334406	000283675.1	Bcereus	WP_001075283.1
334413	000010185.1	Fmagna	WP_012290950.1
335541	000014725.1	Swolfei	WP_041427230.1
335659	000284275.1	Bsp	WP_015686073.1
335659	000284275.1	Bsp	WP_015687291.1
336982	000016925.1	Mtuberculosis	WP_003899363.1
338187	000017705.1	Vcampbellii	WP_005429232.1
338187	000017705.1	Vcampbellii	WP_010444363.1
33892	001043255.1	Mbovis	WP_003899363.1
338969	000013605.1	Rferrireducen	WP_011464418.1
339670	000203915.1	Bambifaria	WP_011657650.1
339670	000203915.1	Bambifaria	WP_011659330.1
340099	000019085.1	Tpseudethanol	WP_003867357.1
340099	000019085.1	Tpseudethanol	WP_012269266.1
340184	000725265.1	Ecoli	WP_000839764.1
340184	000725265.1	Ecoli	WP_000978647.1
340184	000725265.1	Ecoli	WP_001020973.1
340184	000725265.1	Ecoli	WP_001295383.1
342108	000009985.1	Mmagneticum	WP_011383521.1
342113	000755985.1	Boklahomensis	WP_010102235.1
342451	000010125.1	Ssaprophyticu	WP_011303883.1
344609	000020185.1	Sboydii	WP_000424603.1
344609	000020185.1	Sboydii	WP_000978678.1
344609	000020185.1	Sboydii	WP_001020994.1
345073	000016245.1	Vcholerae	WP_001019828.1
345073	000016245.1	Vcholerae	WP_001086685.1
345219	000169195.2	Bcoagulans	WP_014095608.1
345219	000169195.2	Bcoagulans	WP_017550884.1
347495	000239195.1	Bcereus	WP_000084880.1
347495	000239195.1	Bcereus	WP_001073001.1
348824	000577275.1	Rsp	WP_024317700.1
349106	000016885.1	Psp	WP_011960413.1
349161	000016165.1	Dreducens	WP_011876452.1
349161	000016165.1	Dreducens	WP_011878152.1
349520	000146875.2	Ppolymyxa	ADM67917.1
349520	000146875.2	Ppolymyxa	ADM70795.1

349746	000018805.1	Ypestis	WP_002210002.1
349746	000018805.1	Ypestis	WP_002210805.1
349747	000016945.1	Ypseudotuberc	WP_011191987.1
349747	000016945.1	Ypseudotuberc	WP_012104654.1
350058	000015305.1	Mvanbaalenii	WP_011780130.1
350688	000018325.1	Aoremlandii	WP_012157944.1
350688	000018325.1	Aoremlandii	WP_012158260.1
350688	000018325.1	Aoremlandii	WP_041718985.1
350701	000959505.1	Bdolosa	WP_035971895.1
351581	000168775.2	Ftularensis	WP_004336874.1
351627	000016545.1	Csaccharolyti	WP_039765107.1
351627	000016545.1	Csaccharolyti	WP_041722736.1
351671	000968195.1	Xdoucetiae	WP_045970981.1
351745	000015185.1	Ssp	WP_011791051.1
351746	000016865.1	Pputida	WP_012051761.1
353496	000191165.1	Ldelbrueckii	WP_014564683.1
353496	000191165.1	Ldelbrueckii	WP_014565255.1
355278	000017605.1	Lbiflexa	WP_012387712.1
35623	000953195.1	Aoculi	WP_045748752.1
357348	000015925.1	Bpseudomallei	WP_004191939.1
357809	000018685.1	Lphytoferment	WP_012201910.1
357809	000018685.1	Lphytoferment	WP_041704596.1
358220	000302535.1	Asp	WP_015014683.1
358681	000010165.1	Bbrevis	WP_012683853.1
358681	000010165.1	Bbrevis	WP_015892152.1
358681	000010165.1	Bbrevis	WP_041749937.1
359786	000016805.1	Saureus	WP_000812829.1
359787	000017125.1	Saureus	WP_000812829.1
360102	000013825.1	Ypestis	WP_002210002.1
360102	000013825.1	Ypestis	WP_002210805.1
360118	000959145.1	Bpseudomallei	WP_004191939.1
360910	000070465.1	Bavium	WP_012416176.1
360910	000070465.1	Bavium	WP_012418330.1
360911	000023045.1	Esp	WP_012727751.1
360911	000023045.1	Esp	WP_015881264.1
361100	000013065.1	Bcereus	WP_000084882.1
361100	000013065.1	Bcereus	WP_001075266.1
362242	000013925.1	Mulcerans	X1
362257	000830005.1	Svietnamensis	WP_041130595.1
362257	000830005.1	Svietnamensis	WP_041132938.1
362257	000830005.1	Svietnamensis	WP_041133587.1
362663	000013305.1	Ecoli	WP_000839777.1
362663	000013305.1	Ecoli	WP_000978670.1

362663	000013305.1	Ecoli	WP_001020992.1
362663	000013305.1	Ecoli	WP_001092280.1
362663	000013305.1	Ecoli	WP_001362902.1
362663	000013305.1	Ecoli	WP_011579305.1
362948	000008925.1	Lsalivarius	YP_535038.1
364106	000013265.1	Ecoli	WP_000839776.1
364106	000013265.1	Ecoli	WP_000978670.1
364106	000013265.1	Ecoli	WP_001021000.1
364106	000013265.1	Ecoli	WP_001296667.1
364106	000013265.1	Ecoli	WP_001362902.1
365044	000015505.1	Pnaphthaleniv	WP_041376652.1
365046	000215705.1	Rtataouinensi	WP_013901274.1
366394	000017145.1	Smedicae	YP_001313905.1
366394	000017145.1	Smedicae	YP_001313906.1
367190	000582515.1	Ysimilis	WP_025381153.1
367190	000582515.1	Ysimilis	WP_025383150.1
36809	001430775.1	Mabscessus	WP_005111050.1
370438	000010565.1	Pthermopropio	WP_012033161.1
370438	000010565.1	Pthermopropio	WP_041533473.1
373153	000014365.1	Spneumoniae	WP_000661024.1
37329	001182745.2	Nfarcinica	CRY79285.1
373384	000013585.1	Sflexneri	WP_000978634.1
373384	000013585.1	Sflexneri	WP_001021029.1
373384	000013585.1	Sflexneri	WP_024260307.1
373994	000316665.1	Rsp	WP_015122491.1
374931	000016485.1	Hinfluenzae	WP_012055127.1
375	000807315.1	Bjaponicum	WP_014493537.1
375	000807315.1	Bjaponicum	WP_014496637.1
375286	000013625.1	Jsp	WP_012079048.1
376619	000009245.1	Ftularensis	WP_004336874.1
377628	000013805.1	Ypestis	WP_002210002.1
377628	000013805.1	Ypestis	WP_002210805.1
379731	000013785.1	Pstutzeri	WP_011913376.1
380703	000014805.1	Ahydrophila	YP_855733.1
381666	000009285.2	Reutropha	CAJ94006.1
381754	000017205.1	Paeruginosa	WP_012076162.1
381754	000017205.1	Paeruginosa	WP_012076537.1
382	000747295.1	Smeliloti	WP_014528432.1
382	000747295.1	Smeliloti	WP_040120805.1
382245	000196395.1	Asalmonicida	WP_005312101.1
38300	001278075.1	Spristinaespi	WP_005307429.1
384676	000026105.1	Pentomophila	WP_011534676.1
386043	000060285.1	Lwelshimeri	WP_011703337.1

386415	000014125.1	Cnovyi	WP_011723205.1
386585	000008865.1	Ecoli	NP_308215.1
386585	000008865.1	Ecoli	NP_308748.1
386585	000008865.1	Ecoli	NP_311868.2
386585	000008865.1	Ecoli	NP_313126.1
386585	000008865.1	Ecoli	NP_313140.1
386656	000016445.1	Ypestis	WP_002210002.1
388272	000168335.1	Paeruginosa	WP_003087961.1
388272	000168335.1	Paeruginosa	WP_023131530.1
390235	000019445.1	Pputida	WP_012315302.1
390333	000056065.1	Ldelbrueckii	WP_004560911.1
390333	000056065.1	Ldelbrueckii	WP_011543702.1
391008	000020665.1	Smaltophilia	WP_012511405.1
391038	000020045.1	Pphymatum	WP_012401567.1
391038	000020045.1	Pphymatum	WP_012405634.1
391038	000020045.1	Pphymatum	WP_012405636.1
391165	000014285.1	Gbethesdensis	WP_011631230.1
391735	000015565.1	Veiseniae	WP_011811210.1
393011	000014605.1	Ftularensis	WP_003018000.1
393115	000009325.1	Ftularensis	WP_003020112.1
393126	000168575.2	Lmonocytogene	WP_009930436.1
393127	000168595.2	Lmonocytogene	WP_014602264.1
393130	000168635.2	Lmonocytogene	WP_003722054.1
393133	000168695.2	Lmonocytogene	WP_014601195.1
393305	000009345.1	Yenterocoliti	YP_001004824.1
393305	000009345.1	Yenterocoliti	YP_001007441.1
393305	000009345.1	Yenterocoliti	YP_001007613.1
393305	000009345.1	Yenterocoliti	YP_001007683.1
394503	000022065.1	Ccellulolytic	WP_012634609.1
394503	000022065.1	Ccellulolytic	WP_012634870.1
395019	000018505.1	Bmultivorans	WP_006398616.1
395019	000018505.1	Bmultivorans	WP_012216569.1
395095	000153685.2	Mtuberculosis	WP_003899363.1
395494	000145255.1	Gcapsiferrifo	WP_013292400.1
395495	000019785.1	Lcholodnii	WP_012346914.1
395960	000020445.1	Rpalustris	WP_012495619.1
395961	000022045.1	Csp	WP_012630562.1
395962	000024045.1	Csp	WP_015784137.1
395963	000019845.1	Bindica	WP_012383934.1
395965	000021745.1	Msilvestris	WP_012589275.1
396513	000009405.1	Scarnosus	WP_012664150.1
397945	000015325.1	Acitrulli	WP_011796325.1
398511	000005825.2	Bpseudofirmus	WP_012957046.1

398511	000005825.2	Bpseudofirmus	WP_012959554.1
398527	000020125.1	Pphytofirmans	WP_012432069.1
398577	000019925.1	Bambifaria	WP_012364454.1
398577	000019925.1	Bambifaria	WP_012366595.1
398578	000018665.1	Dacidovorans	WP_012204812.1
399599	000018765.1	Sbaltica	WP_012197746.1
399726	000019065.1	Tsp	WP_003867357.1
399726	000019065.1	Tsp	WP_009052285.1
399739	000016565.1	Pmendocina	WP_012018493.1
399741	000018085.1	Sproteamacula	WP_012146472.1
399741	000018085.1	Sproteamacula	WP_012146478.1
399741	000018085.1	Sproteamacula	WP_012146749.1
399741	000018085.1	Sproteamacula	WP_012147495.1
399741	000018085.1	Sproteamacula	WP_041418450.1
399742	000016325.1	Esp	WP_012016132.1
399742	000016325.1	Esp	WP_015960370.1
399742	000016325.1	Esp	WP_041689627.1
399804	000169215.2	Sputrefaciens	WP_011791051.1
401614	000014645.1	Ftularensis	WP_003035692.1
401614	000014645.1	Ftularensis	WP_003035692.1
402626	000020205.1	Rpickettii	WP_004632157.1
402882	000017325.1	Sbaltica	WP_012090462.1
40324	001274595.1	Smaltophilia	WP_053450069.1
40324	001274655.1	Smaltophilia	WP_043402035.1
40324	001274675.1	Smaltophilia	WP_043402035.1
403957	000725285.1	Vsp	WP_040953874.1
403957	000725285.1	Vsp	WP_040957096.1
405531	000021305.1	Bcereus	WP_000084903.1
405531	000021305.1	Bcereus	WP_001073003.1
405532	000021205.1	Bcereus	WP_000084899.1
405532	000021205.1	Bcereus	WP_001075299.1
405534	000021225.1	Bcereus	WP_000084879.1
405534	000021225.1	Bcereus	WP_001075283.1
405535	000021785.1	Bcereus	WP_000084880.1
405535	000021785.1	Bcereus	WP_001075274.1
405566	000015385.1	Lhelveticus	WP_012211840.1
405566	000015385.1	Lhelveticus	WP_012212232.1
405566	000015385.1	Lhelveticus	WP_041810707.1
40576	000973125.1	Xbovienii	WP_046336771.1
405955	000014845.1	Ecoli	WP_000671170.1
405955	000014845.1	Ecoli	WP_000839776.1
405955	000014845.1	Ecoli	WP_000978670.1
405955	000014845.1	Ecoli	WP_001021000.1

405955	000014845.1	Ecoli	WP_001296667.1
405955	000014845.1	Ecoli	WP_001362902.1
406425	000019505.1	Bcenocepacia	WP_011545955.1
406425	000019505.1	Bcenocepacia	WP_012338448.1
406425	000019505.1	Bcenocepacia	WP_050780502.1
406818	000027225.1	Xbovienii	WP_012988689.1
407035	001005905.1	Shalodurans	WP_046791345.1
407976	000021665.1	Sbaltica	WP_012197746.1
409438	000010385.1	Ecoli	WP_000839754.1
409438	000010385.1	Ecoli	WP_000978647.1
409438	000010385.1	Ecoli	WP_001020973.1
409438	000010385.1	Ecoli	WP_001295383.1
409438	000010385.1	Ecoli	WP_001297242.1
410289	000009445.1	Mbovis	WP_003899363.1
411154	000060345.1	Gforsetii	WP_011710038.1
412022	000015605.1	Bmallei	WP_004191939.1
412694	000015065.1	Bthuringiensi	WP_000084880.1
412694	000015065.1	Bthuringiensi	WP_001075278.1
412694	000832885.1	Bthuringiensi	WP_018780882.1
412694	000832885.1	Bthuringiensi	WP_018783057.1
413882	001017435.1	Pbrachysporum	WP_047195262.1
413882	001017435.1	Pbrachysporum	WP_053013806.1
413996	000967285.1	Mbovis	WP_003899363.1
413997	000017985.1	Ecoli	WP_000040191.1
413997	000017985.1	Ecoli	WP_000839790.1
413997	000017985.1	Ecoli	WP_000978645.1
413997	000017985.1	Ecoli	WP_001020950.1
413997	000017985.1	Ecoli	WP_001295383.1
413999	000063585.1	Cbotulinum	YP_001252602.1
413999	000063585.1	Cbotulinum	YP_001255608.1
41431	000021805.1	Csp	WP_012595621.1
41514	000018625.1	Senterica	WP_000137596.1
41514	000018625.1	Senterica	WP_000839744.1
41514	000018625.1	Senterica	WP_000978611.1
41514	000018625.1	Senterica	WP_001021063.1
41514	000018625.1	Senterica	WP_001100644.1
416344	000262695.1	Bsp	WP_011885706.1
416344	000262695.1	Bsp	WP_014725479.1
418127	000010445.1	Saureus	WP_000812829.1
418136	000016105.1	Ftularensis	WP_003020112.1
41899	001411805.1	Bplantarii	WP_042625743.1
41899	001411805.1	Bplantarii	WP_055138169.1
419610	000018845.1	Mextorquens	WP_012252687.1

419947	000016145.1	Mtuberculosis	WP_003899363.1
420246	000015745.1	Gthermodenitr	WP_008878744.1
420246	000015745.1	Gthermodenitr	WP_011886599.1
420662	000015725.1	Mpetroleiphil	WP_011829520.1
423368	000016045.1	Senterica	WP_000839726.1
423368	000016045.1	Senterica	WP_000978691.1
423368	000016045.1	Senterica	WP_001021052.1
423368	000016045.1	Senterica	WP_001100652.1
423368	000016045.1	Senterica	WP_001292401.1
426117	000019365.1	Msp	WP_012331497.1
426355	000019725.1	Mradiotoleran	WP_012321324.1
426430	000010465.1	Saureus	WP_000812832.1
428406	000023425.1	Rpickettii	WP_012762551.1
429009	000024605.1	Adegensii	WP_049757179.1
431943	000016505.1	Ckluyveri	WP_012103526.1
431943	000016505.1	Ckluyveri	WP_012104115.1
431946	000010485.1	Ecoli	WP_000978630.1
431946	000010485.1	Ecoli	WP_001021005.1
431946	000010485.1	Ecoli	WP_001296667.1
431946	000010485.1	Ecoli	WP_001531487.1
431946	000010485.1	Ecoli	WP_012896845.1
435998	000336425.1	Smarcescens	WP_004931982.1
435998	000336425.1	Smarcescens	WP_015376427.1
435998	000336425.1	Smarcescens	WP_015378888.1
435998	000336425.1	Smarcescens	WP_041922371.1
438753	000010525.1	Acaulinodans	WP_012171543.1
438753	000010525.1	Acaulinodans	WP_043878919.1
439184	000800845.2	Ecoli	AJM72412.1
439184	000800845.2	Ecoli	AJM72813.1
439184	000800845.2	Ecoli	AJM75161.1
439184	000800845.2	Ecoli	AJM76388.1
439184	000800845.2	Ecoli	AJM76402.1
439292	000093085.1	Bselenitiredu	WP_013171014.1
439292	000093085.1	Bselenitiredu	WP_013172544.1
439843	000020745.1	Senterica	WP_000839742.1
439843	000020745.1	Senterica	WP_000978689.1
439843	000020745.1	Senterica	WP_000992185.1
439843	000020745.1	Senterica	WP_001021041.1
439843	000020745.1	Senterica	WP_001100652.1
439843	000020745.1	Senterica	WP_001292402.1
439851	000020925.1	Senterica	WP_000839715.1
439851	000020925.1	Senterica	WP_000978691.1
439851	000020925.1	Senterica	WP_001021060.1

439851	000020925.1	Senterica	WP_001100658.1
439851	000020925.1	Senterica	WP_001292402.1
439855	000019645.1	Ecoli	WP_000040183.1
439855	000019645.1	Ecoli	WP_000978629.1
439855	000019645.1	Ecoli	WP_001021006.1
439855	000019645.1	Ecoli	WP_001295383.1
439855	000019645.1	Ecoli	WP_001376254.1
43989	000017845.1	Csp	WP_009543527.1
440085	000021845.1	Mextorquens	WP_003605432.1
441156	000959265.1	Bpseudomallei	WP_004191939.1
441162	000959365.1	Boklahomensis	WP_010102235.1
441620	000019945.1	Mpopuli	WP_012452867.1
441768	000018785.1	Alaidlawii	WP_012243243.1
441770	000017025.1	Cbotulinum	WP_011947924.1
441770	000017025.1	Cbotulinum	WP_012048125.1
441771	000017045.1	Cbotulinum	YP_001386012.1
441771	000017045.1	Cbotulinum	YP_001388847.1
441772	000017065.1	Cbotulinum	WP_011987181.1
441772	000017065.1	Cbotulinum	WP_012100824.1
441952	000018925.1	Ftularensis	X1
44251	000756615.1	Pdurus	WP_042204548.1
44251	000756615.1	Pdurus	WP_042206467.1
443149	000270345.1	Mtuberculosis	WP_003899363.1
443150	000270365.1	Mtuberculosis	WP_003899363.1
443218	000214175.1	Asubflavus	X1
444103	001011035.1	Ssp	WP_047019439.1
444177	000017965.1	Lsphaericus	WP_012291859.1
444177	000017965.1	Lsphaericus	WP_031418880.1
444450	000021125.1	Ecoli	WP_000839815.1
444450	000021125.1	Ecoli	WP_000978640.1
444450	000021125.1	Ecoli	WP_001020954.1
444450	000021125.1	Ecoli	WP_001292415.1
444450	000021125.1	Ecoli	WP_001295383.1
44454	000770235.1	Mavium	WP_009977695.1
44454	000770235.1	Mavium	WP_011724548.1
44574	001007935.1	Ncommunis	WP_046849187.1
445932	000020145.1	Eminutum	WP_012415177.1
446462	000023245.1	Amirum	WP_049796913.1
449447	000010625.1	Maeruginosa	WP_012264848.1
451515	000013465.1	Saureus	WP_000812827.1
451516	000017085.1	Saureus	WP_000812827.1
451709	000832865.1	Bcereus	WP_000084880.1
451709	000832865.1	Bcereus	WP_001075278.1

452638	000019745.1	Pnecessarius	WP_012358112.1
452652	000269985.1	Ksetae	WP_014133744.1
452652	000269985.1	Ksetae	WP_033260383.1
454166	000020885.1	Senterica	WP_000839737.1
454166	000020885.1	Senterica	WP_000978690.1
454166	000020885.1	Senterica	WP_000992186.1
454166	000020885.1	Senterica	WP_001021045.1
454166	000020885.1	Senterica	WP_001100652.1
454166	000020885.1	Senterica	WP_001292402.1
454169	000020705.1	Senterica	WP_000839725.1
454169	000020705.1	Senterica	WP_000978689.1
454169	000020705.1	Senterica	WP_001021052.1
454169	000020705.1	Senterica	WP_001100652.1
454169	000020705.1	Senterica	WP_001292402.1
456481	000017685.1	Lbiflexa	WP_012387712.1
457405	000158275.2	Fnucleatum	WP_008701157.1
457425	000359525.1	Salbus	WP_003946882.1
457428	000739105.1	Slividans	WP_003971830.1
457570	000020005.1	Nthermophilus	WP_012446521.1
458233	000010585.1	Mcaseolyticus	WP_015912160.1
458233	000010585.1	Mcaseolyticus	WP_050742459.1
458234	000017785.1	Ftularensis	WP_003018000.1
459424	000026445.2	Mliflandii	WP_015355832.1
460265	000022085.1	Mnodulans	WP_015927042.1
46170	000695215.1	Saureus	WP_000812832.1
46234	000312705.1	Asp	WP_015083257.1
465817	000026185.1	Etasmaniensis	WP_012440655.1
465817	000026185.1	Etasmaniensis	WP_012442553.1
469008	000009565.2	Ecoli	CAQ30700.1
469008	000009565.2	Ecoli	CAQ31158.1
469008	000009565.2	Ecoli	CAQ33275.1
469008	000009565.2	Ecoli	CAQ34466.2
469008	000009565.2	Ecoli	CAQ34481.1
469383	000025265.1	Cwoesei	WP_012933767.1
469383	000025265.1	Cwoesei	WP_012934275.1
469383	000025265.1	Cwoesei	WP_012936059.1
469602	000163915.2	Fnucleatum	WP_005889192.1
469604	000162235.2	Fnucleatum	WP_008799184.1
469607	000400875.1	Fnucleatum	WP_008798408.1
471223	000023385.1	Gsp	WP_012748804.1
471223	000023385.1	Gsp	WP_015863303.1
471821	000146025.1	utermite	BAG13995.1
476213	000018385.1	Senterica	WP_000839723.1

476213	000018385.1	Senterica	WP_001021042.1
476213	000018385.1	Senterica	WP_001100652.1
476213	000018385.1	Senterica	WP_001292405.1
477245	000931445.1	Scyaneogriseu	WP_044378571.1
477245	000931445.1	Scyaneogriseu	WP_044386696.1
477974	000018425.1	Cdesulforudis	WP_049752516.1
478433	000154585.2	Mtuberculosis	WP_003899363.1
478434	000023625.1	Mtuberculosis	WP_003899363.1
478435	000154605.2	Mtuberculosis	WP_003899363.1
47917	001006005.1	Sfonticola	WP_024483249.1
47917	001006005.1	Sfonticola	WP_024483388.1
47917	001006005.1	Sfonticola	WP_024483781.1
47917	001006005.1	Sfonticola	WP_024484679.1
47917	001006005.1	Sfonticola	WP_024485570.1
47917	001006005.1	Sfonticola	WP_024485574.1
479431	000024365.1	Nmultipartita	X1
479436	000024945.1	Vparvula	WP_012864127.1
481743	000024685.1	Psp	WP_012818300.1
481743	000024685.1	Psp	WP_036665945.1
481805	000019385.1	Ecoli	WP_000040192.1
481805	000019385.1	Ecoli	WP_000978624.1
481805	000019385.1	Ecoli	WP_001020950.1
481805	000019385.1	Ecoli	WP_001295383.1
481805	000019385.1	Ecoli	WP_001326492.1
482957	000012945.1	Blata	WP_011352882.1
482957	000012945.1	Blata	WP_011354387.1
482957	000012945.1	Blata	WP_011355669.1
482957	000012945.1	Blata	WP_011355670.1
484021	000009885.1	Kpneumoniae	WP_002889384.1
484021	000009885.1	Kpneumoniae	WP_002892486.1
484021	000009885.1	Kpneumoniae	WP_014906933.1
484022	000019285.1	Fphilomiragia	WP_004288241.1
484770	000725345.1	Psp	WP_051789134.1
485916	000024205.1	Dacetoxidans	WP_012813514.1
485916	000024205.1	Dacetoxidans	WP_015759344.1
487213	000019025.1	Spneumoniae	WP_000661006.1
487214	000019265.1	Spneumoniae	WP_000661011.1
487521	000277125.1	Mintracellula	WP_014379805.1
487521	000277125.1	Mintracellula	WP_014380494.1
488142	000747565.1	Ssp	WP_033635725.1
488142	000747565.1	Ssp	WP_033644924.1
488142	000747565.1	Ssp	WP_033651471.1
488142	000747565.1	Ssp	WP_042785262.1

488221	000018965.1	Spneumoniae	WP_000661021.1
488222	000018985.1	Spneumoniae	WP_000661009.1
488223	000019005.1	Spneumoniae	WP_000665647.1
488447	001029145.1	Bcontaminans	WP_011354387.1
488447	001029145.1	Bcontaminans	WP_046544643.1
488447	001029145.1	Bcontaminans	WP_046549860.1
488447	001029145.1	Bcontaminans	WP_047849613.1
488447	001029145.1	Bcontaminans	WP_047850241.1
491915	000019045.1	Aflavithermus	WP_012573793.1
491915	000019045.1	Aflavithermus	WP_012575440.1
491916	000020265.1	Retli	WP_012482182.1
491916	000020265.1	Retli	WP_012482183.1
492670	000769555.1	Bvelezensis	WP_017417709.1
492670	000769555.1	Bvelezensis	WP_022552467.1
492670	000987825.1	Bvelezensis	WP_046559288.1
492670	000987825.1	Bvelezensis	WP_046559566.1
492670	000988345.1	Bvelezensis	WP_015239787.1
492670	000988345.1	Bvelezensis	WP_038463551.1
497965	000147335.1	Csp	WP_013320263.1
498211	000019225.1	Cjaponicus	WP_012489216.1
498213	000019305.1	Cbotulinum	WP_003403338.1
498213	000019305.1	Cbotulinum	WP_003404847.1
498214	000019545.1	Cbotulinum	WP_012342676.1
498214	000019545.1	Cbotulinum	WP_012343443.1
498216	000019245.3	Lcasei	WP_003564627.1
498217	000020865.1	Etarda	WP_012847161.1
498217	000020865.1	Etarda	WP_012847625.1
498217	000020865.1	Etarda	WP_012849827.1
498761	000019165.1	Hmodesticaldu	WP_012282317.1
502800	000019465.1	Ypseudotuberc	WP_012104654.1
502800	000019465.1	Ypseudotuberc	WP_012304347.1
502801	000020085.1	Ypseudotuberc	WP_011191987.1
502801	000020085.1	Ypseudotuberc	WP_012413942.1
507522	000019565.1	Kpneumoniae	WP_004204462.1
507522	000019565.1	Kpneumoniae	WP_008806477.1
507522	000019565.1	Kpneumoniae	WP_012542564.1
508767	000020285.1	Cbotulinum	WP_003373549.1
508767	000020285.1	Cbotulinum	WP_012451323.1
509193	000175295.2	Tbrockii	WP_003867357.1
509193	000175295.2	Tbrockii	WP_012269266.1
510831	000023305.1	Ftularensis	WP_003020112.1
511	000967305.2	Afaecalis	WP_042480898.1
511	000967305.2	Afaecalis	WP_045930821.1

511051	000284335.1	Cexile	WP_014453687.1
511062	000243075.1	Osp	WP_014293166.1
511145	000005845.2	Ecoli	NP_414728.1
511145	000005845.2	Ecoli	NP_415220.1
511145	000005845.2	Ecoli	NP_417440.4
511145	000005845.2	Ecoli	NP_418541.2
511145	000005845.2	Ecoli	NP_418555.1
512565	000284295.1	Amissouriensi	WP_014443451.1
512566	000019825.1	Spneumoniae	WP_000661013.1
515619	000020605.1	Erectale	WP_012744031.1
515619	000020605.1	Erectale	WP_012744096.1
515620	000146185.1	Eeligens	WP_012738298.1
515620	000146185.1	Eeligens	WP_012739654.1
515621	000020345.1	Cbotulinum	WP_003360141.1
515621	000020345.1	Cbotulinum	WP_003363053.1
515622	000145035.1	Bproteoclasti	WP_013279492.1
515622	000145035.1	Bproteoclasti	WP_013280589.1
516950	000019985.1	Spneumoniae	WP_000661009.1
518	000829175.1	Bbronchisepti	WP_033454558.1
520	001013565.1	Bpertussis	WP_003808393.1
520	001307525.1	Bpertussis	WP_003808393.1
520	001307565.1	Bpertussis	WP_003808393.1
520	001307585.1	Bpertussis	WP_003808393.1
520	001307605.1	Bpertussis	WP_003808393.1
520	001307625.1	Bpertussis	WP_003808393.1
520	001307645.1	Bpertussis	WP_003808393.1
520	001307665.1	Bpertussis	WP_003808393.1
520	001307685.1	Bpertussis	WP_003808393.1
520	001307705.1	Bpertussis	WP_003808393.1
520	001307725.1	Bpertussis	WP_003808393.1
520	001307745.1	Bpertussis	WP_003808393.1
521098	000024285.1	Aacidocaldari	WP_008340586.1
521098	000024285.1	Aacidocaldari	WP_012809574.1
521460	000022325.1	Cbescii	WP_015908407.1
521460	000022325.1	Cbescii	WP_041727326.1
522306	000024165.1	Caccumulibact	WP_015768438.1
522373	000072485.1	Smaltophilia	WP_012480597.1
52242	001314245.1	Lgallinarum	WP_060471017.1
52242	001314245.1	Lgallinarum	WP_060472056.1
523791	000024085.1	Kkoreensis	WP_015781360.1
523794	000023905.1	Lbuccalis	WP_015770031.1
523796	000009585.1	Saureus	WP_000812828.1
525381	000196595.1	Spneumoniae	WP_000661006.1

525919	000024105.1	Aprevotii	WP_015777937.1
526224	000092845.1	Bmurdochii	WP_013115198.1
526977	000832845.1	Bcereus	WP_000084889.1
526977	000832845.1	Bcereus	WP_001075267.1
527001	000385905.1	Senterica	WP_000978690.1
527001	000385905.1	Senterica	WP_001021059.1
527001	000385905.1	Senterica	WP_001100652.1
527021	000306745.1	Bthuringiensi	WP_000084899.1
527021	000306745.1	Bthuringiensi	WP_001073005.1
528244	001305595.1	Athiophilum	WP_045583002.1
529120	000172635.2	Amacleodii	WP_014950275.1
529122	000497525.2	Bthuringiensi	AHA69260.1
529122	000497525.2	Bthuringiensi	AHA73522.1
529507	000069965.1	Pmirabilis	WP_004239731.1
529507	000069965.1	Pmirabilis	WP_012367547.1
529884	000699505.1	Rlacicola	WP_038503617.1
535289	000022305.1	Aebreus	WP_015913679.1
536056	000023365.1	Ecoli	WP_000040195.1
536056	000023365.1	Ecoli	WP_000978651.1
536056	000023365.1	Ecoli	WP_001020973.1
536056	000023365.1	Ecoli	WP_001295383.1
536056	000023365.1	Ecoli	WP_001326492.1
536227	001038625.1	Ccarboxidivor	WP_007060416.1
536227	001038625.1	Ccarboxidivor	WP_007061883.1
536227	001038625.1	Ccarboxidivor	WP_007063044.1
536232	000022765.1	Cbotulinum	WP_003357785.1
536232	000022765.1	Cbotulinum	WP_012704294.1
537973	000155515.2	Lparacasei	WP_003564627.1
539329	000833455.1	Fphilomiragia	WP_004288241.1
541229	000193355.1	Bthuringiensi	WP_000084899.1
541229	000193355.1	Bthuringiensi	WP_001073005.1
54291	000829965.1	Rornithinolyt	WP_041144143.1
54291	000829965.1	Rornithinolyt	WP_041144422.1
54291	000829965.1	Rornithinolyt	WP_041147218.1
54291	001455225.1	Rornithinolyt	WP_004858145.1
54291	001455225.1	Rornithinolyt	WP_004858936.1
54291	001455225.1	Rornithinolyt	WP_044346282.1
543728	000023345.1	Vparadoxus	WP_012747842.1
543734	000026485.1	Lcasei	WP_012491659.1
543737	001267475.1	Ftularensis	WP_003020112.1
54388	000818075.1	Senterica	WP_000839743.1
54388	000818075.1	Senterica	WP_000978696.1
54388	000818075.1	Senterica	WP_001021039.1

54388	000818075.1	Senterica	WP_001100647.1
54388	000818075.1	Senterica	WP_001292406.1
543913	000342265.1	bproteobacter	WP_015420693.1
544404	000022225.1	Ecoli	WP_000839815.1
544404	000022225.1	Ecoli	WP_000978640.1
544404	000022225.1	Ecoli	WP_001020954.1
544404	000022225.1	Ecoli	WP_001292415.1
544404	000022225.1	Ecoli	WP_001295383.1
544556	000024705.1	Gsp	WP_011230565.1
544556	000024705.1	Gsp	WP_012820430.1
545693	000025825.1	Bmegaterium	WP_013054843.1
545693	000025825.1	Bmegaterium	WP_013056060.1
546	001022155.1	Cfreundii	WP_003018509.1
546	001022155.1	Cfreundii	WP_032934840.1
546	001022155.1	Cfreundii	WP_044700851.1
546	001022275.1	Cfreundii	WP_003018509.1
546	001022275.1	Cfreundii	WP_032934840.1
546	001022275.1	Cfreundii	WP_044700851.1
546	001281005.1	Cfreundii	WP_008786969.1
546	001281005.1	Cfreundii	WP_048236098.1
546	001281005.1	Cfreundii	WP_060683238.1
546269	000163895.2	Falocis	WP_049770195.1
546271	000208405.1	Ssputigena	WP_006192776.1
546271	000208405.1	Ssputigena	WP_013740827.1
546342	000145595.1	Saureus	WP_000812832.1
547048	000186725.1	Ypestis	WP_002210002.1
547048	000186725.1	Ypestis	WP_002210805.1
548	001021995.1	Eaerogenes	WP_015367858.1
548	001021995.1	Eaerogenes	WP_015369855.1
548	001021995.1	Eaerogenes	WP_032713402.1
548	001021995.1	Eaerogenes	WP_047740318.1
548473	000159535.2	Saureus	WP_000812850.1
550	000770155.1	Ecloacae	WP_013095667.1
550	000770155.1	Ecloacae	WP_029882968.1
550	000770155.1	Ecloacae	WP_029883013.1
550	000784865.1	Ecloacae	WP_014883022.1
550	000784865.1	Ecloacae	WP_023336776.1
550	000784865.1	Ecloacae	WP_023337770.1
550	000784905.1	Ecloacae	WP_022647114.1
550	000784905.1	Ecloacae	WP_022649374.1
550	000784905.1	Ecloacae	WP_038415690.1
550	000807405.4	Ecloacae	ALA00953.1
550	000807405.4	Ecloacae	ALA01421.1

550	000807405.4	Ecloacae	ALA03769.1
550	000807425.4	Ecloacae	AKZ83010.1
550	000807425.4	Ecloacae	AKZ83461.1
550	000807425.4	Ecloacae	AKZ85780.1
550	000814125.3	Ecloacae	WP_015572729.1
550	000814125.3	Ecloacae	WP_040117379.1
550	000814125.3	Ecloacae	WP_040118319.1
550	000814205.1	Esp	WP_001303432.1
550	000814205.1	Esp	WP_006809600.1
550	000814205.1	Esp	WP_006810032.1
550	000814225.1	Ecloacae	WP_022650420.1
550	000814225.1	Ecloacae	WP_022650609.1
550	000814225.1	Ecloacae	WP_022651843.1
550	001022015.1	Ecloacae	WP_015571121.1
550	001022015.1	Ecloacae	WP_015572729.1
550	001022015.1	Ecloacae	WP_032620254.1
550	001022055.1	Ecloacae	WP_015571121.1
550	001022055.1	Ecloacae	WP_015572729.1
550	001022055.1	Ecloacae	WP_032620254.1
550	001022075.1	Ecloacae	WP_015571121.1
550	001022075.1	Ecloacae	WP_015572729.1
550	001022075.1	Ecloacae	WP_032620254.1
550	001022255.1	Ecloacae	WP_015571121.1
550	001022255.1	Ecloacae	WP_015572729.1
550	001022255.1	Ecloacae	WP_032620254.1
550	001029645.1	Ecloacae	WP_014168717.1
550	001029645.1	Ecloacae	WP_020884975.1
550	001029645.1	Ecloacae	WP_047956096.1
550537	000009505.1	Senterica	WP_000839732.1
550537	000009505.1	Senterica	WP_000978691.1
550537	000009505.1	Senterica	WP_001021050.1
550537	000009505.1	Senterica	WP_001100652.1
550537	000009505.1	Senterica	WP_001292402.1
550538	000009525.1	Senterica	WP_000978691.1
550538	000009525.1	Senterica	WP_001021056.1
550538	000009525.1	Senterica	WP_001100655.1
550538	000009525.1	Senterica	WP_001292402.1
550542	000174795.2	Gsp	WP_011230565.1
550542	000174795.2	Gsp	WP_012820430.1
551115	000196515.1	Nazollae	WP_013192343.1
552536	000021185.1	Lmonocytogene	WP_012582337.1
554290	000026565.1	Senterica	WP_000839743.1
554290	000026565.1	Senterica	WP_000978696.1

554290	000026565.1	Senterica	WP_001021039.1
554290	000026565.1	Senterica	WP_001100647.1
554290	000026565.1	Senterica	WP_001292406.1
555079	000144645.1	Toceani	WP_013274859.1
555079	000144645.1	Toceani	WP_013276919.1
555778	000024765.1	Hneapolitanus	WP_012823960.1
557598	000021025.1	Lhongkongensi	WP_012696516.1
557599	000157895.2	Mkansasii	AGZ53084.1
557599	000157895.2	Mkansasii	AGZ53465.1
557722	000026645.1	Paeruginosa	WP_003113592.1
557722	000026645.1	Paeruginosa	WP_016253674.1
557724	000756185.1	Bpseudomallei	WP_004191939.1
561007	000069185.1	Mabscessus	YP_001703259.1
56110	000317105.1	Oacuminata	WP_015146685.1
561229	000023565.1	Dchrysanthemi	WP_012770882.1
561230	000023605.1	Pcarotovorum	WP_012773563.1
561231	000024645.1	Pwasabiae	WP_014700965.1
561275	000010685.1	Mbovis	WP_003899363.1
561276	000026665.1	Spneumoniae	WP_000661015.1
561304	000026685.1	Mleprae	WP_010907773.1
562	000597845.1	Ecoli	WP_000978651.1
562	000597845.1	Ecoli	WP_001020973.1
562	000597845.1	Ecoli	WP_001295383.1
562	000597845.1	Ecoli	WP_001326492.1
562	000597845.1	Ecoli	WP_021570042.1
562	000599665.1	Ecoli	WP_000978649.1
562	000599665.1	Ecoli	WP_001295383.1
562	000599665.1	Ecoli	WP_001601040.1
562	000599665.1	Ecoli	WP_025210855.1
562	000784925.1	Ecoli	WP_000671170.1
562	000784925.1	Ecoli	WP_001020966.1
562	000784925.1	Ecoli	WP_001296667.1
562	000784925.1	Ecoli	WP_022645360.1
562	000784925.1	Ecoli	WP_022646105.1
562	000784925.1	Ecoli	WP_022646452.1
562	000801165.1	Ecoli	WP_000839757.1
562	000801165.1	Ecoli	WP_000978649.1
562	000801165.1	Ecoli	WP_001295383.1
562	000801165.1	Ecoli	WP_001342032.1
562	000801165.1	Ecoli	WP_039264359.1
562	000801185.2	Ecoli	WP_000978647.1
562	000801185.2	Ecoli	WP_001021030.1
562	000801185.2	Ecoli	WP_001295383.1

562	000801185.2	Ecoli	WP_001297242.1
562	000801185.2	Ecoli	WP_001616442.1
562	000814145.2	Ecoli	WP_000978651.1
562	000814145.2	Ecoli	WP_001020973.1
562	000814145.2	Ecoli	WP_001295383.1
562	000814145.2	Ecoli	WP_001326492.1
562	000819645.1	Ecoli	WP_000839748.1
562	000819645.1	Ecoli	WP_001295383.1
562	000819645.1	Ecoli	WP_001372249.1
562	000819645.1	Ecoli	WP_044502105.1
562	000819645.1	Ecoli	WP_044502386.1
562	000830035.1	Ecoli	WP_000040191.1
562	000830035.1	Ecoli	WP_000839790.1
562	000830035.1	Ecoli	WP_000978645.1
562	000830035.1	Ecoli	WP_001020950.1
562	000830035.1	Ecoli	WP_001295383.1
562	000833145.1	Ecoli	WP_000040191.1
562	000833145.1	Ecoli	WP_000839790.1
562	000833145.1	Ecoli	WP_000978645.1
562	000833145.1	Ecoli	WP_001020950.1
562	000833145.1	Ecoli	WP_001295383.1
562	000931565.1	Ecoli	WP_000839781.1
562	000931565.1	Ecoli	WP_000978629.1
562	000931565.1	Ecoli	WP_001021005.1
562	000931565.1	Ecoli	WP_001296667.1
562	000931565.1	Ecoli	WP_001531487.1
562	000952955.1	Ecoli	WP_000040195.1
562	000952955.1	Ecoli	WP_000978651.1
562	000952955.1	Ecoli	WP_001020973.1
562	000952955.1	Ecoli	WP_001295383.1
562	000952955.1	Ecoli	WP_001326492.1
562	000953515.1	Ecoli	WP_000040195.1
562	000953515.1	Ecoli	WP_000978651.1
562	000953515.1	Ecoli	WP_001020973.1
562	000953515.1	Ecoli	WP_001295383.1
562	000953515.1	Ecoli	WP_001326492.1
562	000971615.1	Ecoli	WP_000839764.1
562	000971615.1	Ecoli	WP_000978647.1
562	000971615.1	Ecoli	WP_001020973.1
562	000971615.1	Ecoli	WP_001100642.1
562	000971615.1	Ecoli	WP_001342032.1
562	000987875.1	Ecoli	WP_000040192.1
562	000987875.1	Ecoli	WP_000978651.1

562	000987875.1	Ecoli	WP_001021017.1
562	000987875.1	Ecoli	WP_001295383.1
562	000987875.1	Ecoli	WP_014640006.1
562	000988355.1	Ecoli	WP_000040195.1
562	000988355.1	Ecoli	WP_000978651.1
562	000988355.1	Ecoli	WP_001020973.1
562	000988355.1	Ecoli	WP_001295383.1
562	000988355.1	Ecoli	WP_001326492.1
562	000988385.1	Ecoli	WP_000040195.1
562	000988385.1	Ecoli	WP_000978651.1
562	000988385.1	Ecoli	WP_001020973.1
562	000988385.1	Ecoli	WP_001295383.1
562	000988385.1	Ecoli	WP_001326492.1
562	000988425.1	Ecoli	AKF62426.1
562	000988425.1	Ecoli	AKF62881.1
562	000988425.1	Ecoli	AKF65033.1
562	000988425.1	Ecoli	AKF66114.1
562	000988425.1	Ecoli	AKF66129.1
562	000988445.1	Ecoli	AKF66566.1
562	000988445.1	Ecoli	AKF67021.1
562	000988445.1	Ecoli	AKF69173.1
562	000988445.1	Ecoli	AKF70254.1
562	000988445.1	Ecoli	AKF70269.1
562	000988465.1	Ecoli	WP_000040195.1
562	000988465.1	Ecoli	WP_000978651.1
562	000988465.1	Ecoli	WP_001020973.1
562	000988465.1	Ecoli	WP_001295383.1
562	000988465.1	Ecoli	WP_001326492.1
562	001007915.1	Ecoli	WP_000839747.1
562	001007915.1	Ecoli	WP_000978649.1
562	001007915.1	Ecoli	WP_001020980.1
562	001007915.1	Ecoli	WP_001295383.1
562	001007915.1	Ecoli	WP_001297242.1
562	001039415.1	Ecoli	WP_000040191.1
562	001039415.1	Ecoli	WP_000839790.1
562	001039415.1	Ecoli	WP_000978645.1
562	001039415.1	Ecoli	WP_001020950.1
562	001039415.1	Ecoli	WP_001295383.1
562	001043215.1	Ecoli	WP_000040195.1
562	001043215.1	Ecoli	WP_000978651.1
562	001043215.1	Ecoli	WP_001020973.1
562	001043215.1	Ecoli	WP_001295383.1
562	001043215.1	Ecoli	WP_001326492.1

562	001183645.1	Ecoli	WP_000040195.1
562	001183645.1	Ecoli	WP_000978651.1
562	001183645.1	Ecoli	WP_001020973.1
562	001183645.1	Ecoli	WP_001295383.1
562	001183645.1	Ecoli	WP_001326492.1
562	001183665.1	Ecoli	WP_000040195.1
562	001183665.1	Ecoli	WP_000978651.1
562	001183665.1	Ecoli	WP_001020973.1
562	001183665.1	Ecoli	WP_001295383.1
562	001183665.1	Ecoli	WP_001326492.1
562	001183685.1	Ecoli	WP_000040195.1
562	001183685.1	Ecoli	WP_000978651.1
562	001183685.1	Ecoli	WP_001020973.1
562	001183685.1	Ecoli	WP_001295383.1
562	001183685.1	Ecoli	WP_001326492.1
562	001276585.1	Ecoli	WP_000040195.1
562	001276585.1	Ecoli	WP_000978651.1
562	001276585.1	Ecoli	WP_001020973.1
562	001276585.1	Ecoli	WP_001295383.1
562	001276585.1	Ecoli	WP_001326492.1
562	001280325.1	Ecoli	WP_000671170.1
562	001280325.1	Ecoli	WP_000839776.1
562	001280325.1	Ecoli	WP_000978670.1
562	001280325.1	Ecoli	WP_001021000.1
562	001280325.1	Ecoli	WP_001296667.1
562	001280325.1	Ecoli	WP_001362902.1
562	001280345.1	Ecoli	WP_000671170.1
562	001280345.1	Ecoli	WP_000839776.1
562	001280345.1	Ecoli	WP_000978670.1
562	001280345.1	Ecoli	WP_001021000.1
562	001280345.1	Ecoli	WP_001296667.1
562	001280345.1	Ecoli	WP_001362902.1
562	001280385.1	Ecoli	WP_000671170.1
562	001280385.1	Ecoli	WP_000839776.1
562	001280385.1	Ecoli	WP_000978670.1
562	001280385.1	Ecoli	WP_001021000.1
562	001280385.1	Ecoli	WP_001296667.1
562	001280385.1	Ecoli	WP_001362902.1
562	001280405.1	Ecoli	WP_000839776.1
562	001280405.1	Ecoli	WP_000978670.1
562	001280405.1	Ecoli	WP_001021000.1
562	001280405.1	Ecoli	WP_001296667.1
562	001280405.1	Ecoli	WP_001362902.1

562	001420935.1	Ecoli	WP_001020973.1
562	001420935.1	Ecoli	WP_032205813.1
562	001420935.1	Ecoli	WP_032207721.1
562	001420955.1	Ecoli	WP_000040182.1
562	001420955.1	Ecoli	WP_001020992.1
562	001420955.1	Ecoli	WP_001296667.1
562	001420955.1	Ecoli	WP_001318032.1
562	001420955.1	Ecoli	WP_060565042.1
562	001442495.1	Ecoli	WP_000839748.1
562	001442495.1	Ecoli	WP_000978647.1
562	001442495.1	Ecoli	WP_001020973.1
562	001442495.1	Ecoli	WP_001295383.1
562	001442495.1	Ecoli	WP_001372249.1
562	001455385.1	Ecoli	WP_000040195.1
562	001455385.1	Ecoli	WP_000978651.1
562	001455385.1	Ecoli	WP_001020973.1
562	001455385.1	Ecoli	WP_001295383.1
562	001455385.1	Ecoli	WP_001326492.1
562	001469815.1	Ecoli	WP_000839781.1
562	001469815.1	Ecoli	WP_000978629.1
562	001469815.1	Ecoli	WP_001021005.1
562	001469815.1	Ecoli	WP_001296667.1
562	001469815.1	Ecoli	WP_001531487.1
562	001485455.1	Ecoli	WP_000671170.1
562	001485455.1	Ecoli	WP_001020966.1
562	001485455.1	Ecoli	WP_001296667.1
562	001485455.1	Ecoli	WP_001564026.1
562	001485455.1	Ecoli	WP_022645360.1
562	001485455.1	Ecoli	WP_022646452.1
562970	000092905.1	Ktusciae	WP_013074114.1
562970	000092905.1	Ktusciae	WP_041304752.1
562971	000758265.1	Axylosoxidans	WP_006385861.1
562971	000758265.1	Axylosoxidans	WP_038504053.1
563174	000209755.1	Lmonocytogene	WP_012582337.1
566546	000184185.1	Ecoli	WP_000839749.1
566546	000184185.1	Ecoli	WP_000978647.1
566546	000184185.1	Ecoli	WP_001021030.1
566546	000184185.1	Ecoli	WP_001295383.1
566546	000184185.1	Ecoli	WP_001297242.1
568206	000021445.1	Banthracis	WP_000084880.1
568206	000021445.1	Banthracis	WP_001075286.1
568703	000026505.1	Lrhamnosus	WP_005684411.1
568704	000026525.1	Lrhamnosus	WP_015764501.1

568704	000026525.1	Lrhamnosus	WP_041248008.1
568706	000306945.1	Bpertussis	WP_003808393.1
568707	000318015.1	Bbronchisepti	YP_006968645.1
568708	000027025.1	Senterica	WP_000839734.1
568708	000027025.1	Senterica	WP_000978690.1
568708	000027025.1	Senterica	WP_001021054.1
568708	000027025.1	Senterica	WP_001100652.1
568708	000027025.1	Senterica	WP_001292402.1
568709	000493535.1	Senterica	WP_000839738.1
568709	000493535.1	Senterica	WP_000978690.1
568709	000493535.1	Senterica	WP_001021054.1
568709	000493535.1	Senterica	WP_001292402.1
568709	000493535.1	Senterica	WP_022562407.1
568816	000230275.1	Aintestini	WP_014128412.1
568819	000026705.1	Lmonocytogene	WP_012681468.1
570416	000688795.1	Bthuringiensi	WP_000084904.1
570416	000688795.1	Bthuringiensi	WP_001075296.1
571	000724525.1	Koxytoca	WP_004848492.1
571	000724525.1	Koxytoca	WP_014226846.1
571	000724525.1	Koxytoca	WP_014228008.1
571	001022115.1	Koxytoca	WP_004099846.1
571	001022115.1	Koxytoca	WP_024273750.1
571	001022115.1	Koxytoca	WP_047720685.1
571	001022195.1	Koxytoca	WP_004848492.1
571	001022195.1	Koxytoca	WP_014226846.1
571	001022195.1	Koxytoca	WP_025107388.1
571	001022295.1	Koxytoca	WP_004099846.1
571	001022295.1	Koxytoca	WP_024273750.1
571	001022295.1	Koxytoca	WP_047720685.1
572264	000022505.1	Bcereus	WP_000084880.1
572264	000022505.1	Bcereus	WP_001075278.1
572418	000253355.1	Mafricanum	WP_003910589.1
572477	000025485.1	Avinosum	WP_012971255.1
572544	000165505.1	Ipolytropus	WP_013388924.1
573	000739495.1	Kpneumoniae	WP_002889384.1
573	000739495.1	Kpneumoniae	WP_002892486.1
573	000739495.1	Kpneumoniae	WP_004151766.1
573	000764615.1	Kpneumoniae	WP_002892486.1
573	000764615.1	Kpneumoniae	WP_004194933.1
573	000764615.1	Kpneumoniae	WP_038434373.1
573	000775955.1	Kpneumoniae	WP_002889384.1
573	000775955.1	Kpneumoniae	WP_009484134.1
573	000775955.1	Kpneumoniae	WP_038434551.1

573	000807395.3	Kpneumoniae	AJB55989.1
573	000807395.3	Kpneumoniae	AJB58932.1
573	000814305.1	Kpneumoniae	WP_002889384.1
573	000814305.1	Kpneumoniae	WP_002892486.1
573	000814305.1	Kpneumoniae	WP_004151766.1
573	000968155.1	Kpneumoniae	WP_002889384.1
573	000968155.1	Kpneumoniae	WP_002892486.1
573	000968155.1	Kpneumoniae	WP_004197024.1
573	001022035.1	Kpneumoniae	WP_002889384.1
573	001022035.1	Kpneumoniae	WP_002892486.1
573	001022035.1	Kpneumoniae	WP_004151766.1
573	001022175.1	Kpneumoniae	WP_002889384.1
573	001022175.1	Kpneumoniae	WP_002892486.1
573	001022175.1	Kpneumoniae	WP_004174419.1
573	001022235.1	Kpneumoniae	WP_002889384.1
573	001022235.1	Kpneumoniae	WP_002892486.1
573	001022235.1	Kpneumoniae	WP_004151766.1
573	001307175.1	Kpneumoniae	WP_002892486.1
573	001307175.1	Kpneumoniae	WP_004194933.1
573	001307175.1	Kpneumoniae	WP_043906894.1
573	001455995.1	Kpneumoniae	WP_002889384.1
573	001455995.1	Kpneumoniae	WP_002892486.1
573	001455995.1	Kpneumoniae	WP_009308638.1
573	001456055.1	Kpneumoniae	WP_002889384.1
573	001456055.1	Kpneumoniae	WP_002892486.1
573	001456055.1	Kpneumoniae	WP_004174419.1
573	001456095.1	Kpneumoniae	WP_002889384.1
573	001456095.1	Kpneumoniae	WP_002892486.1
573	001456095.1	Kpneumoniae	WP_004174419.1
573	001456135.1	Kpneumoniae	WP_002889384.1
573	001456135.1	Kpneumoniae	WP_002892486.1
573	001456135.1	Kpneumoniae	WP_004174419.1
573	001482345.1	Kpneumoniae	WP_002892486.1
573	001482345.1	Kpneumoniae	WP_004185624.1
573	001482345.1	Kpneumoniae	WP_058837148.1
573061	000145275.1	Ccellulovoran	WP_010075342.1
573061	000145275.1	Ccellulovoran	WP_029169275.1
573062	000148425.1	Tsp	WP_003867357.1
573062	000148425.1	Tsp	WP_009052285.1
573235	000091005.1	Ecoli	WP_000839764.1
573235	000091005.1	Ecoli	WP_000978647.1
573235	000091005.1	Ecoli	WP_001020970.1
573235	000091005.1	Ecoli	WP_001295383.1

573235	000091005.1	Ecoli	WP_001342032.1
573569	000219045.1	Fsp	WP_013923585.1
573737	000972785.2	Poxalativoran	WP_046291164.1
574093	000146975.1	Spneumoniae	WP_000661013.1
574521	000026545.1	Ecoli	WP_000040207.1
574521	000026545.1	Ecoli	WP_000839792.1
574521	000026545.1	Ecoli	WP_000978629.1
574521	000026545.1	Ecoli	WP_001020955.1
574521	000026545.1	Ecoli	WP_001296667.1
576611	000973625.1	Pasymbioticus	WP_046329778.1
57743	001409155.1	Senterica	WP_000839727.1
57743	001409155.1	Senterica	WP_000978689.1
57743	001409155.1	Senterica	WP_001021042.1
57743	001409155.1	Senterica	WP_001100652.1
57743	001409155.1	Senterica	WP_001534205.1
579112	000021605.1	Vcholerae	WP_001019826.1
579112	000021605.1	Vcholerae	WP_001086685.1
579405	000023545.1	Dparadisiaca	WP_015854708.1
57975	000808035.2	Bthailandensi	WP_009892476.1
580327	000145615.1	Tthermosaccha	WP_013296644.1
580327	000145615.1	Tthermosaccha	WP_013296769.1
580327	000145615.1	Tthermosaccha	WP_013298087.1
580331	000025645.1	Titalicus	WP_012994260.1
580331	000025645.1	Titalicus	WP_012995062.1
580332	000025705.1	Slithotrophic	WP_013030664.1
581103	000166075.1	Gsp	WP_003247383.1
581103	000166075.1	Gsp	WP_013401330.1
582744	000023745.1	Mglucosetroph	WP_015829688.1
583345	000023705.1	Mmobilis	WP_015832468.1
583346	000010265.1	Ckluyveri	WP_012103526.1
583346	000010265.1	Ckluyveri	WP_012104115.1
583358	000092965.1	Tmathranii	WP_012995062.1
583358	000092965.1	Tmathranii	WP_013149593.1
584	001281545.1	Pmirabilis	X1
584721	000259545.1	Pmultocida	WP_005716647.1
585	001281565.1	Pvulgaris	WP_017628327.1
585034	000026265.1	Ecoli	WP_000839767.1
585034	000026265.1	Ecoli	WP_000978647.1
585034	000026265.1	Ecoli	WP_001020973.1
585034	000026265.1	Ecoli	WP_001295383.1
585034	000026265.1	Ecoli	WP_001297242.1
585035	000026285.1	Ecoli	YP_002390040.1
585035	000026285.1	Ecoli	YP_002390532.1

585035	000026285.1	Ecoli	YP_002392869.1
585035	000026285.1	Ecoli	YP_002394119.1
585035	000026285.1	Ecoli	YP_002394132.1
585035	000026285.1	Ecoli	YP_002394199.1
585054	000026225.1	Efergusonii	WP_000855539.1
585054	000026225.1	Efergusonii	WP_001021009.1
585054	000026225.1	Efergusonii	WP_002431488.1
585054	000026225.1	Efergusonii	WP_015953586.1
585055	000026245.1	Ecoli	WP_000839766.1
585055	000026245.1	Ecoli	WP_000978647.1
585055	000026245.1	Ecoli	WP_001020973.1
585055	000026245.1	Ecoli	WP_001295383.1
585055	000026245.1	Ecoli	WP_001297242.1
585057	000026345.1	Ecoli	YP_002406235.1
585057	000026345.1	Ecoli	YP_002406677.1
585057	000026345.1	Ecoli	YP_002409308.1
585057	000026345.1	Ecoli	YP_002410410.1
585057	000026345.1	Ecoli	YP_002410425.1
585394	000225345.1	Rhominis	WP_014079238.1
585394	000225345.1	Rhominis	WP_014081315.1
585395	000010745.1	Ecoli	WP_000839768.1
585395	000010745.1	Ecoli	WP_000978651.1
585395	000010745.1	Ecoli	WP_001020975.1
585395	000010745.1	Ecoli	WP_001295383.1
585395	000010745.1	Ecoli	WP_001342032.1
585396	000010765.1	Ecoli	WP_000839773.1
585396	000010765.1	Ecoli	WP_000978647.1
585396	000010765.1	Ecoli	WP_001020973.1
585396	000010765.1	Ecoli	WP_001342032.1
585423	000737575.1	Ssp	WP_052378147.1
585425	000737595.1	Ssp	WP_038554301.1
586416	000725365.1	Taidingensis	WP_038559853.1
586416	000725365.1	Taidingensis	WP_038562509.1
58712	001447095.1	Senterica	WP_000978690.1
58712	001447095.1	Senterica	WP_001100652.1
58712	001447095.1	Senterica	WP_001292402.1
58712	001447095.1	Senterica	WP_023243687.1
58712	001447095.1	Senterica	WP_023244110.1
587753	000698865.1	Pchlororaphis	WP_038632058.1
587753	000963835.1	Pchlororaphis	WP_045883504.1
587753	001023535.1	Pchlororaphis	WP_047738612.1
588	000754345.1	Pstuartii	WP_004923888.1
588	000754345.1	Pstuartii	WP_014656883.1

588858	000022165.1	Senterica	WP_000839738.1
588858	000022165.1	Senterica	WP_000978690.1
588858	000022165.1	Senterica	WP_001021054.1
588858	000022165.1	Senterica	WP_001100652.1
588858	000022165.1	Senterica	WP_001292400.1
590409	000025065.1	Dzeae	WP_012883613.1
591001	000025305.1	Afermentans	WP_012938019.1
591001	000025305.1	Afermentans	WP_012939225.1
591020	000022245.1	Sflexneri	WP_000978635.1
591020	000022245.1	Sflexneri	WP_001020991.1
591020	000022245.1	Sflexneri	WP_005085661.1
591167	000176115.2	Spratensis	WP_014152475.1
591946	000284495.1	Ecoli	WP_000978665.1
591946	000284495.1	Ecoli	WP_001021003.1
591946	000284495.1	Ecoli	WP_001296667.1
591946	000284495.1	Ecoli	WP_001338015.1
591946	000284495.1	Ecoli	WP_001362902.1
59201	001006525.1	Senterica	WP_000839738.1
59201	001006525.1	Senterica	WP_000978690.1
59201	001006525.1	Senterica	WP_001021054.1
59201	001006525.1	Senterica	WP_001100652.1
59201	001006525.1	Senterica	WP_001292402.1
592021	000022865.1	Banthracis	WP_000084880.1
592021	000022865.1	Banthracis	WP_001075286.1
592022	000025805.1	Bmegaterium	WP_013056060.1
592022	000025805.1	Bmegaterium	WP_013081330.1
592316	000175935.2	Psp	WP_013507959.1
592316	000175935.2	Psp	WP_013510387.1
593588	000022585.1	Vcholerae	WP_001019827.1
593588	000022585.1	Vcholerae	WP_001086685.1
594679	000815225.1	Fguangzhouens	WP_039125194.1
595	000953495.1	Senterica	WP_000839737.1
595	000953495.1	Senterica	WP_001021045.1
595	000953495.1	Senterica	WP_001100652.1
595	000953495.1	Senterica	WP_001292402.1
595	000953495.1	Senterica	WP_023993535.1
595495	000147855.3	Ecoli	ADX49413.1
595495	000147855.3	Ecoli	ADX51771.1
595495	000147855.3	Ecoli	ADX52305.1
595495	000147855.3	Ecoli	ADX52749.1
595495	000147855.3	Ecoli	ADX52764.1
595496	000022345.1	Ecoli	WP_000040195.1
595496	000022345.1	Ecoli	WP_000978651.1

595496	000022345.1	Ecoli	WP_001020973.1
595496	000022345.1	Ecoli	WP_001295383.1
595496	000022345.1	Ecoli	WP_001326492.1
595500	000835205.1	Bglumae	WP_042624153.1
595500	000835205.1	Bglumae	WP_042625743.1
595537	000184745.1	Vparadoxus	WP_013541259.1
596153	000179015.2	Adenitrifican	WP_013519508.1
596154	000204645.1	Adenitrifican	WP_013722498.1
59893	001272655.1	Ppeoriae	WP_013310938.1
59893	001272655.1	Ppeoriae	WP_053324312.1
59919	000011465.1	Pmarinus	WP_011132717.1
59920	000012465.1	Pmarinus	WP_011294754.1
59922	000015705.1	Pmarinus	WP_011825641.1
600	001305815.1	Senterica	WP_000978690.1
600	001305815.1	Senterica	WP_001021052.1
600	001305815.1	Senterica	WP_001100652.1
600	001305815.1	Senterica	WP_001292402.1
600	001305815.1	Senterica	WP_017465457.1
60480	000014685.1	Ssp	WP_011624390.1
60481	000014665.1	Ssp	WP_011624840.1
60520	001443645.1	Lparaplantaru	WP_033609985.1
60550	001028665.1	Bpyrrocinia	WP_047898758.1
60550	001028665.1	Bpyrrocinia	WP_047901303.1
60550	001028665.1	Bpyrrocinia	WP_047903595.1
608506	000145215.1	Cobsidiansis	WP_013289790.1
608506	000145215.1	Cobsidiansis	WP_041742795.1
610130	000144625.1	Csaccharolyti	WP_013270761.1
610130	000144625.1	Csaccharolyti	WP_013271109.1
611	001441205.1	Senterica	WP_000839725.1
611	001441205.1	Senterica	WP_000978689.1
611	001441205.1	Senterica	WP_001021052.1
611	001441205.1	Senterica	WP_001100652.1
611	001441205.1	Senterica	WP_001292402.1
614	000975245.1	Sliquefaciens	WP_020828344.1
614	000975245.1	Sliquefaciens	WP_046372636.1
614	000975245.1	Sliquefaciens	WP_046372778.1
614	000975245.1	Sliquefaciens	WP_046374642.1
615	001022215.1	Smarcescens	WP_047729236.1
615	001022215.1	Smarcescens	WP_047729992.1
615	001022215.1	Smarcescens	WP_047729994.1
615	001022215.1	Smarcescens	WP_047730847.1
615	001280365.1	Smarcescens	WP_016929309.1
615	001280365.1	Smarcescens	WP_033635725.1

615	001280365.1	Smarcescens	WP_033644924.1
615	001280365.1	Smarcescens	WP_060660315.1
615	001294565.1	Smarcescens	WP_033649629.1
615	001294565.1	Smarcescens	WP_033649815.1
615	001294565.1	Smarcescens	WP_039567427.1
615	001294565.1	Smarcescens	WP_060706945.1
615	001417865.1	Smarcescens	WP_004931974.1
615	001417865.1	Smarcescens	WP_004931982.1
615	001417865.1	Smarcescens	WP_060558422.1
615	001417865.1	Smarcescens	WP_060560469.1
61635	000967915.1	Abrassicae	WP_030004440.1
61645	000807415.4	Easburiae	AKZ72127.1
61645	000807415.4	Easburiae	AKZ72528.1
61645	000807415.4	Easburiae	AKZ74918.1
61645	001022095.1	Easburiae	WP_045401114.1
61645	001022095.1	Easburiae	WP_047742496.1
61645	001022095.1	Easburiae	WP_047743798.1
61647	000757785.1	Pgergoviae	WP_043082011.1
61647	000757785.1	Pgergoviae	WP_043082064.1
61647	000757785.1	Pgergoviae	WP_043082510.1
61647	000757785.1	Pgergoviae	WP_043085195.1
61647	000757785.1	Pgergoviae	WP_052097838.1
61648	001022135.1	Kintermedia	WP_047368897.1
61648	001022135.1	Kintermedia	WP_047371758.1
621	001027225.1	Sboydii	WP_000040185.1
621	001027225.1	Sboydii	WP_000424603.1
621	001027225.1	Sboydii	WP_000978676.1
623	000953035.1	Sflexneri	WP_000978635.1
623	000953035.1	Sflexneri	WP_001021013.1
623	000953035.1	Sflexneri	WP_005099260.1
626418	000022645.2	Bglumae	WP_012734936.1
626418	000022645.2	Bglumae	WP_015876723.1
62928	000061505.1	Asp	WP_011766923.1
62977	000046845.1	Asp	WP_004923764.1
630	000834195.1	Yenterocoliti	WP_005173170.1
630	000834195.1	Yenterocoliti	WP_005173646.1
630	000834195.1	Yenterocoliti	WP_005173675.1
630	000834195.1	Yenterocoliti	WP_005175347.1
630	000834735.1	Yenterocoliti	WP_005157162.1
630	000834735.1	Yenterocoliti	WP_013649152.1
630	000834735.1	Yenterocoliti	WP_013649386.1
630	000834735.1	Yenterocoliti	WP_013650370.1
630	000987925.1	Yenterocoliti	WP_005175347.1

630	000987925.1	Yenterocoliti	WP_046050275.1
630	000987925.1	Yenterocoliti	WP_046050355.1
630	000987925.1	Yenterocoliti	WP_046694571.1
630	001305635.1	Yenterocoliti	WP_005157162.1
630	001305635.1	Yenterocoliti	WP_013649152.1
630	001305635.1	Yenterocoliti	WP_013649386.1
630	001305635.1	Yenterocoliti	WP_013650370.1
630626	000262305.1	Sblattae	WP_002439058.1
630626	000262305.1	Sblattae	WP_002440785.1
630626	000262305.1	Sblattae	WP_002441073.1
630626	000262305.1	Sblattae	WP_002441748.1
630626	000262305.1	Sblattae	WP_002444891.1
631	000834515.1	Yintermedia	WP_032906684.1
631	000834515.1	Yintermedia	WP_042569177.1
631	000834515.1	Yintermedia	WP_042569236.1
632	000834235.1	Ypestis	WP_002210002.1
632	000834235.1	Ypestis	WP_002210805.1
632	000834275.1	Ypestis	WP_002210002.1
632	000834275.1	Ypestis	WP_002210805.1
632	000834335.1	Ypestis	WP_002210002.1
632	000834335.1	Ypestis	WP_002210805.1
632	000834495.1	Ypestis	WP_002210002.1
632	000834495.1	Ypestis	WP_002210805.1
632	000834775.1	Ypestis	WP_002210002.1
632	000834775.1	Ypestis	WP_002210805.1
632	000834885.1	Ypestis	WP_002210002.1
632	000834885.1	Ypestis	WP_002210805.1
632292	000166355.1	Chydrothermal	WP_013402643.1
632292	000166355.1	Chydrothermal	WP_013404009.1
632335	000166695.1	Ckristjansson	WP_013431900.1
632335	000166695.1	Ckristjansson	WP_013433030.1
632348	000166775.1	Ckronotskyens	WP_013431050.1
632348	000166775.1	Ckronotskyens	WP_041741537.1
632516	000193435.3	Clactoaceticu	AEM73839.1
632516	000193435.3	Clactoaceticu	AEM74421.1
632518	000166335.1	Cowensensis	WP_013411366.1
632518	000166335.1	Cowensensis	WP_041738068.1
633	000750315.1	Ypseudotuberc	WP_011191987.1
633	000750315.1	Ypseudotuberc	WP_038401022.1
633	000834355.1	Ypseudotuberc	WP_011191987.1
633	000834355.1	Ypseudotuberc	WP_012413942.1
633	000834415.1	Ypseudotuberc	WP_011191987.1
633	000834415.1	Ypseudotuberc	WP_038401022.1

633	000834435.1	Ypseudotuberc	WP_011191987.1
633	000834435.1	Ypseudotuberc	WP_012413942.1
633699	000091405.1	Ljohnsonii	WP_012846826.1
634499	000027265.1	Epyrifoliae	WP_012669239.1
634499	000027265.1	Epyrifoliae	WP_041474093.1
634500	000196615.1	Ebillingiae	WP_013200861.1
634500	000196615.1	Ebillingiae	WP_013203701.1
634503	000022885.2	Eictaluri	WP_015869742.1
634503	000022885.2	Eictaluri	WP_015869760.1
634503	000022885.2	Eictaluri	WP_015870265.1
634503	000022885.2	Eictaluri	WP_015872621.1
634503	000022885.2	Eictaluri	WP_015872623.1
634956	000178395.2	Gthermoglucos	WP_003247383.1
634956	000178395.2	Gthermoglucos	WP_003252032.1
635013	000092945.1	Tpotens	WP_013119009.1
636	001011055.1	Etarda	WP_005280999.1
636	001011055.1	Etarda	WP_005282982.1
636	001011055.1	Etarda	WP_047059123.1
636	001011055.1	Etarda	WP_047059126.1
636	001011055.1	Etarda	WP_047060587.1
63737	000020025.1	Npunctiforme	WP_012407291.1
637380	000143605.1	Bcereus	WP_000084883.1
637380	000143605.1	Bcereus	WP_001075265.1
637381	000022925.1	Lmonocytogene	WP_012951103.1
637382	000022805.1	Ypestis	WP_002210002.1
637382	000022805.1	Ypestis	WP_002210805.1
637385	000022825.1	Ypestis	WP_002210002.1
637385	000022825.1	Ypestis	WP_002210805.1
637386	000022845.1	Ypestis	WP_002210002.1
637386	000022845.1	Ypestis	WP_002210805.1
637887	000184925.1	Rthermocellum	WP_003513882.1
637887	000184925.1	Rthermocellum	WP_003514219.1
637910	000027085.1	Crodentium	WP_012904572.1
637910	000027085.1	Crodentium	WP_012905027.1
637910	000027085.1	Crodentium	WP_012908837.1
640081	000236665.1	Dsuillum	WP_014236346.1
640131	000025465.1	Kvariicola	WP_008806477.1
640131	000025465.1	Kvariicola	WP_012968785.1
640131	000025465.1	Kvariicola	WP_012968903.1
640510	000176935.2	Bsp	WP_013587492.1
640511	000092885.1	Bsp	WP_013088724.1
640512	000148685.1	Bsp	WP_013338491.1
640513	000224675.1	Easburiae	WP_014068983.1

640513	000224675.1	Easburiae	WP_014069359.1
640513	000224675.1	Easburiae	WP_014071721.1
642492	000178835.2	Clentocellum	WP_013655073.1
642492	000178835.2	Clentocellum	WP_013656574.1
643561	000176855.2	Aavenae	WP_013594422.1
643648	000092405.1	Slipocalidus	WP_049764986.1
644	000940915.1	Ahydrophila	WP_017408594.1
644	000963645.1	Ahydrophila	WP_045790429.1
644	001455365.1	Ahydrophila	WP_016349881.1
64471	000014585.1	Ssp	WP_011618956.1
644801	000327065.1	Pstutzeri	WP_015276897.1
645462	000085225.1	Pdifficile	WP_003437158.1
645462	000085225.1	Pdifficile	WP_009892114.1
645657	000209795.2	Bsubtilis	WP_014478592.1
645657	000209795.2	Bsubtilis	WP_014479695.1
645991	000190635.1	Sglycolicus	WP_013623410.1
645991	000190635.1	Sglycolicus	WP_013626168.1
646529	000255115.3	Dacidiphilus	AFM39145.1
646529	000255115.3	Dacidiphilus	AFM39228.1
649639	000177235.2	Bcellulosilyt	WP_013486664.1
649639	000177235.2	Bcellulosilyt	WP_013489246.1
649716	000834985.1	Ypestis	WP_002210002.1
65093	000317635.1	Hsp	WP_015226450.1
652	001447335.1	Aschubertii	WP_060586925.1
652103	000177255.2	Rpalustris	WP_041807403.1
652616	000350205.1	Mtuberculosis	WP_003899363.1
65393	000021825.1	Csp	WP_015955419.1
653938	000093125.2	Lmonocytogene	WP_012951103.1
655816	000146565.1	Bsubtilis	WP_003218293.1
655816	000146565.1	Bsubtilis	WP_003221301.1
655817	000148365.1	Ecoli	WP_000978663.1
655817	000148365.1	Ecoli	WP_001021003.1
655817	000148365.1	Ecoli	WP_001296667.1
655817	000148365.1	Ecoli	WP_001305317.1
655817	000148365.1	Ecoli	WP_001524296.1
656178	000934605.1	Pvervacti	WP_044454786.1
656179	001029105.1	Pfaecigallina	WP_047907331.1
659019	000193185.2	Mtuberculosis	WP_003899363.1
660470	000147715.3	Mprima	AFK06994.1
661410	000083545.1	Mextorquens	WP_015821424.1
663	001469735.1	Valginolyticu	WP_005374800.1
663	001469735.1	Valginolyticu	WP_005451159.1
663278	000178115.2	Eharbinense	WP_013484675.1

663278	000178115.2	Eharbinense	WP_049776666.1
663951	000027045.1	Saureus	WP_000812832.1
665029	000091565.1	Eamylovora	WP_004155423.1
665029	000091565.1	Eamylovora	WP_004159429.1
666	000765415.1	Vcholerae	WP_001019827.1
666	000765415.1	Vcholerae	WP_001086685.1
666	000969235.1	Vcholerae	WP_001086691.1
666	000969235.1	Vcholerae	WP_046122446.1
666	000969265.1	Vcholerae	WP_046126335.1
666	000969265.1	Vcholerae	WP_046127384.1
666	001045415.1	Vcholerae	WP_001019827.1
666	001045415.1	Vcholerae	WP_001086685.1
666681	000093025.1	Mversatilis	WP_013147809.1
666686	000242895.3	Bsp	AGK51809.1
666686	000242895.3	Bsp	AGK52919.1
66692	000009825.1	Bclausii	WP_011244913.1
66692	000009825.1	Bclausii	WP_011247250.1
667120	000264765.2	Eanguillarum	WP_034163233.1
667120	000264765.2	Eanguillarum	WP_034164754.1
667120	000264765.2	Eanguillarum	WP_034165623.1
667120	000264765.2	Eanguillarum	WP_038631285.1
667120	000264765.2	Eanguillarum	WP_038631389.1
670	001244315.1	Vparahaemolyt	WP_011106546.1
670	001244315.1	Vparahaemolyt	WP_053046750.1
670	001304775.1	Vparahaemolyt	WP_005481757.1
670	001304775.1	Vparahaemolyt	WP_011106546.1
670	001433415.1	Vparahaemolyt	WP_005481757.1
670	001433415.1	Vparahaemolyt	WP_011106546.1
670	001433415.1	Vparahaemolyt	WP_025548026.1
670	001433415.1	Vparahaemolyt	WP_057619330.1
671143	000091165.1	Cmethylomirab	WP_012814310.1
672	000746665.1	Vvulnificus	WP_011079948.1
672	000746665.1	Vvulnificus	WP_011151494.1
672	001433435.1	Vvulnificus	WP_011079948.1
672	001433435.1	Vvulnificus	WP_060533060.1
673518	000512775.1	Banthracis	WP_000084880.1
673518	000512775.1	Banthracis	WP_001075286.1
676032	000195555.1	Fcf	WP_014547777.1
679895	000750555.1	Ecoli	WP_000040195.1
679895	000750555.1	Ecoli	WP_000978651.1
679895	000750555.1	Ecoli	WP_001020973.1
679895	000750555.1	Ecoli	WP_001295383.1
679895	000750555.1	Ecoli	WP_001326492.1

679936	000237975.1	Sacidophilus	WP_013985131.1
681288	000024585.1	Saureus	WP_000812829.1
682634	000176835.2	Splymuthica	WP_004952204.1
682634	000176835.2	Splymuthica	WP_006327912.1
682795	000178955.2	Gmallensis	WP_014265075.1
683837	000027145.1	Lseeligeri	WP_012986540.1
685038	000183345.1	Ecoli	YP_006118555.1
685038	000183345.1	Ecoli	YP_006118989.1
685038	000183345.1	Ecoli	YP_006121258.1
685038	000183345.1	Ecoli	YP_006122472.1
685038	000183345.1	Ecoli	YP_006122487.1
685039	000210315.1	Saureus	WP_000812844.1
685727	000196695.1	Requi	WP_013416253.1
686	000963555.1	Vcholerae	WP_001019827.1
686	000963555.1	Vcholerae	WP_001086685.1
688245	000093145.2	Ctestosteroni	WP_012837900.1
68895	000832305.1	Cbasilensis	WP_043348756.1
691437	000092445.1	Gsp	WP_011230565.1
691437	000092445.1	Gsp	WP_013143956.1
692420	000196735.1	Bamyloliquefa	WP_013350743.1
692420	000196735.1	Bamyloliquefa	WP_013352125.1
693216	000027065.2	Cturicensis	WP_012815367.1
693216	000027065.2	Cturicensis	WP_015740544.1
693216	000027065.2	Cturicensis	WP_015742504.1
69328	000494915.1	Psp	WP_023379745.1
693444	000330845.1	Ebacterium	WP_015963041.1
693444	000330845.1	Ebacterium	WP_015964930.1
693444	000330845.1	Ebacterium	WP_015965341.1
693444	000330845.1	Ebacterium	WP_015965830.1
693746	000283575.1	Ovalericigene	WP_014117917.1
693746	000283575.1	Ovalericigene	WP_041615580.1
693970	000215895.1	Sbaltica	WP_011848112.1
693973	000178875.2	Sbaltica	WP_012197746.1
693974	000147735.3	Sbaltica	AEG09620.1
693986	000757795.1	Moryzae	WP_043348722.1
695562	000194115.1	Lamylovorus	WP_014565799.1
696281	000215085.1	Druminis	WP_013840193.1
696281	000215085.1	Druminis	WP_013842840.1
696406	000212715.2	Ecoli	WP_000040192.1
696406	000212715.2	Ecoli	WP_000839760.1
696406	000212715.2	Ecoli	WP_000978651.1
696406	000212715.2	Ecoli	WP_001020973.1
696406	000212715.2	Ecoli	WP_001295383.1

696867	000335875.2	Senterica	WP_000839732.1
696867	000335875.2	Senterica	WP_000978691.1
696867	000335875.2	Senterica	WP_001100652.1
696867	000335875.2	Senterica	WP_001292402.1
696867	000335875.2	Senterica	WP_001680209.1
697281	000213255.1	Maustraliensi	WP_041643771.1
697281	000213255.1	Maustraliensi	WP_041644578.1
697283	000299015.1	Spneumoniae	WP_000665647.1
697284	000511405.1	Plarvae	WP_023482262.1
697284	000511405.1	Plarvae	WP_024094445.1
697303	000147695.3	Twiegelii	AEM77612.1
697303	000147695.3	Twiegelii	AEM78556.1
697329	000179635.2	Ralbus	WP_013499343.1
697329	000179635.2	Ralbus	WP_050793268.1
698737	000025085.1	Slugdunensis	WP_002460725.1
698737	000025085.1	Slugdunensis	WP_002479477.1
698761	000330885.1	Rtropici	WP_015343834.1
698936	000147775.3	Smeliloti	AEG07021.1
698936	000147775.3	Smeliloti	AEG07022.1
698948	000328545.1	Tthermosaccha	WP_013298087.1
698948	000328545.1	Tthermosaccha	WP_015310643.1
698948	000328545.1	Tthermosaccha	WP_015310744.1
699034	000211235.1	Pdifficile	YP_006198017.1
699034	000211235.1	Pdifficile	YP_006200597.1
699035	000210435.1	Pdifficile	WP_003421509.1
699035	000210435.1	Pdifficile	WP_009905185.1
699246	000025225.2	Mindolicus	WP_012993293.1
701177	000025165.1	Ecoli	WP_000839816.1
701177	000025165.1	Ecoli	WP_000978640.1
701177	000025165.1	Ecoli	WP_001020954.1
701177	000025165.1	Ecoli	WP_001292415.1
701177	000025165.1	Ecoli	WP_001295383.1
701347	000164865.1	Elignolyticus	WP_013364807.1
701347	000164865.1	Elignolyticus	WP_013365210.1
701347	000164865.1	Elignolyticus	WP_013367100.1
701347	000164865.1	Elignolyticus	WP_013367489.1
701347	000164865.1	Elignolyticus	WP_013367518.1
701347	000164865.1	Elignolyticus	WP_013368001.1
703339	000025145.2	Saureus	ADC36667.1
706191	000025405.2	Pananatis	WP_013027109.1
707235	000224435.1	Mtuberculosis	WP_003899363.1
707241	000218265.1	Smeliloti	WP_014531133.1
707241	000218265.1	Smeliloti	WP_014531824.1

707241	000218265.1	Smeliloti	WP_014531825.1
70775	000831585.1	Pplecoglossic	WP_024087408.1
710421	000266905.1	Mchubuense	WP_014813352.1
710685	000230895.3	Mrhodesiae	AEV73953.1
712538	001189555.1	Ssp	WP_050341370.1
712538	001189555.1	Ssp	WP_050343713.1
712898	000148935.1	Pvagans	WP_013356852.1
712898	000148935.1	Pvagans	WP_013358988.1
712961	000143435.1	Lsalivarius	WP_014568079.1
713604	000220945.1	Amediterranei	WP_013226148.1
713604	000220945.1	Amediterranei	WP_013229207.1
71421	000027305.1	Hinfluenzae	NP_438749.1
714359	000092165.1	Bthuringiensi	WP_000084899.1
714359	000092165.1	Bthuringiensi	WP_001075300.1
714962	000025745.1	Ecoli	WP_000839776.1
714962	000025745.1	Ecoli	WP_001296667.1
714962	000025745.1	Ecoli	WP_001362902.1
716540	000027205.1	Eamylovora	WP_004155423.1
716540	000027205.1	Eamylovora	WP_004159429.1
716541	000025565.1	Ecloacae	YP_003611501.1
716541	000025565.1	Ecloacae	YP_003613510.1
716541	000025565.1	Ecloacae	YP_003614784.1
717522	000234725.1	Mbovis	WP_003899363.1
717605	000227705.3	Tcomposti	AGA58355.1
717605	000227705.3	Tcomposti	AGA59763.1
717772	000227665.3	Taerophilum	AHF01922.1
717773	000214825.1	Tcyclicum	WP_013835985.1
718251	000146305.1	Etarda	WP_012847161.1
718251	000146305.1	Etarda	WP_012847625.1
718251	000146305.1	Etarda	WP_012849827.1
718274	000283735.1	Senterica	WP_000839738.1
718274	000283735.1	Senterica	WP_001021054.1
718274	000283735.1	Senterica	WP_001100652.1
718274	000283735.1	Senterica	WP_001292402.1
720554	000237085.1	Cclariflavum	WP_014253975.1
720554	000237085.1	Cclariflavum	WP_027622270.1
720555	000165925.1	Batrophaeus	WP_003328608.1
720555	000165925.1	Batrophaeus	WP_003329126.1
72407	000742755.1	Kpneumoniae	WP_002889384.1
72407	000742755.1	Kpneumoniae	WP_002892486.1
72407	000742755.1	Kpneumoniae	WP_004197024.1
72758	001028645.1	Scapitis	WP_047797017.1
741091	000187705.1	Rsp	WP_013574210.1

741091	000187705.1	Rsp	WP_013576854.1
741093	000262125.1	Ecoli	WP_000839815.1
741093	000262125.1	Ecoli	WP_000978640.1
741093	000262125.1	Ecoli	WP_001020954.1
741093	000262125.1	Ecoli	WP_001292415.1
741093	000262125.1	Ecoli	WP_001295383.1
742013	000214395.1	Dsp	WP_013803052.1
743277	000193595.3	Bpertussis	AIW90769.1
743720	000213805.1	Pfulva	WP_013791358.1
743835	000512835.1	Banthracis	WP_000084880.1
743835	000512835.1	Banthracis	WP_001075286.1
743973	000276685.1	Tequigenitali	WP_013521635.1
745156	000829985.1	Ecoli	WP_000839760.1
745156	000829985.1	Ecoli	WP_000978651.1
745156	000829985.1	Ecoli	WP_001020973.1
745156	000829985.1	Ecoli	WP_001295383.1
745156	000829985.1	Ecoli	WP_021570718.1
745156	000829985.1	Ecoli	WP_021570940.1
745310	000347675.2	Ssp	WP_015457230.1
745310	000347675.2	Ssp	WP_041864870.1
74546	000012645.1	Pmarinus	WP_011376645.1
74547	000011485.1	Pmarinus	WP_011130438.1
746697	000265385.1	Asublithincol	WP_014783579.1
747	000754275.1	Pmultocida	WP_014326116.1
748247	000349945.1	Asp	WP_015434928.1
748280	000283535.1	Psp	WP_014087637.1
748280	000283535.1	Psp	WP_014088400.1
748672	000834945.1	Ypseudotuberc	WP_011191987.1
748672	000834945.1	Ypseudotuberc	WP_012104654.1
748678	000834925.1	Ypestis	WP_002210002.1
748678	000834925.1	Ypestis	WP_002210805.1
748727	000143685.1	Cljungdahlii	WP_013236828.1
748727	000143685.1	Cljungdahlii	WP_013240050.1
749927	000196835.1	Amediterranei	YP_003766478.1
749927	000196835.1	Amediterranei	YP_003769566.1
75105	001449005.1	Pcaribensis	WP_054929876.1
75105	001449005.1	Pcaribensis	WP_060608099.1
75379	000092605.1	Tintermedia	WP_013123127.1
754093	000497505.1	Sdysenteriae	YP_008847682.1
754093	000497505.1	Sdysenteriae	YP_008847683.1
754093	000497505.1	Sdysenteriae	YP_008851586.1
754093	000497505.1	Sdysenteriae	YP_008852785.1
754093	000497505.1	Sdysenteriae	YP_008853296.1

754093	000497505.1	Sdysenteriae	YP_008853297.1
754523	000517605.1	Rleguminosaru	WP_041472645.1
755178	000317675.1	Caponinum	WP_041922552.1
755731	000244875.1	Csp	WP_014311858.1
756499	000243155.3	Ddehalogenans	AFL98545.1
756499	000243155.3	Ddehalogenans	AFL98644.1
756828	000800825.1	Bsp	WP_039175872.1
756828	000800825.1	Bsp	WP_039178238.1
757424	000143225.1	Hseropedicae	WP_013235395.1
758678	000092345.1	Cbotulinum	WP_012100824.1
758793	000402035.1	Bsp	WP_044041918.1
758796	000828875.1	Bsp	WP_014190694.1
758831	000831565.1	Ecoli	WP_000839757.1
758831	000831565.1	Ecoli	WP_000978613.1
758831	000831565.1	Ecoli	WP_001020973.1
758831	000831565.1	Ecoli	WP_001297242.1
758831	000831565.1	Ecoli	WP_023566340.1
760568	000214705.1	Dkuznetsovii	WP_013821200.1
76114	000025965.1	Aaromaticum	WP_011238315.1
762376	000165835.1	Axylosoxidans	WP_013396238.1
762376	000165835.1	Axylosoxidans	WP_013396374.1
766760	000408885.1	Bparalichenif	WP_020449790.1
766760	000408885.1	Bparalichenif	WP_020451250.1
76731	001483865.1	Rdepolymerans	WP_058935494.1
767455	000182835.1	Ldelbrueckii	WP_013439207.1
767456	000525715.1	Lhelveticus	WP_025283554.1
767456	000525715.1	Lhelveticus	WP_025283900.1
767462	000189515.1	Lhelveticus	WP_014564182.1
767462	000189515.1	Lhelveticus	WP_041809532.1
767817	000233715.3	Dgibsoniae	AGK99704.1
768490	000214195.1	Ssp	WP_013814260.1
768490	000214195.1	Ssp	WP_013814499.1
768492	000214235.1	Splymuthica	WP_013814260.1
768492	000214235.1	Splymuthica	WP_013814499.1
768493	000214805.1	Ssp	WP_013814260.1
768493	000214805.1	Ssp	WP_013814499.1
768494	000258885.1	Banthracis	WP_000084880.1
768494	000258885.1	Banthracis	WP_001075286.1
76856	001296185.1	Fnucleatum	ALF24983.1
76857	001433955.1	Fnucleatum	WP_060495712.1
76859	001296085.1	Fnucleatum	WP_060676018.1
76869	000019125.1	Pputida	WP_041166547.1
768704	000231385.3	Dmeridiei	AFQ42134.1

768704	000231385.3	Dmeridiei	AFQ42206.1
768706	000235605.1	Dorientis	WP_014182695.1
768706	000235605.1	Dorientis	WP_042330633.1
78245	000017645.1	Xautotrophicu	WP_012112661.1
78398	000769535.1	Pcarotovorum	AIU87544.1
796606	000724485.1	Bmethanolicus	WP_003347112.1
796606	000724485.1	Bmethanolicus	WP_003348775.1
796730	000272775.3	Senterica	WP_000978691.1
796730	000272775.3	Senterica	WP_001021052.1
796730	000272775.3	Senterica	WP_001100652.1
796730	000272775.3	Senterica	WP_001292401.1
796731	000272755.3	Senterica	WP_000839726.1
796731	000272755.3	Senterica	WP_000978691.1
796731	000272755.3	Senterica	WP_001021052.1
796731	000272755.3	Senterica	WP_001100652.1
796731	000272755.3	Senterica	WP_001292401.1
796732	000272735.3	Senterica	WP_000839726.1
796732	000272735.3	Senterica	WP_000978691.1
796732	000272735.3	Senterica	WP_001021052.1
796732	000272735.3	Senterica	WP_001100652.1
796732	000272735.3	Senterica	WP_001292401.1
796733	000272715.3	Senterica	WP_000839726.1
796733	000272715.3	Senterica	WP_000978691.1
796733	000272715.3	Senterica	WP_001021052.1
796733	000272715.3	Senterica	WP_001100652.1
796733	000272715.3	Senterica	WP_001292401.1
79880	000737305.2	Bclausii	WP_035201586.1
79880	000737305.2	Bclausii	WP_035202135.1
80854	000953735.1	Mviscosa	WP_045112309.1
82654	000317065.1	Psp	WP_015164628.1
82985	001026985.1	Pfontium	WP_053007590.1
83331	000008585.1	Mtuberculosis	WP_003899363.1
83332	000195955.2	Mtuberculosis	YP_177889.1
83333	000800765.1	Ecoli	WP_000040195.1
83333	000800765.1	Ecoli	WP_000978651.1
83333	000800765.1	Ecoli	WP_001020973.1
83333	000800765.1	Ecoli	WP_001295383.1
83333	000800765.1	Ecoli	WP_001326492.1
83334	001307215.1	Ecoli	WP_000839815.1
83334	001307215.1	Ecoli	WP_000978640.1
83334	001307215.1	Ecoli	WP_001020954.1
83334	001307215.1	Ecoli	WP_001295383.1
83334	001307215.1	Ecoli	WP_001459256.1

84022	001042715.1	Caceticum	WP_044824589.1
84022	001042715.1	Caceticum	WP_044824611.1
84022	001042715.1	Caceticum	WP_044825303.1
85569	000493675.1	Senterica	WP_000978690.1
85569	000493675.1	Senterica	WP_001021054.1
85569	000493675.1	Senterica	WP_001100652.1
85569	000493675.1	Senterica	WP_001292402.1
85569	000493675.1	Senterica	WP_022742820.1
85643	000021765.1	Tsp	WP_004312326.1
85698	001051055.1	Axylosoxidans	WP_049076248.1
85698	001457475.1	Axylosoxidans	WP_006385861.1
85698	001457475.1	Axylosoxidans	WP_024069700.1
858215	000189775.3	Txylanolyticu	AEF16136.1
858215	000189775.3	Txylanolyticu	AEF16227.1
858215	000189775.3	Txylanolyticu	AEF17164.1
858305	000272895.3	Senterica	AJB07437.1
858305	000272895.3	Senterica	AJB09292.1
858305	000272895.3	Senterica	AJB09817.1
858305	000272895.3	Senterica	AJB10943.1
858305	000272895.3	Senterica	AJB11451.1
858306	000272815.2	Senterica	WP_000839729.1
858306	000272815.2	Senterica	WP_000978691.1
858306	000272815.2	Senterica	WP_001021052.1
858306	000272815.2	Senterica	WP_001100652.1
858306	000272815.2	Senterica	WP_001292401.1
858307	000272835.3	Senterica	WP_000839726.1
858307	000272835.3	Senterica	WP_000978691.1
858307	000272835.3	Senterica	WP_001021052.1
858307	000272835.3	Senterica	WP_001100652.1
858307	000272835.3	Senterica	WP_001292401.1
858308	000272795.2	Senterica	AIT49017.1
858308	000272795.2	Senterica	AIT50131.1
858308	000272795.2	Senterica	AIT50413.1
859199	000188955.5	Senterica	AHW08789.1
859199	000188955.5	Senterica	AHW10553.1
859199	000188955.5	Senterica	AHW11004.1
859199	000188955.5	Senterica	AHW12090.1
859199	000188955.5	Senterica	AHW12614.1
859657	000283475.1	Rsolanacearum	WP_013211801.1
860235	001302585.1	Kphytohabitan	WP_054295236.1
861360	000197735.1	Garilaitensis	WP_013350023.1
86192	000761195.1	Pchlororaphis	WP_038579496.1
862719	000283655.1	Alipoferum	WP_014249344.1

862751	000177195.2	Ssp	WP_014050148.1
862964	000210875.1	Hinfluenzae	WP_015701751.1
862965	000210895.1	Hparainfluenz	WP_014064454.1
863638	000191905.1	Cacetobutylic	WP_010963620.1
863638	000191905.1	Cacetobutylic	WP_010965635.1
86416	000389635.1	Cpasteurianum	WP_015613582.1
86416	000389635.1	Cpasteurianum	WP_015616175.1
864803	000767745.1	Cbeijerinckii	WP_012060311.1
864803	000767745.1	Cbeijerinckii	WP_012060567.1
86662	000775975.1	Bweihenstepha	WP_033708702.1
86662	000775975.1	Bweihenstepha	WP_033709392.1
866768	000023665.1	Ecoli	WP_000040191.1
866768	000023665.1	Ecoli	WP_000978645.1
866768	000023665.1	Ecoli	WP_001020950.1
866768	000023665.1	Ecoli	WP_001295383.1
866768	000023665.1	Ecoli	WP_001326492.1
866895	000284515.1	Hhalophilus	WP_014641337.1
866895	000284515.1	Hhalophilus	WP_014643059.1
866913	000430165.1	Senterica	WP_000839738.1
866913	000430165.1	Senterica	WP_000978690.1
866913	000430165.1	Senterica	WP_001021054.1
866913	000430165.1	Senterica	WP_001100652.1
866913	000430165.1	Senterica	WP_001292402.1
867076	001447315.1	Psp	ALP36746.1
867076	001447315.1	Psp	ALP38491.1
868595	000214435.1	Dnigrificans	WP_013809385.1
868595	000214435.1	Dnigrificans	WP_013809909.1
868597	000223885.1	Smaltophilia	WP_014037588.1
869215	000210955.1	Spneumoniae	WP_000661000.1
869216	000210935.1	Spneumoniae	WP_000661009.1
869269	000210975.1	Spneumoniae	WP_000661010.1
869303	000210995.1	Spneumoniae	WP_000661011.1
869304	000211015.1	Spneumoniae	WP_000661000.1
869306	000211035.2	Spneumoniae	CCP30546.1
869307	000211055.2	Spneumoniae	CCP34505.1
869309	000180515.2	Spneumoniae	CCM08503.1
869311	000211075.1	Spneumoniae	WP_000661010.1
869312	000211095.1	Spneumoniae	WP_000661010.1
869729	000148605.1	Ecoli	WP_000839776.1
869729	000148605.1	Ecoli	WP_000978670.1
869729	000148605.1	Ecoli	WP_001021000.1
869729	000148605.1	Ecoli	WP_001296667.1
869729	000148605.1	Ecoli	WP_001362902.1

869816	000144955.1	Saureus	WP_000812846.1
871738	000512895.1	Drestrictus	WP_019224869.1
871738	000512895.1	Drestrictus	WP_019226625.1
871963	000243135.3	Ddichloroelim	AGA67602.1
871963	000243135.3	Ddichloroelim	AGA67706.1
871968	000231405.3	Dmetallireduc	AHF05688.1
871968	000231405.3	Dmetallireduc	AHF05759.1
875328	000214155.1	Msinense	WP_041318064.1
875328	000214155.1	Msinense	WP_041320102.1
875453	000952975.1	Psp	WP_045078188.1
877468	000442415.1	Senterica	WP_000839726.1
877468	000442415.1	Senterica	WP_000978691.1
877468	000442415.1	Senterica	WP_001021052.1
877468	000442415.1	Senterica	WP_001100652.1
877468	000442415.1	Senterica	WP_001292401.1
87883	000756005.1	Bmultivorans	WP_006398616.1
87883	000756005.1	Bmultivorans	WP_048804479.1
879088	000307615.1	Lmonocytogene	WP_010959076.1
879089	000306905.1	Lmonocytogene	WP_014929846.1
879090	000306985.1	Lmonocytogene	WP_014602264.1
880633	000165775.3	Lhelveticus	AFR22131.1
881621	000252975.1	Livanovii	WP_014093876.1
882020	000307005.1	Lmonocytogene	WP_009930436.1
882094	000307085.1	Lmonocytogene	WP_012681468.1
882095	000307025.1	Lmonocytogene	WP_003727667.1
882096	000307045.1	Lmonocytogene	WP_014601195.1
882097	000307065.1	Lmonocytogene	WP_003728581.1
882378	000198775.1	Prhizoxinica	WP_041753106.1
884204	000260515.1	Bpseudomallei	WP_004191939.1
885275	000233895.1	Ecoli	WP_000839794.1
885275	000233895.1	Ecoli	WP_000978663.1
885275	000233895.1	Ecoli	WP_001021003.1
885275	000233895.1	Ecoli	WP_001296667.1
885275	000233895.1	Ecoli	WP_001524296.1
885276	000233875.1	Ecoli	WP_000839794.1
885276	000233875.1	Ecoli	WP_000978663.1
885276	000233875.1	Ecoli	WP_001021003.1
885276	000233875.1	Ecoli	WP_001296667.1
885276	000233875.1	Ecoli	WP_001524296.1
886715	000329365.2	Senterica	WP_000839732.1
886715	000329365.2	Senterica	WP_000978691.1
886715	000329365.2	Senterica	WP_001021050.1
886715	000329365.2	Senterica	WP_001100652.1

886715	000329365.2	Senterica	WP_001292402.1
886882	000164985.2	Ppolymyxa	ADO54003.1
886882	000164985.2	Ppolymyxa	ADO57309.1
887061	000183115.1	Msp	WP_013441716.1
888727	001189495.1	Esulci	WP_050330459.1
889933	000253135.1	Saureus	WP_000812830.1
891974	000180175.2	Pstali	WP_010618737.1
90370	001302605.1	Senterica	WP_000978690.1
90370	001302605.1	Senterica	WP_001021059.1
90370	001302605.1	Senterica	WP_001100652.1
90371	000636135.1	Senterica	WP_000978690.1
90371	000636135.1	Senterica	WP_001021054.1
90371	000636135.1	Senterica	WP_001100652.1
90371	000636135.1	Senterica	WP_001292402.1
903814	000152245.2	Elimosum	WP_041689961.1
909946	000188735.1	Senterica	WP_000839739.1
909946	000188735.1	Senterica	WP_000978690.1
909946	000188735.1	Senterica	WP_001021054.1
909946	000188735.1	Senterica	WP_001100652.1
909946	000188735.1	Senterica	WP_001292402.1
909954	000204985.1	Ljohnsonii	WP_014567955.1
910348	000257275.1	Ecoli	WP_000978649.1
910348	000257275.1	Ecoli	WP_001021017.1
910348	000257275.1	Ecoli	WP_001295383.1
914127	000186585.1	Vvulnificus	WP_011079948.1
914127	000186585.1	Vvulnificus	WP_011151494.1
914127	000186585.1	Vvulnificus	WP_015728410.1
914149	000166455.2	Vcholerae	AET28128.1
914149	000166455.2	Vcholerae	AET29167.1
926034	000280315.2	Senterica	WP_000839732.1
926034	000280315.2	Senterica	WP_000978691.1
926034	000280315.2	Senterica	WP_001021050.1
926034	000280315.2	Senterica	WP_001100652.1
926034	000280315.2	Senterica	WP_001292402.1
926562	000236705.1	Ohongkongensi	WP_014202848.1
926566	000265425.1	Troseus	WP_014787698.1
927666	000253155.1	Soralis	WP_041170811.1
927704	000284095.1	Sruminantium	WP_014423651.1
927704	000284095.1	Sruminantium	WP_014425426.1
929506	000204565.1	Cbotulinum	WP_013726647.1
930170	000190515.1	Bthuringiensi	WP_000084888.1
930170	000190515.1	Bthuringiensi	WP_001075285.1
930171	000189535.1	Pphenanthreni	WP_013601979.1

930406	000827105.1	Ecoli	WP_000978647.1
930406	000827105.1	Ecoli	WP_001020973.1
930406	000827105.1	Ecoli	WP_001295383.1
930406	000827105.1	Ecoli	WP_001383513.1
93060	000018065.1	Pmarinus	WP_012007909.1
93061	000013425.1	Saureus	YP_499029.1
93062	000012045.1	Saureus	WP_000812832.1
930781	000195395.4	Lmonocytogene	AGR29529.2
930782	000195435.4	Lmonocytogene	EGF40495.2
930944	000253175.1	Yenterocoliti	WP_005156244.1
930944	000253175.1	Yenterocoliti	WP_005157162.1
930944	000253175.1	Yenterocoliti	WP_005157181.1
930944	000253175.1	Yenterocoliti	WP_005176880.1
931276	000340885.1	Csaccharoperb	WP_015391729.1
931276	000340885.1	Csaccharoperb	WP_015394710.1
931281	000183645.1	Pputida	WP_041167131.1
931626	000247605.1	Awoodii	WP_041670172.1
93218	000826965.3	Papista	WP_042116973.1
93218	001010785.1	Papista	WP_042116973.1
93218	001027265.1	Papista	WP_042116973.1
93218	001465595.1	Papista	WP_042116973.1
93219	001465545.1	Pnorimbergens	WP_058375436.1
93220	000604065.2	Ppnomenusa	WP_023597105.1
93220	000767615.2	Ppnomenusa	WP_038620312.1
93221	000815105.1	Ppulmonicola	WP_039409254.1
93222	000814845.1	Psputorum	WP_039399991.1
932677	000270125.1	Pananatis	WP_014594525.1
932677	000270125.1	Pananatis	WP_015701049.1
932677	000270125.1	Pananatis	WP_022622238.1
932919	000197755.2	Lmonocytogene	WP_003727667.1
932920	000210815.2	Lmonocytogene	WP_009930436.1
935198	000020165.1	Cbotulinum	WP_012423322.1
935198	000020165.1	Cbotulinum	WP_012423632.1
935296	000334515.1	Eaerogenes	WP_015367858.1
935296	000334515.1	Eaerogenes	WP_015368149.1
935296	000334515.1	Eaerogenes	WP_015369717.1
935296	000334515.1	Eaerogenes	WP_015369855.1
935297	000195065.1	Vcholerae	WP_001019828.1
935297	000195065.1	Vcholerae	WP_001086684.1
935705	000486365.2	Senterica	WP_000978690.1
935705	000486365.2	Senterica	WP_001021052.1
935705	000486365.2	Senterica	WP_001100652.1
935705	000486365.2	Senterica	WP_001292402.1

935705	000486365.2	Senterica	WP_017465457.1
936156	000186745.1	Bsubtilis	WP_015715092.1
936156	000186745.1	Bsubtilis	WP_015715827.1
937773	000185885.1	Spseudinterme	WP_015728587.1
937774	000185745.1	Tequigenitali	WP_013521635.1
938141	000486445.2	Senterica	WP_001021044.1
938141	000486445.2	Senterica	WP_001100652.1
938141	000486445.2	Senterica	WP_023202161.1
938141	000486445.2	Senterica	WP_023202341.1
938141	000486445.2	Senterica	WP_024146105.1
938142	000487295.2	Senterica	WP_000978691.1
938142	000487295.2	Senterica	WP_001100652.1
938142	000487295.2	Senterica	WP_001292402.1
938142	000487295.2	Senterica	WP_023237313.1
938142	000487295.2	Senterica	WP_023237541.1
941193	000226155.1	Paeruginosa	WP_014603046.1
941193	000226155.1	Paeruginosa	WP_014603098.1
94122	000203935.1	Ssp	WP_011718604.1
941323	000968515.1	Ecoli	WP_000839808.1
941323	000968515.1	Ecoli	WP_000978651.1
941323	000968515.1	Ecoli	WP_001020973.1
941323	000968515.1	Ecoli	WP_001295383.1
941639	000217835.1	Bcoagulans	WP_013857976.1
941639	000217835.1	Bcoagulans	WP_013858924.1
941968	000253195.1	Cbotulinum	WP_014519453.1
941968	000253195.1	Cbotulinum	WP_014521757.1
94624	000067205.1	Bpetrii	WP_012250976.1
946483	000477435.1	Csymbiobacter	WP_022771308.1
95486	000755725.1	Bcenocepacia	WP_009690200.1
95486	000755725.1	Bcenocepacia	WP_040131222.1
95486	000764955.1	Bcenocepacia	WP_040140538.1
95486	000764955.1	Bcenocepacia	WP_040141407.1
95486	001484665.1	Bcenocepacia	WP_006488824.1
95486	001484665.1	Bcenocepacia	WP_006488848.1
95486	001484665.1	Bcenocepacia	WP_006497175.1
956149	000339015.1	Csakazakii	WP_004385449.1
956149	000339015.1	Csakazakii	WP_004387170.1
956149	000339015.1	Csakazakii	WP_015386340.1
96241	000816805.1	Bsubtilis	WP_003218293.1
96241	000816805.1	Bsubtilis	WP_003221301.1
964	001040945.1	Hseropedicae	WP_013235395.1
96563	000219605.1	Pstutzeri	WP_013982902.1
98228	000829195.1	Bsp	WP_041072445.1

98228	000829195.1	Bsp	WP_041075949.1
983594	001411495.1	Bcepacia	WP_021163792.1
983594	001411495.1	Bcepacia	WP_027789577.1
983917	000284255.1	Rgelatinosus	WP_043785423.1
984129	000195535.1	Fcf	WP_014549442.1
984211	000487575.2	Senterica	WP_000978690.1
984211	000487575.2	Senterica	WP_001100652.1
984211	000487575.2	Senterica	WP_001292402.1
984211	000487575.2	Senterica	WP_023243687.1
984211	000487575.2	Senterica	WP_024155456.1
984892	000189495.1	Spseudinterme	WP_014614824.1
985002	000236925.1	Sargenteus	WP_000812826.1
985006	000237265.1	Saureus	WP_000812849.1
985079	000959525.1	Bmultivorans	WP_006410262.1
985665	000235585.1	Pterrae	WP_014279019.1
985665	000235585.1	Pterrae	WP_014282344.1
985762	001442815.1	Sagnetis	WP_060552288.1
988812	001046955.1	Malgicola	WP_048530733.1
990282	000213635.1	Senterica	WP_000839738.1
990282	000213635.1	Senterica	WP_000978690.1
990282	000213635.1	Senterica	WP_001021054.1
990282	000213635.1	Senterica	WP_001100652.1
990282	000213635.1	Senterica	WP_001292402.1
991791	000218855.1	Cacetobutylic	WP_010963620.1
991791	000218855.1	Cacetobutylic	WP_010965635.1
99287	000006945.1	Senterica	NP_459239.1
99287	000006945.1	Senterica	NP_459686.1
99287	000006945.1	Senterica	NP_461494.1
99287	000006945.1	Senterica	NP_462030.1
99287	000006945.1	Senterica	NP_463161.1
994476	000192105.1	Yenterocoliti	WP_005157162.1
994476	000192105.1	Yenterocoliti	WP_013649152.1
994476	000192105.1	Yenterocoliti	WP_013649386.1
994476	000192105.1	Yenterocoliti	WP_013650370.1
99598	000316575.1	Csp	WP_015127386.1
996285	000195105.1	Pstutzeri	WP_014596789.1
996633	000828595.1	Senterica	WP_000839738.1
996633	000828595.1	Senterica	WP_000978690.1
996633	000828595.1	Senterica	WP_001021054.1
996633	000828595.1	Senterica	WP_001100652.1
996633	000828595.1	Senterica	WP_023972725.1
997761	000258535.2	Pmucilaginosu	WP_014371513.1
997761	000258535.2	Pmucilaginosu	WP_014653035.1

998088	000204115.1	Averonii	WP_005349824.1
998088	000204115.1	Averonii	WP_005356279.1
998092	000194075.3	Mbovis	AHM08292.1
998820	000194765.1	Lcasei	WP_012491659.1
999378	000194785.1	Lcasei	WP_012491659.1
999541	000194745.1	Bgladioli	WP_013699054.1
999552	000511355.1	Lmethylohalid	WP_024092062.1
999891	000195515.1	Bamyloliquefa	WP_013350743.1
999891	000195515.1	Bamyloliquefa	WP_014470448.1

Supplementary Table 6. Primers used in this study.

Primer name	5'-3' sequences	Specific features
LdcF cloning and sequencing in pDONR201		
FTN_0504F	**GGGGACAAGTTTGTACAAAAAAGCAGGCT**TAGAAAACCTGTACTTCCAGGGTAAAACTGTTGTATTTGTCTATAAAG	**attB1 site**, TEV site
FTN_0504R	**GGGGACCACTTTGTACAAGAAAGCTGGGT**CTTATTATTTATCATCAATAACTTTGATATAGAGC	**attB2 site**, STOP codons
FTN0504-759F	GATGATGGTTGATGTTAACCC	
FTN0504-1376F	ATAATATCTCGAATAAAGAGGC	
KO construction, cloning and screening		
FTN_0504ForUp	ATTGCTAAATGTTTTAGGTGG	
FTN_0504RevUp	gcttatcgataccgtcgacctcCATAATTTTACGACTTAGAAAAATAACTAC	Overlap with Kana cassette
FTN_0504DownF	gatatcgatcctgcagctatgcTAATATCTTATTTTTTGAAATAAAATGC	Overlap with Kana cassette
FTN_0504DownRev	TAACCAGTACGTGCTACCATCC	
Primer OH234 (F)	gaggtcgacggtatcgataagc	Kana cassette
Primer OH235 (R)	gcatagctgcaggatcgatatc	Kana cassette
FTN_0504UpSeqF	ATGAATGATGTTGTTTTAAGTTGC	
FTN_0504DownSeqR	TAACCCTGCAGGTTGTGCTCC	
Complementation plasmid construction (pFNLTP6-ldcF) and sequencing		
Ldc_FnoF_NotI	*aattaa***GCGGCCGC**ATGAAAACTGTTGTATTTGTC	**NotI site**
Ldc_FnoR_AgeI	*ttaattaa***ACCGGT**TTATTTATCATCAATAACTTTGATATAGAGC	**AgeI site**
LDC340F	TGATGCTTTAGCTGGTGAGG	
LDC604F	AAGCACATAAGGACGCTG	
LDC910F	AAGTGGCCTGAATATGCGG	
pFNLT6_3925F	AATATCTAGACTTGCAAGAGCTTGG	
pFNLT6_5403R	AATGCACGCAAATACATACCTGCC	
